# The RNA helicase DDX6 regulates cell-fate specification in neural stem cells via miRNAs

**DOI:** 10.1093/nar/gkv138

**Published:** 2015-02-26

**Authors:** Sarah Nicklas, Satoshi Okawa, Anna-Lena Hillje, Laura González-Cano, Antonio del Sol, Jens C. Schwamborn

**Affiliations:** 1 Stem Cell Biology and Regeneration Group, Institute of Cell Biology, ZMBE, Westfälische Wilhelms-Universität Münster, D-48149 Münster, Germany; 2 Developmental and Cellular Biology Group, Luxembourg Centre for Systems Biomedicine (LCSB), University of Luxembourg, L-4362 Esch-Belval, Luxembourg; 3 Computational Biology Group, Luxembourg Centre for Systems Biomedicine (LCSB), University of Luxembourg, L-4362 Esch-Belval, Luxembourg

## Abstract

In neural stem cells (NSCs), the balance between stem cell maintenance and neuronal differentiation depends on cell-fate determinants such as TRIM32. Previously, we have shown that TRIM32 associates with the RNA-induced silencing complex and increases the activity of microRNAs such as Let-7a. However, the exact mechanism of microRNA regulation by TRIM32 during neuronal differentiation has yet to be elucidated. Here, we used a mass spectrometry approach to identify novel protein–protein interaction partners of TRIM32 during neuronal differentiation. We found that TRIM32 associates with proteins involved in neurogenesis and RNA-related processes, such as the RNA helicase DDX6, which has been implicated in microRNA regulation. We demonstrate, that DDX6 colocalizes with TRIM32 in NSCs and neurons and that it increases the activity of Let-7a. Furthermore, we provide evidence that DDX6 is necessary and sufficient for neuronal differentiation and that it functions in cooperation with TRIM32.

## INTRODUCTION

Neural stem cells (NSCs) have the ability to either self-renew or to give rise to different neural lineages, including neurons, astrocytes and oligodendrocytes (1). The process of generating functional neurons from NSCs is called neurogenesis. Neurogenesis occurs at a high level during mouse embryonic brain development, with NSCs giving rise to all the neurons of the central nervous system (2). In the adult brain, neurogenesis is restricted to two neurogenic niches: the subventricular zone of the lateral ventricles and the subgranular zone of the hippocampus (1). It has been shown that neurogenesis is not only relevant for brain function in mice (3) but also occurs in the adult brains of songbirds (4), monkeys (5) and humans (6–8).

The progression from NSCs to mature neurons is tightly regulated by various signaling pathways and a complex interplay of protein-coding and non-coding RNAs. One highly conserved class of non-coding RNAs are microRNAs (miRNAs), which are endogenously encoded, short (20–24 nt), single-stranded RNA molecules that post-transcriptionally regulate gene expression (9,10). To perform their regulatory functions, miRNAs are incorporated into the RNA-induced silencing complex (RISC), the major components of which are Argonaute proteins (Ago). MicroRNAs guide RISC to target mRNAs by complementary base-pairing with their 3′ untranslated regions (3′ UTRs) to mediate translational repression, mRNA degradation or cleavage (11–13).

During neuronal differentiation, miRNAs are temporally and spatially expressed and act as important regulatory switches that control the balance between stem cell maintenance and neuronal differentiation (14–16). Many miRNAs are specifically enriched within the mammalian brain, where they not only exert global effects such as the induction of neuronal differentiation but also function locally at the growth cone or at synapses (17). Furthermore, altered miRNA function or expression in NSCs has been associated with several neurological disorders, such as Parkinson's or Alzheimer's disease (18,19).

One key regulator of neuronal differentiation is the Let-7 family of microRNAs, which is highly conserved across species in both sequence and function (20). Let-7 members become upregulated during mouse brain development and their expression levels dramatically increase upon neuronal differentiation of NSCs (20,21). Consistent with this, overexpressing the Let-7 family member Let-7a in NSCs has been shown to promote neuronal differentiation, whereas Let-7a inhibition preserves their NSC fate (22). The dynamic expression pattern of miRNAs necessitates their tight regulation during the course of differentiation. However, little is known about the upstream regulators of miRNAs.

One of the regulators of Let-7a activity is the neuronal cell-fate determinant TRIM32 (22). TRIM32 belongs to the TRIM-NHL family of proteins that is characterized by the presence of an N-terminal RING finger, one or two B boxes, a coiled-coil region and a C-terminal NHL domain (23). This conserved protein family has been implicated in diverse biological processes, such as developmental timing, cell cycle progression, transcriptional regulation, apoptosis and signaling pathways (24). Previously, we have shown that TRIM32 suppresses proliferation and induces neuronal differentiation in NSCs of the embryonic (22,25,26) and adult mouse brain (27), as well as muscle differentiation in adult muscle stem cells (28). TRIM32 exerts its effect via two mechanisms. Through its N-terminal RING finger, TRIM32 ubiquitinates the transcription factor c-Myc, thereby targeting it for proteasomal degradation and inducing cell-cycle exit (22,25,29). Additionally, through its C-terminal NHL domain, TRIM32 directly binds the RISC protein Ago1, which leads to enhanced activity of specific microRNAs including Let-7a (22). However, the exact mechanism by which TRIM32 regulates microRNAs to promote neuronal differentiation remains elusive.

Interestingly, TRIM-NHL proteins have also been described as RISC cofactors during the regulation of cell fate choices in other species, such as *Drosophila* and *Caenorhabditis elegans* (30,31). Similar to its mammalian homolog TRIM32, *C. elegans* NHL-2 has been shown to enhance the activity of the Let-7 family of microRNAs during progenitor cell differentiation (31). NHL-2 colocalizes and directly interacts with the RNA helicase CGH-1 and this interaction is thought to be responsible for the enhanced activity of associated microRNAs (31,32).

Here, we used a mass spectrometry approach to identify novel potential TRIM32-associated proteins that may play a role during neuronal differentiation. By applying bioinformatics tools, we identified an enrichment of proteins involved in neurogenesis and RNA-related processes among the potential TRIM32-associated proteins. One candidate discovered in this screen was the RNA helicase DDX6, which is the mammalian homolog of CGH-1 (33,34). We demonstrate, that also in the mammalian system, TRIM32 and DDX6 colocalize in NSCs and neurons and that DDX6 regulates the activity of the microRNA Let-7a. Consistent with these data, overexpression of DDX6 in NSCs leads to an increase in neuronal differentiation, whereas loss of DDX6 function inhibits the generation of new neurons. Because this inhibition is even more pronounced in the absence of TRIM32, we conclude that both proteins cooperate during the induction of neuronal differentiation in NSCs. Furthermore, both effects of DDX6, the stimulation of Let-7a activity and the induction of neuronal differentiation, appear to depend on its helicase activity. In summary, we present here a novel protein–protein interaction network centered on the RNA-regulating proteins TRIM32 and DDX6, which is involved in the process of NSC differentiation.

## MATERIALS AND METHODS

### Reagents and plasmids

For immunolabeling and protein biochemical methods, the following antibodies were used: anti-TRIM32-3150 (Gramsch Laboratories), anti-TRIM32-3149 (Gramsch Laboratories), anti-TRIM32-M09 (Abnova), anti-TRIM32-GS (Genescript), anti-TRIM32-1137 (Gramsch Laboratories) (25,27,28), anti-DDX6 (Abcam), anti-Ago2/eIF2C2 (Abcam), anti-Ago2 (Sigma), anti-Pumilio1 (anti-Pum1) (Abcam), anti-FLAG M2 (Sigma), anti-glyceraldehyde 3-phosphate dehydrogenase (anti-GAPDH) (Abcam), anti-enhanced green fluorescent protein (anti-EGFP) (Abcam), anti-Sox2 (Abcam), anti-Ki67 (BD Pharmingen), anti-phospho-Histone 3 (anti-PH3) (Millipore), anti-Tuj1 (Covance), anti-MAP2 (Millipore), anti-GFAP (Millipore) and anti-S100β (Sigma). Alexa-fluorophore-conjugated antibodies (Invitrogen) and horseradish peroxidase-coupled antibodies (GE Healthcare) were used as secondary antibodies for immunofluorescence staining and for immunoblotting, respectively. DNA was counterstained using Hoechst 33258 (Invitrogen).

The following plasmids were used: pEGFP-N1 (Clontech Laboratories), pEGFP-TRIM32 (23), pIRESneo-FLAG/HA-EGFP, pIRESneo-FLAG/HA-Ago1, pIRESneo-FLAG/HA-Ago2, pIRESneo-FLAG/HA-Ago3 and pIRESneo-FLAG/HA-Ago4-FLAG-HA, pcDNA3.1D-Firefly-Let-7a with four perfectly matched Let-7a binding sites (Let-7a-Sensor), pcDNA3.1D-Firefly-Let-7a-mut with mismatches in the Let-7a binding sites (Mut-Let-7a-Sensor), pcDNA3.1D-Let-7a-1 (all kindly provided by Dr Sven Diederichs); Venus1C-FLAG-DDX6 (kindly provided by Dr Dirk H. Ostareck); pFLAG-CMV2-DDX6 (kindly provided by Dr Masayuki Matsushita) and pEYFP-DDX6-EQ (kindly provided by Dr Stanley M. Lemon).

DDX6 knockdown was achieved with the commercially available shRNA-expression vectors pGFP-V-RS-DDX6-shRNA3 (AAGAGCCTGTATGTGGCAGAATACCACAG) (Origene). The same vector expressing a scrambled (scrbl.) sequence (GCACTACCAGAGCTAACTCAGATAGTACT) was used as a control (Origene).

### Mice

TRIM32 −/− mice were obtained from the Mutant Mouse Regional Resource Centers (MMRRC) (https://www.mmrrc.org/catalog/sds.php?mmrrc_id=11810/011810.html) (27). For all mice, breeding, maintenance and experimental procedures were performed in accordance with the German Federal law on the Care and Use of laboratory animals.

### Cell culture

Primary NSCs were isolated from single TRIM32 +/+ and TRIM32 −/− mouse brains at embryonic day (E) 12.5–14.5. NSCs were cultivated as neurospheres on uncoated polystyrene tissue culture dishes in DMEM/Ham's F12 medium (PAA) supplemented with 10 ng/ml EGF (Peprotech), 10 ng/ml bFGF-2 (Peprotech), 1× N2 (Invitrogen), L-Glutamine (PAA) and Penicillin/Streptomycin (PAA). For splitting, neurospheres were collected by centrifugation for 3 min at 900 rpm and mechanically dissociated into single cells by resuspending in maintenance medium. For immunocytochemical staining, neurospheres were seeded onto coverslips coated with poly-ornithine (Sigma) and Laminin (Sigma).

Neuronal differentiation was induced by exchanging 50% of the maintenance medium with DMEM/Ham's F12 medium (PAA) supplemented with 10 ng/ml bFGF-2 (Peprotech), 1× N2 (Invitrogen), 1× B27 (Invitrogen), L-Glutamine (PAA) and Penicillin/Streptomycin (PAA). For glial differentiation, the medium was changed completely to DMEM/Ham's F12 medium (PAA) supplemented with 1× N2 (Invitrogen), 1% heat-inactivated foetal calf serum (FCS) (PAA), L-Glutamine (PAA) and Penicillin/Streptomycin (PAA). Differentiation was induced for 5 days, after which cells were processed for immunocytochemical staining.

Neuroblastoma (N2a) cells, HEK293T cells and NIH3T3 cells were cultivated on uncoated 10-cm polystyrene tissue culture dishes in DMEM (Sigma) supplemented with 10% heat-inactivated FCS (PAA), L-Glutamine (PAA) and Penicillin/Streptomycin (PAA). For transfection, N2a cells were seeded onto poly-ornithine-coated 6-well polystyrene tissue culture plates 1 day prior to transfection, whereas HEK293T cells were seeded onto poly-D-Lysine-coated (Sigma) 10-cm polystyrene tissue culture dishes and NIH3T3 cells were seeded onto poly-D-Lysine-coated coverslips.

### Transfection

Neurospheres were electroporated with 7.5 μg of the indicated plasmids using the Amaxa mouse NSC Nucleofector Kit (Lonza) and the Amaxa Nucleofector II Device (Lonza) according to the manufacturer's instructions.

N2a, HEK293T and NIH3T3 cells were transfected using Turbofect (Fermentas) according to the manufacturer's instructions. Each transfection experiment was repeated at least three independent times.

### Immunoprecipitation and western blot

For immunoprecipitation assays, HEK293T cells were lysed 48 h after transfection with lysis buffer (2% Triton X-100 and Complete protease inhibitor cocktail (PIC) (Roche) in phosphate-buffered saline (PBS)) for 30 min at 4°C. The cell lysates were centrifuged for 30 min at 13 000 rpm at 4°C and the protein concentrations of the supernatants were determined using a bicinchoninic acid (BCA) protein assay kit (Thermo Scientific) according to the manufacturer's instructions. After adjustment to equal protein concentrations, a fraction of the lysate was mixed with sample buffer and boiled at 95°C for 5 min. The remaining cell lysate was incubated with the precipitating antibody for 4 h at 4°C and then overnight with protein-G agarose beads (GE Healthcare). To digest endogenous RNAs using micrococcal nuclease, beads were resuspended in 100 μl lysis buffer containing 1 mM CaCl_2_ and 600 U micrococcal nuclease and incubated for 10 min at RT. As an alternative approach, beads were incubated with 10 μg of RNAse A (Invitrogen) for 30 min at 4°C. Bound proteins were eluted by boiling the samples for 15 min at 95°C in protein sample buffer. The beads were centrifuged for 5 min at 13 000 rpm and the supernatant was subjected to sodium dodecyl sulphate-polyacrylamide gel electrophoresis (SDS-PAGE) and western blotting.

For immunoprecipitation assays from brain tissues, E10.5–E14.5 or adult mouse brains were lysed for 1 h at 4°C in lysis buffer (2% Triton X-100 and PIC in PBS) that was supplemented with 120 μg/ml RNAse A and 6 μg/ml DNAse to reduce non-specific binding. The lysates were immunoprecipitated as described above. To further reduce non-specific binding, beads were pre-blocked for 1 h at 4°C with 1% BSA (Fraction V) (Roth) in PBS. All immunoprecipitation experiments were repeated at least three independent times.

For immunoblot analysis, IP samples and lysates were size-separated by electrophoresis on 4–12% NuPAGE Bis-Tris gradient gels according to the manufacturer's instructions (Invitrogen). After transfer to nitrocellulose membranes, equal loading of the blotted protein was verified by Ponceau S (Sigma) staining. Subsequently, membranes were blocked for 1 h at RT in 5% skimmed milk powder and 0.2% Tween in PBS before incubating overnight at 4°C with the primary antibodies. Horseradish peroxidase-conjugated secondary antibodies and enhanced chemiluminescence reagents (ECL kit) (GE Healthcare) were used for detection. Western blots were analyzed using ImageJ software.

### Immunocytochemistry

For immunocytochemical staining, cells were fixed with 4% paraformaldehyde in 120 mM PBS, pH 7.4 (4% PFA/PBS) followed by permeabilization for 3 min using 0.05% Triton X-100 in PBS. Next, cells were blocked with 10% FCS in PBS for 1 h at RT and subjected to immunofluorescence staining with primary and secondary antibodies diluted in blocking solution.

For cell counting, images were collected on a Zeiss epifluorescence microscope, whereas for colocalization analysis, images were collected using a Zeiss confocal microscope. Image analysis was performed using ZEN lite (Zeiss), Adobe Photoshop and ImageJ software. Colocalization analyses were conducted using the ImageJ Plugin JACoP as described previously (35).

### Luciferase assays

The activity of the microRNA Let-7a was determined using the Luciferase Assay System (Promega) according to the manufacturer's instructions. Briefly, N2a cells were transfected with 4 μg of DNA in 6-well tissue culture plates. Each transfection was performed twice with either the Let-7a-Sensor or the Mut-Let-7a-Sensor (36) cotransfected with pcDNA3.1D-Let-7a-1 and an empty vector, EGFP, TRIM32-EGFP-Myc, DDX6-FLAG, mutant DDX6-EQ-EYFP, scrambled shRNA or DDX6 shRNA. After 72 h, cells were lysed in Passive Lysis Buffer (Promega). Luminescence measurements were performed in quadruplicate in 96-well plates containing 20 μl lysate per well. For each overexpression sample, the signal obtained from the WT-Let-7a-Sensor was normalized to that of the mutant-Let-7a-Sensor.

### Mass spectrometry analysis

#### Sample preparation

Stable cultures of NSCs were generated as previously described (37). NSCs were grown as monolayers under maintenance or neuronal-differentiation-inducing conditions as previously described (25). Cells were scraped in cold PBS containing PIC and centrifuged for 5 min at 1100 rpm at 4°C. The pellets from 22 10-cm dishes of proliferating cells and 25 10-cm dishes of differentiated cells were pooled and centrifuged with the identical settings. The resulting pellet was resuspended in 4–5 pellet volumes of buffer A (10 mM Hepes pH 7.6, 1.5 mM MgCl_2_, 10 mM KCl, 0.5 mM Dithiothreitol (DTT), 1× PIC), mixed by inversion and incubated on ice for 10 min before centrifuging for 10 min at 3000 rpm at 4°C. Whole-mouse brain tissue lysed in buffer A was used as a negative control. The pellet was resuspended in 2 pellet volumes of buffer A, homogenized using the loose pestle (pestle A) of a Dounce homogenizer (20 strokes) and centrifuged for 10 min at 3000 rpm at 4°C. The supernatant was saved as the cytoplasmic fraction, the pellet was centrifuged again for 1 min and the supernatant was discarded. The pellet was resuspended in 1.5 pellet volumes (volume from above) of buffer C (20 mM Hepes pH 7.6, 20% glycerol, 420 mM NaCl, 1.5 mM MgCl_2_, 0.2 mM ethylenediaminetetraacetic acid (EDTA), 0.5 mM DTT, 1 x PIC) homogenized using pestle B (20 strokes) of a Dounce homogenizer, rotated using an overhead rotator for 30 min at 4°C and centrifuged for 15 min at 13 000 rpm. The supernatant contained the nuclear fraction. The NaCl concentration of the nuclear fraction was adjusted from 420 to 150 mM using H_2_O and the nuclear and cytoplasmic fractions were incubated with the precipitation antibody for 4 h at 4°C (for TRIM32: 1137/3149 (Gramsch Laboratories); for control: EGFP (Abcam)). A total of 100 μl of protein-G Sepharose beads (GE Healthcare) were washed four times in non-stick microcentrifuge tubes (Alpha laboratories) with buffer C-100* (20 mM Hepes pH 7.6, 20% glycerol, 100 mM KCl, 1.5 mM MgCl_2_, 0.2 mM EDTA, 0.02% NP40, 0.5 mM DTT, 1 x PIC) and 1.5 ml of the appropriate fraction was added. One twenty-fifth volume of 25× PIC and 25 U/μl Benzonase (Novagen) were added and the samples were incubated on an overhead shaker at 4°C overnight. The next day, the beads were washed five times with 700 μl buffer C-100*. The bound TRIM32 protein was eluted using equal amounts of two TRIM32 peptides (1137: MESFTEEQLRPKLLH; 3149: Cys-VKIYSYHLRRYSTP-COOH (Metabion)) diluted in buffer C-100* at a total concentration of 0.2 mg/ml. A total of four elutions were performed. For the first elution, 70 μl of peptide was added, incubated for 30 min at 4°C and centrifuged for 2 min at 2000 rpm. A total of 65 μl of the supernatant was collected and centrifuged for 1 min at 2000 rpm to remove bead contamination. For the second–fourth elution steps, 60 μl of peptide was added to the beads and the elution was performed as described for elution 1. The fractions were pooled, and the proteins were concentrated by TCA precipitation and analyzed by mass spectrometry.

#### Mass spectrometry

The identification of proteins by mass spectrometry was performed as previously described (38). In summary, SDS-PAGE gel lanes were cut into slices and subjected to in-gel reduction with dithiothreitol, alkylation with iodoacetamide and digestion with trypsin, essentially as described by (39). Nanoflow LC-MS/MS was performed on an 1100 series capillary LC system (Agilent Technologies) coupled to an LTQ-Orbitrap mass spectrometer (Thermo) operating in positive mode and equipped with a nanospray source. Peptide mixtures were trapped on a ReproSil C18 reversed phase column. Peptide separation was performed on ReproSil C18 reversed phase column using a linear gradient from 0 to 80% B (*A* = 0.1% formic acid; *B* = 80% (v/v) acetonitrile, 0.1% formic acid) in 70 min and at a constant flow rate of 200 nl/min using a splitter. The column eluent was directly sprayed into the ESI source of the mass spectrometer. Mass spectra were acquired in continuum mode; fragmentation of the peptides was performed in data-dependent mode. Peak lists were automatically created from raw data files using the Mascot Distiller software (version 2.1; MatrixScience). The Mascot search algorithm (version 2.2, MatrixScience) was used for searching against the NCBInr database (release NCBInr_20090222; taxonomy: Mus musculus) or the IPI_mouse_database (release 20090924). The peptide tolerance was typically set to 10 ppm and the fragment ion tolerance to 0.8 Da. A maximum number of two missed cleavages by trypsin were allowed and carbamidomethylated cysteine and oxidized methionine were set as fixed and variable modifications, respectively. The Mascot score cut-off value for a positive protein hit was set to 60, based on at least two peptides. In case of protein identifications with Mascot scores between 50 and 60, or that were based on only one peptide, individual peptide MS/MS spectra were checked manually and either interpreted as valid identifications or discarded.

#### Protein quantification

For both the differentiating and proliferating NSC samples, the spectral counts of the respective cytoplasmic and nuclear fractions were summed prior to quantification. We identified 1950 proteins in the differentiating NSC sample and 2266 proteins in the proliferating NSC sample, whereas 29 proteins were identified in the negative control. In total, 2641 unique proteins were identified in all samples. Proteins enriched in the NSC samples versus the negative control were identified using the one-sided Poisson significance test between the protein spectral count of the negative control and the mean spectral count of the two NSC samples. Given the relatively low sample complexity, the missing spectral count was assumed to be 0. Proteins with a *P*-value below 0.1 were considered significantly enriched in the NSC samples. The number of enriched proteins was 1407 at this significance level. Of these, 1274 proteins were identified in both NSC samples, which were used in the next step to assess the differential abundance of potential TRIM32-associated proteins between NSCs under maintenance conditions and after the induction of neuronal differentiation. The proteins were binned by intensity (log2-transformed spectral count) and the statistical significance was estimated in each bin with a robust *z*-test. In each bin, the log2 ratio of protein spectral counts was standardized and the *z*-score was computed after trimming the 2.5% outliers on both sides. The *z*-score of the trimmed data points was extrapolated from the *z*-score function. The *z*-score was then converted into the *P*-value. Proteins with a *P*-value below 0.1 and an absolute log2 fold-change above 0.5 were reported as differentially abundant proteins. We identified 118 proteins, including TRIM32 itself, to be significantly more abundant under differentiation conditions than under stem-cell-maintenance conditions. In contrast, 79 proteins were more abundant in NSCs under maintenance conditions.

#### Bioinformatic analysis

Gene Ontology Biological Process was downloaded from http://www.geneontology.org. Fisher's exact test was performed to identify the statistical enrichment of Gene Ontology (GO) categories using the 118 proteins that were more abundant in differentiating NSCs as the test set. Proteins identified in the negative control, differentiating NSCs and/or proliferating NSCs were taken as the background set. The *P*-value was corrected for multiple testing by the Benjamini–Hochberg method. Categories with an adjusted *P*-value below 0.1 were defined as significantly enriched. The mouse protein–protein interaction data of the biological process GO terms ‘positive regulation of neurogenesis’ and RNA-regulation-related processes were retrieved from MetaCore (GeneGo Inc. (40)) with the direct interactions algorithm. Non-physical interaction types such as ‘transcription regulation’ and ‘transport’ were discarded. The networks were loaded in Cytoscape (41) and proteins enriched in the NSC samples, as well as their first neighbors, were visualized.

## RESULTS

### Identification of DDX6 and Argonaute 2 as novel TRIM32 interaction partners

**Figure 1.**
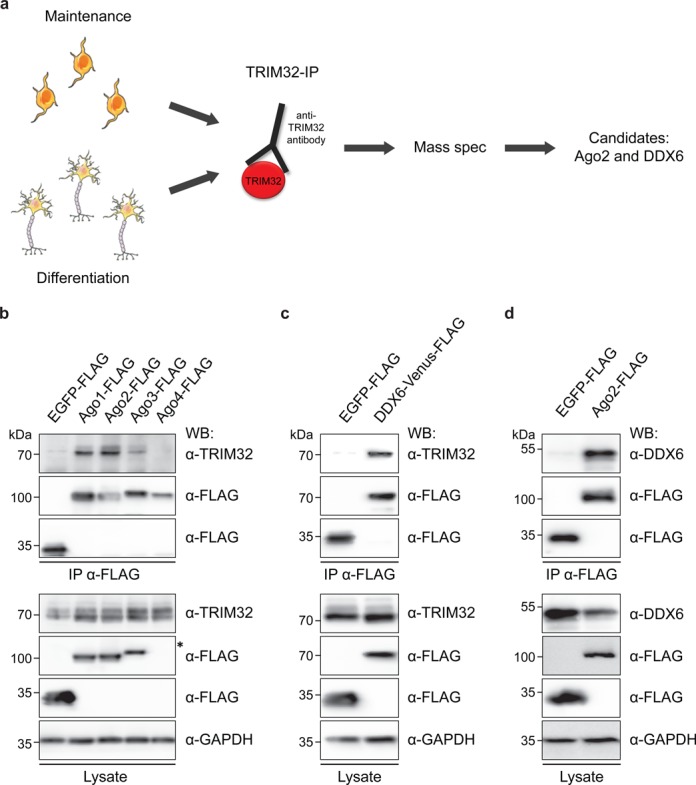
Identification of Ago2 and DDX6 as novel TRIM32-associated proteins via mass spectrometry. (**a**) Scheme depicting the experimental design of the mass spectrometry approach. TRIM32 was precipitated from NSCs either grown under maintenance conditions or 3 days after the induction of neuronal differentiation using an anti-TRIM32 antibody. Proteins that coprecipitated with TRIM32 were analyzed by mass spectrometry, which identified Ago2 and DDX6 as potential TRIM32 interaction partners (the full list of identified proteins is provided in Supplementary Table S1). (**b**) HEK293T cells were transfected with the indicated constructs and the different FLAG-tagged Ago proteins were immunoprecipitated with an anti-FLAG antibody. Ago-associated TRIM32 was detected with an anti-TRIM32 antibody. TRIM32 and FLAG-tagged Ago proteins were detected with specific antibodies as indicated for the immunoprecipitation (IP) and the lysate. Note that although Ago4 was detectable after enrichment via precipitation, no Ago4-expression could be detected within the lysate (asterisk). (**c**) HEK293T cells were transfected with the indicated constructs and Venus-FLAG-tagged DDX6 was immunoprecipitated with an anti-FLAG antibody. DDX6-associated TRIM32 was detected with an anti-TRIM32 antibody. TRIM32 and Venus-FLAG-tagged DDX6 were detected with specific antibodies as indicated for the IP and the lysate. (**d**) HEK293T cells were transfected with the indicated constructs and FLAG-tagged Ago2 was immunoprecipitated with an anti-FLAG antibody. Ago2-associated DDX6 was detected with an anti-DDX6 antibody. DDX6 and FLAG-tagged Ago2 were detected with specific antibodies as indicated for the IP and the lysate. In (b–d), GAPDH western blots were used as loading control.

Previously, we have shown that the cell-fate determinant TRIM32 induces neuronal differentiation in mouse NSCs by increasing the activity of particular microRNAs (22,25,27). Therefore, TRIM32 was an excellent starting point to identify novel neurogenesis- and RNA-regulating proteins. However, the exact mechanism of microRNA-regulation by TRIM32 during neuronal differentiation has yet to be elucidated. We therefore used a mass spectrometry approach to identify novel interaction partners of TRIM32 in NSCs (38). To this end, TRIM32 was precipitated from NSCs cultured under maintenance conditions, as well as under neuronal differentiation-inducing conditions (37) and the proteins that coprecipitated with TRIM32 were analyzed by mass spectrometry (Figure 1a, Supplementary Figures S1 and S2, Supplementary Table S1). After filtering for potential interaction candidates that were significantly enriched in the NSC samples versus the negative control (Supplementary Figure S1a), we performed a gene set enrichment analysis of biological process GO terms of those proteins that were significantly more abundant under differentiation conditions than under stem-cell-maintenance conditions (Supplementary Figure S1b–d; Supplementary Table S2). Consistent with our previous results (22,25,27), many potential TRIM32-interacting proteins were associated with the term ‘positive regulation of neurogenesis’ (Supplementary Figure S1d and e; Supplementary Table S2; Supplementary Table S3), underscoring the role of TRIM32 in neuronal differentiation. Interestingly, many GO categories associated with the regulation of RNA were enriched (Supplementary Figure S1d, Supplementary Figure S2, Supplementary Table S2; Supplementary Table S3).

One interesting candidate identified by this approach is the Argonaute 2 (Ago2) protein, which is part of RISC (Figure 1a, Supplementary Figure S2, Supplementary Table S1; Supplementary Table S3). Because we have already described an interaction between TRIM32 and the Argonaute 1 protein (22), we extended our analysis to the entire family of Ago, which consists of four members in mammals. Therefore, we overexpressed FLAG-tagged Ago1-4 in HEK293T cells and precipitated the proteins using an anti-FLAG antibody. Using this approach, we were able to coimmunoprecipitate TRIM32 together with Ago1, 2 and 3, but not with Ago4 (Figure 1b). However, because only low expression levels of Ago4 were observed in the lysates (see asterisk in Figure 1b), any potential interaction with TRIM32 may have been undetectable. Given that our mass spectrometry approach identified Ago2 as a potential TRIM32 interaction partner in proliferating and differentiating NSCs and because Ago2 has been well studied for its function in post-transcriptional gene silencing, we focused on this family member for the following experiments.

Another interesting candidate revealed by the mass spectrometry analysis is the RNA helicase DDX6 (Figure 1a, Supplementary Figure S2, Supplementary Table S1; Supplementary Table S3), whose *C. elegans* homolog CGH-1 was shown to interact with the TRIM32 homolog NHL-2 (31). Overexpressed DDX6-Venus-FLAG in HEK293T cells coimmunoprecipitated with TRIM32, confirming that this interaction is conserved between the mammalian homologs (Figure 1c). Finally, the precipitation of overexpressed FLAG-tagged Ago2 from HEK293T cells showed that Ago2 and DDX6 also form a complex (Figure 1d).

To determine whether these interactions occur directly or are mediated by RNA, lysates were treated with Micrococcal nuclease (MNase) or RNAse A prior to immunoprecipitation, leading to the preferential degradation of single-stranded RNA. The different proteins were still able to bind one another, albeit more weakly in the case of the interactions between Ago2 and TRIM32 or DDX6 (Supplementary Figure S3a-c and data not shown). Therefore, we conclude that the interaction between TRIM32 and DDX6 is RNA independent, while the interactions between Ago2 and TRIM32 as well as Ago2 and DDX6 are partially RNA dependent.

**Figure 2.**
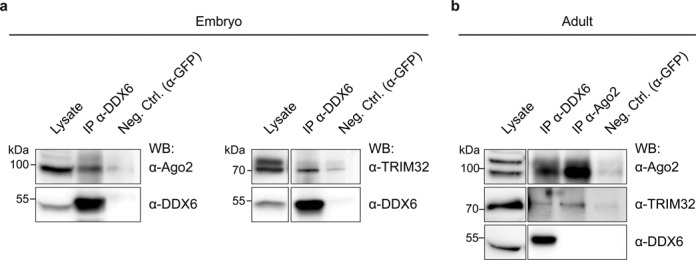
TRIM32, Ago2 and DDX6 directly interact with each other *in vivo*. (**a**) Immunoblots of IPs from E10.5 to E14.5 embryonic brain lysates using antibodies against DDX6 or GFP (negative control). DDX6, TRIM32 and Ago2 were detected with the indicated specific antibodies in the IP and lysate. (**b**) Immunoblots of IPs from adult brain lysates using antibodies against DDX6, Ago2 or GFP (negative control). DDX6, TRIM32 and Ago2 were detected with the indicated specific antibodies in the IP and lysate. Abbreviations: EGFP, enhanced green fluorescent protein; Ago, Argonaute; IP, immunoprecipitation; WB, western blot; Neg. Ctrl., negative control.

To further investigate whether these interactions also occur *in vivo*, DDX6 was precipitated from mouse embryonic brain lysates, which contain a large fraction of undifferentiated NSCs. Using this approach, we were able to detect the binding of both Ago2 and TRIM32 to DDX6 (Figure 2a), suggesting that these proteins form a complex *in vivo* in the embryonic brain. These interactions were found to persist in the adult brain (Figure 2b) which primarily consists of differentiated cells. Thus, TRIM32, Ago2 and DDX6 may interact with one another in both undifferentiated and differentiated cells. Interestingly, we also detected the binding of all three proteins to the RNA-binding protein Pumilio 1 (Pum1) *in vivo* (Supplementary Figure S3d and e). Pum1 has recently been implicated in the regulation of mRNAs via microRNAs in *C. elegans* (42), and in *Drosophila*, Pum1 has been shown to interact with the TRIM32 homolog Brat, thereby increasing its activity (43). Finally, Pum1 was also detected in our mass spectrometry analysis (Supplementary Table S1), further underscoring the quality of the obtained data.

### TRIM32, DDX6 and Ago2 colocalize

To analyze the subcellular localization of TRIM32, DDX6 and Ago2, we first overexpressed the recombinant proteins in NIH3T3 cells, which are well suited for localization analysis because of their flat morphology. All three proteins exhibited diffuse staining throughout the cytoplasm and were strongly enriched in cytoplasmic punctae (Supplementary Figure S4). Interestingly, when overexpressing TRIM32 and Ago2, TRIM32 and DDX6, or Ago2 and DDX6, two different populations of cytoplasmic punctae were observed: those in which both proteins colocalized and those in which both proteins appeared to exclude each other. However, when measured over the entire cell, colocalization was observed in each case (Supplementary Figure S4).

**Figure 3.**
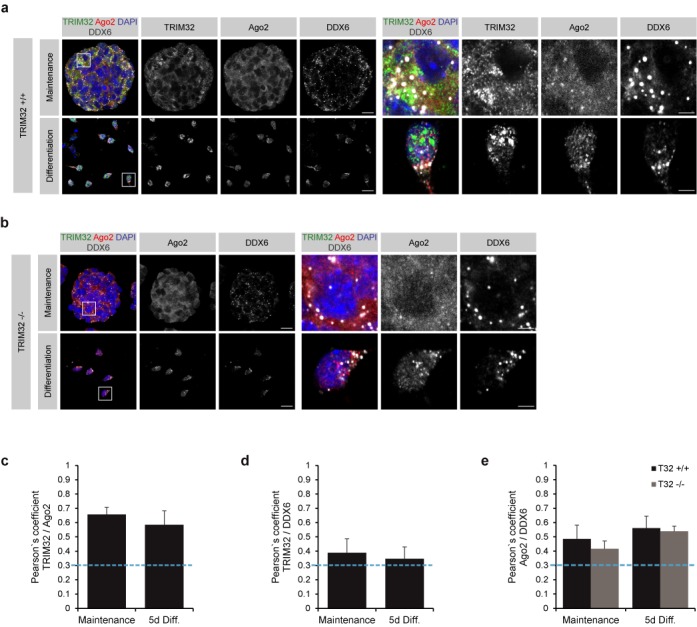
TRIM32, Ago2 and DDX6 colocalize in NSCs and neurons. (**a** and **b**) Immunostaining of neurospheres derived from TRIM32 +/+ (a) and TRIM32 −/− (b) mice, cultured under maintenance conditions and 5 days after the induction of neuronal differentiation, with the indicated markers. Single optical planes are shown. The right panels show a higher magnification of the boxed areas. Scale bars = 20 μm; for high magnifications, 5 μm. (**c**–**e**) Diagrams showing Pearson's coefficients for the colocalization of TRIM32 and Ago2 (**c**), TRIM32 and DDX6 (**d**) and Ago2 and DDX6 (**e**) in NSCs and neurons. Colocalization was defined as a Pearson's coefficient ≥0.3 (blue dotted line) (mean ± SEM; *n* ≥ 20 cells, *N* = 3 independent TRIM32 +/+ and TRIM32 −/− neurosphere cultures).

To extend the colocalization analysis to the endogenous proteins, we generated neurosphere cultures from E12.5–E14.5 TRIM32 +/+ and −/− embryos and analyzed the subcellular distribution of TRIM32, DDX6 and Ago2 under maintenance and neuronal differentiation conditions. Under maintenance conditions, TRIM32, DDX6 and Ago2 localized to the cytoplasm of TRIM32 +/+ neurospheres and were found to colocalize with each other (Figure 3a, c–e, Supplementary Figure S5a and c). Under neuronal differentiation conditions, nuclear translocation of TRIM32 was observed (Figure 3a, Supplementary Figure S5a and c), consistent with our previous results (25,27). Interestingly, also a pool of Ago2 translocated to the nucleus upon differentiation, while DDX6 remained primarily in the cytoplasm (Figure 3a, Supplementary Figure S5a and c). However, all three proteins were also observed to colocalize under these conditions (Figure 3c–e, Supplementary Figure S5a and c). A similar distribution of Ago2 and DDX6 was observed in TRIM32 −/− neurospheres, with both proteins localizing to the cytoplasm under maintenance conditions, while a pool of Ago2 localized to the nucleus under neuronal differentiation conditions (Figure 3b, Supplementary Figure S5b and d). We did not detect any difference in the degree of colocalization of DDX6 and Ago2 in the absence or presence of TRIM32 (TRIM32 +/+ versus −/− cells) (Figure 3e, Supplementary Figure S5b and d).

### Neuronal differentiation is impaired in TRIM32 −/− NSCs

**Figure 4.**
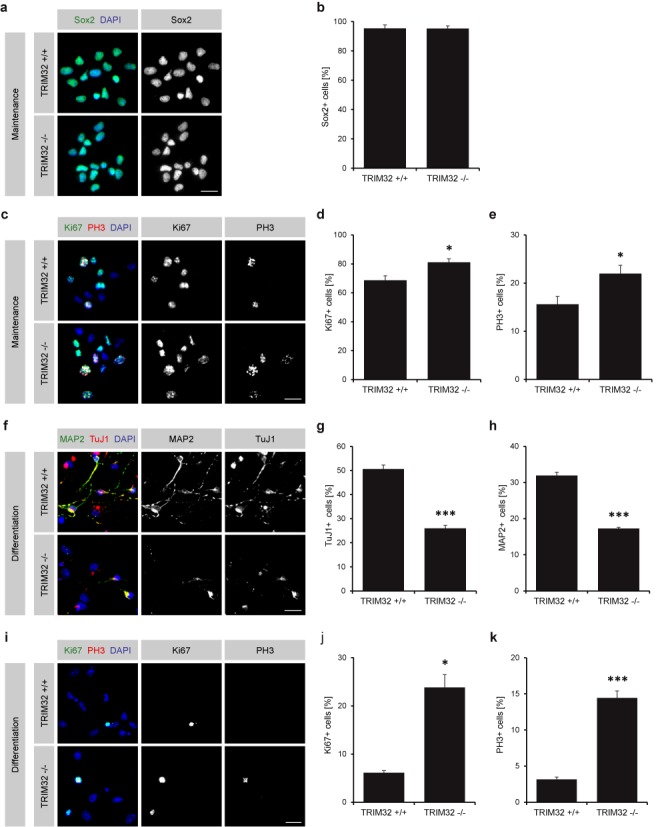
Absence of TRIM32 results in increased proliferation and reduced neuronal differentiation of NSCs. (**a** and **c**) Immunostaining of neurospheres derived from TRIM32 +/+ and TRIM32 −/− mice cultured under maintenance conditions with the indicated markers. Scale bars = 20 μm. (**b**, **d** and **e**) Quantification of the percentage of TRIM32 +/+ and −/− NSCs positive for the NSC marker Sox2 (b), the proliferation marker Ki67 (d) and the mitotic marker PH3 (e) (mean ± SEM; *n* ≥ 1200 cells, *N* = 4 independent TRIM32 +/+ and −/− neurosphere cultures; *t*-test, *P < 0.05 compared to TRIM32 +/+). (**f** and **i**) Immunostaining of neurospheres derived from TRIM32 +/+ and TRIM32 −/− mice 5 days after the induction of neuronal differentiation with the indicated markers. Scale bars = 20 μm. (**g**, **h**, **j** and **k**) Quantification of the percentage of TRIM32 +/+ and −/− neurons positive for the young neuronal marker TuJ1 (g), the mature neuronal marker MAP2 (h), the proliferation marker Ki67 (j) and the mitotic marker PH3 (k) (mean ± SEM; *n* ≥ 1400 cells, *N* ≥ 3 independent TRIM32 +/+ and −/− neurosphere cultures; *t*-test (TuJ1, MAP2, PH3), ***P < 0.005; Mann–Whitney U test (Ki67), *P < 0.05 compared to TRIM32 +/+).

Because TRIM32 has been described as necessary for neuronal differentiation in the embryonic (22,25) and adult mouse brain (27), we investigated the differentiation potential of TRIM32 +/+ and −/− neurospheres. We first investigated neurospheres growing under maintenance conditions. Almost all TRIM32 +/+ and −/− NSCs were positive for the NSC marker Sox2 (Figure 4a and b), indicating that the loss of TRIM32 has no effect on the maintenance of stem cell fate in NSCs. However, in TRIM32 −/− NSCs, a significant increase in the amount of mitotically active (Ki67-positive) and dividing (PH3-positive) cells was detected (Figure 4c–e), demonstrating that proliferation is increased upon loss of TRIM32 under maintenance conditions.

We then differentiated TRIM32 +/+ and −/− NSCs into neurons for 5 days. At this time point, ∼50% of the TRIM32 +/+ cells had upregulated the young neuronal marker TuJ1 and almost 32% had started to express the more mature neuronal marker MAP2 (Figure 4f-h). In contrast, only 26 and 17% of the TRIM32 −/− cells had differentiated into TuJ1- and MAP2-positive cells, respectively (Figure 4f–h). In addition, the effect on proliferating cells became more pronounced upon the induction of neuronal differentiation. Whereas only a few proliferating cells were detected among the TRIM32 +/+ cells 5 days after differentiation, a strong increase in the amount of Ki67- and PH3-positive cells was detected in the TRIM32 −/− cells (Figure 4i–k, Supplementary Figure S6a). These results demonstrate that neuronal differentiation in neurospheres is impaired following the loss of TRIM32. Remarkably, the differentiation-inducing potential of TRIM32 appears to be restricted to the neuronal lineage, as indicated by the fact that glial differentiation was unaffected upon loss of TRIM32 (Supplementary Figure S6b–e).

### DDX6 stimulates the activity of the microRNA Let-7a

**Figure 5.**
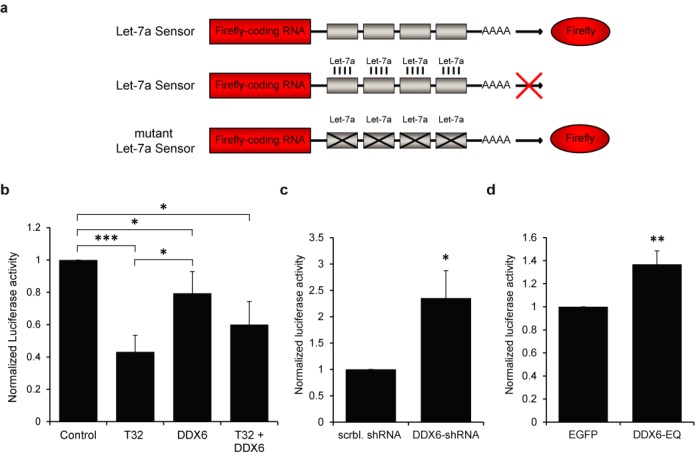
DDX6 induces the activity of the microRNA Let-7a. (**a**) Scheme depicting the luciferase sensors used in the luciferase assays. A Let-7a sensor was used that encodes firefly luciferase followed by Let-7a binding sites in its 3′ UTR. Luciferase is expressed in the absence of Let-7a and its activity can be measured via luciferase assays. In contrast, in the presence of Let-7a, luciferase expression is post-transcriptionally inhibited by binding of Let-7a to its binding sites. A mutant Let-7a sensor that contains mismatches in its seed region and can no longer be silenced by Let-7a binding was used as a control. (**b**) Diagram showing the normalized activity of Let-7a luciferase sensors coexpressed with Let-7a and TRIM32-EGFP, DDX6-FLAG or TRIM32-EGFP and DDX6-FLAG in N2a cells (mean ± SEM; *N* = 10 independent experiments; Mann–Whitney U test, *P < 0.05, ***P < 0.005). (**c**) Diagram showing the normalized activity of Let-7a luciferase sensors coexpressed with Let-7a and either scrambled (scrbl.) shRNA or DDX6-shRNA in N2a cells (mean ± SEM; *N* = 4 independent experiments; Mann–Whitney U test, *P < 0.05 compared to scrambled shRNA). (**d**) Diagram showing the normalized activity of Let-7a luciferase sensors coexpressed with Let-7a and EGFP or mutant DDX6-EQ-EYFP in N2a cells (mean ± SEM; *N* = 9 independent experiments; *t*-test, **P < 0.01 compared to EGFP). (b–d) Note that a decrease in the luciferase signal reflects increased Let-7a activity and *vice versa*.

We have previously shown that one of the mechanisms by which TRIM32 induces neuronal differentiation is through the activation of the microRNA Let-7a (22). Given that the *C. elegans* homolog of DDX6 has also been implicated in the regulation of the Let-7 family of microRNAs (31), we sought to investigate whether mammalian DDX6 similarly influences Let-7a activity. Therefore, luciferase assays were performed using a Let-7a sensor encoding for firefly luciferase followed by Let-7a binding sites in the 3′ UTR (Figure 5a). A mutant Let-7a sensor that contains mismatches in its seed region and can no longer be silenced by Let-7a binding was used as a control (Figure 5a). Each construct was then transfected into neuroblastoma (N2a) cells together with DDX6, TRIM32 or both. Consistent with previously published data (22), the overexpression of TRIM32 led to a strong decrease in the luciferase signal, demonstrating the activation of Let-7a. DDX6 overexpression also significantly stimulated Let-7a activity and this effect was further enhanced upon coexpression of TRIM32 (Figure 5b).

We next asked whether DDX6 is necessary for Let-7a activity. Therefore, N2a cells were transfected with shRNA constructs containing either a scrambled sequence or a sequence targeting DDX6, which resulted in ∼50% knockdown (Supplementary Figure S7a,b). Following knockdown of DDX6, a significant increase in the luciferase signal could be detected (Figure 5c), demonstrating that Let-7a activity is decreased in the absence of DDX6.

To investigate whether the helicase domain of DDX6 is required for activating Let-7a, we used a mutant DDX6 construct (DDX6-EQ) that contains a point mutation within the DEAD box motif II of DDX6 and thus lacks helicase activity (44). Interestingly, the overexpression of this mutant in N2a cells led to a significant increase in the luciferase signal, representing reduced Let-7a activity (Figure 5d). This effect was not due to a disruption of the interaction with TRIM32, as DDX6-EQ was nevertheless able to coimmunoprecipitate TRIM32 (Supplementary Figure S7c). These data indicate that DDX6 function is necessary for the activation of Let-7a and that this effect depends on its helicase activity.

### DDX6 induces differentiation in NSCs

**Figure 6.**
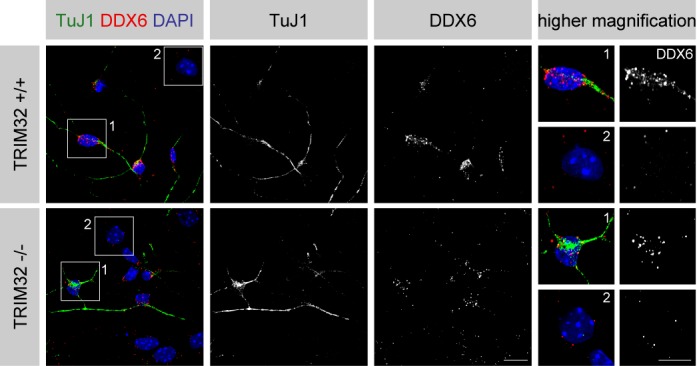
DDX6 is upregulated during neuronal differentiation in NSCs. Immunostaining of neurospheres derived from TRIM32 +/+ and TRIM32 −/− mice 5 days after the induction of neuronal differentiation with the indicated markers. The right panels show single cells at higher magnification. Scale bar = 20 μm; for high magnifications, 10 μm.

The fact that DDX6 is able to increase the activity of the neuronal differentiation-inducing miRNA Let-7a (22) suggests that DDX6 may play a role in the induction of neuronal differentiation. This hypothesis was further supported by the observation that DDX6 is upregulated upon neuronal differentiation of TRIM32 +/+ and −/− neurospheres, particularly in cells with a neuronal morphology compared to those with an astrocytic morphology. Indeed, costaining of DDX6 with the neuronal marker TuJ1 showed a substantially stronger signal for DDX6 in TuJ1-positive cells compared with TuJ1-negative cells (Figure 6).

Therefore, we sought to examine whether DDX6 has the ability to induce neuronal differentiation in NSCs and whether this is influenced by the presence or absence of TRIM32. To address these questions, neurospheres obtained from TRIM32 +/+ or −/− mice were nucleofected with DDX6 or EGFP expression constructs. One day after nucleofection, cells were differentiated into neurons for 5 days. To assess the degree of neuronal differentiation, cells were stained for the young neuronal marker TuJ1 as well as the proliferation marker Ki67.

**Figure 7.**
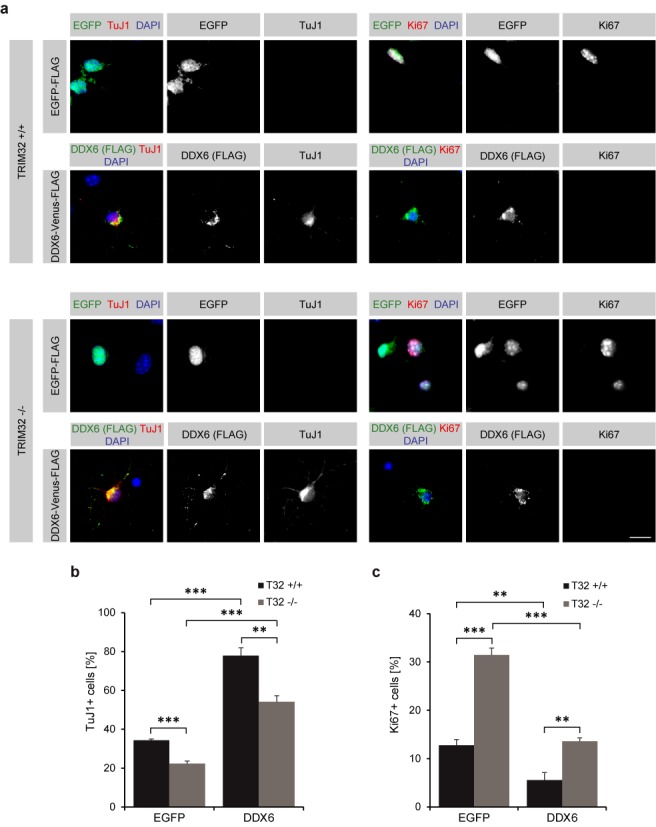
DDX6 is sufficient to induce neuronal differentiation in NSCs. (**a**) TRIM32 +/+ and −/− neurospheres were nucleofected with EGFP-FLAG or DDX6-Venus-FLAG and differentiated into neurons for 5 days. Immunostaining of the cells labeled as indicated is shown. Overexpressed DDX6 shows a dotted cytoplasmic localization. Scale bar = 15 μm. (**b** and **c**) Diagram showing the percentage of nucleofected TRIM32 +/+ and −/− neurons that are positive for the young neuronal marker TuJ1 (b) or the proliferation marker Ki67 (c) (mean ± SEM; *n* ≥ 160 cells, *N* = 4 independent TRIM32 +/+ and −/− neurosphere cultures; *t*-test, **P < 0.01, ***P < 0.005).

DDX6 overexpression in TRIM32 +/+ NSCs greatly increased the amount of TuJ1-positive cells (78% of cells) compared with the EGFP control transfection (34% of cells) (Figure 7a and b, Supplementary Figure S8a). Concomitant with this increase, the amount of proliferating, Ki67-positive cells was significantly decreased from 13 to <6% under these conditions (Figure 7a and c, Supplementary Figure S8a). When comparing the EGFP control-transfected TRIM32 +/+ to −/− NSCs, the amount of TuJ1-positive cells decreased to 22% and the amount of Ki67-positive cells increased to 31% in TRIM32 −/− NSCs (Figure 7, Supplementary Figure S8a), consistent with our previous results (Figure 4). Remarkably, overexpressing DDX6 in TRIM32-deficient NSCs could partially rescue this phenotype, resulting in an increase in the amount of TuJ1-positive cells (54%) and a decrease in the amount of Ki67-positive cells (14%), albeit to a lesser extent than in TRIM32 +/+ NSCs (Figure 7, Supplementary Figure S8a). Therefore, DDX6 inhibits proliferation and induces neuronal differentiation in NSCs in the presence and absence of TRIM32. However, because DDX6 cannot fully rescue the TRIM32 −/− phenotype, both proteins appear to be required for proper neuronal differentiation.

### Loss of DDX6 function inhibits the differentiation of NSCs

To further analyze whether DDX6 is required for neuronal differentiation, we introduced our scrambled- or DDX6-targeting shRNA constructs (Supplementary Figure S7a,b) into TRIM32 +/+ and −/− NSCs by nucleofection. The amount of TuJ1- and Ki67-positive cells was quantified 5 days after the induction of neuronal differentiation.

**Figure 8.**
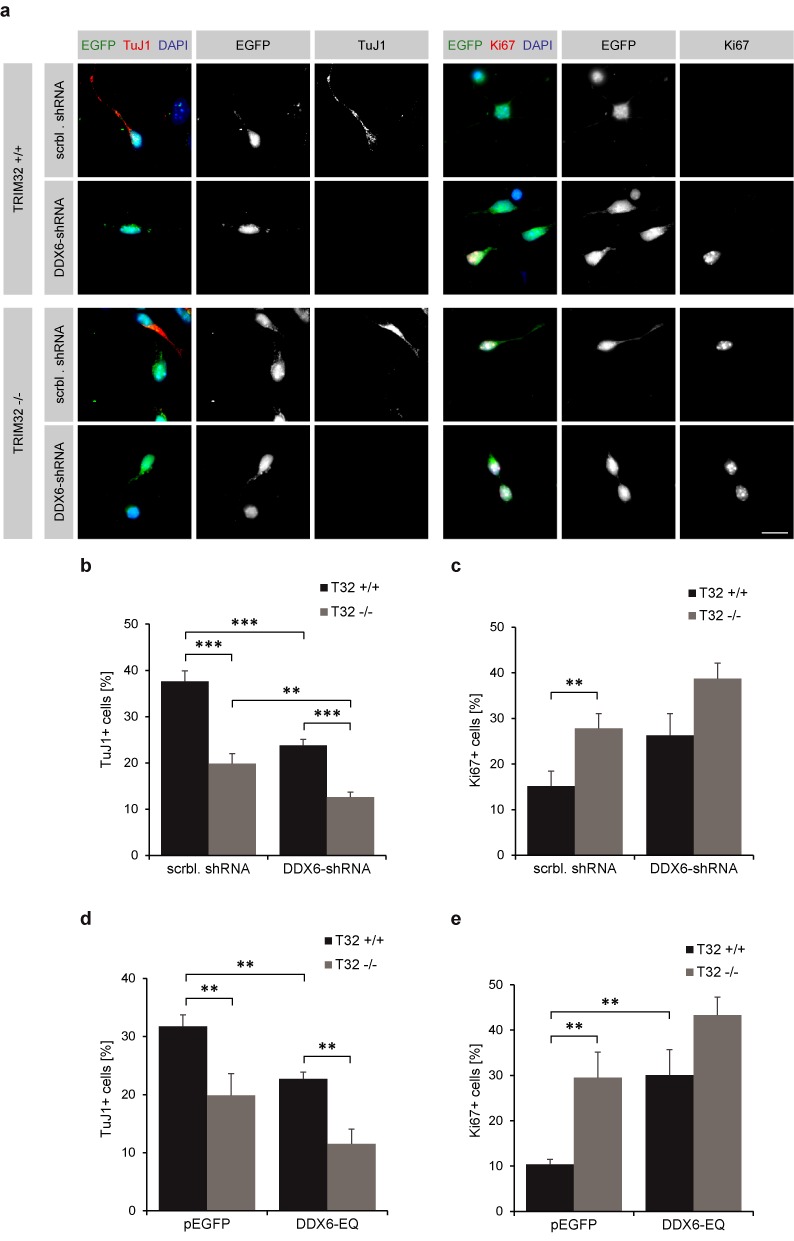
DDX6 function is necessary to induce neuronal differentiation in NSCs. (**a**) TRIM32 +/+ and −/− neurospheres were nucleofected with scrambled or DDX6–shRNA constructs (both expressing GFP) and were differentiated into neurons for 5 days. Immunostaining of the cells labeled as indicated is shown. Scale bar = 15 μm. (**b** and **c**) Diagram showing the percentage of nucleofected TRIM32 +/+ and −/− neurons that are positive for the young neuronal marker TuJ1 (b) or the proliferation marker Ki67 (c) upon knockdown of DDX6 (mean ± SEM; *n* ≥ 160 cells, *N* = 4 independent TRIM32 +/+ and −/− neurosphere cultures; *t*-test or Mann–Whitney U test, **P < 0.01, ***P < 0.005). (**d** and **e**) Diagram showing the percentage of nucleofected TRIM32 +/+ and −/− neurons that are positive for the young neuronal marker TuJ1 (d) or the proliferation marker Ki67 (e) upon DDX6-EQ overexpression. Representative images are shown in Supplementary Figure S6 (mean ± SEM; *n* ≥ 160, *N* = 4 independent TRIM32 +/+ and −/− neurosphere cultures; *t*-test or Mann–Whitney U test, **P < 0.01, ***P < 0.005).

Knockdown of DDX6 in TRIM32 +/+ NSCs reduced the amount of TuJ1-positive cells from 38% (control conditions) to 24% and slightly increased the amount of Ki67-positive cells (from 15 to 26%) (Figure 8a–c, Supplementary Figure S8b). This effect was even more pronounced, when DDX6 was knocked down in TRIM32 −/− NSCs, after which only 13% of electroporated cells still expressed TuJ1 and 39% of cells expressed Ki67 (Figure 8a–c, Supplementary Figure S8b). These results indicate that DDX6 and TRIM32 cooperate during the induction of neuronal differentiation in NSCs.

We next sought to investigate whether the neuronal-fate-inducing effect of DDX6 is dependent on its helicase activity. Therefore, TRIM32 +/+ and -/- NSCs were electroporated with the helicase-deficient DDX6-EQ mutant. Because the DDX6-EQ mutant had a similar effect on neuronal differentiation as the shRNA-mediated knockdown of DDX6 (Figure 8, Supplementary Figure S8b, Supplementary Figure S9), we conclude that the positive effect of DDX6 on neuronal differentiation is mediated by its helicase activity.

## DISCUSSION

In the present study, we provide evidence that the two RNA-regulating proteins TRIM32 and DDX6 cooperate in the regulation of microRNAs to promote neuronal differentiation. Using mass spectrometry and bioinformatics tools, we identified a novel set of potential TRIM32-associated proteins involved in neuronal differentiation. As the main purpose of this approach was to screen for novel potential TRIM32 interaction partners, it was conducted only once and interesting candidates were verified in additional assays. Nevertheless, the quality of our mass spectrometry approach is supported by the detection of several previously reported TRIM32 interaction partners, including α-actinin and tropomyosin-1α (45), myosin (46), HIV Tat (47) as well as UBE2N and UBE2V1 (29) (Supplementary Table S1). However, all conclusion drawn from newly identified proteins that were not verified in additional assays obviously have a lower degree of confidence and remain to be confirmed.

Many of the potentially TRIM32-associated proteins identified in the mass spectrometry analysis were associated with the GO term ‘positive regulation of neurogenesis’, which is consistent with the well-described role of TRIM32 in promoting neuronal differentiation in the embryonic and adult mouse brain (22,25,27). The importance of TRIM32 during neuronal differentiation is further highlighted by the impaired neuronal differentiation potential that we observed in TRIM32 −/− NSCs compared to TRIM32 +/+ NSCs.

Interestingly, we detected an enrichment of TRIM32-associated proteins involved in RNA-related processes, such as RNA stabilization or processing, consistent with the role of TRIM32 as a RISC cofactor in microRNA-mediated gene silencing during neuronal differentiation (22,48). In addition, the RISC protein Ago2 was identified as one of the TRIM32-associated proteins involved in RNA regulation. We further extended our analysis to the entire family of Ago and showed that TRIM32 can interact with Ago1, 2 and 3, but not with Ago4. However, only low levels of Ago4 expression could be obtained using our approach, suggesting that a potential interaction with TRIM32 may have gone undetected. Nevertheless, the abundance of the Ago4 protein is relatively low compared with the other Ago family members in many different cell types (49), indicating that it may play a minor role in microRNA-mediated gene silencing.

Given that Ago2 was identified as a candidate TRIM32 interaction partner in proliferating and differentiating NSCs and given that Ago2 has been well studied for its function in post-transcriptional gene silencing, as it is the only catalytic Ago protein in mammals (50), we focused on Ago2 in the subsequent experiments. We demonstrated that TRIM32 and Ago2 interact *in vivo* in both the embryonic and adult mouse brain, which contain large fractions of undifferentiated NSCs and postmitotic neurons, respectively. This result is consistent with the observed colocalization of TRIM32 and Ago2 in NSCs and differentiated neurons, further confirming that the interaction between these two proteins is important in both proliferating and differentiating cells. Because both proteins associate with each other in NSCs prior to the induction of neuronal differentiation, these NSCs may already be primed to undergo differentiation.

TRIM32 efficiently increased Let-7a activity, underscoring its function as a RISC cofactor in the induction of neuronal differentiation. Functioning as a RISC cofactor during the regulation of cell fate determination appears to be a common property of TRIM-NHL proteins. In *Drosophila*, the TRIM-NHL protein Mei-P26 has been shown to associate with RISC via Ago1, but in contrast to TRIM32, Mei-P26 was shown to inhibit microRNA activity to regulate proliferation within the germline (30). Brat, another TRIM-NHL protein in *Drosophila*, binds Ago1 and functions to post-transcriptionally inhibit Myc expression, thereby promoting the differentiation of neuroblasts (51). Remarkably, TRIM32 also regulates c-Myc protein levels, though in contrast to Brat, this is accomplished through the ubiquitination of c-Myc rather than through post-transcriptional gene silencing, thereby targeting c-Myc for proteasomal degradation (22,25). Interestingly, Myc has also been shown to be a direct target of Let-7 (52), suggesting that TRIM32 can additionally silence c-Myc expression indirectly at the post-transcriptional level. The fact that Myc also inhibits Let-7 transcription implies that both function in a double-negative feedback loop (52,53) that appears to be regulated by TRIM32. Another TRIM-NHL protein that functions in progenitor cell differentiation by regulating the microRNA Let-7 is NHL-2. In *C. elegans*, NHL-2 associates with the Ago ALG-1 and -2 and enhances the activity of the Let-7 family of microRNAs in concert with the RNA helicase CGH-1 (31).

Interestingly, one of the TRIM32-associated proteins identified by our mass spectrometry analysis in proliferating and differentiating NSCs was DDX6, the mammalian homolog of CGH-1 (33,34). TRIM32 and DDX6 interact in embryonic and adult mouse brains and colocalize in proliferating and differentiating NSCs, suggesting that both proteins function together during neuronal differentiation. We further showed that also Ago2 and DDX6 directly interact with each other both *in vitro* and *in vivo* and colocalize in NSCs and in neurons, consistent with a previous report that demonstrated the interaction between DDX6 and Ago1 as well as Ago2 in human cells (54). These findings suggest, that TRIM32, DDX6 and Ago2 form an miRNA activity-regulating complex.

DDX6 is a member of the evolutionarily conserved family of DEAD-box helicases that is characterized by the presence of an Asp-Glu-Ala-Asp (DEAD) motif and has been implicated in almost every aspect of RNA metabolism including transcription, splicing, translation, processing, degradation and storage (55,56). DEAD-box helicases usually function as part of larger multicomponent complexes and use ATP to unwind short RNA duplexes or to remodel RNA–protein complexes (34,56). Several studies indicated that DDX6 acts as a general translational repressor in the regulation of mRNA translation in yeast, human as well as mouse hippocampal neurons (54,57–59). DDX6 has thus been shown to associate with RISC and to function in microRNA-mediated gene silencing (54). In this context, recent publications reported a direct interaction between DDX6 and CNOT1, the scaffold subunit of the CCR4-NOT deadenylase complex (60–62). This interaction was found to stimulate the ATPase activity of DDX6 (61,62) and to be critical for miRNA-mediated gene silencing (60). Interestingly, CNOT1 as well as several poly(A) binding proteins have been identified in our mass spectrometry analysis (Supplementary Table S1).

Consistent with these findings, we demonstrated that the overexpression of DDX6 leads to a significant increase in Let-7a activity. This effect was further enhanced by TRIM32 coexpression, similar to the *C*. *elegans* homologs NHL-2 and CGH-1 (31). These data suggest that both TRIM32 and DDX6 are able to increase the activity of Let-7a. Furthermore, DDX6 is also required for Let-7a activity, as knockdown of DDX6 abolished Let-7a activity, consistent with a previous report showing that the depletion of DDX6 released microRNA-mediated gene silencing in human cells (54). Remarkably, a mutant DDX6 construct (DDX6-EQ), which lacks helicase activity due to a point mutation within the DEAD box motif II of DDX6 (44), also strongly decreased Let-7a activity. The fact that mutant DDX6 not only fails to activate Let-7a but also inhibits its activity suggests that this mutant functions in a dominant-negative manner, as has been described during hepatitis C viral replication (44). As was shown for the interaction of DDX6-EQ with viral proteins (44), the DDX6 EQ-mutation did not abolish the interaction with TRIM32, demonstrating that the helicase activity is necessary for activating Let-7a.

Consistent with its role in the repression of mRNA translation, DDX6 has been described as an essential component of P-bodies, which are cytoplasmic foci containing translationally repressed mRNAs. Interestingly, DDX6, TRIM32 and to a lesser extent Ago2 were strongly enriched in cytoplasmic punctae when overexpressed in NIH3T3 cells, as well as *in vivo* in NSCs and neurons. Notably, two different populations of these punctae were observed, those in which TRIM32 and Ago2, TRIM32 and DDX6 or Ago2 and DDX6 colocalized, and those in which the different proteins excluded one another. Some of these cytoplasmic punctae may represent P-bodies, as it has been shown that DDX6 and Ago2 colocalize in P-bodies in human cells (63), as do CGH-1 (*C. elegans* DDX6) and NHL-2 (*C. elegans* TRIM32) in neuronal and hypodermal cells in *C. elegans* (31,54). Because RISC colocalizes with P-bodies, they were proposed to be the sites where target mRNAs are repressed or degraded by RISC (63,64). However, the translational repression of mRNAs by microRNAs nevertheless occurs following the disruption of P-bodies, suggesting that efficient post-transcriptional gene silencing does not require P-bodies (54). Furthermore, it was shown that the microRNA Let-7 inhibits translation at the initiation step in the cytoplasm, indicating that repressed mRNAs localize to P-bodies for storage as a consequence rather than a cause of repression (65). Because TRIM32, DDX6 and Ago2 also showed diffuse cytoplasmic staining, they may form a complex in the cytoplasm to regulate microRNA-mediated gene silencing, after which the inhibited target mRNAs are subsequently stored in P-bodies, a process that may not require the combined action of all three proteins.

Interestingly, we observed the upregulation of DDX6 in differentiating neurons but not in astrocytes. Furthermore, because we were able to demonstrate that DDX6 significantly increases the activity of Let-7a, one of the key regulators of neuronal differentiation, we speculated that DDX6 may function to promote neuronal differentiation. Indeed, overexpression of DDX6 in NSCs strongly induced their differentiation into neurons. This effect was less pronounced in the absence of TRIM32, suggesting that both proteins are required for proper neuronal differentiation. However, because DDX6 could nevertheless induce differentiation in some TRIM32-deficient NSCs, it may possess additional functionality, independent of TRIM32. This is further supported by our observation that TRIM32 and DDX6 colocalized to some, but not all, cytoplasmic punctae. The loss of DDX6 function, either by shRNA or mutant DDX6-EQ, resulted in a strong decrease in the amount of TuJ1-positive neurons, along with an increase in the amount of proliferating cells, suggesting that functional DDX6 is required for neuronal differentiation. This effect was even more pronounced in the absence of TRIM32, indicating that both proteins cooperate during the induction of neuronal differentiation in NSCs. To promote neuronal differentiation, TRIM32 may recruit DDX6 to RISC and facilitate or stabilize its binding to the microRNA or target mRNA. Additionally, TRIM32 may stimulate the helicase activity of DDX6, leading to the unwinding of secondary structures within the target mRNA or the remodeling of RNA–protein complexes. Alternatively, DDX6 may bind to the mRNA first and recruit TRIM32 into the complex. However, because NSCs were still able to differentiate following the depletion of either DDX6 or TRIM32, both proteins can also act independently of each other in promoting neuronal differentiation. In future studies, it would be interesting to map the interaction surfaces of DDX6 and TRIM32 and to analyze the influence of abrogating their interaction on miRNA activity and neuronal differentiation.

In summary, our results provide significant insights into the regulation of microRNA-mediated neuronal differentiation. We identified a set of novel candidate interaction partners of TRIM32 during neuronal differentiation that are associated with the regulation of RNA, including Ago2 and DDX6. Of these, the RNA helicase DDX6 was able to efficiently induce neuronal differentiation in cooperation with TRIM32 by increasing the activity of the microRNA Let-7a.

## SUPPLEMENTARY DATA

Supplementary Data are available at NAR Online.

SUPPLEMENTARY DATA

## References

[1] Gage F.H. (2000). Mammalian neural stem cells. Science.

[2] Gotz M., Huttner W.B. (2005). The cell biology of neurogenesis. Nat. Rev. Mol. Cell Biol..

[3] Reynolds B.A., Weiss S. (1992). Generation of neurons and astrocytes from isolated cells of the adult mammalian central nervous system. Science.

[4] Goldman S.A., Nottebohm F. (1983). Neuronal production, migration, and differentiation in a vocal control nucleus of the adult female canary brain. Proc. Natl. Acad. Sci. U.S.A..

[5] Bunk E.C., Stelzer S., Hermann S., Schafers M., Schlatt S., Schwamborn J.C. (2011). Cellular organization of adult neurogenesis in the Common Marmoset. Aging Cell.

[6] Eriksson P.S., Perfilieva E., Bjork-Eriksson T., Alborn A.M., Nordborg C., Peterson D.A., Gage F.H. (1998). Neurogenesis in the adult human hippocampus. Nat. Med..

[7] Spalding K.L., Bergmann O., Alkass K., Bernard S., Salehpour M., Huttner H.B., Bostrom E., Westerlund I., Vial C., Buchholz B.A. (2013). Dynamics of hippocampal neurogenesis in adult humans. Cell.

[8] Ernst A., Alkass K., Bernard S., Salehpour M., Perl S., Tisdale J., Possnert G., Druid H., Frisen J. (2014). Neurogenesis in the striatum of the adult human brain. Cell.

[9] Bartel D.P. (2004). MicroRNAs: genomics, biogenesis, mechanism, and function. Cell.

[10] Winter J., Jung S., Keller S., Gregory R.I., Diederichs S. (2009). Many roads to maturity: microRNA biogenesis pathways and their regulation. Nat. Cell Biol..

[11] Eulalio A., Huntzinger E., Izaurralde E. (2008). Getting to the root of miRNA-mediated gene silencing. Cell.

[12] Hammell C.M. (2008). The microRNA-argonaute complex: a platform for mRNA modulation. RNA Biol..

[13] Bartel D.P. (2009). MicroRNAs: target recognition and regulatory functions. Cell.

[14] Palm T., Hemmer K., Winter J., Fricke I.B., Tarbashevich K., Sadeghi Shakib F., Rudolph I.M., Hillje A.L., De Luca P., Bahnassawy L. (2013). A systemic transcriptome analysis reveals the regulation of neural stem cell maintenance by an E2F1-miRNA feedback loop. Nucleic Acids Res..

[15] Shi Y., Zhao X., Hsieh J., Wichterle H., Impey S., Banerjee S., Neveu P., Kosik K.S. (2010). MicroRNA regulation of neural stem cells and neurogenesis. J. Neurosci..

[16] Coolen M., Thieffry D., Drivenes O., Becker T.S., Bally-Cuif L. (2012). miR-9 controls the timing of neurogenesis through the direct inhibition of antagonistic factors. Dev. Cell.

[17] Sun A.X., Crabtree G.R., Yoo A.S. (2013). MicroRNAs: regulators of neuronal fate. Curr. Opin. Cell Biol..

[18] Maffioletti E., Tardito D., Gennarelli M., Bocchio-Chiavetto L. (2014). Micro spies from the brain to the periphery: new clues from studies on microRNAs in neuropsychiatric disorders. Front. Cell. Neurosci..

[19] Palm T., Bahnassawy L., Schwamborn J. (2012). miRNAs and neural stem cells: a team to treat Parkinson's disease. RNA Biol..

[20] Roush S., Slack F.J. (2008). The let-7 family of microRNAs. Trends Cell Biol..

[21] Wulczyn F.G., Smirnova L., Rybak A., Brandt C., Kwidzinski E., Ninnemann O., Strehle M., Seiler A., Schumacher S., Nitsch R. (2007). Post-transcriptional regulation of the let-7 microRNA during neural cell specification. FASEB J..

[22] Schwamborn J.C., Berezikov E., Knoblich J.A. (2009). The TRIM-NHL protein TRIM32 activates microRNAs and prevents self-renewal in mouse neural progenitors. Cell.

[23] Reymond A., Meroni G., Fantozzi A., Merla G., Cairo S., Luzi L., Riganelli D., Zanaria E., Messali S., Cainarca S. (2001). The tripartite motif family identifies cell compartments. EMBO J..

[24] Napolitano L.M., Meroni G. (2012). TRIM family: pleiotropy and diversification through homomultimer and heteromultimer formation. IUBMB Life.

[25] Hillje A.L., Worlitzer M.M., Palm T., Schwamborn J.C. (2011). Neural stem cells maintain their stemness through protein kinase C zeta-mediated inhibition of TRIM32. Stem Cells.

[26] Gonzalez-Cano L., Hillje A.L., Fuertes-Alvarez S., Marques M.M., Blanch A., Ian R.W., Irwin M.S., Schwamborn J.C., Marin M.C. (2013). Regulatory feedback loop between TP73 and TRIM32. Cell Death Dis..

[27] Hillje A.L., Pavlou M.A., Beckmann E., Worlitzer M.M., Bahnassawy L., Lewejohann L., Palm T., Schwamborn J.C. (2013). TRIM32-dependent transcription in adult neural progenitor cells regulates neuronal differentiation. Cell Death Dis..

[28] Nicklas S., Otto A., Wu X., Miller P., Stelzer S., Wen Y., Kuang S., Wrogemann K., Patel K., Ding H. (2012). TRIM32 regulates skeletal muscle stem cell differentiation and is necessary for normal adult muscle regeneration. PLoS One.

[29] Napolitano L.M., Jaffray E.G., Hay R.T., Meroni G. (2011). Functional interactions between ubiquitin E2 enzymes and TRIM proteins. Biochem. J..

[30] Neumuller R.A., Betschinger J., Fischer A., Bushati N., Poernbacher I., Mechtler K., Cohen S.M., Knoblich J.A. (2008). Mei-P26 regulates microRNAs and cell growth in the Drosophila ovarian stem cell lineage. Nature.

[31] Hammell C.M., Lubin I., Boag P.R., Blackwell T.K., Ambros V. (2009). nhl-2 Modulates microRNA activity in Caenorhabditis elegans. Cell.

[32] Loedige I., Filipowicz W. (2009). TRIM-NHL proteins take on miRNA regulation. Cell.

[33] Navarro R.E., Shim E.Y., Kohara Y., Singson A., Blackwell T.K. (2001). cgh-1, a conserved predicted RNA helicase required for gametogenesis and protection from physiological germline apoptosis in C. elegans. Development.

[34] Weston A., Sommerville J. (2006). Xp54 and related (DDX6-like) RNA helicases: roles in messenger RNP assembly, translation regulation and RNA degradation. Nucleic Acids Res..

[35] Bolte S., Cordelieres F.P. (2006). A guided tour into subcellular colocalization analysis in light microscopy. J. microsc..

[36] Diederichs S., Jung S., Rothenberg S.M., Smolen G.A., Mlody B.G., Haber D.A. (2008). Coexpression of Argonaute-2 enhances RNA interference toward perfect match binding sites. Proc. Natl. Acad. Sci. U.S.A..

[37] Conti L., Pollard S.M., Gorba T., Reitano E., Toselli M., Biella G., Sun Y., Sanzone S., Ying Q.L., Cattaneo E. (2005). Niche-independent symmetrical self-renewal of a mammalian tissue stem cell. PLoS Biol..

[38] van den Berg D.L., Snoek T., Mullin N.P., Yates A., Bezstarosti K., Demmers J., Chambers I., Poot R.A. (2010). An Oct4-centered protein interaction network in embryonic stem cells. Cell Stem Cell.

[39] Wilm M., Shevchenko A., Houthaeve T., Breit S., Schweigerer L., Fotsis T., Mann M. (1996). Femtomole sequencing of proteins from polyacrylamide gels by nano-electrospray mass spectrometry. Nature.

[40] Nikolsky Y., Ekins S., Nikolskaya T., Bugrim A. (2005). A novel method for generation of signature networks as biomarkers from complex high throughput data. Toxicol. Lett..

[41] Shannon P., Markiel A., Ozier O., Baliga N.S., Wang J.T., Ramage D., Amin N., Schwikowski B., Ideker T. (2003). Cytoscape: a software environment for integrated models of biomolecular interaction networks. Genome Res..

[42] Kedde M., van Kouwenhove M., Zwart W., Oude Vrielink J.A., Elkon R., Agami R. (2010). A Pumilio-induced RNA structure switch in p27–3′ UTR controls miR-221 and miR-222 accessibility. Nat. Cell Biol..

[43] Sonoda J., Wharton R.P. (2001). Drosophila Brain Tumor is a translational repressor. Genes Dev..

[44] Jangra R.K., Yi M., Lemon S.M. (2010). DDX6 (Rck/p54) is required for efficient hepatitis C virus replication but not for internal ribosome entry site-directed translation. J. Virol..

[45] Cohen S., Zhai B., Gygi S.P., Goldberg A.L. (2012). Ubiquitylation by Trim32 causes coupled loss of desmin, Z-bands, and thin filaments in muscle atrophy. J. Cell Biol..

[46] Kudryashova E., Kudryashov D., Kramerova I., Spencer M.J. (2005). Trim32 is a ubiquitin ligase mutated in limb girdle muscular dystrophy type 2H that binds to skeletal muscle myosin and ubiquitinates actin. J. Mol. Biol..

[47] Fridell R.A., Harding L.S., Bogerd H.P., Cullen B.R. (1995). Identification of a novel human zinc finger protein that specifically interacts with the activation domain of lentiviral Tat proteins. Virology.

[48] Wulczyn F.G., Cuevas E., Franzoni E., Rybak A. (2011). miRNAs need a trim : regulation of miRNA activity by trim-NHL proteins. Adv. Exp. Med. Biol..

[49] Petri S., Dueck A., Lehmann G., Putz N., Rudel S., Kremmer E., Meister G. (2011). Increased siRNA duplex stability correlates with reduced off-target and elevated on-target effects. RNA.

[50] Meister G. (2013). Argonaute proteins: functional insights and emerging roles. Nat. Rev. Genet..

[51] Betschinger J., Mechtler K., Knoblich J.A. (2006). Asymmetric segregation of the tumor suppressor brat regulates self-renewal in Drosophila neural stem cells. Cell.

[52] Sampson V.B., Rong N.H., Han J., Yang Q., Aris V., Soteropoulos P., Petrelli N.J., Dunn S.P., Krueger L.J. (2007). MicroRNA let-7a down-regulates MYC and reverts MYC-induced growth in Burkitt lymphoma cells. Cancer Res..

[53] Bussing I., Slack F.J., Grosshans H. (2008). let-7 microRNAs in development, stem cells and cancer. Trends Mol. Med..

[54] Chu C.Y., Rana T.M. (2006). Translation repression in human cells by microRNA-induced gene silencing requires RCK/p54. PLoS Biol..

[55] Cordin O., Banroques J., Tanner N.K., Linder P. (2006). The DEAD-box protein family of RNA helicases. Gene.

[56] Linder P., Jankowsky E. (2011). From unwinding to clamping—the DEAD box RNA helicase family. Nat. Rev. Mol. Cell Biol..

[57] Coller J., Parker R. (2005). General translational repression by activators of mRNA decapping. Cell.

[58] Naarmann I.S., Harnisch C., Muller-Newen G., Urlaub H., Ostareck-Lederer A., Ostareck D.H. (2010). DDX6 recruits translational silenced human reticulocyte 15-lipoxygenase mRNA to RNP granules. RNA.

[59] Saito K., Kondo E., Matsushita M. (2011). MicroRNA 130 family regulates the hypoxia response signal through the P-body protein DDX6. Nucleic Acids Res..

[60] Rouya C., Siddiqui N., Morita M., Duchaine T.F., Fabian M.R., Sonenberg N. (2014). Human DDX6 effects miRNA-mediated gene silencing via direct binding to CNOT1. RNA.

[61] Chen Y., Boland A., Kuzuoglu-Ozturk D., Bawankar P., Loh B., Chang C.T., Weichenrieder O., Izaurralde E. (2014). A DDX6-CNOT1 complex and W-binding pockets in CNOT9 reveal direct links between miRNA target recognition and silencing. Mol. Cell.

[62] Mathys H., Basquin J., Ozgur S., Czarnocki-Cieciura M., Bonneau F., Aartse A., Dziembowski A., Nowotny M., Conti E., Filipowicz W. (2014). Structural and biochemical insights to the role of the CCR4-NOT complex and DDX6 ATPase in microRNA repression. Mol. Cell.

[63] Sen G.L., Blau H.M. (2005). Argonaute 2/RISC resides in sites of mammalian mRNA decay known as cytoplasmic bodies. Nat. Cell Biol..

[64] Liu J., Valencia-Sanchez M.A., Hannon G.J., Parker R. (2005). MicroRNA-dependent localization of targeted mRNAs to mammalian P-bodies. Nat. Cell Biol..

[65] Pillai R.S., Bhattacharyya S.N., Artus C.G., Zoller T., Cougot N., Basyuk E., Bertrand E., Filipowicz W. (2005). Inhibition of translational initiation by Let-7 MicroRNA in human cells. Science.

