# Supplementary material for: The RNA helicase DDX6 regulates cell-fate specification in neural stem cells via miRNAs
Source: Nucleic Acids Res. 2015 Feb 26;43(5):2638–54. doi: 10.1093/nar/gkv138 (PMC4357729; doi:10.1093/nar/gkv138)
Supplement: SUPPLEMENTARY DATA [file supp_43_5_2638__index.html]

The RNA helicase DDX6 regulates cell-fate specification in neural stem cells via miRNAs — SUPPLEMENTARY DATA 

# The RNA helicase DDX6 regulates cell-fate specification in neural stem cells via miRNAs

## SUPPLEMENTARY DATA

**Files in this Data Supplement:**

- Supplementary Data
- Supplementary Data
- Supplementary Data
- Supplementary Data
