# Supplementary material for: The RNA helicase DDX6 regulates cell-fate specification in neural stem cells via miRNAs
Source: Nucleic Acids Res. 2015 Feb 26;43(5):2638–54. doi: 10.1093/nar/gkv138 (PMC4357729; doi:10.1093/nar/gkv138)
Supplement: SUPPLEMENTARY DATA [file supp_gkv138_nar-03344-y-2014-File010.pdf]

The RNA helicase DDX6 regulates cell-fate specification in neural stem cells via miRNAs.

Sarah Nicklas[1,2], Satoshi Okawa[3], Anna-Lena Hillje[1,2], Laura González-Cano[1,2], Antonio del Sol[3], Jens C. Schwamborn[1,2*]

- Supplementary Information -

[1]Stem Cell Biology and Regeneration Group, Institute of Cell Biology, ZMBE, Westfälische Wilhelms-Universität Münster, Münster, Germany

[2]Developmental and Cellular Biology Group, Luxembourg Centre for Systems Biomedicine (LCSB), University of Luxembourg, Esch-Belval, Luxembourg

[3]Computational Biology Group, Luxembourg Centre for Systems Biomedicine (LCSB), University of Luxembourg, Esch-Belval, Luxembourg

* Correspondence: Jens C. Schwamborn, Luxembourg Centre for Systems Biomedicine (LCSB), University of Luxembourg, 7, avenue des Hauts-Fourneaux, L-4362 Esch-sur-Alzette Luxembourg; E-mail: jens.schwamborn@uni.lu

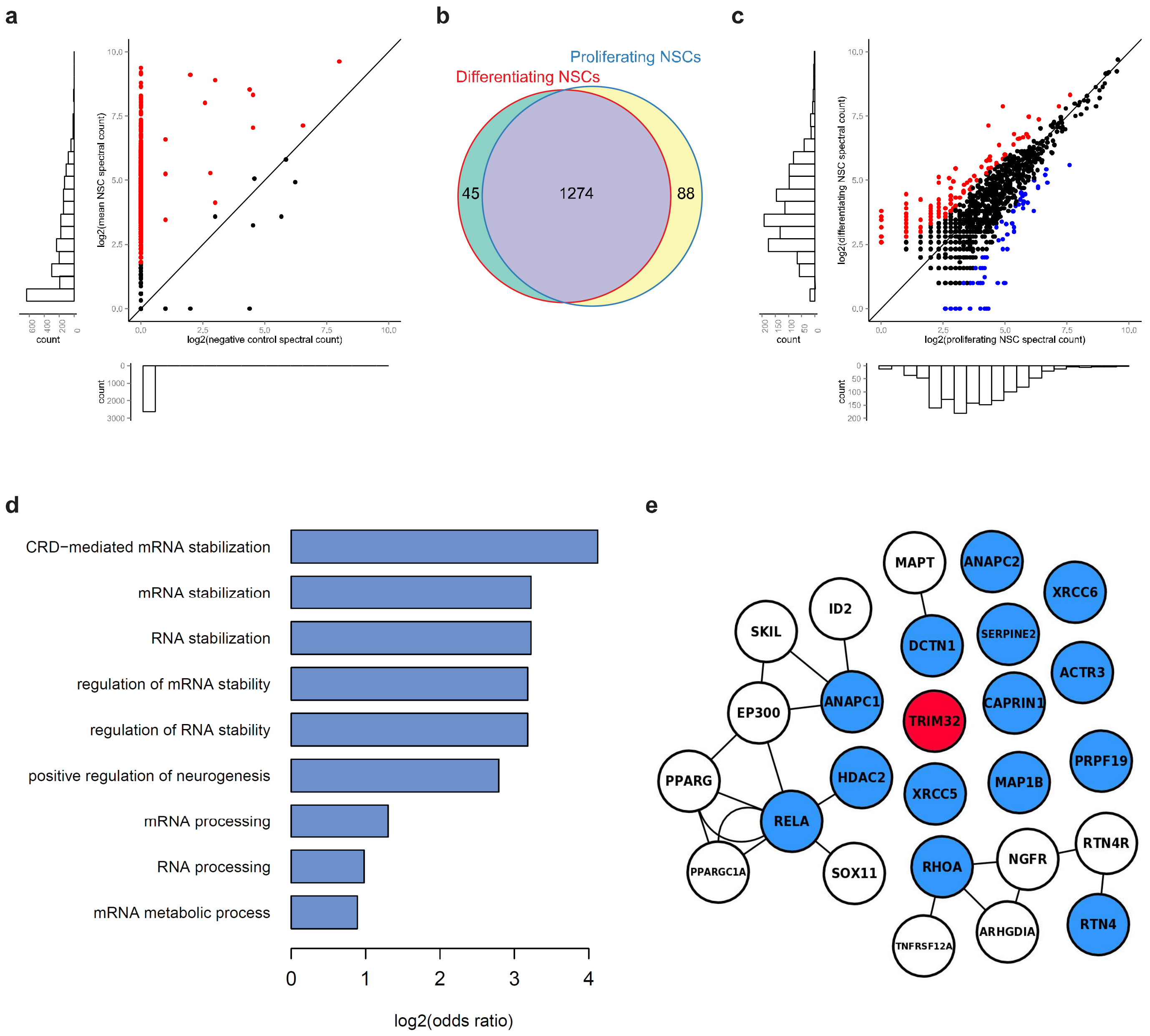

Supplementary Figure 1

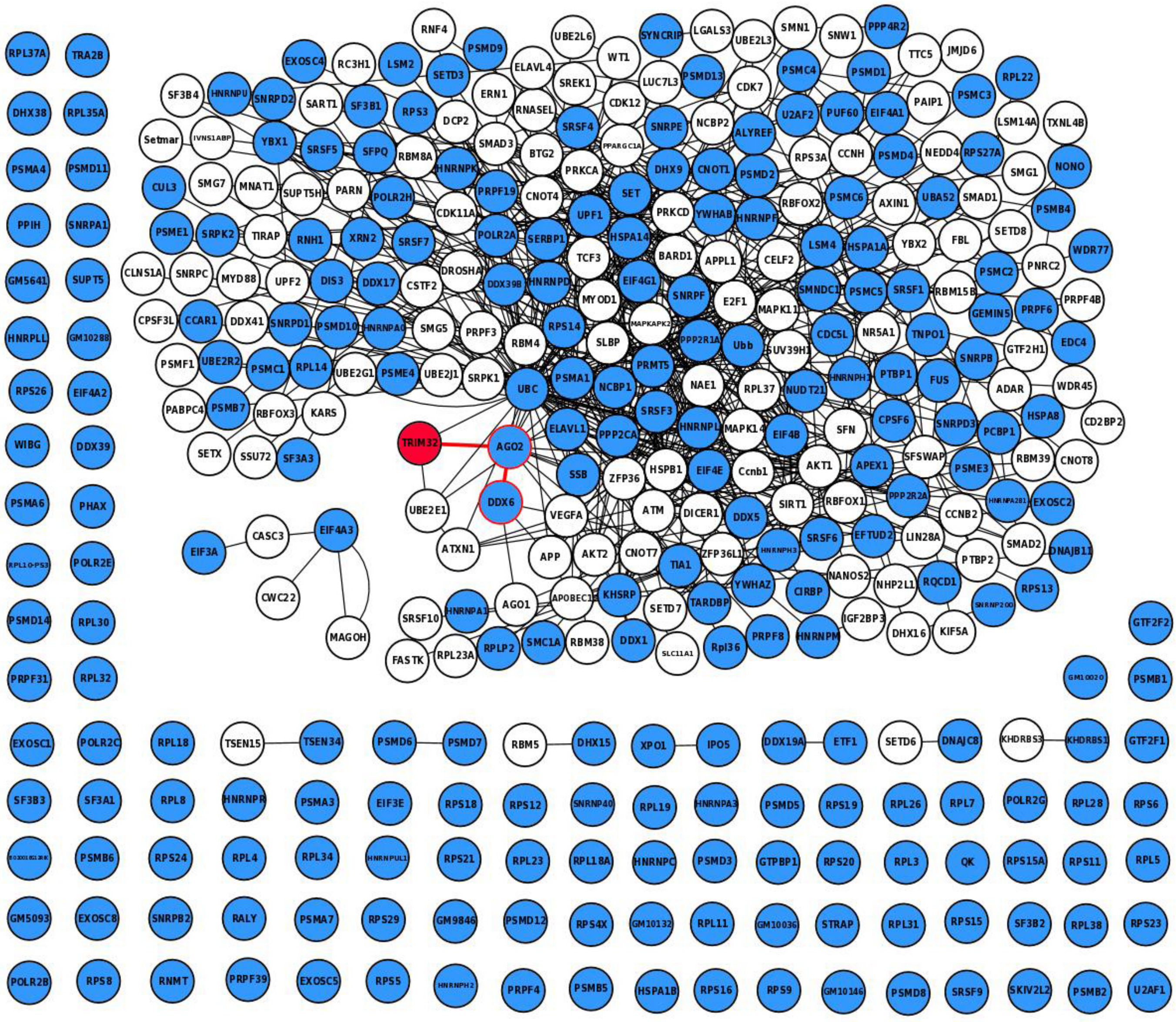

Supplementary Figure 2

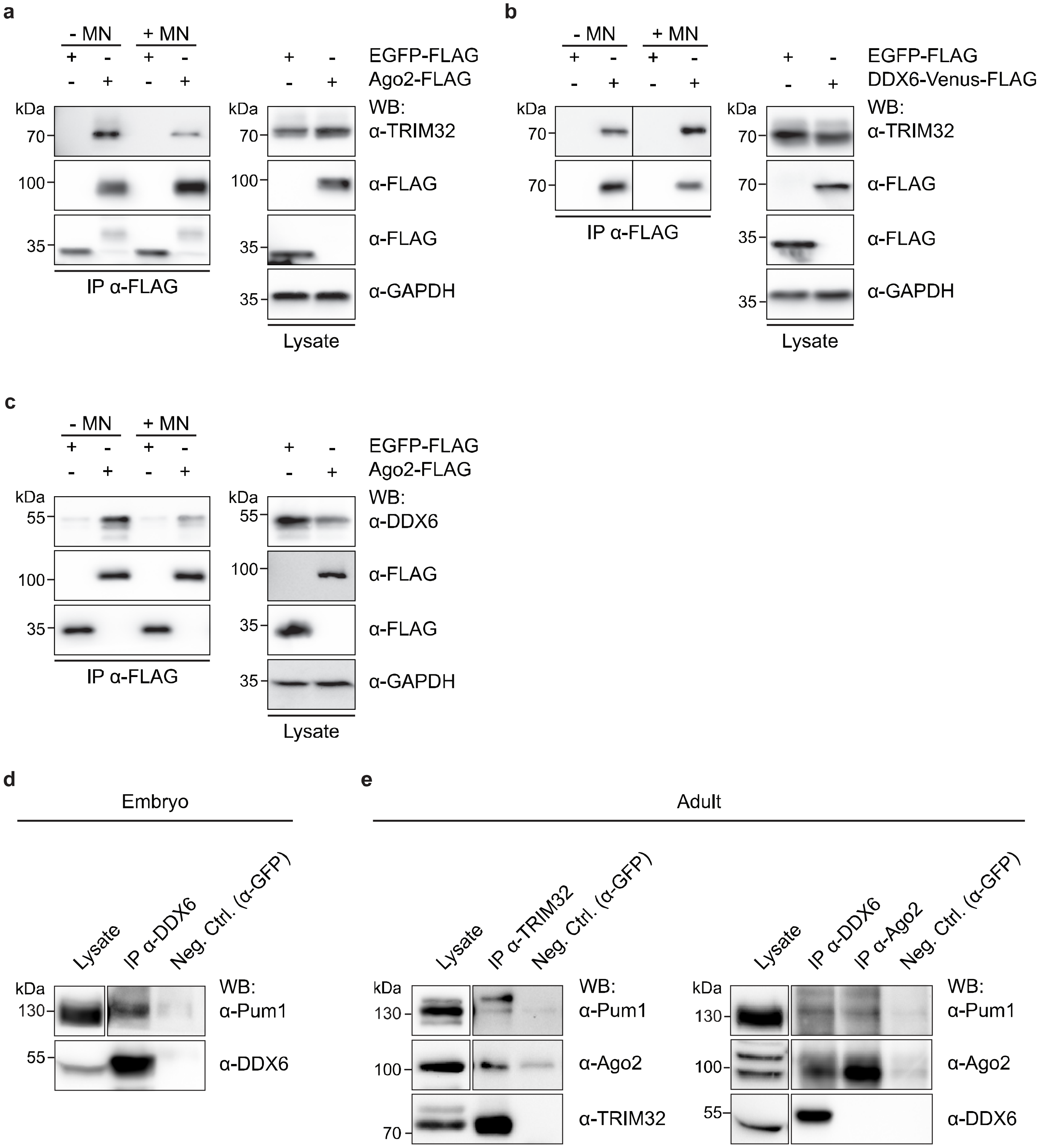

Supplementary Figure 3

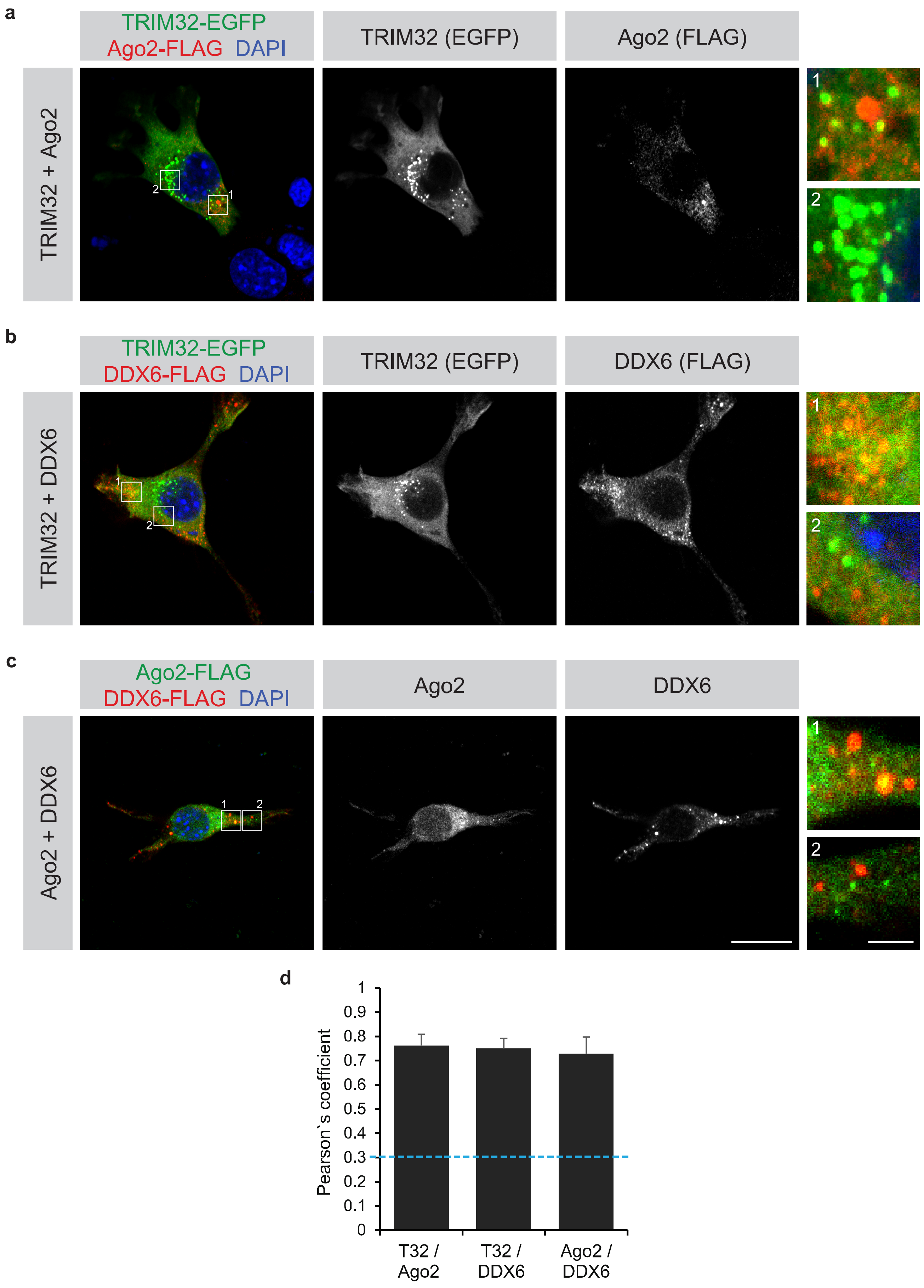

Supplementary Figure 4

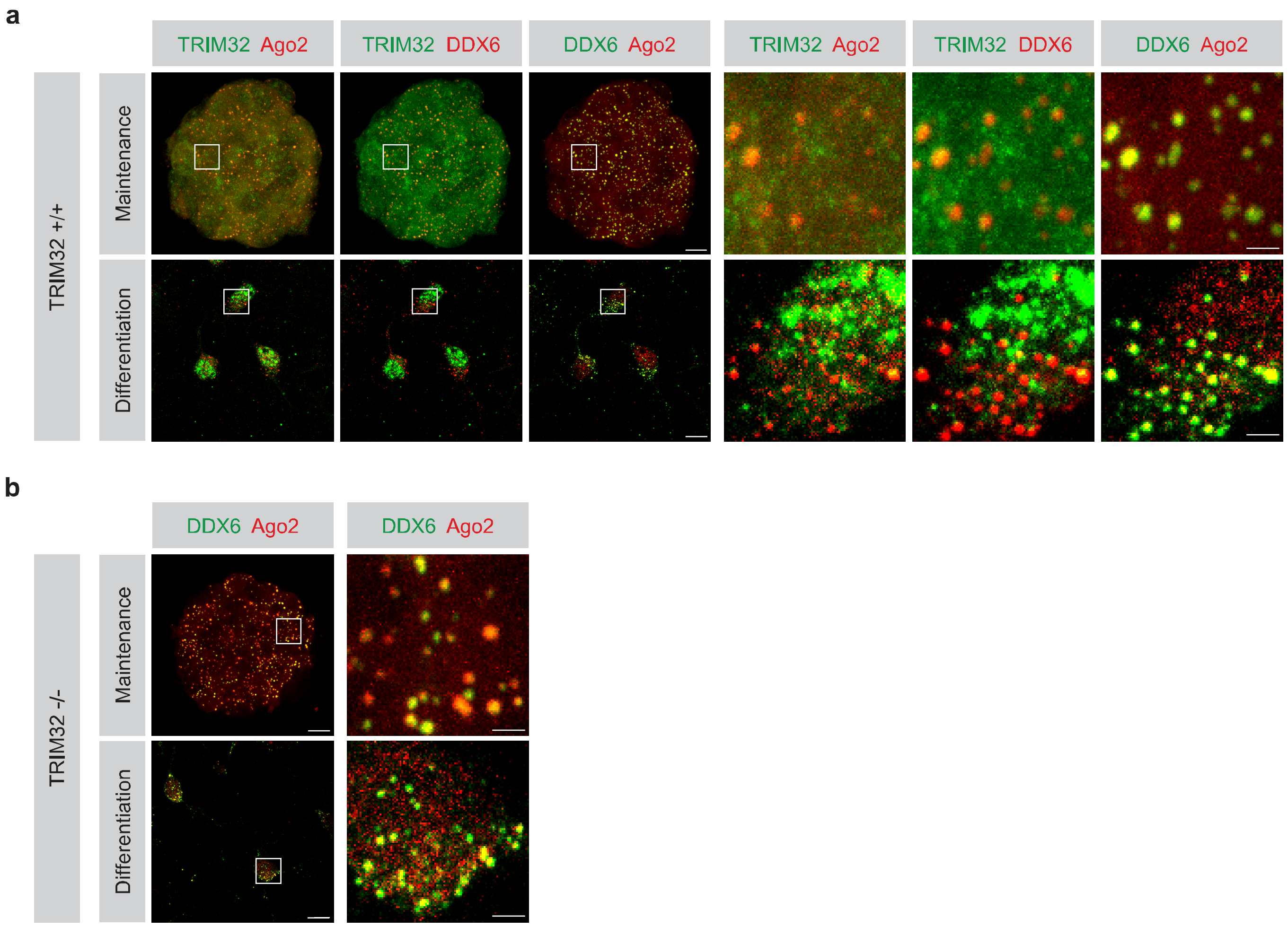

Supplementary Figure 5

c

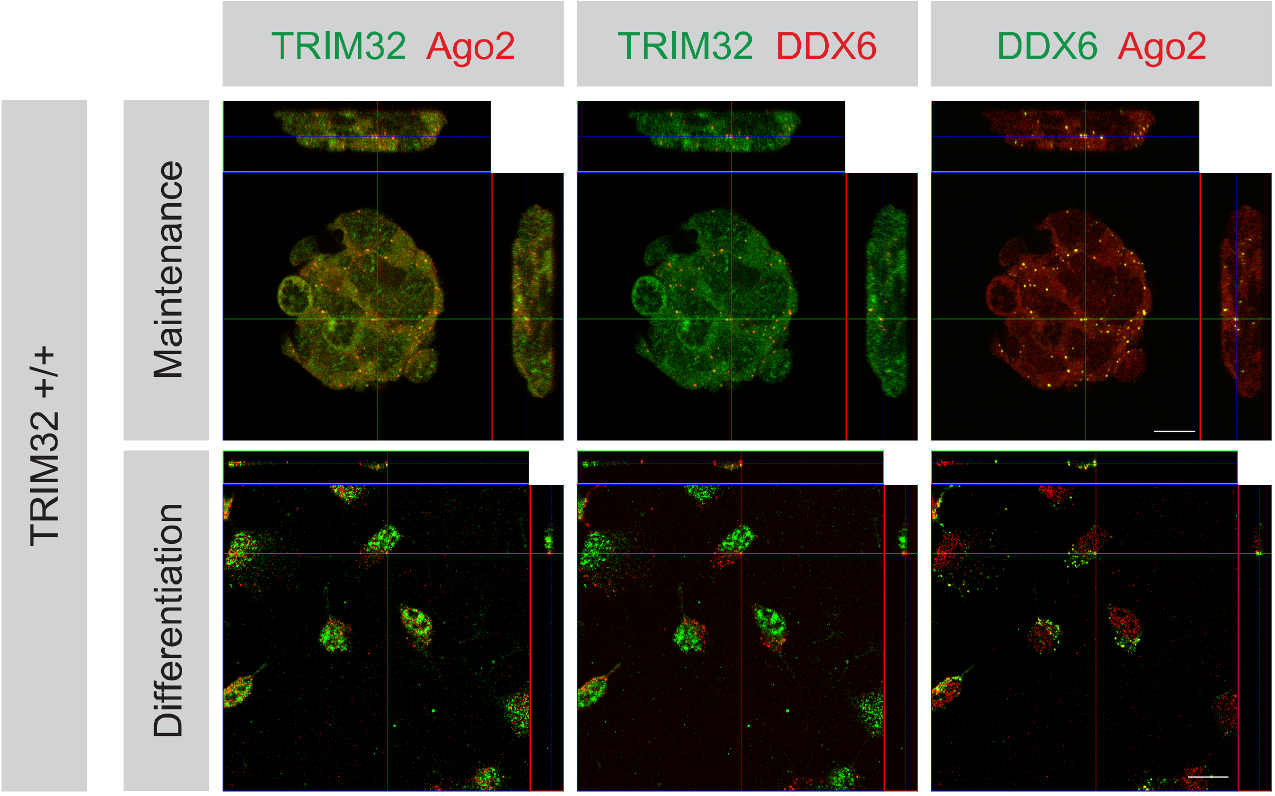
TRIM32 Ago2
TRIM32 DDX6
DDX6 Ago2
TRIM32 +/+
Maintenance
Differentiation

d

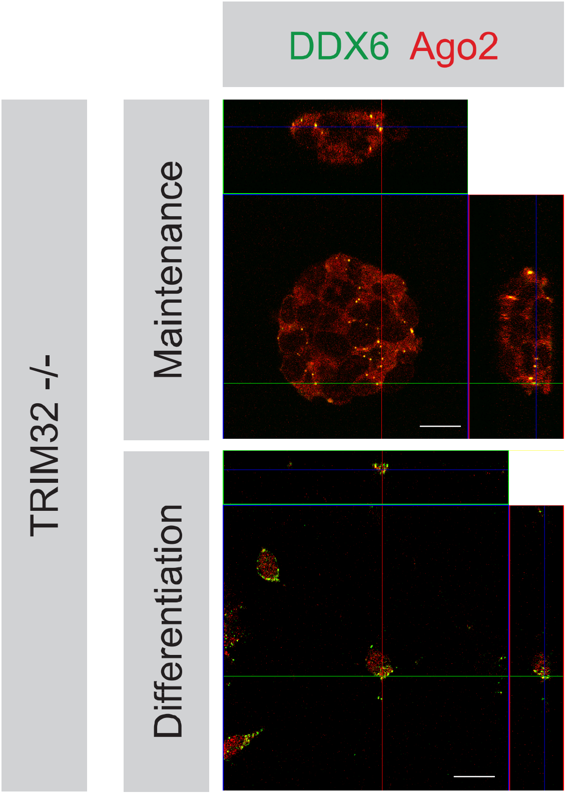
DDX6 Ago2
TRIM32 -/-
Maintenance
Differentiation

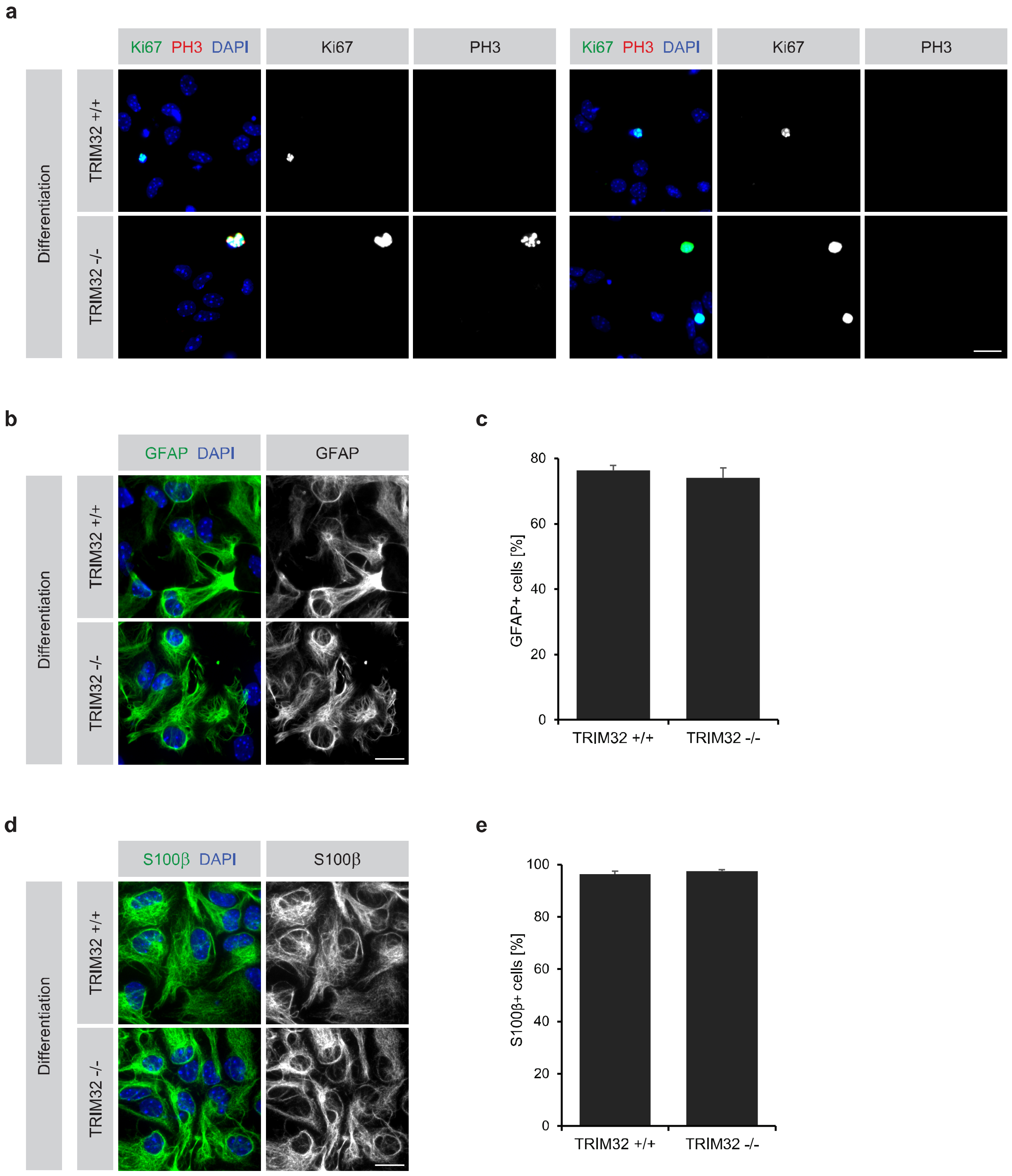

Supplementary Figure 6

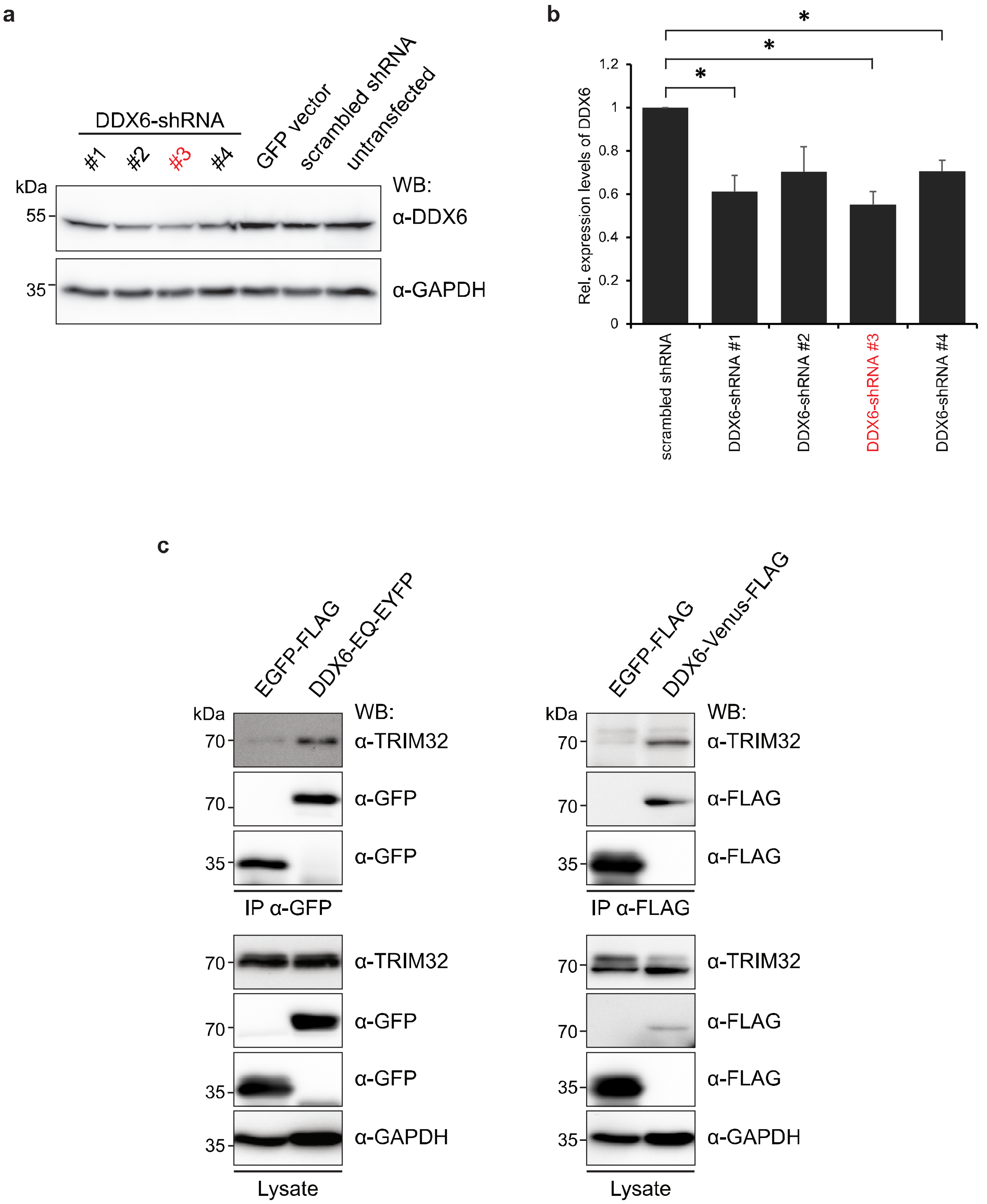

Supplementary Figure 7

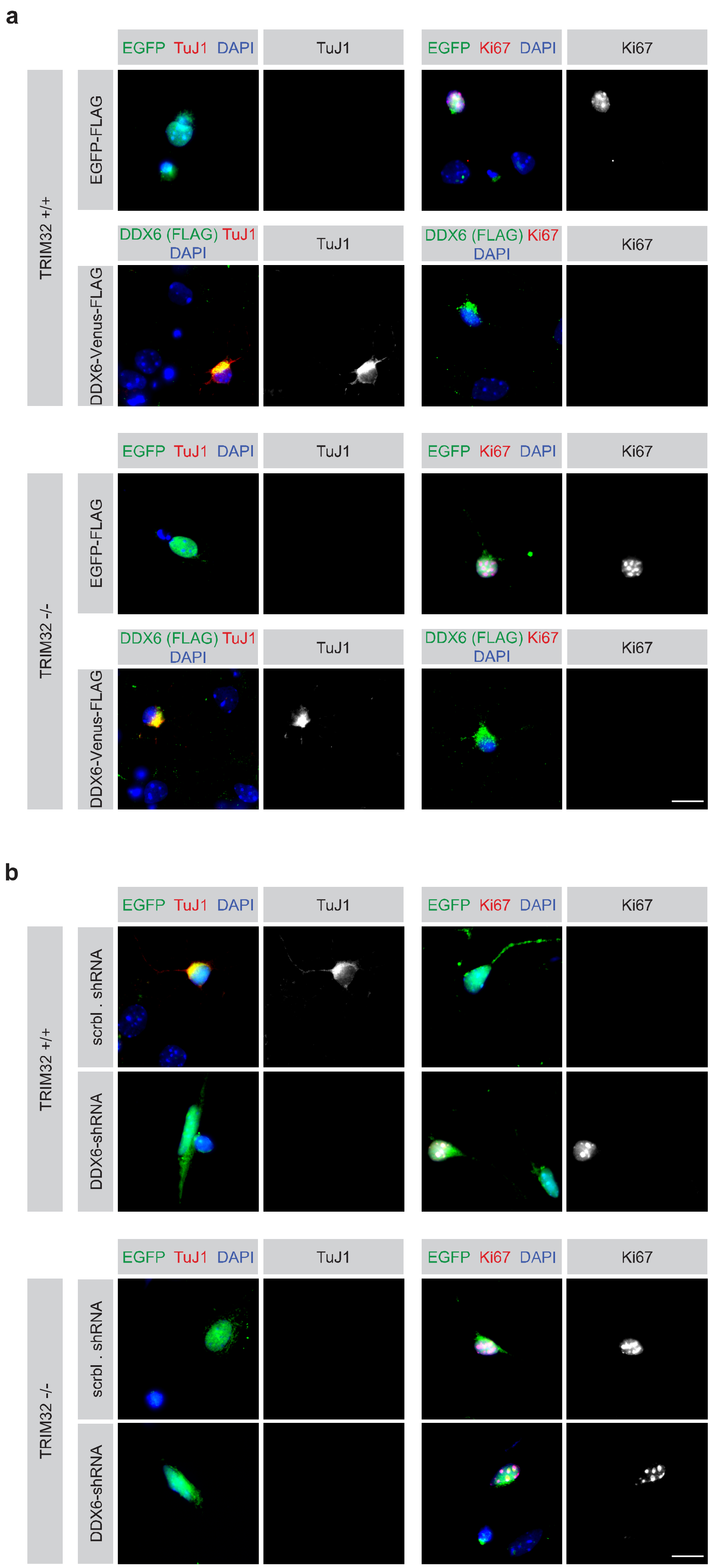

Supplementary Figure 8

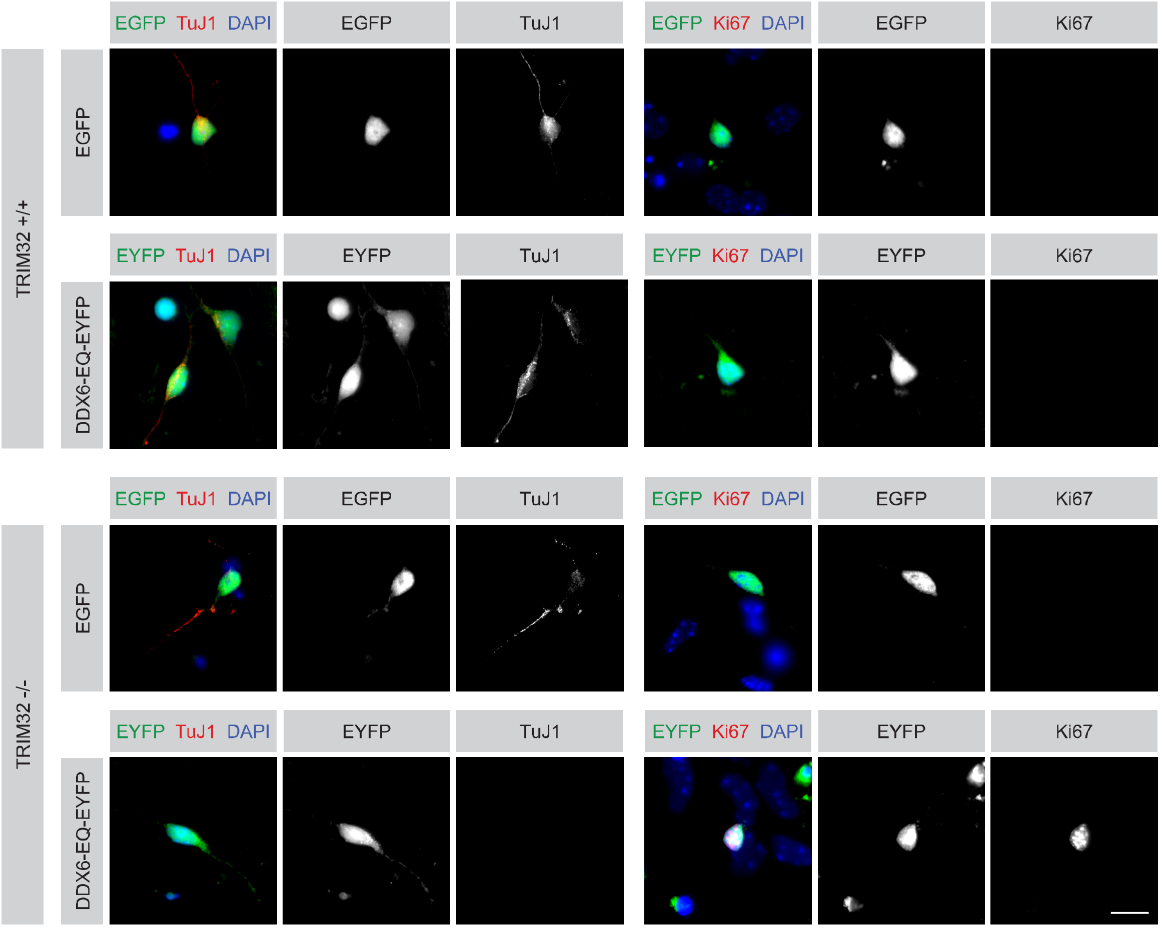

Supplementary Figure 9

Supplementary Figure legends:

Supplementary Figure 1. Identification of novel TRIM32-associated proteins via mass spectrometry.

(**a**) Plot and histogram of the mean NSC spectral count (differentiating NSCs and proliferating NSCs) and the negative control spectral count in log2 scale. Red dots indicate proteins identified in NSC samples that are significantly enriched ($p \leq 0.1$) above the negative control. (**b**) Venn diagram showing the degree of overlap between proteins identified in differentiating and proliferating NSCs. (**c**) Plot and histogram of the spectral count of 1274 identified proteins that overlap between differentiating and proliferating NSCs in log2 scale. Proteins that are significantly ($p \leq 0.1$ and log2 (absolute fold-change) ≥ 0.5) more abundant in the differentiating and proliferating NSCs are indicated by red and blue dots, respectively. (**d**) Enriched biological process GO terms of TRIM32-associated proteins that were significantly (adjusted for $p \leq 0.1$) more abundant during neuronal differentiation, with a focus on neurogenesis and RNA regulation (full GO term analysis is available in Supplementary Table 2). (**e**) Network analysis of TRIM32-associated proteins associated with the enriched GO term "positive regulation of neurogenesis" shown in (**d**). The red circle depicts the TRIM32 bait protein, blue circles represent TRIM32-associated proteins significantly enriched above the negative control, and white circles represent the 1st order neighbours of identified proteins. Protein-protein interaction data were retrieved from MetaCore.

Supplementary Figure 2. Identification of RNA-related TRIM32-associated proteins via mass spectrometry.

Network analysis of TRIM32-associated proteins associated with the RNA regulation-related GO processes shown in Supplementary Fig. 1d. The red circle depicts the TRIM32 bait protein, blue circles represent TRIM32-associated proteins significantly enriched above the

negative control, and white circles represent the 1st order neighbours of identified proteins. The interactions between TRIM32 and Ago2, as well as Ago2 and DDX6, are highlighted in red. Protein-protein interaction data were retrieved from MetaCore.

Supplementary Figure 3. The interactions between TRIM32, Ago2 and DDX6 are partially RNA dependent.

(**a**) HEK293T cells were transfected with the indicated constructs, and FLAG-tagged Ago2 was immunoprecipitated with an anti-FLAG antibody. IP samples were treated with micrococcal nuclease (MN). Ago2-associated TRIM32 was detected with an anti-TRIM32 antibody. TRIM32 and FLAG-tagged Ago2 were detected with specific antibodies as indicated for the IP and the lysate. (**b**) HEK293T cells were transfected with the indicated constructs, and Venus-FLAG-tagged DDX6 was immunoprecipitated with an anti-FLAG antibody. IP samples were treated with MN. DDX6-associated TRIM32 was detected with an anti-TRIM32 antibody. TRIM32 and Venus-FLAG-tagged DDX6 were detected with specific antibodies as indicated for the IP and the lysate. (**c**) HEK293T cells were transfected with the indicated constructs and FLAG-tagged Ago2 was immunoprecipitated with an anti-FLAG antibody. IP samples were treated with MN. Ago2-associated DDX6 was detected with an anti-DDX6 antibody. DDX6 and FLAG-tagged Ago2 were detected with specific antibodies as indicated for the IP and the lysate. In (**a-c**), GAPDH Western blots were used as loading control. (**d**) Immunoblots of IPs from embryonic brain lysates using antibodies against DDX6 or GFP (negative control). DDX6 and Pum1 were detected using the indicated specific antibodies in the IP and lysate. (**e**) Immunoblots of IPs from adult brain lysates using antibodies against TRIM32, DDX6, Ago2 or GFP (negative control). DDX6, TRIM32, Ago2 and Pum1 were detected using the indicated specific antibodies in the IP and lysate. Abbreviations: EGFP, enhanced green fluorescent protein; Ago, Argonaute; MN, micrococcal nuclease; IP, immunoprecipitation; WB, Western blot; Neg. Ctrl., negative control.

Supplementary Figure 4. TRIM32, Ago2 and DDX6 colocalise in NIH3T3.

(**a** and **b**) Immunostaining of NIH3T3 cells transfected with TRIM32-EGFP and Ago2-FLAG (**a**), TRIM32-EGFP and DDX6-FLAG (**b**) or Ago2-FLAG and DDX6-FLAG (**c**) with the indicated markers. Note that because both Ago2 and DDX6 are FLAG-tagged, antibodies recognising the endogenous proteins were used in (**c**). Single optical planes are shown in (**a-c**). The right panels show two distinct parts of the transfected cell at higher magnification to highlight either colocalisation (1) or exclusion (2) in enriched punctae. Scale bar = 15 µm; for high magnifications, 2 µm. (**d**) Diagram showing Pearson`s coefficients for the colocalisation of overexpressed TRIM32, Ago2 and DDX6. Colocalisation was defined as a Pearson`s coefficient ≥ 0.3 (blue dotted line) (mean ± SEM; n ≥ 19 cells, N = 3 independent transfection experiments).

Supplementary Figure 5. TRIM32, Ago2 and DDX6 colocalise in NSCs and neurons.

(**a** and **b**) Immunostaining of neurospheres derived from TRIM32 +/+ (**a**) and TRIM32 -/- (**b**) mice, cultured under maintenance conditions and 5 d after the induction of neuronal differentiation, with the indicated markers. Maximum intensity projections are shown. The right panels show a higher magnification of the boxed areas. Scale bars = 15 µm; for high magnifications, 2 µm. (**c** and **d**) Orthogonal images of neurospheres derived from TRIM32 +/+ (**c**) and TRIM32 -/- (**d**) mice, cultured under maintenance conditions and 5 d after the induction of neuronal differentiation, with the indicated markers. Scale bar = 20 µm.

Supplementary Figure 6. Absence of TRIM32 does not affect glial differentiation of NSCs.

(**a**) Immunostaining (additional to Fig. 4i) of neurospheres derived from TRIM32 +/+ and TRIM32 -/- mice 5 d after the induction of neuronal differentiation with the indicated markers.

Scale bars = 20 µm. (**b** and **d**) Immunostaining of neurospheres derived from TRIM32 +/+ and TRIM32 -/- mice 5 d after the induction of glial differentiation with the indicated markers. Scale bars = 20 µm. (**c** and **e**) Quantification of the percentage of TRIM32 +/+ and -/- cells positive for the astrocyte markers GFAP (**c**) and S100β (**e**) (mean ± SEM; $n \geq 1300$ cells, $N = 3$ independent TRIM32 +/+ and -/- neurosphere cultures).

Supplementary Figure 7. Knock-down of DDX6 by shRNA is efficient, and mutant DDX6-EQ directly binds TRIM32.

(**a**) Western blot analysis of N2a cells transfected for 72 h with four different shRNAs targeting DDX6, the same vector expressing GFP or the same vector expressing a scrambled sequence. DDX6 protein expression levels were detected using an anti-DDX6 antibody. A GAPDH Western blot was used as loading control. (**b**) Quantification of the relative expression levels of DDX6 normalised to GAPDH (mean ± SEM; $N = 4$ independent experiments; Mann-Whitney U test, $*p < 0.05$). (**c**) HEK293T cells were transfected with the indicated constructs, and EYFP-tagged DDX6-EQ was immunoprecipitated using an anti-GFP antibody, whereas Venus-FLAG-tagged DDX6 was immunoprecipitated using an anti-FLAG antibody. DDX6- and DDX6-EQ-associated TRIM32 was detected using an anti-TRIM32 antibody. TRIM32, EYFP-tagged DDX6-EQ and Venus-FLAG-tagged DDX6 were detected using specific antibodies as indicated for the IP and the lysate. A GAPDH Western Blot was used as loading control. Abbreviations: WB, Western bot; EGFP, enhanced green fluorescent protein; EYFP, enhanced yellow fluorescent protein; IP, immunoprecipitation.

Supplementary Figure 8. DDX6 is necessary and sufficient to induce neuronal differentiation in NSCs.

(**a**) TRIM32 +/+ and -/- neurospheres were nucleofected with EGFP-FLAG or DDX6-Venus-FLAG and differentiated into neurons for 5 d. Immunostaining (additional to Fig. 7) of the cells

labelled as indicated is shown. Scale bar = 15 µm. (**b**) TRIM32 +/+ and -/- neurospheres were nucleofected with scrambled or DDX6-shRNA constructs (both expressing GFP) and were differentiated into neurons for 5 d. Immunostaining (additional to Fig. 8) of the cells labelled as indicated is shown. Scale bar = 15 µm.

Supplementary Figure 9. Induction of neuronal differentiation by DDX6 depends on its helicase activity.

TRIM32 +/+ and -/- neurospheres were nucleofected with EGFP or DDX6-EQ-EYFP and differentiated into neurons for 5 d. Immunostaining of the cells labelled as indicated is shown. Scale bar = 15 µm. The corresponding quantifications are shown in Fig. 8.
