# Supplementary material for: The RNA helicase DDX6 regulates cell-fate specification in neural stem cells via miRNAs
Source: Nucleic Acids Res. 2015 Feb 26;43(5):2638–54. doi: 10.1093/nar/gkv138 (PMC4357729; doi:10.1093/nar/gkv138)
Supplement: SUPPLEMENTARY DATA [file supp_gkv138_nar-03344-y-2014-File011.pdf]

Trim32 enriched Proteins

Trim32 pull-down enriched proteins in the two NSC samples in comparison to the negative

Uniprot accession	Gene symbol	Protein description	Spectral count control
Q9D937	1810009A15RIK	RIKEN cDNA 1810009A15 gene	NA
Q9CPN9	2210010C04RIK	RIKEN cDNA 2210010C04 gene	NA
D3YZA1;D3Z7T7;E9	2500003M10RIK	Uncharacterized protein	NA
Q9CQE8	2700060E02RIK	RIKEN cDNA 2700060E02 gene	NA
E9Q4P0;E9QNP0	2810422J05RIK	Uncharacterized protein	NA
Q6ZWQ9	2900073G15RIK	MCG5400	NA
Q3THG9	AARSD1	alanyl-tRNA synthetase domain cc	NA
P55096	ABCD3	ATP-binding cassette, sub-family	NA
P61222	ABCE1	ATP-binding cassette, sub-family	NA
Q6P542	ABCF1	ATP-binding cassette, sub-family	NA
Q99LE6	ABCF2	ATP-binding cassette, sub-family	NA
Q8BWT1	ACAA2	acetyl-Coenzyme A acyltransferas	NA
Q5SWU9	ACACA	acetyl-Coenzyme A carboxylase a	NA
Q9D7B6	ACAD8	acyl-Coenzyme A dehydrogenase	NA
P51174	ACADL	acyl-Coenzyme A dehydrogenase	NA
Q07417	ACADS	acyl-Coenzyme A dehydrogenase	NA
Q9DBL1	ACADSB	acyl-Coenzyme A dehydrogenase	NA
P50544	ACADVL	acyl-Coenzyme A dehydrogenase	NA
Q8QZT1	ACAT1	acetyl-Coenzyme A acetyltransfera	NA
Q8BMP6	ACBD3	acyl-Coenzyme A binding domain	NA
Q91V92	ACLY	ATP citrate lyase	NA
Q99KI0	ACO2	aconitase 2, mitochondrial	NA
Q9R0H0	ACOX1	acyl-Coenzyme A oxidase 1, palm	NA
Q99PU5	ACSBG1	acyl-CoA synthetase bubblegum fa	NA
P68134	ACTA1	actin, alpha 1, skeletal muscle	NA
P62737	ACTA2	actin, alpha 2, smooth muscle, ao	NA
P60710	ACTB	actin, beta	23
Q8BFZ3	ACTBL2	actin, beta-like 2	NA
P68033	ACTC1	actin, alpha, cardiac muscle 1	NA
P63260	ACTG1	actin, gamma, cytoplasmic 1	23
Q7TPR4	ACTN1	actinin, alpha 1	NA
P57780	ACTN4	actinin alpha 4	NA
P61164	ACTR1A	ARP1 actin-related protein 1A, cei	NA
Q8R5C5	ACTR1B	ARP1 actin-related protein 1B, cei	NA
P61161	ACTR2	ARP2 actin-related protein 2	NA
Q99JY9	ACTR3	ARP3 actin-related protein 3	NA
Q9JKV1	ADRM1	adhesion regulating molecule 1	NA
Q8CJG0	AGO2	argonaute RISC catalytic subunit 2	NA
P50247	AHCY	S-adenosylhomocysteine hydrolas	NA
F7BRM2	AHNAK	AHNAK nucleoprotein (desmoyoki	NA
Q8BK64	AHSA1	AHA1, activator of heat shock pro	NA
Q6PDI5	AI314180	expressed sequence AI314180	NA
P31230	AIMP1	aminoacyl tRNA synthetase comp	NA
Q8R010	AIMP2	aminoacyl tRNA synthetase comp	NA
O08915	AIP	aryl-hydrocarbon receptor-interact	NA
Q9WTP7	AK3	adenylate kinase 3	NA
Q9WUR9	AK4	adenylate kinase 4	NA
Q9DBR0	AKAP8	A kinase (PRKA) anchor protein 8	NA
P07724	ALB	albumin	NA

Q9Z110	ALDH18A1	aldehyde dehydrogenase 18 famil	NA
P05064	ALDOA	aldolase A, fructose-bisphosphate	NA
P05063	ALDOC	aldolase C, fructose-bisphosphate	NA
O08583	ALYREF	Aly/REF export factor	NA
P53995	ANAPC1	anaphase promoting complex sub	NA
Q8BZQ7	ANAPC2	anaphase promoting complex sub	NA
Q91W96	ANAPC4	anaphase promoting complex sub	NA
Q8BTZ4	ANAPC5	anaphase-promoting complex sub	NA
E9PW46;Q8C8R3;F(	ANK2	ankyrin 2, brain	NA
Q810B6	ANKFY1	ankyrin repeat and FYVE domain	NA
Q99NH0	ANKRD17	ankyrin repeat domain 17	NA
Q505D1	ANKRD28	ankyrin repeat domain 28	NA
O35381	ANP32A	acidic (leucine-rich) nuclear phosp	NA
Q9EST5	ANP32B	acidic (leucine-rich) nuclear phosp	NA
P97822	ANP32E	acidic (leucine-rich) nuclear phosp	NA
P07356	ANXA2	annexin A2	NA
Q5SVG5;Q8CC13;O	AP1B1	adaptor protein complex AP-1, be	NA
P17426	AP2A1	adaptor-related protein complex 2	NA
P17427	AP2A2	adaptor-related protein complex 2	NA
Q9DBG3	AP2B1	adaptor-related protein complex 2	NA
Q3TWV4;P84091	AP2M1	adaptor-related protein complex 2	NA
Q8R146	APEH	acylpeptide hydrolase	NA
P28352	APEX1	apurinic/apyrimidinic endonucleas	NA
O35841	API5	apoptosis inhibitor 5	NA
Q5XJY5	ARCN1	archain 1	NA
P84078	ARF1	ADP-ribosylation factor 1	NA
P61205	ARF3	ADP-ribosylation factor 3	NA
P61750	ARF4	ADP-ribosylation factor 4	NA
P84084	ARF5	ADP-ribosylation factor 5	NA
Q5FWK3	ARHGAP1	Rho GTPase activating protein 1	NA
Q8K0Q5	ARHGAP18	Rho GTPase activating protein 18	NA
Q60875	ARHGEF2	rho/rac guanine nucleotide exchar	NA
Q8VEH3	ARL8A	ADP-ribosylation factor-like 8A	NA
Q9CVB6	ARPC2	actin related protein 2/3 complex,	NA
Q9JM76	ARPC3	actin related protein 2/3 complex,	NA
P59999	ARPC4	actin related protein 2/3 complex,	NA
Q9D365	ARXES1	adipocyte-related X-chromosome	NA
Q9CQE6	ASF1A	ASF1 anti-silencing function 1 hon	NA
O54984	ASNA1	arsA arsenite transporter, ATP-bir	NA
Q8BSY0	ASPH	aspartate-beta-hydroxylase	NA
Q8VBT9	ASPSCR1	alveolar soft part sarcoma chromc	NA
F8WI94;O55143	ATP2A2	ATPase, Ca2 transporting, cardiac	NA
Q03265	ATP5A1	ATP synthase, H transporting, mit	NA
P56480	ATP5B	ATP synthase, H transporting mitc	NA
A2AKU9;Q8C2Q8;Q!	ATP5C1	ATP synthase, H transporting, mit	NA
Q9CQQ7	ATP5F1	ATP synthase, H transporting, mit	NA
Q9DCX2	ATP5H	ATP synthase, H transporting, mit	NA
Q9DB20	ATP5O	ATP synthase, H transporting, mit	NA
P50516	ATP6V1A	ATPase, H transporting, lysosoma	NA
P62814	ATP6V1B2	ATPase, H transporting, lysosoma	NA
P28658	ATXN10	ataxin 10	NA
E9QQ60;O70305;E9	ATXN2	ataxin 2	NA
Q7TQH0	ATXN2L	ataxin 2-like	NA
Q9CWZ3	B020018G12RIK	RIKEN cDNA B020018G12 gene	NA
Q3UI43	BABAM1	BRISC and BRCA1 A complex me	NA

Q3UF95;Q9Z1R2;G3	BAG6	BCL2-associated athanogene 6	NA
Q07813	BAX	BCL2-associated X protein	NA
Q61361	BCAN	brevican	NA
Q61335	BCAP31	B cell receptor associated protein	NA
P50136;Q3U3J1	BCKDHA	branched chain ketoacid dehydrog	NA
P59017	BCL2L13	BCL2-like 13 (apoptosis facilitator)	NA
O88738	BIRC6	baculoviral IAP repeat-containing	NA
P46737	BRCC3	BRCA1/BRCA2-containing comple	NA
Q8K3W0	BRE	brain and reproductive organ-expr	NA
Q64152	BTF3	basic transcription factor 3	NA
Q9WVA3	BUB3	budding uninhibited by benzimidaz	NA
O54825	BYSL	bystin-like	NA
Q9CQC6	BZW1	basic leucine zipper and W2 doma	NA
Q91VK1	BZW2	basic leucine zipper and W2 doma	NA
A2AU62;A2AU61	C130057N11RIK	HnRNP-associated with lethal yell	NA
Q8R5L1;O35658	C1QBP	complement component 1, q subc	NA
Q9CXW3	CACYBP	calcyclin binding protein	NA
E9QAI5;B2RQC6;B7	CAD	carbamoyl-phosphate synthetase	NA
E9QA15;Q8VCQ8;E	CALD1	caldesmon 1	NA
P62204	CALM1	calmodulin 1	NA
P14211	CALR	calreticulin	NA
G3UXA8;G3UWR0;(	CALU	calumenin	NA
P28652	CAMK2B	calcium/calmodulin-dependent pro	NA
Q6ZQ38	CAND1	cullin associated and neddylation	NA
Q6ZQ73	CAND2	cullin-associated and neddylation-	NA
P35564	CANX	calnexin	NA
P40124	CAP1	CAP, adenylate cyclase-associate	NA
O08529	CAPN2	calpain 2	NA
O88456	CAPNS1	calpain, small subunit 1	NA
Q60865	CAPRIN1	cell cycle associated protein 1	NA
E9PWZ5;Q5RKN9	CAPZA1	capping protein (actin filament) mu	NA
P47754	CAPZA2	capping protein (actin filament) mu	NA
P47757	CAPZB	capping protein (actin filament) mu	NA
Q9ER72	CARS	cysteinyl-tRNA synthetase	NA
Q91VT4	CBR4	carbonyl reductase 4	NA
Q9DCC5;P23198;D3	CBX3	chromobox 3	NA
Q8CH18	CCAR1	cell division cycle and apoptosis re	NA
Q8VDP4	CCAR2	cell cycle activator and apoptosis r	NA
Q9D8X2	CCDC124	coiled-coil domain containing 124	NA
Q8CDN8	CCDC38	coiled-coil domain containing 38	NA
D3YZP9	CCDC6	coiled-coil domain containing 6	NA
Q5SNZ0	CCDC88A	coiled coil domain containing 88A	NA
P25322	CCND1	cyclin D1	NA
P80314	CCT2	chaperonin containing Tcp1, subu	NA
P80318	CCT3	chaperonin containing Tcp1, subu	NA
P80315	CCT4	chaperonin containing Tcp1, subu	NA
P80316	CCT5	chaperonin containing Tcp1, subu	NA
P80317;E9QPA6	CCT6A	chaperonin containing Tcp1, subu	NA
P80313	CCT7	chaperonin containing Tcp1, subu	NA
P42932	CCT8	chaperonin containing Tcp1, subu	NA
Q8R422	CD109	CD109 antigen	NA
Q8R349	CDC16	CDC16 cell division cycle 16	NA
Q8BGZ4;G3X8W7	CDC23	CDC23 cell division cycle 23	NA
Q61081	CDC37	cell division cycle 37	NA
P60766	CDC42	cell division cycle 42	NA

Q6A068	CDC5L	cell division cycle 5-like (S. pombe	NA
P11440	CDK1	cyclin-dependent kinase 1	NA
P97377	CDK2	cyclin-dependent kinase 2	NA
P30285	CDK4	cyclin-dependent kinase 4	NA
Q99J95	CDK9	cyclin-dependent kinase 9 (CDC2-	NA
Q4VAA2	CDV3	carnitine deficiency-associated ge	NA
P18760	CFL1	cofilin 1, non-muscle	NA
P45591	CFL2	cofilin 2, muscle	NA
Q9CY57	CHTOP	chromatin target of PRMT1	NA
P60824	CIRBP	cold inducible RNA binding protein	NA
Q8BMK4	CKAP4	cytoskeleton-associated protein 4	NA
A2AGT5	CKAP5	cytoskeleton associated protein 5	NA
Q04447	CKB	creatine kinase, brain	NA
Q80TV8	CLASP1	CLIP associating protein 1	NA
Q08EB5;E9Q8N5;E9	CLASP2	CLIP associating protein 2	NA
Q9Z1Q5	CLIC1	chloride intracellular channel 1	NA
O88696	CLPP	ClpP caseinolytic peptidase, ATP-	NA
Q9JHS4	CLPX	caseinolytic peptidase X (E.coli)	NA
B1AWD8;E9QM56;B	CLTA	clathrin, light polypeptide (Lca)	NA
Q68FD5	CLTC	clathrin, heavy polypeptide (Hc)	NA
Q5SW19	CLUH	clustered mitochondria (cluA/CLU	NA
P53996	CNBP	cellular nucleic acid binding protei	NA
Q9DAW9	CNN3	calponin 3, acidic	NA
Q6ZQ08	CNOT1	CCR4-NOT transcription complex	NA
P16330	CNP	2',3'-cyclic nucleotide 3' phosphod	NA
Q8K297	COLGALT1	collagen beta(1-O)galactosyltrans	NA
Q8BXC6	COMMD2	COMM domain containing 2	NA
Q8CIE6	COPA	coatomer protein complex subunit	NA
Q9JIF7	COPB1	coatomer protein complex, subuni	NA
O55029	COPB2	coatomer protein complex, subuni	NA
O89079	COPE	coatomer protein complex, subuni	NA
E9Q5P4;Q9QZE5	COPG	Coatomer subunit gamma	NA
Q9QZE5	COPG1	coatomer protein complex, subuni	NA
Q9QXK3	COPG2	coatomer protein complex, subuni	NA
Q9CQ13	COPRS	coordinator of PRMT5, differentiat	NA
P61202	COPS2	COP9 (constitutive photomorphog	NA
O88543	COPS3	COP9 (constitutive photomorphog	NA
O88544	COPS4	COP9 (constitutive photomorphog	NA
O35864	COPS5	COP9 (constitutive photomorphog	NA
O88545	COPS6	COP9 (constitutive photomorphog	NA
Q9CZ04	COPS7A	COP9 (constitutive photomorphog	NA
Q8VBV7	COPS8	COP9 (constitutive photomorphog	NA
P61924	COPZ1	coatomer protein complex, subuni	NA
Q9WUM4	CORO1C	coronin, actin binding protein 1C	NA
Q6NVF9	CPSF6	cleavage and polyadenylation spe	NA
Q9DCT8	CRIP2	cysteine rich protein 2	NA
Q9CZU6	CS	citrate synthase	NA
Q91W50	CSDE1	cold shock domain containing E1,	NA
Q9ERK4	CSE1L	chromosome segregation 1-like (S	NA
Q8BK63	CSNK1A1	casein kinase 1, alpha 1	NA
Q60737	CSNK2A1	casein kinase 2, alpha 1 polypepti	NA
O54833	CSNK2A2	casein kinase 2, alpha prime polyp	NA
P67871	CSNK2B	casein kinase 2, beta polypeptide	NA
P26231	CTNNA1	catenin (cadherin associated prote	NA
Q02248;F6QZ47;E9	CTNNB1	catenin (cadherin associated prote	NA

E9Q901;D3Z2H2;E9	CTNND1	catenin (cadherin associated prote	NA
P70698	CTPS	cytidine 5'-triphosphate synthase	NA
Q62018	CTR9	Ctr9, Paf1/RNA polymerase II com	NA
Q60598;Q921L6	CTTN	cortactin	NA
Q3TPM3;Q9WTX6	CUL1	cullin 1	NA
Q9D4H8	CUL2	cullin 2	NA
Q9JLV5	CUL3	cullin 3	NA
Q3TCH7	CUL4A	cullin 4A	NA
E9PXY1;A2A432	CUL4B	cullin 4B	NA
Q8VE73	CUL7	cullin 7	NA
Q9DCN2	CYB5R3	cytochrome b5 reductase 3	NA
Q9D0M3	CYC1	cytochrome c-1	NA
P62897	CYCS	cytochrome c, somatic	NA
Q7TMB8;E9PZS0	CYFIP1	cytoplasmic FMR1 interacting prot	NA
Q8K0C4	CYP51	cytochrome P450, family 51	NA
Q9ER88;G3X9M0	DAP3	death associated protein 3	NA
Q922B2	DARS	aspartyl-tRNA synthetase	NA
Q9JII5;Q3UGB5	DAZAP1	DAZ associated protein 1	NA
Q9QXS6	DBN1	drebrin 1	NA
Q62418	DBNL	drebrin-like	NA
Q8N7N5	DCAF8	DDB1 and CUL4 associated facto	NA
Q8BHC4	DCAKD	dephospho-CoA kinase domain co	NA
E9Q3M3;O08788;E9	DCTN1	dynactin 1	NA
Q99KJ8	DCTN2	dynactin 2	NA
Q9WUB4	DCTN6	dynactin 6	NA
Q3U1J4	DDB1	damage specific DNA binding prot	NA
O54734	DDOST	dolichyl-di-phosphooligosaccharid	NA
Q91VR5	DDX1	DEAD (Asp-Glu-Ala-Asp) box poly	NA
Q501J6	DDX17	DEAD (Asp-Glu-Ala-Asp) box poly	NA
Q61655	DDX19A	DEAD (Asp-Glu-Ala-Asp) box poly	NA
Q9JIK5	DDX21	DEAD (Asp-Glu-Ala-Asp) box poly	NA
Q8VDW0	DDX39	DEAD (Asp-Glu-Ala-Asp) box poly	NA
Q9Z1N5	DDX39B	DEAD (Asp-Glu-Ala-Asp) box poly	NA
Q62167	DDX3X	DEAD/H (Asp-Glu-Ala-Asp/His) bo	NA
Q8BTS0;Q61656	DDX5	DEAD (Asp-Glu-Ala-Asp) box poly	NA
P54823	DDX6	DEAD (Asp-Glu-Ala-Asp) box poly	NA
Q9CQ62	DECR1	2,4-dienoyl CoA reductase 1, mito	NA
Q99LB2	DHRS4	dehydrogenase/reductase (SDR fa	NA
O35286	DHX15	DEAH (Asp-Glu-Ala-His) box polyp	NA
Q6PGC1	DHX29	DEAH (Asp-Glu-Ala-His) box polyp	NA
Q99PU8	DHX30	DEAH (Asp-Glu-Ala-His) box polyp	NA
Q8VHK9	DHX36	DEAH (Asp-Glu-Ala-His) box polyp	NA
Q80X98	DHX38	DEAH (Asp-Glu-Ala-His) box polyp	NA
E9QNN1;O70133	DHX9	DEAH (Asp-Glu-Ala-His) box polyp	NA
Q9D0D4	DIMT1	DIM1 dimethyladenosine transfera	NA
Q9CSH3	DIS3	DIS3 mitotic control homolog (S. c	NA
Q9ESX5	DKC1	dyskeratosis congenita 1, dyskerin	NA
Q8BMF4	DLAT	dihydrolipoamide S-acetyltransfera	NA
O08749	DLD	dihydrolipoamide dehydrogenase	NA
Q9D2G2	DLST	dihydrolipoamide S-succinyltransfe	NA
P63037	DNAJA1	DnaJ (Hsp40) homolog, subfamily	NA
Q9QYJ0	DNAJA2	DnaJ (Hsp40) homolog, subfamily	NA
Q99KV1	DNAJB11	DnaJ (Hsp40) homolog, subfamily	NA
Q9DC23	DNAJC10	DnaJ (Hsp40) homolog, subfamily	NA
Q91YW3	DNAJC3	DnaJ (Hsp40) homolog, subfamily	NA

Q9QYI3	DNAJC7	DnaJ (Hsp40) homolog, subfamily	NA
Q6NZB0;A2ALF0;A2	DNAJC8	DnaJ (Hsp40) homolog, subfamily	NA
Q8K1M6	DNM1L	dynamin 1-like	NA
G3X9G4;Q3TCR7;F8	DNM2	dynamin 2	NA
A2A9M4;Q8R1A4;A2	DOCK7	dedicator of cytokinesis 7	NA
Q80YA7	DPP8	dipeptidylpeptidase 8	NA
P32233	DRG1	developmentally regulated GTP bi	NA
Q9QXB9	DRG2	developmentally regulated GTP bi	NA
Q91ZU6;E9PXE5;E9	DST	dystonin	NA
Q9R0P5	DSTN	destrin	NA
Q9DD18	DTD1	D-tyrosyl-tRNA deacylase 1	NA
Q9JHU4	DYNC1H1	dynein cytoplasmic 1 heavy chain	NA
O88487;Q3TPJ8;A2I	DYNC1I2	dynein cytoplasmic 1 intermediate	NA
Q8R1Q8	DYNC1LI1	dynein cytoplasmic 1 light interme	NA
Q6PDL0	DYNC1LI2	dynein, cytoplasmic 1 light interme	NA
P63168	DYNLL1	dynein light chain LC8-type 1	NA
Q9D0M5	DYNLL2	dynein light chain LC8-type 2	NA
O35459	ECH1	enoyl coenzyme A hydratase 1, pe	NA
Q8BH95	ECHS1	enoyl Coenzyme A hydratase, sho	NA
Q9WUR2	ECI2	enoyl-Coenzyme A delta isomeras	NA
Q3UJB9;D6RE33	EDC4	enhancer of mRNA decapping 4	NA
Q9JMG1	EDF1	endothelial differentiation-related f	NA
P10126	EEF1A1	eukaryotic translation elongation fa	21
O70251	EEF1B2	eukaryotic translation elongation fa	NA
E9QN08;Q80T06;D3	EEF1D	eukaryotic translation elongation fa	NA
Q9D1M4	EEF1E1	eukaryotic translation elongation fa	NA
Q9D8N0	EEF1G	eukaryotic translation elongation fa	NA
P58252	EEF2	eukaryotic translation elongation fa	NA
O08796	EEF2K	eukaryotic elongation factor-2 kina	NA
A2AH85;G3UZ34;O0	EFTUD2	elongation factor Tu GTP binding	NA
Q01279	EGFR	epidermal growth factor receptor	NA
P48024	EIF1	eukaryotic translation initiation fac	NA
Q60872	EIF1A	eukaryotic translation initiation fac	NA
Q8BMJ3	EIF1AX	eukaryotic translation initiation fac	NA
Q8BJW6	EIF2A	eukaryotic translation initiation fac	NA
Q99LC8	EIF2B1	eukaryotic translation initiation fac	NA
Q99LD9	EIF2B2	eukaryotic translation initiation fac	NA
Q61749	EIF2B4	eukaryotic translation initiation fac	NA
Q6ZWX6	EIF2S1	eukaryotic translation initiation fac	NA
Q99L45	EIF2S2	eukaryotic translation initiation fac	NA
Q9Z0N1	EIF2S3X	eukaryotic translation initiation fac	NA
P23116	EIF3A	eukaryotic translation initiation fac	NA
Q8JZQ9	EIF3B	eukaryotic translation initiation fac	NA
Q8R1B4	EIF3C	eukaryotic translation initiation fac	NA
O70194	EIF3D	eukaryotic translation initiation fac	NA
P60229	EIF3E	eukaryotic translation initiation fac	NA
Q9DCH4	EIF3F	eukaryotic translation initiation fac	NA
Q9Z1D1	EIF3G	eukaryotic translation initiation fac	NA
Q91WK2	EIF3H	eukaryotic translation initiation fac	NA
Q9QZD9	EIF3I	eukaryotic translation initiation fac	NA
Q3UGC7	EIF3J1	eukaryotic translation initiation fac	NA
Q66JS6	EIF3J2	eukaryotic translation initiation fac	NA
Q9DBZ5	EIF3K	eukaryotic translation initiation fac	NA
Q8QZY1	EIF3L	eukaryotic translation initiation fac	NA
Q99JX4	EIF3M	eukaryotic translation initiation fac	NA

P60843	EIF4A1	eukaryotic translation initiation fac	NA
P10630	EIF4A2	eukaryotic translation initiation fac	NA
Q91VC3	EIF4A3	eukaryotic translation initiation fac	NA
Q8BGD9	EIF4B	eukaryotic translation initiation fac	NA
P63073	EIF4E	eukaryotic translation initiation fac	NA
Q6NZJ6	EIF4G1	eukaryotic translation initiation fac	NA
Q62448;G3XA17;F7(	EIF4G2	eukaryotic translation initiation fac	NA
Q80XI3;A2AMI2;E9Q	EIF4G3	eukaryotic translation initiation fac	NA
Q9WUK2	EIF4H	eukaryotic translation initiation fac	NA
P59325	EIF5	eukaryotic translation initiation fac	NA
P63242	EIF5A	eukaryotic translation initiation fac	NA
Q05D44	EIF5B	eukaryotic translation initiation fac	NA
O55135	EIF6	eukaryotic translation initiation fac	NA
Q80Y81	ELAC2	elaC homolog 2 (E. coli)	NA
P70372	ELAVL1	ELAV (embryonic lethal, abnorma	NA
Q91WG4	ELP2	elongator acetyltransferase compl	NA
Q9CZX0	ELP3	elongator acetyltransferase compl	NA
NA	ELP5	elongator acetyltransferase compl	NA
Q8BK75	ELP6	elongator acetyltransferase compl	NA
Q9CRD2	EMC2	ER membrane protein complex su	NA
Q9EP72	EMC7	ER membrane protein complex su	NA
O70378	EMC8	ER membrane protein complex su	NA
O08579	EMD	emerin	NA
Q3UMY5;F8WJ93	EML4	echinoderm microtubule associate	NA
P17182;B1ARR7	ENO1	enolase 1, alpha non-neuron	NA
Q9D379;E9PWK1	EPHX1	epoxide hydrolase 1, microsomal	NA
Q8CGC7	EPRS	glutamyl-prolyl-tRNA synthetase	NA
P42567	EPS15	epidermal growth factor receptor p	NA
F8VPM7;E9QL63;Q9	ERC1	ELKS/RAB6-interacting/CAST fan	NA
Q9DC16	ERGIC1	endoplasmic reticulum-golgi interr	NA
Q8BFZ9	ERLIN2	ER lipid raft associated 2	NA
Q8BWY3	ETF1	eukaryotic translation termination	NA
Q99LC5	ETFA	electron transferring flavoprotein, a	NA
Q61545	EWSR1	Ewing sarcoma breakpoint region	NA
Q9DAA6	EXOSC1	exosome component 1	NA
Q8VBV3	EXOSC2	exosome component 2	NA
Q921I9	EXOSC4	exosome component 4	NA
Q9CRA8	EXOSC5	exosome component 5	NA
Q8BTW3	EXOSC6	Exosome complex component MT	NA
Q9D753	EXOSC8	exosome component 8	NA
P51880	FABP7	fatty acid binding protein 7, brain	NA
P54731	FAF1	Fas-associated factor 1	NA
Q6A0A9	FAM120A	family with sequence similarity 12(	NA
Q8C3F2	FAM120C	family with sequence similarity 12(	NA
Q3TCJ1;D3Z4D8;G3	FAM175B	family with sequence similarity 17!	NA
Q80VD1	FAM98B	family with sequence similarity 98,	NA
Q8C0C7	FARSA	phenylalanyl-tRNA synthetase, alp	NA
Q9WUA2	FARSB	phenylalanyl-tRNA synthetase, be	NA
P19096	FASN	fatty acid synthase	NA
P35550	FBL	fibrillarin	NA
P37889	FBLN2	fibulin 2	NA
Q80UW2	FBXO2	F-box protein 2	NA
Q91Z50;E9PYV9;P3	FEN1	flap structure specific endonuclea:	NA
F2Z455;A6H6N4	FHL3	four and a half LIM domains 3	NA
Q62446	FKBP3	FK506 binding protein 3	NA

P30416	FKBP4	FK506 binding protein 4	NA
Q9Z247	FKBP9	FK506 binding protein 9	NA
Q9JJ28	FLII	flightless I homolog (Drosophila)	NA
B7FAU9;Q8BTM8;B7	FLNA	filamin, alpha	NA
Q80X90	FLNB	filamin, beta	NA
D3Z6U8;P35922;E9(	FMR1	fragile X mental retardation syndro	NA
Q80TY0;D3YYV4;A2	FNBP1	formin binding protein 1	NA
F8VQ05	FRYL	furry homolog-like (Drosophila)	NA
Q61553	FSCN1	fascin homolog 1, actin bundling p	NA
P29391;Q9CPX4	FTL1	ferritin light chain 1	NA
Q3TUE1;Q91WJ8	FUBP1	far upstream element (FUSE) binc	NA
Q3TIX6;A2AJ72	FUBP3	far upstream element (FUSE) binc	NA
P56959	FUS	fused in sarcoma	NA
Q61584	FXR1	fragile X mental retardation gene 1	NA
F6QH50	FXR2	fragile X mental retardation, autos	NA
P97855	G3BP1	GTPase activating protein (SH3 do	NA
P97379	G3BP2	GTPase activating protein (SH3 do	NA
P60521	GABARAPL2	gamma-aminobutyric acid (GABA)	NA
Q8BHN3	GANAB	alpha glucosidase 2 alpha neutral	NA
P16858	GAPDH	glyceraldehyde-3-phosphate dehy	2
Q6PAR5	GAPVD1	GTPase activating protein and VP	NA
Q9CY66	GAR1	GAR1 ribonucleoprotein homolog	NA
Q9CZD3	GARS	glycyl-tRNA synthetase	NA
Q7TMG8;O55126	GBAS	glioblastoma amplified sequence	NA
E9PVA8	GCN1L1	GCN1 general control of amino-ac	NA
Q9CRY7	GDPD1	glycerophosphodiester phosphodi	NA
Q8BX17	GEMIN5	gem (nuclear organelle) associate	NA
Q9D1H7	GET4	golgi to ER traffic protein 4 homolo	NA
Q9D7M1	GID8	GID complex subunit 8 homolog (S	NA
Q91W43	GLDC	glycine decarboxylase	NA
Q61543	GLG1	golgi apparatus protein 1	NA
Q9CQM9	GLRX3	glutaredoxin 3	NA
P26443	GLUD1	glutamate dehydrogenase 1	NA
E9QAZ2	GM10020	predicted pseudogene 10020	NA
E9PYL9	GM10036	predicted gene 10036	NA
E9Q3T0	GM10073	predicted pseudogene 10073	NA
D3YYI8	GM10093	predicted pseudogene 10093	NA
D3Z5M2	GM10110	predicted gene 10110	NA
D3Z6C3	GM10119	Uncharacterized protein	NA
D3Z426	GM10120	MCG49049	NA
P17742	GM10123	predicted pseudogene 10123	NA
P62751	GM10132	predicted gene 10132	NA
Q9CQQ8	GM10146	predicted gene 10146	NA
D3Z4H3	GM10154	Uncharacterized protein	NA
P52293	GM10184	predicted pseudogene 10184	NA
E9PV77	GM10257	Uncharacterized protein	NA
F5H8M6	GM10260	predicted gene 10260	NA
D3Z3R2	GM10268	Uncharacterized protein	NA
P35979	GM10275	predicted pseudogene 10275	NA
D3Z3K1	GM10288	predicted gene 10288	NA
E9Q5U3	GM10358	Glyceraldehyde-3-phosphate dehy	2
F6VWT4	GM10362	Uncharacterized protein	NA
P62984	GM11808	predicted gene 11808	NA
Q8CBB6;Q921L4	GM13646	Histone H2B	NA
D3Z3R0	GM16477	predicted pseudogene 16477	NA

F6QL70	GM17669	predicted gene, 17669	NA
Q6ZWV7	GM2000	predicted gene 2000	NA
E9PX42	GM2606	Glyceraldehyde-3-phosphate dehy	2
E9PWH9	GM4294	Ribosomal protein L15	NA
E9Q1S8	GM4609	Glyceraldehyde-3-phosphate dehy	2
D3YXG3	GM4963	Uncharacterized protein	NA
E9PZB3	GM5093	predicted gene 5093	NA
D3Z4J0	GM5436	MCG51900	NA
D3Z5N9	GM5449	predicted pseudogene 5449	NA
E9PX72	GM5559	Glyceraldehyde-3-phosphate dehy	2
E9PV04	GM5576	Uncharacterized protein	NA
Q8BG05	GM5641	predicted gene 5641	NA
D3Z3R1	GM5745	60S ribosomal protein L36	NA
E9Q2T1	GM5848	Uncharacterized protein	NA
D3Z6J9	GM5908	Uncharacterized protein	NA
D3YWT9	GM5921	Uncharacterized protein	NA
D3YWA0	GM6139	predicted gene 6139	NA
Q9D1J3	GM6563	predicted pseudogene 6563	NA
D3Z4I7	GM6807	60S ribosomal protein L6	NA
D3YXU0	GM7263	Uncharacterized protein	NA
P14115	GM7536	predicted gene 7536	NA
F6X7C4	GM7866	Uncharacterized protein	NA
P12970	GM8226	predicted gene 8226	NA
E9PV46	GM8841	Uncharacterized protein	NA
E9Q5F8	GM9049	Uncharacterized protein	NA
D3YVN7	GM9755	predicted pseudogene 9755	NA
Q8C854	GM9833	predicted gene 9833	NA
Q6ZWU9	GM9846	predicted gene 9846	NA
Q3THK7	GMPS	guanine monophosphate syntheta	NA
P08752	GNAI2	guanine nucleotide binding protein	NA
P62874	GNB1	guanine nucleotide binding protein	NA
P62880	GNB2	guanine nucleotide binding protein	NA
P68040	GNB2L1	guanine nucleotide binding protein	NA
P36916	GNL1	guanine nucleotide binding protein	NA
Q8CI11	GNL3	guanine nucleotide binding protein	NA
Q99LD4;Q3MIA8;G3	GPS1	G protein pathway suppressor 1	NA
Q8BGT5	GPT2	glutamic pyruvate transaminase (a	NA
P11352	GPX1	glutathione peroxidase 1	NA
P10649	GSTM1	glutathione S-transferase, mu 1	NA
P19157	GSTP1	glutathione S-transferase, pi 1	NA
P62915	GTF2B	general transcription factor IIB	NA
Q9D0D5	GTF2E1	general transcription factor II E, po	NA
Q9D902	GTF2E2	general transcription factor II E, po	NA
Q3THK3	GTF2F1	general transcription factor IIF, po	NA
Q8R0A0	GTF2F2	general transcription factor IIF, po	NA
Q9ESZ8	GTF2I	general transcription factor II I	NA
O08582	GTPBP1	GTP binding protein 1	NA
P10922	H1F0	H1 histone family, member 0	NA
Q8R1M2	H2AFJ	H2A histone family, member J	NA
Q3THW5	H2AFV	H2A histone family, member V	NA
P27661	H2AFX	H2A histone family, member X	NA
P0C0S6	H2AFZ	H2A histone family, member Z	NA
P50171	H2-KE6	H2-K region expressed gene 6	NA
P84244	H3F3A	H3 histone, family 3A	NA
Q61425	HADH	hydroxyacyl-Coenzyme A dehydro	NA

Q8BMS1	HADHA	hydroxyacyl-Coenzyme A dehydro	NA
Q99JY0	HADHB	hydroxyacyl-Coenzyme A dehydro	NA
Q61035	HARS	histidyl-tRNA synthetase	NA
Q99KK9	HARS2	histidyl-tRNA synthetase 2, mitoch	NA
Q8BY71	HAT1	histone aminotransferase 1	NA
O09106	HDAC1	histone deacetylase 1	NA
P70288	HDAC2	histone deacetylase 2	NA
P51859	HDGF	hepatoma-derived growth factor	NA
Q3UMU9	HDGFRP2	hepatoma-derived growth factor, r	NA
Q8VDJ3	HDLBP	high density lipoprotein (HDL) binc	NA
Q69ZR2	HECTD1	HECT domain containing 1	NA
P29416	HEXA	hexosaminidase A	NA
Q8R409	HEXIM1	hexamethylene bis-acetamide indu	NA
Q8VD75	HIP1	huntingtin interacting protein 1	NA
P43275	HIST1H1A	histone cluster 1, H1a	NA
P43276	HIST1H1B	histone cluster 1, H1b	NA
P15864	HIST1H1C	histone cluster 1, H1c	NA
P43277	HIST1H1D	histone cluster 1, H1d	NA
P43274	HIST1H1E	histone cluster 1, H1e	NA
P22752	HIST1H2AB	histone cluster 1, H2ab	NA
Q8CGP5	HIST1H2AF	histone cluster 1, H2af	NA
Q8CGP6	HIST1H2AH	histone cluster 1, H2ah	NA
Q8CGP7	HIST1H2AK	histone cluster 1, H2ak	NA
F8WIX8	HIST1H2AL	histone cluster 1, H2al	NA
Q64475	HIST1H2BB	histone cluster 1, H2bb	NA
Q6ZWY9	HIST1H2BC	histone cluster 1, H2bc	NA
P10853	HIST1H2BF	histone cluster 1, H2bf	NA
Q64478	HIST1H2BH	histone cluster 1, H2bh	NA
Q8CGP1	HIST1H2BK	histone cluster 1, H2bk	NA
P10854	HIST1H2BM	histone cluster 1, H2bm	NA
Q8CGP2	HIST1H2BP	histone cluster 1, H2bp	NA
P68433	HIST1H3A	histone cluster 1, H3a	NA
P84228	HIST1H3B	histone cluster 1, H3b	NA
P62806	HIST1H4A	histone cluster 1, H4a	NA
Q6GSS7	HIST2H2AA1	histone cluster 2, H2aa1	NA
Q64523	HIST2H2AC	histone cluster 2, H2ac	NA
Q64525	HIST2H2BB	histone cluster 2, H2bb	NA
Q8BFU2	HIST3H2A	histone cluster 3, H2a	NA
P30681	HMGB2	high mobility group box 2	NA
Q9CX86	HNRNPA0	heterogeneous nuclear ribonucleo	NA
Q5EBP8;P49312	HNRNPA1	heterogeneous nuclear ribonucleo	NA
O88569	HNRNPA2B1	heterogeneous nuclear ribonucleo	NA
Q8BG05	HNRNPA3	heterogeneous nuclear ribonucleo	NA
Q99020	HNRNPAB	heterogeneous nuclear ribonucleo	NA
Q9Z204	HNRNPC	heterogeneous nuclear ribonucleo	NA
Q60668	HNRNPD	heterogeneous nuclear ribonucleo	NA
Q9Z130	HNRNPDL	heterogeneous nuclear ribonucleo	NA
Q9Z2X1	HNRNPF	heterogeneous nuclear ribonucleo	NA
O35737	HNRNPH1	heterogeneous nuclear ribonucleo	NA
P70333	HNRNPH2	heterogeneous nuclear ribonucleo	NA
D3YWT1;D3Z3N4	HNRNPH3	heterogeneous nuclear ribonucleo	NA
P61979	HNRNPK	heterogeneous nuclear ribonucleo	NA
Q8R081	HNRNPL	heterogeneous nuclear ribonucleo	NA
NA	HNRNPLL	heterogeneous nuclear ribonucleo	NA
Q9D0E1	HNRNPM	heterogeneous nuclear ribonucleo	NA

Q8VHM5;A2AW41	HNRNPR	heterogeneous nuclear ribonucleo	NA
G3XA10;Q8VEK3	HNRNPU	heterogeneous nuclear ribonucleo	NA
Q8VDM6	HNRNPUL1	heterogeneous nuclear ribonucleo	NA
Q00PI9	HNRNPUL2	heterogeneous nuclear ribonucleo	NA
D3YTQ3;Q9Z130;F6	HNRPDL	Uncharacterized protein	NA
Q921F4;F6SB69	HNRPLL	Heterogeneous nuclear ribonucleo	NA
Q8BUK6	HOOK3	hook homolog 3 (Drosophila)	NA
Q99N15;A2AFQ2;O(	HSD17B10	hydroxysteroid (17-beta) dehydrog	NA
O70503	HSD17B12	hydroxysteroid (17-beta) dehydrog	NA
P51660	HSD17B4	hydroxysteroid (17-beta) dehydrog	NA
Q2TPA8	HSDL2	hydroxysteroid dehydrogenase like	NA
P07901	HSP90AA1	heat shock protein 90, alpha (cyto	NA
P11499	HSP90AB1	heat shock protein 90 alpha (cytos	NA
P08113	HSP90B1	heat shock protein 90, beta (Grp9	NA
Q99M31	HSPA14	heat shock protein 14	NA
Q61696	HSPA1A	heat shock protein 1A	NA
P17879	HSPA1B	heat shock protein 1B	NA
P17156	HSPA2	heat shock protein 2	NA
Q3U2G2	HSPA4	heat shock protein 4	NA
P20029	HSPA5	heat shock protein 5	NA
P63017	HSPA8	heat shock protein 8	NA
P38647	HSPA9	heat shock protein 9	NA
P63038	HSPD1	heat shock protein 1 (chaperonin)	NA
Q8BGC0	HTATSF1	HIV TAT specific factor 1	NA
A2AFQ0;Q7TMY8	HUWE1	HECT, UBA and WWE domain co	NA
Q9JKR6	HYOU1	hypoxia up-regulated 1	NA
Q8BU30	IARS	isoleucine-tRNA synthetase	NA
Q9D6R2	IDH3A	isocitrate dehydrogenase 3 (NAD)	NA
Q91VA7	IDH3B	isocitrate dehydrogenase 3 (NAD)	NA
Q07113	IGF2R	insulin-like growth factor 2 recepto	NA
Q7TT37	IKBKAP	inhibitor of kappa light polypeptide	NA
Q9CXY6	ILF2	interleukin enhancer binding facto	NA
Q9Z1X4	ILF3	interleukin enhancer binding facto	NA
Q8CAQ8	IMMT	inner membrane protein, mitochor	NA
P24547	IMPDH2	inosine 5'-phosphate dehydrogena	NA
P46660	INA	internexin neuronal intermediate fi	NA
Q8K2V6	IPO11	importin 11	NA
Q8VI75	IPO4	importin 4	NA
Q8BKC5	IPO5	importin 5	NA
Q9EPL8	IPO7	importin 7	NA
E9QKZ2;Q91YE6	IPO9	importin 9	NA
Q9JKF1	IQGAP1	IQ motif containing GTPase activa	NA
Q3UQ44	IQGAP2	IQ motif containing GTPase activa	NA
P09055	ITGB1	integrin beta 1 (fibronectin recepto	NA
Q99MN1	KARS	lysyl-tRNA synthetase	NA
Q6WVG3	KCTD12	BTB/POZ domain-containing prote	NA
Q9JHP7	KDELC1	KDEL (Lys-Asp-Glu-Leu) containir	NA
Q60749	KHDRBS1	KH domain containing, RNA bindi	NA
Q3U0V1	KHSRP	KH-type splicing regulatory proteir	NA
Q61768	KIF5B	kinesin family member 5B	NA
Q60960	KPNA1	karyopherin (importin) alpha 1	NA
O35344	KPNA3	karyopherin (importin) alpha 3	NA
O35343	KPNA4	karyopherin (importin) alpha 4	NA
O35345	KPNA6	karyopherin (importin) alpha 6	NA
P70168	KPNB1	karyopherin (importin) beta 1	NA

P04104	KRT1	keratin 1	NA
A2A513;P02535;E9Q	KRT10	keratin 10	NA
Q61414	KRT15	keratin 15	NA
Q9Z2K1	KRT16	keratin 16	NA
P19001	KRT19	keratin 19	NA
Q3TTY5	KRT2	keratin 2	NA
Q61765	KRT31	keratin 31	NA
Q62168	KRT32	keratin 32	NA
Q61897	KRT33B	keratin 33B	NA
P07744	KRT4	keratin 4	NA
Q6IFX2	KRT42	keratin 42	NA
Q922U2	KRT5	keratin 5	NA
P50446	KRT6A	keratin 6A	NA
Q9Z331	KRT6B	keratin 6B	NA
Q9DCV7	KRT7	keratin 7	NA
Q8BGZ7	KRT75	keratin 75	NA
Q6IFZ6	KRT77	keratin 77	NA
E9Q464;E9Q0F0	KRT78	keratin 78	NA
Q8VED5	KRT79	keratin 79	NA
Q99M74	KRT82	keratin 82	NA
Q9Z2T6	KRT85	keratin, type II cuticular Hb5	NA
E9QJY4;Q61595;F8V	KTN1	kinectin 1	NA
Q6ZQ58	LARP1	La ribonucleoprotein domain famil	NA
G3X9Q6;F6YE50;E9	LARP4	La ribonucleoprotein domain famil	NA
Q6A0A2	LARP4B	La ribonucleoprotein domain famil	NA
Q05CL8	LARP7	La ribonucleoprotein domain famil	NA
Q8BMJ2	LARS	leucyl-tRNA synthetase	NA
A2BE28	LAS1L	LAS1-like (S. cerevisiae)	NA
Q61792;A2A6G9;A2	LASP1	LIM and SH3 protein 1	NA
P06151	LDHA	lactate dehydrogenase A	NA
P35951	LDLR	low density lipoprotein receptor	NA
Q5XJE5	LEO1	Leo1, Paf1/RNA polymerase II co	NA
P16045	LGALS1	lectin, galactose binding, soluble 1	NA
Q9ERG0	LIMA1	LIM domain and actin binding 1	NA
Q80Y17	LLGL1	lethal giant larvae homolog 1 (Dro	NA
Q9DBH5	LMAN2	lectin, mannose-binding 2	NA
P48678	LMNA	lamin A	NA
P14733	LMNB1	lamin B1	NA
P21619	LMNB2	lamin B2	NA
Q8CGK3	LONP1	lon peptidase 1, mitochondrial	NA
Q9DBN5	LONP2	lon peptidase 2, peroxisomal	NA
Q8VI56	LRP4	low density lipoprotein receptor-re	NA
Q6PB66	LRPPRC	leucine-rich PPR-motif containing	NA
E9PV22;Q505F5	LRRC47	leucine rich repeat containing 47	NA
Q922Q8	LRRC59	leucine rich repeat containing 59	NA
Q3UZ39	LRRFIP1	leucine rich repeat (in FLII) interac	NA
Q9D0R8	LSM12	LSM12 homolog (S. cerevisiae)	NA
O35900	LSM2	LSM2 homolog, U6 small nuclear	NA
Q9QXA5	LSM4	LSM4 homolog, U6 small nuclear	NA
Q6A009	LTN1	listerin E3 ubiquitin protein ligase 1	NA
Q6NSQ7	LTV1	LTV1 homolog (S. cerevisiae)	NA
E9PX54;E9Q715;Q7	LUC7L2	LUC7-like 2 (S. cerevisiae)	NA
P70202	LXN	latexin	NA
Q08288	LYAR	Ly1 antibody reactive clone	NA
Q9WTL7	LYPLA2	lysophospholipase 2	NA

E9QA63;B1ARU4;Q9	MACF1	microtubule-actin crosslinking fact	NA
Q9QYH6	MAGED1	melanoma antigen, family D, 1	NA
Q9CQY5	MAGT1	magnesium transporter 1	NA
Q3TMX5;Q80ZP8;Q9	MANF	mesencephalic astrocyte-derived n	NA
Q64133	MAOA	monoamine oxidase A	NA
Q9QYR6	MAP1A	microtubule-associated protein 1 A	NA
P14873	MAP1B	microtubule-associated protein 1B	NA
Q8C052	MAP1S	microtubule-associated protein 1S	NA
P20357	MAP2	microtubule-associated protein 2	NA
P31938	MAP2K1	mitogen-activated protein kinase k	NA
P27546	MAP4	microtubule-associated protein 4	NA
F7BS72;E9PVG7;F8	MAP4K4	mitogen-activated protein kinase k	NA
P63085	MAPK1	mitogen-activated protein kinase 1	NA
Q63844;D3Z3G6	MAPK3	mitogen-activated protein kinase 3	NA
Q61166	MAPRE1	microtubule-associated protein, R	NA
P26645	MARCKS	myristoylated alanine rich protein k	NA
P28667	MARCKSL1	MARCKS-like 1	NA
Q68FL6	MARS	methionine-tRNA synthetase	NA
E9PUP5;E9PUR0;E9	MASKBP3	Uncharacterized protein	NA
Q8K310	MATR3	matrin 3	NA
G3XA55	MCG_10837	MCG10837	NA
G3UWG1	MCG_115977	MCG115977	NA
G3UW34	MCG_130981	MCG130981	NA
G3X9L6	MCG_55033	MCG55033	NA
P97310	MCM2	minichromosome maintenance de	NA
P25206	MCM3	minichromosome maintenance de	NA
P49717	MCM4	minichromosome maintenance de	NA
P49718;Q52KC3	MCM5	minichromosome maintenance de	NA
P97311	MCM6	minichromosome maintenance de	NA
Q61881	MCM7	minichromosome maintenance de	NA
P08249	MDH2	malate dehydrogenase 2, NAD (m	NA
A2ANY6	MDN1	midasin homolog (yeast)	NA
Q99KE1	ME2	malic enzyme 2, NAD()-dependen	NA
Q91VH6	MEMO1	mediator of cell motility 1	NA
Q8K3A9	MEPCE	methylphosphate capping enzyme	NA
Q8BP48	METAP1	methionyl aminopeptidase 1	NA
D3Z7D2;Q9Z120	METTL1	methyltransferase like 1	NA
Q9CQ20	MID1IP1	Mid1 interacting protein 1 (gastrula	NA
Q6ZQI3;D3Z1M3	MLEC	malectin	NA
E9PYX7;E9Q852;E9	MLLT4	myeloid/lymphoid or mixed-lineage	NA
Q6PEB6	MOB4	MOB family member 4, phocein	NA
E0CYJ0;E0CXA9	MOBKL3	Uncharacterized protein	NA
Q9CQT1	MRI1	methylthioribose-1-phosphate isor	NA
Q99N96	MRPL1	mitochondrial ribosomal protein L1	NA
Q9D1P0	MRPL13	mitochondrial ribosomal protein L1	NA
Q9CPR5	MRPL15	mitochondrial ribosomal protein L1	NA
Q9D8P4	MRPL17	mitochondrial ribosomal protein L1	NA
Q9CQL5	MRPL18	mitochondrial ribosomal protein L1	NA
Q9D338	MRPL19	mitochondrial ribosomal protein L1	NA
Q9D773	MRPL2	mitochondrial ribosomal protein L2	NA
Q9D1N9	MRPL21	mitochondrial ribosomal protein L2	NA
Q8BU88	MRPL22	mitochondrial ribosomal protein L2	NA
O35972	MRPL23	mitochondrial ribosomal protein L2	NA
Q921S7	MRPL37	mitochondrial ribosomal protein L3	NA
Q8K2M0	MRPL38	mitochondrial ribosomal protein L3	NA

Q9JKF7	MRPL39	mitochondrial ribosomal protein L3	NA
Q9DCU6	MRPL4	mitochondrial ribosomal protein L4	NA
Q9Z2Q5	MRPL40	mitochondrial ribosomal protein L4	NA
Q9CY73	MRPL44	mitochondrial ribosomal protein L4	NA
Q9D0Q7	MRPL45	mitochondrial ribosomal protein L4	NA
Q8K2Y7	MRPL47	mitochondrial ribosomal protein L4	NA
Q99N94	MRPL9	mitochondrial ribosomal protein L9	NA
Q9DCA2;Q3U8Y1	MRPS11	mitochondrial ribosomal protein S1	NA
Q9DC71	MRPS15	mitochondrial ribosomal protein S1	NA
Q924T2	MRPS2	mitochondrial ribosomal protein S2	NA
Q9CXW2	MRPS22	mitochondrial ribosomal protein S2	NA
Q8VE22	MRPS23	mitochondrial ribosomal protein S2	NA
Q9D125	MRPS25	mitochondrial ribosomal protein S2	NA
Q80ZS3	MRPS26	mitochondrial ribosomal protein S2	NA
Q8BK72	MRPS27	mitochondrial ribosomal protein S2	NA
Q9D0G0	MRPS30	mitochondrial ribosomal protein S3	NA
Q61733	MRPS31	mitochondrial ribosomal protein S3	NA
Q9JIK9	MRPS34	mitochondrial ribosomal protein S3	NA
Q8BJZ4	MRPS35	mitochondrial ribosomal protein S3	NA
Q99N87	MRPS5	mitochondrial ribosomal protein S5	NA
Q80X85	MRPS7	mitchondrial ribosomal protein S7	NA
Q9D7N3	MRPS9	mitochondrial ribosomal protein S9	NA
Q9D0I8	MRTO4	MRT4, mRNA turnover 4, homolog	NA
Q61474	MSI1	musashi RNA-binding protein 1	NA
Q920Q6	MSI2	musashi RNA-binding protein 2	NA
P26041	MSN	moesin	NA
A2ARP8	MTAP1A	Microtubule-associated protein 1 A	NA
G3UZJ2;Q3TLQ0;G3	MTAP2	Uncharacterized protein	NA
Q791V5	MTCH2	mitochondrial carrier homolog 2 (C	NA
P00405	MT-CO2	mitochondrially encoded cytochror	NA
Q80WJ7	MTDH	metadherin	NA
Q922D8	MTHFD1	methylenetetrahydrofolate dehydro	NA
Q3V3R1	MTHFD1L	methylenetetrahydrofolate dehydro	NA
P18155	MTHFD2	methylenetetrahydrofolate dehydro	NA
Q5SX40	MYH1	myosin, heavy polypeptide 1, skele	NA
Q61879	MYH10	myosin, heavy polypeptide 10, nor	NA
Q6URW6	MYH14	myosin, heavy polypeptide 14	NA
Q5SX41;G3UW82	MYH2	myosin, heavy polypeptide 2, skele	NA
Q5SX39	MYH4	myosin, heavy polypeptide 4, skele	NA
A2AQP0	MYH7B	myosin, heavy chain 7B, cardiac m	NA
P13542	MYH8	myosin, heavy polypeptide 8, skele	NA
Q8VDD5	MYH9	myosin, heavy polypeptide 9, non-	NA
P05977	MYL1	myosin, light polypeptide 1	NA
Q60605	MYL6	myosin, light polypeptide 6, alkali,	NA
Q9JMH9	MYO18A	myosin XVIIIA	NA
P46735	MYO1B	myosin IB	NA
Q99104	MYO5A	myosin VA	NA
Q9QY36	NAA10	N(alpha)-acetyltransferase 10, Na	NA
Q80UM3;G3X8Y3	NAA15	N(alpha)-acetyltransferase 15, Na	NA
Q8BWZ3	NAA25	N(alpha)-acetyltransferase 25, Na	NA
Q6PGB6	NAA50	N(alpha)-acetyltransferase 50, Na	NA
P70670	NACA	nascent polypeptide-associated co	NA
Q8BSH9;E9PW66;P	NAP1L1	nucleosome assembly protein 1-lik	NA
Q78ZA7	NAP1L4	nucleosome assembly protein 1-lik	NA
Q9DB05	NAPA	N-ethylmaleimide sensitive fusion	NA

Q8BP47	NARS	asparaginyl-tRNA synthetase	NA
B1AU75	NASP	nuclear autoantigenic sperm prote	NA
F7C5V8;P13595;E9(	NCAM1	neural cell adhesion molecule 1	NA
Q3UYV9	NCBP1	nuclear cap binding protein subun	NA
P28660	NCKAP1	NCK-associated protein 1	NA
P09405	NCL	nucleolin	NA
Q9CZA6	NDE1	nuclear distribution gene E homol	NA
Q9DCT2	NDUFS3	NADH dehydrogenase (ubiquinon	NA
Q9D1J1	NECAP2	NECAP endocytosis associated 2	NA
P46935	NEDD4	neural precursor cell expressed, d	NA
Q9ES74	NEK7	NIMA (never in mitosis gene a)-re	NA
Q922L6	NELFCD	negative elongation factor comple	NA
Q8CCP0	NEMF	nuclear export mediator factor	NA
Q6P5H2	NES	nestin	NA
B1AUC0;F8WH09;F	NFIA	nuclear factor I/A	NA
P97863;F8WHI4;F8\	NFIB	nuclear factor I/B	NA
Q9CRB2	NHP2	NHP2 ribonucleoprotein	NA
Q9CQE1	NIPSNAP3B	nipsnap homolog 3B (C. elegans)	NA
Q80TM9	NISCH	nischarin	NA
O70310	NMT1	N-myristoyltransferase 1	NA
E9PVC9;E9Q5C9	NOLC1	nucleolar and coiled-body phosph	NA
Q6GQT9	NOMO1	nodal modulator 1	NA
Q99K48;F6XLC7	NONO	non-POU-domain-containing, octa	NA
Q9CPT5	NOP16	NOP16 nucleolar protein	NA
Q9D6Z1	NOP56	NOP56 ribonucleoprotein	NA
Q6DFW4	NOP58	NOP58 ribonucleoprotein	NA
Q9D6T0	NOSIP	nitric oxide synthase interacting pr	NA
P60670	NPLOC4	nuclear protein localization 4 hom	NA
Q61937	NPM1	nucleophosmin 1	NA
Q9CPP0	NPM3	nucleoplasmin 3	NA
Q8BHG1	NRD1	nardilysin, N-arginine dibasic conv	NA
Q9CZ44	NSFL1C	NSFL1 (p97) cofactor (p47)	NA
Q1HFZ0	NSUN2	NOL1/NOP2/Sun domain family m	NA
Q91X76	NT5DC2	5'-nucleotidase domain containing	NA
P54729	NUB1	negative regulator of ubiquitin-like	NA
O35685	NUDC	nuclear distribution gene C homol	NA
Q8VHN8	NUDT16L1	nudix (nucleoside diphosphate linl	NA
Q9CQF3	NUDT21	nudix (nucleoside diphosphate linl	NA
E9Q7G0	NUMA1	nuclear mitotic apparatus protein	NA
Q6ZQG1;F6PXL5	NUP205	nucleoporin 205	NA
Q9JIH2	NUP50	nucleoporin 50	NA
Q8BJ71	NUP93	nucleoporin 93	NA
Q99JX7	NXF1	nuclear RNA export factor 1	NA
D3YYU8	OBSL1	obscurin-like 1	NA
Q60597	OGDH	oxoglutarate (alpha-ketoglutarate)	NA
E9Q7L0	OGDHL	oxoglutarate dehydrogenase-like	NA
Q9CZ30	OLA1	Obg-like ATPase 1	NA
Q8BWU5	OSGEP	O-sialoglycoprotein endopeptidas	NA
Q7TQI3	OTUB1	OTU domain, ubiquitin aldehyde b	NA
Q8K2H2	OTUD6B	OTU domain containing 6B	NA
Q6P9R2	OXSR1	oxidative-stress responsive 1	NA
Q60715	P4HA1	procollagen-proline, 2-oxoglutarat	NA
P09103	P4HB	prolyl 4-hydroxylase, beta polypep	NA
P50580	PA2G4	proliferation-associated 2G4	NA
P29341	PABPC1	poly(A) binding protein, cytoplasm	NA

A3KFU5;A3KFU8;Q9	PABPC4	poly(A) binding protein, cytoplasm	NA
D3Z055;G3UY42;D3	PABPN1	poly(A) binding protein, nuclear 1	NA
Q9WVE8	PACSIN2	protein kinase C and casein kinas	NA
Q8K2T8	PAF1	Paf1, RNA polymerase II associat	NA
Q9DCL9	PAICS	phosphoribosylaminoimidazole ca	NA
Q921K2	PARP1	poly (ADP-ribose) polymerase fan	NA
Q9D0B6	PBDC1	polysaccharide biosynthesis doma	NA
Q3TVI8	PBXIP1	pre B cell leukemia transcription fa	NA
P60335	PCBP1	poly(rC) binding protein 1	NA
Q61990	PCBP2	poly(rC) binding protein 2	NA
E0CYV0;P23506;F7I	PCMT1	protein-L-isoaspartate (D-aspartat	NA
P17918	PCNA	proliferating cell nuclear antigen	NA
Q3UHX2	PDAP1	PDGFA associated protein 1	NA
Q61823	PDCD4	programmed cell death 4	NA
Q3TIU4	PDE12	phosphodiesterase 12	NA
P35486	PDHA1	pyruvate dehydrogenase E1 alpha	NA
Q9D051	PDHB	pyruvate dehydrogenase (lipoamic	NA
P27773	PDIA3	protein disulfide isomerase associ	NA
P08003	PDIA4	protein disulfide isomerase associ	NA
Q3TML0;Q922R8	PDIA6	protein disulfide isomerase associ	NA
Q8CI51	PDLIM5	PDZ and LIM domain 5	NA
Q3UXD9;Q99MZ7	PECR	peroxisomal trans-2-enoyl-CoA re	NA
E9Q3F7;Q7TN75	PEG10	paternally expressed 10	NA
P47857	PFKM	phosphofructokinase, muscle	NA
P62962	PFN1	profilin 1	NA
Q9JJV2	PFN2	profilin 2	NA
Q9DBJ1	PGAM1	phosphoglycerate mutase 1	NA
Q9JJT9;F6RPD4	PHAX	phosphorylated adaptor for RNA e	NA
P67778	PHB	prohibitin	NA
O35129	PHB2	prohibitin 2	NA
Q61753	PHGDH	3-phosphoglycerate dehydrogenas	NA
Q7M6Y3	PICALM	phosphatidylinositol binding clathri	NA
Q9CWW6	PIN4	protein (peptidyl-prolyl cis/trans iso	NA
P52480	PKM	pyruvate kinase, muscle	NA
P27612	PLAA	phospholipase A2, activating prote	NA
P51432	PLCB3	phospholipase C, beta 3	NA
Q8K3R3	PLCD4	phospholipase C, delta 4	NA
E9Q153;Q9QXS1;E9	PLEC	plectin	NA
Q9R0E1	PLOD3	procollagen-lysine, 2-oxoglutarate	NA
Q9CPS7	PNO1	partner of NOB1 homolog (S. cere	NA
Q8BYB9	POGLUT1	protein O-glucosyltransferase 1	NA
P52432	POLR1C	polymerase (RNA) I polypeptide C	NA
P08775	POLR2A	polymerase (RNA) II (DNA directe	NA
Q8CFI7	POLR2B	polymerase (RNA) II (DNA directe	NA
Q99M46;P97760;E9	POLR2C	polymerase (RNA) II (DNA directe	NA
Q80UW8	POLR2E	polymerase (RNA) II (DNA directe	NA
P62488	POLR2G	polymerase (RNA) II (DNA directe	NA
Q923G2	POLR2H	polymerase (RNA) II (DNA directe	NA
Q8K205;Q9D4G5	POP1	processing of precursor 1, ribonuc	NA
P24369	PPIB	peptidylprolyl isomerase B	NA
Q9CR16	PPID	peptidylprolyl isomerase D (cyclop	NA
Q9D868	PPIH	peptidyl prolyl isomerase H	NA
Q80TL0	PPM1E	protein phosphatase 1E (PP2C dc	NA
Q61074	PPM1G	protein phosphatase 1G (formerly	NA
P62137	PPP1CA	protein phosphatase 1, catalytic su	NA

P62141	PPP1CB	protein phosphatase 1, catalytic su	NA
P63087	PPP1CC	protein phosphatase 1, catalytic su	NA
Q9DBR7	PPP1R12A	protein phosphatase 1, regulatory	NA
P63330	PPP2CA	protein phosphatase 2 (formerly 2	NA
P62715	PPP2CB	protein phosphatase 2 (formerly 2	NA
Q76MZ3	PPP2R1A	protein phosphatase 2 (formerly 2	NA
Q3TTF6;Q7TNP2;G3	PPP2R1B	protein phosphatase 2 (formerly 2	NA
Q6P1F6	PPP2R2A	protein phosphatase 2 (formerly 2	NA
Q91V89	PPP2R5D	protein phosphatase 2, regulatory	NA
P97470	PPP4C	protein phosphatase 4, catalytic su	NA
E9QPR5;Q8K2V1;E9	PPP4R1	protein phosphatase 4, regulatory	NA
Q0VGB7	PPP4R2	protein phosphatase 4, regulatory	NA
F7BX26;Q60676	PPP5C	protein phosphatase 5, catalytic su	NA
Q9CQR6	PPP6C	protein phosphatase 6, catalytic su	NA
Q7TSI3	PPP6R1	protein phosphatase 6, regulatory	NA
Q922D4	PPP6R3	protein phosphatase 6, regulatory	NA
P35700	PRDX1	peroxiredoxin 1	NA
Q61171	PRDX2	peroxiredoxin 2	NA
P20108	PRDX3	peroxiredoxin 3	NA
O08807	PRDX4	peroxiredoxin 4	NA
Q69ZK0	PREX1	phosphatidylinositol-3,4,5-trisphos	NA
P33610	PRIM2	DNA primase, p58 subunit	NA
P68181	PRKACB	protein kinase, cAMP dependent,	NA
O08795	PRKCSH	protein kinase C substrate 80K-H	NA
Q9WTX2	PRKRA	protein kinase, interferon inducible	NA
Q9JIF0	PRMT1	protein arginine N-methyltransfera	NA
Q8CIG8	PRMT5	protein arginine N-methyltransfera	NA
Q9Z2Y8	PROSC	proline synthetase co-transcribed	NA
Q99KP6	PRPF19	PRP19/PSO4 pre-mRNA process	NA
Q8CCF0	PRPF31	PRP31 pre-mRNA processing fac	NA
E9QJV4;F8WGV4	PRPF39	PRP39 pre-mRNA processing fac	NA
Q9DAW6	PRPF4	PRP4 pre-mRNA processing facto	NA
Q91YR7	PRPF6	PRP6 pre-mRNA splicing factor 6	NA
Q99PV0	PRPF8	pre-mRNA processing factor 8	NA
Q9D7G0	PRPS1	phosphoribosyl pyrophosphate syn	NA
G3UXL2	PRPS1L3	phosphoribosyl pyrophosphate syn	NA
Q9CS42	PRPS2	phosphoribosyl pyrophosphate syn	NA
Q9D0M1	PRPSAP1	phosphoribosyl pyrophosphate syn	NA
Q8R574	PRPSAP2	phosphoribosyl pyrophosphate syn	NA
Q7TSC1	PRRC2A	proline-rich coiled-coil 2A	NA
Q3TLH4;D3Z7M2;D3	PRRC2C	proline-rich coiled-coil 2C	NA
Q9Z1R9	PRSS1	protease, serine, 1 (trypsin 1)	257
Q9R1P4	PSMA1	proteasome (prosome, macropain	NA
P49722	PSMA2	proteasome (prosome, macropain	NA
O70435	PSMA3	proteasome (prosome, macropain	NA
Q9R1P0	PSMA4	proteasome (prosome, macropain	NA
Q9Z2U1	PSMA5	proteasome (prosome, macropain	NA
Q9QUM9	PSMA6	proteasome (prosome, macropain	NA
Q9Z2U0	PSMA7	proteasome (prosome, macropain	NA
O09061	PSMB1	proteasome (prosome, macropain	NA
Q9R1P3	PSMB2	proteasome (prosome, macropain	NA
Q9R1P1	PSMB3	proteasome (prosome, macropain	NA
P99026	PSMB4	proteasome (prosome, macropain	NA
O55234	PSMB5	proteasome (prosome, macropain	NA
Q60692	PSMB6	proteasome (prosome, macropain	NA

P70195	PSMB7	proteasome (prosome, macropain	NA
P62192	PSMC1	protease (prosome, macropain) 2(	NA
P46471	PSMC2	proteasome (prosome, macropain	NA
O88685	PSMC3	proteasome (prosome, macropain	NA
P54775	PSMC4	proteasome (prosome, macropain	NA
P62196	PSMC5	protease (prosome, macropain) 2(	NA
P62334	PSMC6	proteasome (prosome, macropain	NA
Q3TXS7	PSMD1	proteasome (prosome, macropain	NA
Q9Z2X2	PSMD10	proteasome (prosome, macropain	NA
Q8BG32	PSMD11	proteasome (prosome, macropain	NA
Q9D8W5	PSMD12	proteasome (prosome, macropain	NA
Q9WVJ2	PSMD13	proteasome (prosome, macropain	NA
O35593	PSMD14	proteasome (prosome, macropain	NA
Q8VDM4	PSMD2	proteasome (prosome, macropain	NA
P14685	PSMD3	proteasome (prosome, macropain	NA
O35226	PSMD4	proteasome (prosome, macropain	NA
Q8BJY1	PSMD5	proteasome (prosome, macropain	NA
Q99JI4	PSMD6	proteasome (prosome, macropain	NA
P26516	PSMD7	proteasome (prosome, macropain	NA
Q9CX56	PSMD8	proteasome (prosome, macropain	NA
Q9CR00	PSMD9	proteasome (prosome, macropain	NA
G3UXZ5;P97371	PSME1	proteasome (prosome, macropain	NA
P61290	PSME3	proteaseome (prosome, macropai	NA
Q5SSW2	PSME4	proteasome (prosome, macropain	NA
Q9JK23	PSMG1	proteasome (prosome, macropain	NA
Q8CB58;Q8BGJ5;Q(	PTBP1	polypyrimidine tract binding protei	NA
Q14C51	PTCD3	pentatricopeptide repeat domain 3	NA
Q9R0Q7	PTGES3	prostaglandin E synthase 3 (cytos	NA
P34152	PTK2	PTK2 protein tyrosine kinase 2	NA
P26350	PTMA	prothymosin alpha	NA
B9EKR1	PTPRZ1	protein tyrosine phosphatase, rece	NA
Q3UEB3	PUF60	poly-U binding splicing factor 60	NA
Q80U78	PUM1	pumilio 1 (Drosophila)	NA
Q3TQ29;Q80U58;Q3	PUM2	pumilio 2 (Drosophila)	NA
P42669	PURA	purine rich element binding protei	NA
O35295	PURB	purine rich element binding protei	NA
Q91VU7;B7ZNL8;F7	PUS7	pseudouridylate synthase 7 homol	NA
Q922W5	PYCR1	pyrroline-5-carboxylate reductase	NA
Q922Q4	PYCR2	pyrroline-5-carboxylate reductase	NA
D3Z158;Q8BML9;Q8	QARS	glutaminyl-tRNA synthetase	NA
Q9QYS9	QK	quaking	NA
Q5SW88	RAB1	RAB1, member RAS oncogene fa	NA
P61027	RAB10	RAB10, member RAS oncogene f	NA
P62492	RAB11A	RAB11a, member RAS oncogene	NA
P46638	RAB11B	RAB11B, member RAS oncogene	NA
A2CG35;P35283	RAB12	RAB12, member RAS oncogene f	NA
Q91V41	RAB14	RAB14, member RAS oncogene f	NA
P35293	RAB18	RAB18, member RAS oncogene f	NA
Q9D1G1	RAB1B	RAB1B, member RAS oncogene f	NA
P53994	RAB2A	RAB2A, member RAS oncogene f	NA
Q921E2	RAB31	RAB31, member RAS oncogene f	NA
Q6PHN9	RAB35	RAB35, member RAS oncogene f	NA
Q9CZT8	RAB3B	RAB3B, member RAS oncogene f	NA
E9QKE4;Q8BMG7	RAB3GAP2	RAB3 GTPase activating protein s	NA
Q9CQD1	RAB5A	RAB5A, member RAS oncogene f	NA

P61021	RAB5B	RAB5B, member RAS oncogene f	NA
P35278	RAB5C	RAB5C, member RAS oncogene f	NA
P35279	RAB6A	RAB6A, member RAS oncogene f	NA
P51150	RAB7	RAB7, member RAS oncogene fa	NA
P55258	RAB8A	RAB8A, member RAS oncogene f	NA
P61028	RAB8B	RAB8B, member RAS oncogene f	NA
A2AWA9	RABGAP1	RAB GTPase activating protein 1	NA
P63001	RAC1	RAS-related C3 botulinum substra	NA
Q8C570	RAE1	RAE1 RNA export 1 homolog (S. p	NA
Q64012	RALY	hnRNP-associated with lethal yello	NA
P62827	RAN	RAN, member RAS oncogene fam	NA
Q9ERU9	RANBP2	RAN binding protein 2	NA
P46061	RANGAP1	RAN GTPase activating protein 1	NA
P62835	RAP1A	RAS-related protein-1a	NA
Q99JI6	RAP1B	RAS related protein 1b	NA
Q9D0I9	RARS	arginyl-tRNA synthetase	NA
Q3U186	RARS2	arginyl-tRNA synthetase 2, mitoch	NA
Q61820	RASL2-9	RAS-like, family 2, locus 9	NA
Q60972	RBBP4	retinoblastoma binding protein 4	NA
Q60973	RBBP7	retinoblastoma binding protein 7	NA
Q8C2Q3	RBM14	RNA binding motif protein 14	NA
O89086	RBM3	RNA binding motif protein 3	NA
Q9CQT2	RBM7	RNA binding motif protein 7	NA
Q9WV02	RBMX	RNA binding motif protein, X chro	NA
Q91VM5	RBMXL1	RNA binding motif protein, X linke	NA
Q8VE37	RCC1	regulator of chromosome condens	NA
Q05186	RCN1	reticulocalbin 1	NA
Q8BP92	RCN2	reticulocalbin 2	NA
Q9QYF1	RDH11	retinol dehydrogenase 11	NA
P26043	RDX	radixin	NA
Q04207	RELA	v-rel reticuloendotheliosis viral onc	NA
Q9WUK4	RFC2	replication factor C (activator 1) 2	NA
Q8R323	RFC3	replication factor C (activator 1) 3	NA
Q99J62	RFC4	replication factor C (activator 1) 4	NA
Q9D0F6	RFC5	replication factor C (activator 1) 5	NA
Q9QUI0	RHOA	ras homolog gene family, member	NA
Q91VI7	RNH1	ribonuclease/angiogenin inhibitor 1	NA
Q9D0L8	RNMT	RNA (guanine-7-) methyltransfera	NA
Q8VEE4	RPA1	replication protein A1	NA
Q3TE40;Q62193	RPA2	replication protein A2	NA
Q9CQ71	RPA3	replication protein A3	NA
Q80TE0	RPAP1	RNA polymerase II associated pro	NA
Q5XJF6;D6RE43;P5	RPL10A	ribosomal protein L10A	NA
Q6ZWV3	RPL10-PS3	ribosomal protein L10, pseudogen	NA
Q9CXW4	RPL11	ribosomal protein L11	NA
P47963	RPL13	ribosomal protein L13	NA
P19253	RPL13A	ribosomal protein L13A	NA
Q9CR57	RPL14	ribosomal protein L14	NA
Q9CZM2	RPL15	ribosomal protein L15	NA
Q9CPR4;Q6ZWZ7	RPL17	ribosomal protein L17	NA
P35980	RPL18	ribosomal protein L18	NA
P62717	RPL18A	ribosomal protein L18A	NA
A2A547;P84099	RPL19	ribosomal protein L19	NA
Q9CQM8;O09167	RPL21	ribosomal protein L21	NA
P67984	RPL22	ribosomal protein L22	NA

Q9D7S7	RPL22L1	ribosomal protein L22 like 1	NA
P62830	RPL23	ribosomal protein L23	NA
D3YWP3	RPL23A-PS3	ribosomal protein L23A, pseudoge	NA
Q8BP67;E9QNJ0;E9	RPL24	ribosomal protein L24	NA
P61255	RPL26	ribosomal protein L26	NA
P61358	RPL27	ribosomal protein L27	NA
P41105	RPL28	ribosomal protein L28	NA
P47915	RPL29	ribosomal protein L29	NA
P27659	RPL3	ribosomal protein L3	NA
P62889	RPL30	ribosomal protein L30	NA
P62900	RPL31	ribosomal protein L31	NA
P62911	RPL32	ribosomal protein L32	NA
Q9D1R9	RPL34	ribosomal protein L34	NA
O55142	RPL35A	ribosomal protein L35A	NA
Q6ZWZ4	RPL36	ribosomal protein L36	NA
P83882	RPL36AL	ribosomal protein L36A-like	NA
D3YW41	RPL36-PS3	ribosomal protein L36, pseudogen	NA
P61514	RPL37A	ribosomal protein L37a	NA
Q9JJI8	RPL38	ribosomal protein L38	NA
Q9D8E6	RPL4	ribosomal protein L4	NA
P47962	RPL5	ribosomal protein L5	NA
P47911	RPL6	ribosomal protein L6	NA
P14148	RPL7	ribosomal protein L7	NA
P12970	RPL7A	ribosomal protein L7A	NA
D3YVE6	RPL7A-PS10	ribosomal protein L7A, pseudoger	NA
P62918	RPL8	ribosomal protein L8	7
P51410	RPL9	ribosomal protein L9	NA
P14869	RPLP0	ribosomal protein, large, P0	NA
P47955	RPLP1	ribosomal protein, large, P1	NA
P99027	RPLP2	ribosomal protein, large P2	NA
Q91YQ5	RPN1	ribophorin I	NA
Q9DBG6	RPN2	ribophorin II	NA
O88796	RPP30	ribonuclease P/MRP 30 subunit	NA
P63325	RPS10	ribosomal protein S10	NA
P62281	RPS11	ribosomal protein S11	NA
Q6ZWZ6	RPS12	ribosomal protein S12	NA
P62301	RPS13	ribosomal protein S13	NA
P62264	RPS14	ribosomal protein S14	NA
P62843	RPS15	ribosomal protein S15	NA
P62245	RPS15A	ribosomal protein S15A	NA
P14131	RPS16	ribosomal protein S16	NA
P63276	RPS17	ribosomal protein S17	NA
P62270	RPS18	ribosomal protein S18	NA
Q9CZX8	RPS19	ribosomal protein S19	NA
P25444	RPS2	ribosomal protein S2	NA
P60867	RPS20	ribosomal protein S20	NA
Q9CQR2	RPS21	ribosomal protein S21	NA
P62267	RPS23	ribosomal protein S23	NA
P62849	RPS24	ribosomal protein S24	NA
P62852	RPS25	ribosomal protein S25	NA
P62855	RPS26	ribosomal protein S26	NA
P62983	RPS27A	ribosomal protein S27A	NA
Q6ZWY3	RPS27L	ribosomal protein S27-like	NA
P62858	RPS28	ribosomal protein S28	NA
P62274	RPS29	ribosomal protein S29	NA

P62908	RPS3	ribosomal protein S3	NA
P97351	RPS3A1	ribosomal protein S3A1	NA
P62702	RPS4X	ribosomal protein S4, X-linked	NA
Q91V55;P97461;D3Y	RPS5	ribosomal protein S5	NA
P62754	RPS6	ribosomal protein S6	NA
D3YX41	RPS6-PS2	40S ribosomal protein S6	NA
P62082	RPS7	ribosomal protein S7	NA
P62242	RPS8	ribosomal protein S8	NA
Q6ZWN5	RPS9	ribosomal protein S9	NA
P14206	RPSA	ribosomal protein SA	NA
D3YTT7	RPSA-PS10	ribosomal protein SA, pseudogene	NA
Q9JKY0	RQCD1	rcd1 (required for cell differentiatic	NA
Q80X95	RRAGA	Ras-related GTP binding A	NA
Q99K70	RRAGC	Ras-related GTP binding C	NA
Q99PL5;A2AVJ7	RRBP1	ribosome binding protein 1	NA
P07742	RRM1	ribonucleotide reductase M1	NA
E9Q0A1;Q8BVY0;Q3	RSL1D1	ribosomal L1 domain containing 1	NA
Q01730;A2AUR7;Q9	RSU1	Ras suppressor protein 1	NA
Q99LF4	RTCB	RNA 2',3'-cyclic phosphate and 5'-	NA
Q8K0T0	RTN1	reticulon 1	NA
Q9ES97	RTN3	reticulon 3	NA
Q99P72	RTN4	reticulon 4	NA
P60122	RUVBL1	RuvB-like protein 1	NA
Q9WTM5	RUVBL2	RuvB-like protein 2	NA
Q9R1T2	SAE1	SUMO1 activating enzyme subuni	NA
E9PZM6;D3YXK2	SAFB	scaffold attachment factor B	NA
F6TC77;Q80YR5;F6	SAFB2	scaffold attachment factor B2	NA
Q99JZ4	SAR1A	SAR1 gene homolog A (S. cerevis	NA
Q9CQC9	SAR1B	SAR1 gene homolog B (S. cerevis	NA
P26638	SARS	seryl-aminoacyl-tRNA synthetase	NA
Q9JLI8	SART3	squamous cell carcinoma antigen	NA
P70122	SBDS	Shwachman-Bodian-Diamond syn	NA
P13011	SCD2	stearoyl-Coenzyme A desaturase	NA
Q8BRF7	SCFD1	Sec1 family domain containing 1	NA
Q80U72;Q6P9N3	SCRIB	scribbled homolog (Drosophila)	NA
Q8K2B3	SDHA	succinate dehydrogenase comple:	NA
Q9CQA3	SDHB	succinate dehydrogenase comple:	NA
Q9D1M0	SEC13	SEC13 homolog (S. cerevisiae)	NA
A2AIX1;E9QAT4;F7E	SEC16A	SEC16 homolog A (S. cerevisiae)	NA
O08547	SEC22B	SEC22 vesicle trafficking protein h	NA
Q01405	SEC23A	SEC23A (S. cerevisiae)	NA
Q9D662	SEC23B	SEC23B (S. cerevisiae)	NA
F6VJC5;Q3U882;Q8	SEC24B	Sec24 related gene family, memb	NA
G3X972;Q80U83	SEC24C	Sec24 related gene family, memb	NA
Q3UPL0	SEC31A	Sec31 homolog A (S. cerevisiae)	NA
Q9CQS8	SEC61B	Sec61 beta subunit	NA
Q9ERR7	SEP15	selenoprotein	NA
Q8C650	SEPT10	septin 10	NA
Q8C1B7	SEPT11	septin 11	NA
P42208	SEPT2	septin 2	NA
E9Q9F5;O55131;E9	SEPT7	septin 7	NA
Q80UG5	SEPT9	septin 9	NA
Q9CY58	SERBP1	serpine1 mRNA binding protein 1	NA
Q07235	SERPINE2	serine (or cysteine) peptidase inhi	NA
A2BE93;Q9EQU5	SET	SET nuclear oncogene	NA

Q91WC0	SETD3	SET domain containing 3	NA
Q8K4Z5	SF3A1	splicing factor 3a, subunit 1	NA
Q9D554	SF3A3	splicing factor 3a, subunit 3	NA
Q99NB9	SF3B1	splicing factor 3b, subunit 1	NA
Q3UJB0	SF3B2	splicing factor 3b, subunit 2	NA
Q921M3	SF3B3	splicing factor 3b, subunit 3	NA
Q8VIJ6	SFPQ	splicing factor proline/glutamine ri	NA
Q99JR1	SFXN1	sideroflexin 1	NA
Q8R0X7	SGPL1	sphingosine phosphate lyase 1	NA
Q9CZN7	SHMT2	serine hydroxymethyltransferase 2	NA
Q9CZU3	SKIV2L2	superkiller viralicidic activity 2-like	NA
NA	SKP1A	S-phase kinase-associated protei	NA
Q9CR62	SLC25A11	solute carrier family 25 (mitochonc	NA
Q8VEM8	SLC25A3	solute carrier family 25 (mitochonc	NA
P48962	SLC25A4	solute carrier family 25 (mitochonc	NA
P51881	SLC25A5	solute carrier family 25 (mitochonc	NA
Q9CU62	SMC1A	structural maintenance of chromos	NA
Q8CG48	SMC2	structural maintenance of chromos	NA
Q9CW03	SMC3	structural maintenance of chromos	NA
Q8CG47	SMC4	structural maintenance of chromos	NA
Q6P5D8	SMCHD1	SMC hinge domain containing 1	NA
E9Q481;Q6P2K6	SMEK1	SMEK homolog 1, suppressor of r	NA
Q922R5	SMEK2	SMEK homolog 2, suppressor of r	NA
Q8BGT7	SMNDC1	survival motor neuron domain con	NA
Q78PY7	SND1	staphylococcal nuclease and tudo	NA
Q6P4T2	SNRNP200	small nuclear ribonucleoprotein 20	NA
Q6PE01	SNRNP40	small nuclear ribonucleoprotein 40	NA
Q62189	SNRPA	small nuclear ribonucleoprotein pc	NA
P57784	SNRPA1	small nuclear ribonucleoprotein pc	NA
P27048	SNRPB	small nuclear ribonucleoprotein B	NA
Q9CQI7	SNRPB2	U2 small nuclear ribonucleoprotei	NA
P62315	SNRPD1	small nuclear ribonucleoprotein D1	NA
P62317	SNRPD2	small nuclear ribonucleoprotein D2	NA
P62320	SNRPD3	small nuclear ribonucleoprotein D3	NA
P62305	SNRPE	small nuclear ribonucleoprotein E	NA
P62307	SNRPF	small nuclear ribonucleoprotein pc	NA
P63163	SNRPN	small nuclear ribonucleoprotein N	NA
Q8C788	SNX18	sorting nexin 18	NA
Q3UHD6	SNX27	sorting nexin family member 27	NA
O70492;Q78ZM0;D3	SNX3	sorting nexin 3	NA
Q91VH2	SNX9	sorting nexin 9	NA
Q3UMC0	SPATA5	spermatogenesis associated 5	NA
Q9CYN2	SPCS2	signal peptidase complex subunit	NA
A3KGU7;A3KGU5;E	SPNA2	Spectrin alpha 2	NA
Q68FG2	SPNB3	Spectrin beta 3	NA
P16546	SPTAN1	spectrin alpha, non-erythrocytic 1	NA
Q62261	SPTBN1	spectrin beta, non-erythrocytic 1	NA
Q64337	SQSTM1	sequestosome 1	NA
Q91Z67	SRGAP2	SLIT-ROBO Rho GTPase activati	NA
P16254	SRP14	signal recognition particle 14	NA
Q9D104;E9Q8C2	SRP19	signal recognition particle 19	NA
P14576	SRP54A	signal recognition particle 54A	NA
E9PXC0	SRP54C	signal recognition particle 54C	NA
Q8BMA6	SRP68	signal recognition particle 68	NA
E9Q740;F8VQC1	SRP72	signal recognition particle 72	NA

O54781	SRPK2	serine/arginine-rich protein specifi	NA
P47758	SRPRB	signal recognition particle receptor	NA
Q99MR6	SRRT	serrate RNA effector molecule hor	NA
Q6PDM2	SRSF1	serine/arginine-rich splicing factor	NA
Q62093	SRSF2	serine/arginine-rich splicing factor	NA
P84104	SRSF3	serine/arginine-rich splicing factor	NA
Q542V3;E9Q4U8;A2	SRSF4	serine/arginine-rich splicing factor	NA
O35326;E9QKW3;Q	SRSF5	serine/arginine-rich splicing factor	NA
Q3TWW8	SRSF6	serine/arginine-rich splicing factor	NA
Q8BL97	SRSF7	serine/arginine-rich splicing factor	NA
Q9D0B0	SRSF9	serine/arginine-rich splicing factor	NA
P32067	SSB	Sjogren syndrome antigen B	NA
Q9CYR0;Q8R2K3	SSBP1	single-stranded DNA binding prote	NA
Q9CY50	SSR1	signal sequence receptor, alpha	NA
Q62186	SSR4	signal sequence receptor, delta	NA
Q08943	SSRP1	structure specific recognition prote	NA
P56873	SSSCA1	Sjogren's syndrome/scleroderma a	NA
Q99L47	ST13	suppression of tumorigenicity 13	NA
Q60864	STIP1	stress-induced phosphoprotein 1	NA
Q99JB2	STOML2	stomatin (Epb7.2)-like 2	NA
Q9Z1Z2	STRAP	serine/threonine kinase receptor a	NA
Q9ERG2	STRN3	striatin, calmodulin binding protein	NA
P58404	STRN4	striatin, calmodulin binding protein	NA
Q9WUD1	STUB1	STIP1 homology and U-Box conta	NA
Q9Z2I9	SUCLA2	succinate-Coenzyme A ligase, AD	NA
Q9WUM5	SUCLG1	succinate-CoA ligase, GDP-formir	NA
G3X956;Q920B9	SUPT16H	Suppressor of Ty 16 homolog (S.	NA
O55201	SUPT5	suppressor of Ty 5	NA
Q62383	SUPT6	suppressor of Ty 6	NA
P09926	SURF2	surfeit gene 2	NA
Q7TMK9;G3UZI2;G3	SYNCRIP	synaptotagmin binding, cytoplasm	NA
Q8BQ46;E9Q6D4;F6	TAF15	TAF15 RNA polymerase II, TATA	NA
Q921F2	TARDBP	TAR DNA binding protein	NA
Q9D0R2	TARS	threonyl-tRNA synthetase	NA
Q8BLY2	TARSL2	threonyl-tRNA synthetase-like 2	NA
P48428	TBCA	tubulin cofactor A	NA
P10711	TCEA1	transcription elongation factor A (S	NA
P62869	TCEB2	transcription elongation factor B (S	NA
P11983	TCP1	t-complex protein 1	NA
Q9CY27	TECR	trans-2,3-enoyl-CoA reductase	NA
Q8C2C6;Q9Z1A1;Q9	TFG	Trk-fused gene	NA
Q62351	TFRC	transferrin receptor	NA
Q922L6;Q3TW27	TH1L	Negative elongation factor D	NA
Q569Z6	THRAP3	thyroid hormone receptor associat	NA
Q99J36	THUMPD1	THUMP domain containing 1	NA
P52912;D3Z4H6;D3Y	TIA1	cytotoxic granule-associated RNA	NA
P70318	TIAL1	Tia1 cytotoxic granule-associated	NA
Q8BH58	TIPRL	TIP41, TOR signalling pathway re	NA
P39447	TJP1	tight junction protein 1	NA
P40142	TKT	transketolase	NA
P26039	TLN1	talin 1	NA
Q9D1D4	TMED10	transmembrane emp24-like traffic	NA
Q9R0Q3	TMED2	transmembrane emp24 domain tra	NA
Q8R1V4	TMED4	transmembrane emp24 protein tra	NA
Q99KF1	TMED9	transmembrane emp24 protein tra	NA

Q99LQ7	TMEM189	transmembrane protein 189	NA
Q9DBS1	TMEM43	transmembrane protein 43	NA
Q9JHJ0	TMOD3	tropomodulin 3	NA
Q61029	TMPO	thymopoietin	NA
E9QPR6;Q5U405	TMPRSS13	transmembrane protease, serine 1	NA
P83510;B9EKN8;E0(	TNIK	TRAF2 and NCK interacting kinas	NA
Q8BFY9	TNPO1	transportin 1	NA
Q99LG2	TNPO2	transportin 2 (importin 3, karyophe	NA
Q6P2B1	TNPO3	transportin 3	NA
Q9CYG7	TOMM34	translocase of outer mitochondrial	NA
Q9CZW5	TOMM70A	translocase of outer mitochondrial	NA
Q04750	TOP1	topoisomerase (DNA) I	NA
Q64511	TOP2B	topoisomerase (DNA) II beta	NA
P70399	TP53BP1	Tumor suppressor p53-binding pr	NA
P17751	TPI1	triosephosphate isomerase 1	NA
B7ZNL3;E9Q455;E9(	TPM1	tropomyosin 1, alpha	NA
P21107	TPM3	tropomyosin 3, gamma	NA
Q6IRU2	TPM4	tropomyosin 4	NA
Q64514	TPP2	tripeptidyl peptidase II	NA
F6ZDS4	TPR	translocated promoter region	NA
P63028	TPT1	tumor protein, translationally-contr	NA
P62996	TRA2B	transformer 2 beta homolog (Dros	NA
Q9CQN1	TRAP1	TNF receptor-associated protein 1	NA
Q921I1	TRF	transferrin	NA
Q62318	TRIM28	tripartite motif-containing 28	NA
Q8CH72	TRIM32	tripartite motif-containing 32	6
E9PWD4;D3Z413;Q:	TRMT1	tRNA methyltransferase 1	NA
Q9D0C4	TRMT5	TRM5 tRNA methyltransferase 5	NA
Q8CE96	TRMT6	tRNA methyltransferase 6	NA
Q80XC2	TRMT61A	tRNA methyltransferase 61A	NA
Q8K1J6	TRNT1	tRNA nucleotidyl transferase, CC/	NA
O08848	TROVE2	TROVE domain family, member 2	NA
A2AU91;A2AU89;E9	TRP53BP1	transformation related protein 53 k	NA
Q4QQM4	TRP53I11	transformation related protein 53 i	NA
Q792Z1	TRY10	trypsin 10	93
Q8BMZ5	TSEN34	tRNA splicing endonuclease 34 hc	NA
Q62348	TSN	translin	NA
Q5SWD9	TSR1	TSR1 20S rRNA accumulation	NA
Q9D6K7	TTC33	tetratricopeptide repeat domain 33	NA
A2ASS6	TTN	titin	NA
P68369	TUBA1A	tubulin, alpha 1A	8
P68373	TUBA1C	tubulin, alpha 1C	8
P68368	TUBA4A	tubulin, alpha 4A	NA
Q3UX10	TUBAL3	tubulin, alpha-like 3	NA
A2AQ07	TUBB1	tubulin, beta 1 class VI	NA
Q7TMM9	TUBB2A	tubulin, beta 2A class IIA	4
Q9CWF2	TUBB2B	tubulin, beta 2B class IIB	4
Q9ERD7	TUBB3	tubulin, beta 3 class III	NA
Q9D6F9	TUBB4A	tubulin, beta 4A class IVA	NA
P68372	TUBB4B	tubulin, beta 4B class IVB	NA
P99024	TUBB5	tubulin, beta 5 class I	NA
Q922F4	TUBB6	tubulin, beta 6 class V	NA
P83887	TUBG1	tubulin, gamma 1	NA
Q8BFR5	TUFM	Tu translation elongation factor, m	NA
Q91YR1	TWF1	twinfilin, actin-binding protein, hon	NA

Q6PAM1	TXLNA	taxilin alpha	NA
P10639	TXN1	thioredoxin 1	NA
Q91W90	TXNDC5	thioredoxin domain containing 5	NA
Q8CDN6	TXNL1	thioredoxin-like 1	NA
Q9D883	U2AF1	U2 small nuclear ribonucleoprotei	NA
P26369	U2AF2	U2 small nuclear ribonucleoprotei	NA
Q02053	UBA1	ubiquitin-like modifier activating er	NA
Q9Z1F9	UBA2	ubiquitin-like modifier activating er	NA
P62984	UBA52	ubiquitin A-52 residue ribosomal p	NA
A2AMY5;Q91VX2;A2	UBAP2	ubiquitin-associated protein 2	NA
Q80X50	UBAP2L	ubiquitin associated protein 2-like	NA
P0CG49	UBB	ubiquitin B	NA
P0CG50	UBC	ubiquitin C	NA
P62257	UBE2H	ubiquitin-conjugating enzyme E2H	NA
P61087	UBE2K	ubiquitin-conjugating enzyme E2K	NA
P61082	UBE2M	ubiquitin-conjugating enzyme E2M	NA
P61089	UBE2N	ubiquitin-conjugating enzyme E2N	NA
Q6ZPJ3	UBE2O	ubiquitin-conjugating enzyme E2C	NA
Q6ZWZ2	UBE2R2	ubiquitin-conjugating enzyme E2R	NA
Q9CZY3	UBE2V1	ubiquitin-conjugating enzyme E2 v	NA
Q3UE37	UBE2Z	ubiquitin-conjugating enzyme E2Z	NA
Q5DTH1;E9QKT1	UBE3A	ubiquitin protein ligase E3A	NA
Q80U95	UBE3C	ubiquitin protein ligase E3C	NA
Q9ES00	UBE4B	ubiquitination factor E4B, UFD2 hc	NA
P21126	UBL4	ubiquitin-like 4	NA
A2AN08	UBR4	ubiquitin protein ligase E3 compor	NA
E9Q2H1;Q80TP3	UBR5	ubiquitin protein ligase E3 compor	NA
Q922Y1	UBXN1	UBX domain protein 1	NA
Q99PL6	UBXN6	UBX domain protein 6	NA
Q9WUP7	UCHL5	ubiquitin carboxyl-terminal esteras	NA
P70362	UFD1L	ubiquitin fusion degradation 1 like	NA
Q6P5E4	UGGT1	UDP-glucose glycoprotein glucosy	NA
Q91ZJ5	UGP2	UDP-glucose pyrophosphorylase 2	NA
P13439	UMPS	uridine monophosphate synthetas	NA
Q9EPU0	UPF1	UPF1 regulator of nonsense trans	NA
Q9DB77	UQCRC2	ubiquinol cytochrome c reductase	NA
Q9CR68	UQCRFS1	ubiquinol-cytochrome c reductase	NA
Q9Z1Z0	USO1	USO1 vesicle docking factor	NA
Q9JMA1	USP14	ubiquitin specific peptidase 14	NA
Q8R5H1	USP15	ubiquitin specific peptidase 15	NA
B1AY13	USP24	ubiquitin specific peptidase 24	NA
Q8BWR4	USP40	ubiquitin specific peptidase 40	NA
Q8BY87	USP47	ubiquitin specific peptidase 47	NA
P56399	USP5	ubiquitin specific peptidase 5 (isop	NA
E9QLK0;F8VPX1;E9	USP7	ubiquitin specific peptidase 7	NA
Q4FE56;P70398	USP9X	ubiquitin specific peptidase 9, X cl	NA
F8VPU6	USP9Y	ubiquitin specific peptidase 9, Y cl	NA
Q80WQ2	VAC14	Vac14 homolog (S. cerevisiae)	NA
Q9WV55	VAPA	vesicle-associated membrane pro	NA
Q8BH80;Q9QY76	VAPB	vesicle-associated membrane pro	NA
Q9Z1Q9	VARS	valyl-tRNA synthetase	NA
P61759	VBP1	von Hippel-Lindau binding protein	NA
Q64727	VCL	vinculin	NA
Q01853	VCP	valosin containing protein	NA
Q8CDG3	VCPIP1	valosin containing protein (p97)/p4	NA

Q60932	VDAC1	voltage-dependent anion channel	NA
Q60930	VDAC2	voltage-dependent anion channel	NA
Q60931	VDAC3	voltage-dependent anion channel	NA
E9PZV5;P20152	VIM	vimentin	NA
E9Q6I0	VMN2R116	vomeronasal 2, receptor 116	NA
Q80TR8	VPRBP	Vpr (HIV-1) binding protein	NA
Q9QZ88	VPS29	vacuolar protein sorting 29 (S. por	NA
Q9EQH3	VPS35	vacuolar protein sorting 35	NA
Q9CY21	WBSCR22	Williams Beuren syndrome chrom	NA
O88342	WDR1	WD repeat domain 1	NA
Q8K1X1	WDR11	WD repeat domain 11	NA
Q8C6G8	WDR26	WD repeat domain 26	NA
Q99ME2	WDR6	WD repeat domain 6	NA
Q8BVQ0;Q9ERF3;F(	WDR61	WD repeat domain 61	NA
Q99J09	WDR77	WD repeat domain 77	NA
Q8BFQ4	WDR82	WD repeat domain containing 82	NA
Q8CHP5	WIBG	within bgcn homolog (Drosophila)	NA
Q6P5F9	XPO1	exportin 1, CRM1 homolog (yeast)	NA
Q924C1	XPO5	exportin 5	NA
P27641	XRCC5	X-ray repair complementing defec	NA
E9Q0B8;P23475	XRCC6	X-ray repair complementing defec	NA
Q9DBR1	XRN2	5'-3' exoribonuclease 2	NA
A2A7S7;Q91WQ3	YARS	tyrosyl-tRNA synthetase	NA
P62960	YBX1	Y box protein 1	NA
Q9JKB3	YBX3	Y box protein 3	NA
Q9CQW1	YKT6	YKT6 homolog (S. Cerevisiae)	NA
P59326	YTHDF1	YTH domain family 1	NA
E9Q2W5;Q91YT7	YTHDF2	YTH domain family 2	NA
Q8BYK6	YTHDF3	YTH domain family 3	NA
Q9CQV8	YWHAB	tyrosine 3-monooxygenase/tryptop	NA
P62259	YWHAE	tyrosine 3-monooxygenase/tryptop	NA
P61982	YWHAG	tyrosine 3-monooxygenase/tryptop	NA
P68510	YWHAH	tyrosine 3-monooxygenase/tryptop	NA
F6VW30;F6YY69;P6	YWHAQ	tyrosine 3-monooxygenase/tryptop	NA
P63101	YWHAZ	tyrosine 3-monooxygenase/tryptop	NA
Q3TIV5	ZC3H15	zinc finger CCCH-type containing	NA
Q64318	ZEB1	zinc finger E-box binding homeob(	NA
O88532	ZFR	zinc finger RNA binding protein	NA

Trim32 enriched Proteins

control

Spectral count differentiating NSC	Spectral count proliferating NSC	Poisson p value (NSCs vs control)	Robust z-test p value (differentiating NSC vs proliferating NSC)
5	8	0.016	0.826
9	11	0.001	0.815
3	4	0.063	0.652
10	25	3.814697265625e-06	0.124
10	5	0.004	0.169
9	12	0.001	0.924
7	10	0.004	0.989
NA	7	0.063	NA
30	35	2.32830643653869e-10	0.856
8	18	0.000	0.460
5	17	0.000	0.163
26	17	2.38418579101562e-07	0.276
35	49	2.27373675443232e-13	0.540
8	NA	0.063	NA
6	1	0.063	0.018
15	6	0.001	0.044
10	2	0.016	0.025
15.5	5	0.001	0.018
23	36	9.31322574615481e-10	0.534
4	3	0.063	0.741
25	35	9.31322574615481e-10	0.687
9	2	0.016	0.013
37.5	19	3.72529029846191e-09	0.112
6	6	0.016	0.671
44	20	2.32830643653869e-10	0.071
NA	208	4.93038065763134e-32	NA
235	30	4.37623562951089e-20	2.57093004621499e-07
68	117	2.01948391736578e-28	0.317
140	20	8.2718061255303e-25	6.33930123154589e-05
235	407	1.20084874304891e-68	0.180
70.5	92	4.13590306276515e-25	0.772
146	122	4.59177480789961e-41	0.304
23	25	5.96046447753907e-08	0.926
20	23	2.38418579101562e-07	0.986
16	20	3.814697265625e-06	0.856
13	10	0.000	0.331
9	7	0.004	0.442
4	3	0.063	0.741
8	5	0.016	0.285
NA	16	0.004	NA
17	17	7.62939453125e-06	0.797
21	14	3.814697265625e-06	0.293
28	32	9.31322574615481e-10	0.996
19	20	9.53674316406249e-07	0.876
3	5	0.063	0.451
10	NA	0.031	NA
5	2	0.063	0.237
6	9	0.004	0.895
14	11	0.000	0.348

18	12	3.0517578125e-05	0.230
20.5	13	7.62939453125e-06	0.250
9	25	7.62939453125e-06	0.081
14	14	6.103515625e-05	0.579
47	28	3.63797880709171e-12	0.159
6	3	0.063	0.169
11	4	0.004	0.070
18	12	3.0517578125e-05	0.230
36	26	4.65661287307738e-10	0.371
24	30	7.45058059692382e-09	0.856
9	9	0.002	0.579
2	6	0.063	0.121
13.75	40	7.45058059692382e-09	0.066
31	30	9.31322574615481e-10	0.748
29	19	5.96046447753907e-08	0.278
6	11	0.004	0.681
13	19	1.52587890625e-05	0.960
9	14	0.000	0.881
11	14	0.000	0.865
17	30	5.96046447753907e-08	0.391
9	6	0.004	0.327
15	7.5	0.000	0.097
26.5	12.5	9.53674316406249e-07	0.084
7	12	0.001	0.761
16	23	9.53674316406249e-07	0.649
20	15	3.814697265625e-06	0.412
20	15	3.814697265625e-06	0.412
18	12	3.0517578125e-05	0.230
14	11	0.000	0.348
7	6	0.016	0.525
4	9	0.016	0.491
18	44	4.65661287307738e-10	0.135
12.5	9	0.000	0.281
21	22	2.38418579101562e-07	0.869
14	7	0.001	0.097
6	9	0.004	0.895
2	5	0.063	0.192
4	3	0.063	0.741
5	9	0.008	0.702
NA	7	0.063	NA
9	12	0.001	0.924
NA	27	6.103515625e-05	NA
57	30	5.68434188608079e-14	0.089
57	72	5.42101086242751e-20	0.836
2	11	0.016	0.116
2	11	0.016	0.116
NA	15	0.004	NA
8	18	0.000	0.460
4	5	0.063	0.906
10	4	0.008	0.093
14	17	1.52587890625e-05	0.806
NA	7	0.063	NA
6	22	6.103515625e-05	0.129
5	8	0.016	0.826
7	5	0.016	0.376

10	17	6.103515625e-05	0.771
7	9	0.004	0.937
10	NA	0.031	NA
7	24	1.52587890625e-05	0.159
10	NA	0.031	NA
4	3	0.063	0.741
14	40	7.45058059692382e-09	0.072
6	3	0.063	0.169
3	4	0.063	0.652
13	18	1.52587890625e-05	0.971
15	22	3.814697265625e-06	0.621
1	7	0.063	0.038
10	9	0.001	0.470
4	9	0.016	0.491
10	15	0.000	0.927
6	10	0.004	0.782
12	19	1.52587890625e-05	0.859
48.2	194	3.76158192263134e-37	0.022
2	32	7.62939453125e-06	7.59575965706505e-05
13	24	3.814697265625e-06	0.344
45	44	5.68434188608079e-14	0.813
26.5	28	7.45058059692382e-09	0.882
4.7	13	0.002	0.286
47	39	1.13686837721616e-13	0.533
8	16	0.000	0.582
20	30	2.98023223876953e-08	0.590
6	4	0.031	0.327
29	20	5.96046447753907e-08	0.324
8	14	0.000	0.736
39	27	1.16415321826935e-10	0.293
6	11	0.004	0.681
10	9	0.001	0.470
22	23	2.38418579101562e-07	0.865
14	13	6.103515625e-05	0.501
11	3	0.008	0.027
3	7	0.031	0.461
7	1	0.063	0.028
70	45	3.46944695195362e-18	0.220
9	14	0.000	0.881
43	101	2.11758236813575e-22	0.072
7	4	0.016	0.233
9	5	0.008	0.219
NA	7	0.063	NA
73	88	8.2718061255303e-25	0.931
30	56	1.13686837721616e-13	0.196
64	56	8.67361737988403e-19	0.618
65	67	1.35525271560689e-20	0.752
46	45	1.4210854715202e-14	0.814
56	52	5.55111512312579e-17	0.720
73	66	8.47032947254304e-22	0.514
17	24	9.53674316406249e-07	0.675
18	12	3.0517578125e-05	0.230
20	8	6.103515625e-05	0.044
7	6	0.016	0.525
4	6	0.031	0.895

4	4	0.063	0.952
4	17	0.001	0.080
NA	8	0.063	NA
8	22	3.0517578125e-05	0.290
4	7	0.016	0.731
11	13	0.000	0.773
41	33	7.27595761418345e-12	0.486
8	8	0.004	0.671
3	4	0.063	0.652
13	6	0.001	0.074
1	20	0.001	0.001
NA	13	0.016	NA
223	312	2.10843958864611e-81	0.125
NA	7	0.063	NA
NA	7	0.063	NA
4	10	0.008	0.405
6	4	0.031	0.327
6	2	0.063	0.152
7	6	0.016	0.525
243	248	8.84343660041662e-75	0.973
9	15	0.000	0.795
4	10.5	0.008	0.368
21	41	4.65661287307738e-10	0.291
8	20	6.103515625e-05	0.365
10	NA	0.031	NA
NA	11	0.016	NA
3	4	0.063	0.652
27	80	5.55111512312579e-17	0.017
20	42	4.65661287307738e-10	0.231
15	31	1.19209289550781e-07	0.243
10	21	1.52587890625e-05	0.530
16	37	1.49011611938477e-08	0.165
16	37	1.49011611938477e-08	0.165
3	7	0.031	0.461
3	4	0.063	0.652
11	10	0.001	0.480
10	9	0.001	0.470
10	13	0.000	0.892
8	14	0.000	0.736
17	14	1.52587890625e-05	0.388
7	11	0.002	0.868
8	11	0.001	0.963
3	6	0.063	0.599
38	20	1.86264514923096e-09	0.130
4	3	0.063	0.741
5	6	0.016	0.862
99	48	5.29395592033942e-23	0.024
20	30	2.98023223876953e-08	0.590
34	23	3.72529029846191e-09	0.306
5	10	0.004	0.599
26	32	1.86264514923096e-09	0.880
9	11	0.001	0.815
19	19	1.9073486328125e-06	0.797
5	8	0.016	0.826
4	6	0.031	0.895

NA	21	0.001	NA
9	9	0.002	0.579
11	6	0.004	0.128
36	28	2.32830643653869e-10	0.454
5	13	0.002	0.333
6	5	0.016	0.500
10	5	0.004	0.169
7	5	0.016	0.376
6	4	0.031	0.327
2	10	0.016	0.075
7	23	3.0517578125e-05	0.180
NA	9	0.063	NA
8	10	0.002	0.842
5.5	12	0.002	0.491
NA	13	0.016	NA
6	6	0.016	0.671
69	64	1.35525271560689e-20	0.558
11	9	0.001	0.382
7	7	0.008	0.671
25	12	3.814697265625e-06	0.090
9	5	0.008	0.219
2	12	0.008	0.095
9.8	33	4.76837158203125e-07	0.034
17	15	1.52587890625e-05	0.451
4	4	0.063	0.952
70	55	2.168404344971e-19	0.452
1	19	0.001	0.002
23	38	9.31322574615481e-10	0.467
61	41	4.44089209850065e-16	0.262
7	5	0.016	0.376
19	5	0.000	0.030
28	NA	6.103515625e-05	NA
48	26	7.27595761418345e-12	0.103
81	93	6.46234853557053e-27	0.969
60	67	5.42101086242751e-20	0.938
12	23	3.814697265625e-06	0.308
9	5	0.008	0.219
8	5	0.016	0.285
15	6	0.001	0.044
8	10	0.002	0.842
1	9	0.031	0.033
2	10	0.016	0.075
7	1	0.063	0.028
111	41	1.32348898008484e-23	0.003
2	8	0.031	0.140
6	3	0.063	0.169
3	6	0.063	0.599
4	3	0.063	0.741
12.5	10	0.000	0.363
14	12	0.000	0.424
15	25	9.53674316406249e-07	0.456
16	20	3.814697265625e-06	0.856
NA	7	0.063	NA
6	12	0.002	0.582
6	3	0.063	0.169

11	15	0.000	0.952
4.333333333	6	0.031	0.982
8	6	0.008	0.413
NA	7	0.063	NA
3	17	0.001	0.027
13	NA	0.016	NA
5	11	0.004	0.511
11	16	6.103515625e-05	0.966
4	8.666666667	0.016	0.525
6	5	0.016	0.500
4	3	0.063	0.741
127	172	7.00649232162415e-46	0.170
10	8	0.002	0.363
2	5	0.063	0.192
5	3	0.063	0.527
7	7	0.008	0.671
5	4	0.063	0.465
8	4	0.016	0.169
31	7.5	1.9073486328125e-06	0.018
10	5	0.004	0.169
3	7	0.031	0.461
2	6	0.063	0.121
295	449	1.81130290033182e-84	0.309
2.333333333	18	0.001	0.043
6.5	11.875	0.002	0.685
11	11	0.000	0.579
26	21	5.96046447753907e-08	0.499
71	61	1.35525271560689e-20	0.434
7	NA	0.063	NA
44	29	1.45519152283668e-11	0.243
6	1	0.063	0.018
5	5	0.031	0.671
17	13	3.0517578125e-05	0.326
16	13	6.103515625e-05	0.376
19	19	1.9073486328125e-06	0.797
10	13	0.000	0.892
4	9	0.016	0.491
6	10	0.004	0.782
46	61	5.55111512312579e-17	0.628
14	20	7.62939453125e-06	0.658
29	33	4.65661287307738e-10	0.997
134	138	1.14794370197489e-41	0.991
63	65	5.42101086242751e-20	0.913
44	48	1.4210854715202e-14	0.982
34	27	9.31322574615481e-10	0.479
33	40	1.45519152283668e-11	0.786
26	26	1.49011611938477e-08	0.797
37	31	5.82076609134675e-11	0.548
38	45	2.27373675443232e-13	0.829
37	41	1.81898940354586e-12	0.952
14	16	3.0517578125e-05	0.733
14	16	3.0517578125e-05	0.733
11	14	0.000	0.865
51	44	3.5527136788005e-15	0.595
20	24	2.38418579101562e-07	0.920

79	71	2.6469779601697e-23	0.504
32	34	1.16415321826935e-10	0.968
26	24	2.98023223876953e-08	0.679
31	36	5.82076609134675e-11	0.865
7	17	0.000	0.390
32	72	2.22044604925033e-16	0.083
22	28	2.98023223876953e-08	0.829
2	9	0.016	0.101
7	18	0.000	0.342
12	18	3.0517578125e-05	0.927
32	61	1.4210854715202e-14	0.180
4	19	0.000	0.054
7	12	0.001	0.761
9	8	0.004	0.458
40	45	2.27373675443232e-13	0.924
8	2	0.031	0.020
16	12	6.103515625e-05	0.311
6	4	0.031	0.327
4	6	0.031	0.895
1	6	0.063	0.057
NA	9	0.063	NA
2	6	0.063	0.121
NA	13	0.016	NA
6	8	0.008	0.976
5	9	0.008	0.702
11	25	3.814697265625e-06	0.176
119	136	2.93873587705572e-39	0.960
12	4	0.004	0.053
4	5	0.063	0.906
1	8	0.063	0.045
1	18	0.001	0.002
22	28	2.98023223876953e-08	0.829
12	2	0.008	0.014
22	30	1.49011611938477e-08	0.725
7	3	0.031	0.113
11	9	0.001	0.382
5	6	0.016	0.862
5	7	0.016	0.970
5	5	0.031	0.671
9	5	0.008	0.219
13	7	0.001	0.123
4	5	0.063	0.906
31	46	3.63797880709171e-12	0.453
8	7	0.004	0.544
5	5	0.031	0.671
7	5	0.016	0.376
13	21	7.62939453125e-06	0.494
15	29	2.38418579101562e-07	0.300
63	37	8.88178419700125e-16	0.150
17	7	0.000	0.049
8	20	6.103515625e-05	0.365
8	NA	0.063	NA
5	8	0.016	0.826
4	4	0.063	0.952
48	37	2.27373675443232e-13	0.425

55	49	2.22044604925033e-16	0.648
17	14	1.52587890625e-05	0.388
12	25	3.814697265625e-06	0.237
181.6666667	228	1.94469227433166e-62	0.320
58	58	3.46944695195362e-18	0.855
2	8	0.031	0.140
4	11	0.004	0.335
NA	10	0.031	NA
NA	11	0.016	NA
8	8	0.004	0.671
45	21.5	1.16415321826935e-10	0.054
20	34	7.45058059692382e-09	0.433
47.33333333	65.66666667	1.38777878078145e-17	0.555
3	18	0.001	0.021
NA	11	0.016	NA
59	57	3.46944695195362e-18	0.791
10	19	6.103515625e-05	0.639
NA	7	0.063	NA
140	115	2.93873587705572e-39	0.369
89	104	1.53102124485295e-26	0.995
5	5	0.031	0.671
7	2	0.063	0.032
13	4	0.004	0.015
14	16	3.0517578125e-05	0.733
14	71	2.27373675443232e-13	0.002
1	6	0.063	0.057
3	16	0.001	0.035
4	4	0.063	0.952
6	6	0.016	0.671
11	4	0.004	0.070
8	19	6.103515625e-05	0.410
1	7	0.063	0.038
55	21	3.63797880709171e-12	0.035
20	29	5.96046447753907e-08	0.637
35	45	9.09494701772929e-13	0.681
1	6	0.063	0.057
4	3	0.063	0.741
19	28	5.96046447753907e-08	0.614
57	79	3.3881317890172e-21	0.657
NA	7	0.063	NA
24	19	2.38418579101562e-07	0.475
41	61	4.44089209850065e-16	0.450
2	6	0.063	0.121
9	15	0.000	0.795
NA	17	0.004	NA
12	NA	0.016	NA
8	41	5.96046447753907e-08	0.025
26	54	9.09494701772929e-13	0.123
59	61	8.67361737988403e-19	0.917
8	7	0.004	0.544
NA	76	7.46695150155575e-10	NA
26	NA	0.000	NA
10	5	0.004	0.169
12	23	3.814697265625e-06	0.308
21	52	1.45519152283668e-11	0.050

4	9	0.016	0.491
3	14	0.004	0.057
89	104	1.53102124485295e-26	0.995
20	29	5.96046447753907e-08	0.637
NA	76	7.46695150155575e-10	NA
22	56	1.81898940354586e-12	0.043
8	7	0.004	0.544
8	NA	0.063	NA
NA	7	0.063	NA
NA	22	0.011	NA
15	NA	0.004	NA
154	126	7.17464813734314e-43	0.257
10	16	0.000	0.846
10	7	0.004	0.261
5	10	0.004	0.599
25	25	2.98023223876953e-08	0.797
31	37	5.82076609134675e-11	0.814
6	10	0.004	0.782
18	34	1.49011611938477e-08	0.322
2	9	0.016	0.101
5	10	0.004	0.599
10	5	0.004	0.169
21	52	1.45519152283668e-11	0.050
31	37	5.82076609134675e-11	0.814
4	4	0.063	0.952
31	16	5.96046447753907e-08	0.120
14	7	0.001	0.097
25	40	2.32830643653869e-10	0.354
16	10	0.000	0.192
11	12	0.000	0.678
6	11	0.004	0.681
NA	10	0.031	NA
109	113	3.85185988877449e-34	0.764
NA	8	0.063	NA
2	8	0.031	0.140
10	12	0.000	0.792
24	NA	0.000	NA
4	4	0.063	0.952
6	9	0.004	0.895
8	8	0.004	0.671
7	13	0.001	0.666
9	4	0.016	0.124
10	12	0.000	0.792
5	7	0.016	0.970
3	5	0.063	0.451
7	NA	0.063	NA
3	5	0.063	0.451
7	NA	0.063	NA
NA	90	2.8421709430404e-14	NA
24	NA	0.000	NA
80	NA	9.09494701772929e-13	NA
24	NA	0.000	NA
9	5	0.008	0.219
12	NA	0.016	NA
8	9	0.004	0.714

40	27	5.82076609134675e-11	0.266
32	12	2.38418579101562e-07	0.029
14	14	6.103515625e-05	0.579
13	4	0.004	0.015
5	5	0.031	0.671
4	3	0.063	0.741
8	2	0.031	0.020
4	4	0.063	0.952
8	2	0.031	0.020
47	68	3.46944695195362e-18	0.490
8	7	0.004	0.544
9	NA	0.063	NA
5	5	0.031	0.671
16	43	9.31322574615481e-10	0.093
8	NA	0.063	NA
15	2	0.004	0.003
44	8	1.49011611938477e-08	0.005
44	8	1.49011611938477e-08	0.005
35	5	9.53674316406249e-07	0.002
NA	90	2.8421709430404e-14	NA
NA	90	2.8421709430404e-14	NA
NA	90	2.8421709430404e-14	NA
NA	90	2.8421709430404e-14	NA
61	56	3.46944695195362e-18	0.700
36	23	9.31322574615481e-10	0.256
36	23	9.31322574615481e-10	0.256
36	23	9.31322574615481e-10	0.256
36	23	9.31322574615481e-10	0.256
12	23	3.814697265625e-06	0.308
36	23	9.31322574615481e-10	0.256
36	23	9.31322574615481e-10	0.256
12	NA	0.016	NA
12	11	0.000	0.488
51	25	3.63797880709171e-12	0.062
61	56	3.46944695195362e-18	0.700
61	56	3.46944695195362e-18	0.700
36	13.5	2.98023223876953e-08	0.029
NA	90	2.8421709430404e-14	NA
4	10	0.008	0.405
27	24	1.49011611938477e-08	0.626
176	152	4.27642353614753e-50	0.388
253	224	2.26391976970673e-72	0.459
154	126	7.17464813734314e-43	0.257
98	46	2.11758236813575e-22	0.020
40	34	7.27595761418345e-12	0.571
44.33333333	59.66666667	2.22044604925033e-16	0.604
47	44	1.4210854715202e-14	0.735
78	63	8.47032947254304e-22	0.346
67	50	3.46944695195362e-18	0.382
49	33	4.54747350886465e-13	0.264
17	5	0.000	0.012
178	62	7.52316384526271e-37	0.020
102	53	3.30872245021212e-24	0.038
22	16	1.9073486328125e-06	0.378
48	48	3.5527136788005e-15	0.855

32	14.5	1.19209289550781e-07	0.070
166	82	4.70197740328916e-38	0.028
25	22	5.96046447753907e-08	0.612
25.5	12.5	1.9073486328125e-06	0.098
47	44	1.4210854715202e-14	0.735
2	16	0.002	0.038
7	18	0.000	0.342
34	6.666666667	9.53674316406249e-07	0.007
NA	22	0.000	NA
42	54	3.5527136788005e-15	0.681
9.5	4	0.008	0.107
277	289	6.43444698683513e-86	0.940
364	447	6.05092486695211e-123	0.376
222	174	2.48920611114444e-60	0.178
3	4	0.063	0.652
24	34	1.86264514923096e-09	0.670
24	34	1.86264514923096e-09	0.670
58	73	1.35525271560689e-20	0.844
20	39	9.31322574615481e-10	0.292
132	151	1.79366203433576e-43	0.594
235	144	6.3723676445299e-58	0.010
190	267	2.31825384417966e-69	0.120
111	58	5.1698788284564e-26	0.039
3	5	0.063	0.451
16	25	9.53674316406249e-07	0.536
8	10	0.002	0.842
67	72	8.47032947254304e-22	0.835
17	2	0.001	0.002
5	3	0.063	0.527
NA	10	0.031	NA
34	28	4.65661287307738e-10	0.524
28	21	5.96046447753907e-08	0.412
34	21.33333333	3.72529029846191e-09	0.242
NA	13	0.016	NA
10	20	3.0517578125e-05	0.582
14	24	1.9073486328125e-06	0.424
4	9	0.016	0.491
2	17	0.001	0.032
49	52	8.88178419700125e-16	0.966
26	52	1.81898940354586e-12	0.146
19	18	3.814697265625e-06	0.717
88	86	6.46234853557053e-27	0.651
61	21	4.54747350886465e-13	0.020
NA	8	0.063	NA
58	46	2.22044604925033e-16	0.465
6	7	0.016	0.831
6	4	0.031	0.327
9	2	0.016	0.013
38	37	3.63797880709171e-12	0.806
13	13	0.000	0.579
5	12	0.004	0.400
8	15	0.000	0.655
11	15	0.000	0.952
6	13	0.001	0.498
53	64	3.46944695195362e-18	0.793

65	81	1.05879118406788e-22	0.864
135	160	2.80259692864967e-45	0.478
NA	48	5.96046447753907e-08	NA
74	79	1.32348898008484e-23	0.822
22	18	9.53674316406249e-07	0.516
42	67	5.55111512312579e-17	0.357
14	23	3.814697265625e-06	0.474
25	16	9.53674316406249e-07	0.258
14	23	3.814697265625e-06	0.474
9	NA	0.063	NA
51	46	3.5527136788005e-15	0.670
93	122	3.08148791101956e-33	0.761
44	NA	2.38418579101562e-07	NA
43	NA	2.38418579101562e-07	NA
NA	27	6.103515625e-05	NA
NA	56	3.72529029846191e-09	NA
NA	47	5.96046447753907e-08	NA
19	25	2.38418579101562e-07	0.778
54	71	2.168404344971e-19	0.643
4	4	0.063	0.952
8	11	0.001	0.963
2	31	1.52587890625e-05	9.27137397619686e-05
5	34	9.53674316406249e-07	0.008
2	7	0.063	0.196
3	7	0.031	0.461
8	11	0.001	0.963
50	57	5.55111512312579e-17	0.899
5	3	0.063	0.527
12	18	3.0517578125e-05	0.927
20	13	1.52587890625e-05	0.271
NA	18	0.002	NA
4	3	0.063	0.741
3	7	0.031	0.461
8	10	0.002	0.842
8	3	0.016	0.077
2	9	0.016	0.101
6	1	0.063	0.018
33	22	3.72529029846191e-09	0.293
6	3	0.063	0.169
8	NA	0.063	NA
NA	7	0.063	NA
NA	9	0.063	NA
39	27	1.16415321826935e-10	0.293
23	21	2.38418579101562e-07	0.664
NA	20	0.001	NA
4	9	0.016	0.491
3	5	0.063	0.451
3	4	0.063	0.652
5	6	0.016	0.862
3	6	0.063	0.599
2	6	0.063	0.121
3	12	0.004	0.140
5	2	0.063	0.237
2	6	0.063	0.121
4	3	0.063	0.741

35.33333333	58	7.10542735760099e-15	0.323
8	12	0.001	0.927
1	6	0.063	0.057
15	26	9.53674316406249e-07	0.411
4	5	0.063	0.906
12	11	0.000	0.488
9	21	3.0517578125e-05	0.426
3	7	0.031	0.461
87	78	2.06795153138258e-25	0.500
19	13	1.52587890625e-05	0.246
29	48	3.63797880709171e-12	0.314
20	35	3.72529029846191e-09	0.401
31	28	9.31322574615481e-10	0.648
17	17	7.62939453125e-06	0.797
26	24	2.98023223876953e-08	0.679
48	25	1.45519152283668e-11	0.085
4	3	0.063	0.741
46	46	1.4210854715202e-14	0.855
5	5	0.031	0.671
28	15	2.38418579101562e-07	0.139
36	52	5.68434188608079e-14	0.493
8	10	0.002	0.842
32	53	2.27373675443232e-13	0.313
NA	15	0.004	NA
43	41	2.27373675443232e-13	0.768
51	36	5.68434188608079e-14	0.314
51	43	7.10542735760099e-15	0.558
31	29	9.31322574615481e-10	0.698
25	19	2.38418579101562e-07	0.427
10	39	5.96046447753907e-08	0.016
33	34	5.82076609134675e-11	0.910
9	13	0.000	0.975
27	36	2.32830643653869e-10	0.759
7	5	0.016	0.376
5	4	0.063	0.465
3	5	0.063	0.451
4	5	0.063	0.906
1	6	0.063	0.057
NA	7	0.063	NA
2	8	0.031	0.140
6	9	0.004	0.895
6	9	0.004	0.895
7	5	0.016	0.376
12	7	0.001	0.157
12	6	0.002	0.097
6	4	0.031	0.327
4	4	0.063	0.952
8	3	0.016	0.077
6	4	0.031	0.327
5	2	0.063	0.237
5	3	0.063	0.527
12	7	0.001	0.157
5	3	0.063	0.527
6	1	0.063	0.018
5	5	0.031	0.671

9	4	0.016	0.124
6	7	0.016	0.831
6	2	0.063	0.152
7	4	0.016	0.233
7	3	0.031	0.113
5	2	0.063	0.237
6	2	0.063	0.152
7	2	0.063	0.032
8	4	0.016	0.169
8	7	0.004	0.544
18	13	1.52587890625e-05	0.283
7	7	0.008	0.671
5	2	0.063	0.237
10	6	0.004	0.261
7	9	0.004	0.937
5	2	0.063	0.237
8	4	0.016	0.169
6	5	0.016	0.500
8	5	0.016	0.285
10	9	0.001	0.470
11	12	0.000	0.678
16	10	0.000	0.192
3	10	0.016	0.220
3	5	0.063	0.451
10	10	0.001	0.579
83	127	2.46519032881566e-32	0.485
12	11	0.000	0.488
34	20.5	7.45058059692382e-09	0.212
2	12	0.008	0.095
2	7	0.063	0.196
3	10	0.016	0.220
35.5	47	4.54747350886465e-13	0.631
29	22	1.49011611938477e-08	0.425
NA	7	0.063	NA
69	49	1.73472347597681e-18	0.321
62	59	8.67361737988403e-19	0.764
18	14	1.52587890625e-05	0.340
64	31.5	3.5527136788005e-15	0.063
68	59	5.42101086242751e-20	0.604
2	9	0.016	0.101
NA	37	3.814697265625e-06	NA
166	153	6.84227765783601e-49	0.589
9	3	0.016	0.053
11	18	6.103515625e-05	0.818
NA	14	0.008	NA
30	23	1.49011611938477e-08	0.437
3	13	0.004	0.113
4	12	0.004	0.279
2	15	0.004	0.008
3	8	0.016	0.357
6	14	0.001	0.426
18	22	9.53674316406249e-07	0.891
14	18	1.52587890625e-05	0.878
12	12	0.000	0.579
NA	14	0.008	NA

39	47	1.13686837721616e-13	0.796
12	12	0.000	0.579
8	6	0.008	0.413
6	11	0.004	0.681
2	7	0.063	0.196
151	157	4.37905770101507e-47	0.953
3	5	0.063	0.451
NA	7	0.063	NA
3	7	0.031	0.461
44	66	2.7755575615629e-17	0.438
4	4	0.063	0.952
4	3	0.063	0.741
5	13	0.002	0.333
606	728	1.63302522078783e-201	0.438
13	2	0.004	0.011
12	NA	0.016	NA
5	3	0.063	0.527
7	4	0.016	0.233
2	8	0.031	0.140
4	13	0.004	0.185
6	12	0.002	0.582
NA	12	0.016	NA
59	34	1.4210854715202e-14	0.137
2	11	0.016	0.116
8	3	0.016	0.077
5	7	0.016	0.970
4	6	0.031	0.895
13	12	0.000	0.495
55	90	2.11758236813575e-22	0.384
2	5	0.063	0.192
14	14	6.103515625e-05	0.579
27	22	5.96046447753907e-08	0.510
16	16	1.52587890625e-05	0.579
16	8	0.000	0.097
3	9	0.016	0.279
1	6	0.063	0.057
2	5	0.063	0.192
8	12	0.001	0.927
42	NA	4.76837158203125e-07	NA
NA	8	0.063	NA
4	3	0.063	0.741
3	4	0.063	0.652
5	8	0.016	0.826
7	2	0.063	0.032
12	9	0.001	0.311
15	4	0.001	0.031
17.5	60	1.81898940354586e-12	0.021
5	3	0.063	0.527
13	5	0.002	0.038
11	15	0.000	0.952
4	4	0.063	0.952
9	16	0.000	0.717
49	31	9.09494701772929e-13	0.206
64	53	3.46944695195362e-18	0.530
57	80	3.3881317890172e-21	0.633

19	28	5.96046447753907e-08	0.614
3	7	0.031	0.461
11	9	0.001	0.382
2	8	0.031	0.140
19	3	0.000	0.005
15	NA	0.004	NA
NA	8	0.063	NA
NA	9	0.063	NA
58	42	8.88178419700125e-16	0.344
46	34	9.09494701772929e-13	0.370
4.2	5	0.031	0.853
47	54	8.88178419700125e-16	0.885
17	13	3.0517578125e-05	0.326
22	2	0.000	0.000
8	8	0.004	0.671
14	8	0.000	0.148
22	12	7.62939453125e-06	0.148
10	23	1.52587890625e-05	0.169
32	35	5.82076609134675e-11	0.977
46	16.5	4.65661287307738e-10	0.023
4	5	0.063	0.906
6	5	0.016	0.500
NA	12	0.016	NA
10	11	0.001	0.687
5.5	10	0.004	0.692
5	4	0.063	0.465
5	2	0.063	0.237
2	5	0.063	0.192
2	16	0.002	0.038
10	16.5	0.000	0.807
54	19	1.45519152283668e-11	0.023
2	10	0.016	0.075
3	4	0.063	0.652
56	20	3.63797880709171e-12	0.025
13	2	0.004	0.011
6	6	0.016	0.671
7	NA	0.063	NA
30	104	6.77626357803444e-21	0.045
5	17	0.000	0.163
NA	7	0.063	NA
6	2	0.063	0.152
4	5	0.063	0.906
35	23	1.86264514923096e-09	0.280
27	28	3.72529029846191e-09	0.853
4	6	0.031	0.895
10	11	0.001	0.687
7	4	0.016	0.233
5	4	0.063	0.465
2	7	0.063	0.196
103	61	2.06795153138258e-25	0.082
3	13	0.004	0.113
10	6	0.004	0.261
6	1	0.063	0.018
12	14	0.000	0.758
21	19	9.53674316406249e-07	0.651

20	18	1.9073486328125e-06	0.643
NA	17	0.004	NA
NA	8	0.063	NA
20	24	2.38418579101562e-07	0.920
20	24	2.38418579101562e-07	0.920
51	40	1.4210854715202e-14	0.449
10	9	0.001	0.470
10	6	0.004	0.261
8	2	0.031	0.020
12	14	0.000	0.758
10	13	0.000	0.892
24	10	7.62939453125e-06	0.048
3	4	0.063	0.652
14	17	1.52587890625e-05	0.806
5	9	0.008	0.702
22	31	1.49011611938477e-08	0.678
26	21	5.96046447753907e-08	0.499
14	4	0.002	0.011
9	1	0.031	0.003
8	7	0.004	0.544
20	18	1.9073486328125e-06	0.643
4	5	0.063	0.906
6	8	0.008	0.976
56	32	5.68434188608079e-14	0.132
6	7	0.016	0.831
57	62	8.67361737988403e-19	0.987
25	23	5.96046447753907e-08	0.674
10	8	0.002	0.363
32	16	5.96046447753907e-08	0.106
12	12	0.000	0.579
6	2	0.063	0.152
10	4	0.008	0.093
6	6	0.016	0.671
21	27	5.96046447753907e-08	0.813
22	30	1.49011611938477e-08	0.725
22	30	1.49011611938477e-08	0.725
27	34	9.31322574615481e-10	0.845
18	23	9.53674316406249e-07	0.823
24	24	5.96046447753907e-08	0.797
4	13	0.004	0.185
5	30	3.814697265625e-06	0.013
831	748	7.1553129269534e-64	0.511
41	37	1.81898940354586e-12	0.670
34	29	2.32830643653869e-10	0.570
26	16	4.76837158203125e-07	0.227
34	26	9.31322574615481e-10	0.434
27	25	1.49011611938477e-08	0.684
39	19	1.86264514923096e-09	0.096
40	26	1.16415321826935e-10	0.230
55	36	1.4210854715202e-14	0.236
19	13	1.52587890625e-05	0.246
27	21	5.96046447753907e-08	0.454
32	19	1.49011611938477e-08	0.201
50	38	5.68434188608079e-14	0.406
18	14	1.52587890625e-05	0.340

24	21	2.38418579101562e-07	0.604
46	33	9.09494701772929e-13	0.333
27.5	32	9.31322574615481e-10	0.968
37	27	2.32830643653869e-10	0.382
37	29	1.16415321826935e-10	0.448
24.5	33	1.86264514923096e-09	0.743
49	31	9.09494701772929e-13	0.206
70	51	8.67361737988403e-19	0.352
2	6	0.063	0.121
7.428571429	34	4.76837158203125e-07	0.006
33	25	1.86264514923096e-09	0.423
43	31	7.27595761418345e-12	0.339
37	28	2.32830643653869e-10	0.400
40.5	32.5	1.45519152283668e-11	0.482
61	41	4.44089209850065e-16	0.262
16.5	9	0.000	0.128
15	15	3.0517578125e-05	0.579
61	38	8.88178419700125e-16	0.193
29	24	1.49011611938477e-08	0.530
29	21	2.98023223876953e-08	0.374
7	1	0.063	0.028
5	2	0.063	0.237
10	5	0.004	0.169
5	2	0.063	0.237
4	3	0.063	0.741
43	31.5	7.27595761418345e-12	0.358
16	8	0.000	0.097
8	19	6.103515625e-05	0.410
1	16	0.004	0.003
2	7	0.063	0.196
6	2	0.063	0.152
18	14	1.52587890625e-05	0.340
3	8	0.016	0.357
5	13	0.002	0.333
11	11	0.000	0.579
28	21	5.96046447753907e-08	0.412
8	9	0.004	0.714
4	7	0.016	0.731
23	22	2.38418579101562e-07	0.731
24	30	7.45058059692382e-09	0.856
4	6	0.031	0.895
25	24	5.96046447753907e-08	0.736
19	17	3.814697265625e-06	0.635
5	11	0.004	0.511
5	11	0.004	0.511
NA	10	0.031	NA
20	19	9.53674316406249e-07	0.721
2	6	0.063	0.121
22	21	2.38418579101562e-07	0.728
5	7	0.016	0.970
3	9	0.016	0.279
11	15	0.000	0.952
NA	15	0.004	NA
2	13	0.004	0.078
5	6	0.016	0.862

5	5	0.031	0.671
14	9	0.000	0.208
18	26	2.38418579101562e-07	0.642
9	19	6.103515625e-05	0.524
19	17	3.814697265625e-06	0.635
15	14	6.103515625e-05	0.506
4	3	0.063	0.741
4	10	0.008	0.405
2	5	0.063	0.192
17	15	1.52587890625e-05	0.451
27	15	4.76837158203125e-07	0.159
3	10	0.016	0.220
4	18	0.000	0.065
6	11	0.004	0.681
6	10	0.004	0.782
47	49	3.5527136788005e-15	0.933
4	4	0.063	0.952
NA	8	0.063	NA
20	9	6.103515625e-05	0.068
18	14	1.52587890625e-05	0.340
5	7	0.016	0.970
10	9	0.001	0.470
7	7	0.008	0.671
20	12	1.52587890625e-05	0.171
43	24	5.82076609134675e-11	0.119
8	2	0.031	0.020
16	16	1.52587890625e-05	0.579
5	2	0.063	0.237
2	8	0.031	0.140
54	88	4.23516473627151e-22	0.390
2	5	0.063	0.192
11	12	0.000	0.678
5	8	0.016	0.826
7	9	0.004	0.937
7	11	0.002	0.868
5	8	0.016	0.826
16	13	6.103515625e-05	0.376
5	5	0.031	0.671
30	24	7.45058059692382e-09	0.488
15	14	6.103515625e-05	0.506
3	4	0.063	0.652
4	3	0.063	0.741
19	49	5.82076609134675e-11	0.040
23	50	1.45519152283668e-11	0.099
35	45	9.09494701772929e-13	0.681
11	23	7.62939453125e-06	0.234
7	17	0.000	0.390
17	27	2.38418579101562e-07	0.515
20	29	5.96046447753907e-08	0.637
26	54	9.09494701772929e-13	0.123
6	21	6.103515625e-05	0.149
13	27	9.53674316406249e-07	0.239
4	13	0.004	0.185
19	28	5.96046447753907e-08	0.614
18	28	1.19209289550781e-07	0.542

13	19	1.52587890625e-05	0.960
26	50	3.63797880709171e-12	0.173
41	61	4.44089209850065e-16	0.450
15	25	9.53674316406249e-07	0.456
27	37	2.32830643653869e-10	0.718
21	28	5.96046447753907e-08	0.759
13	20	1.52587890625e-05	0.557
4	9	0.016	0.491
26	80	1.11022302462516e-16	0.037
29	37	1.16415321826935e-10	0.695
14	35	5.96046447753907e-08	0.124
9	21	3.0517578125e-05	0.426
9	15	0.000	0.795
6	11	0.004	0.681
10	16	0.000	0.846
6	8	0.008	0.976
10	16	0.000	0.846
7	10	0.004	0.989
11	20	1.52587890625e-05	0.690
17	43	9.31322574615481e-10	0.118
78	102	8.07793566946318e-28	0.768
24	43	5.82076609134675e-11	0.232
9	41	2.98023223876953e-08	0.007
21	52	1.45519152283668e-11	0.050
20	NA	0.001	NA
33	45	9.15771636300637e-07	0.582
17	53	2.91038304567338e-11	0.034
84	40.5	2.168404344971e-19	0.057
4	14	0.002	0.149
6	22	6.103515625e-05	0.129
8	41	5.96046447753907e-08	0.025
2	26	6.103515625e-05	0.007
7	8	0.004	0.809
41	62	2.22044604925033e-16	0.427
27	47	7.27595761418345e-12	0.260
18	33	1.49011611938477e-08	0.351
24	21	2.38418579101562e-07	0.604
35	50	2.27373675443232e-13	0.509
19	35	7.45058059692382e-09	0.347
10.5	29	9.53674316406249e-07	0.083
67	88	3.30872245021212e-24	0.759
37	48	2.27373675443232e-13	0.666
67	119	1.00974195868289e-28	0.279
57	84	8.47032947254304e-22	0.547
31	37	5.82076609134675e-11	0.814
29	26	3.72529029846191e-09	0.638
15	19	7.62939453125e-06	0.836
17	24	9.53674316406249e-07	0.675
19	28	5.96046447753907e-08	0.614
22	56	1.81898940354586e-12	0.043
15	24	9.53674316406249e-07	0.506
42	47	5.68434188608079e-14	0.934
25	19	2.38418579101562e-07	0.427
8	6	0.008	0.413
3	11	0.008	0.175

120	157	2.86985925493724e-42	0.230
57	79	3.3881317890172e-21	0.657
37	98	3.3881317890172e-21	0.035
26	28	7.45058059692382e-09	0.911
19	24	2.38418579101562e-07	0.840
NA	17	0.004	NA
57	81	1.6940658945086e-21	0.611
34	49	2.27373675443232e-13	0.496
35	62	3.5527136788005e-15	0.243
86	86	1.29246970711411e-26	0.694
86	86	1.29246970711411e-26	0.694
2	5	0.063	0.192
7	4	0.016	0.233
7	3	0.031	0.113
15	53	5.82076609134675e-11	0.018
5	9	0.008	0.702
1	6	0.063	0.057
10	13	0.000	0.892
38	40	1.81898940354586e-12	0.951
16	18	7.62939453125e-06	0.979
6	8	0.008	0.976
6.333333333	26.5	1.52587890625e-05	0.011
35	25	9.31322574615481e-10	0.359
42	30	1.45519152283668e-11	0.328
8	2	0.031	0.020
11	1	0.016	0.002
11	NA	0.016	NA
5	4	0.063	0.465
3	4	0.063	0.652
37	45	4.54747350886465e-13	0.780
54	41	3.5527136788005e-15	0.404
20	18	1.9073486328125e-06	0.643
NA	10	0.031	NA
1	8	0.063	0.045
7	9	0.004	0.937
10	12	0.000	0.792
5	7	0.016	0.970
4	7	0.016	0.731
14	9	0.000	0.208
3	13	0.004	0.113
15	21	3.814697265625e-06	0.687
7	14	0.001	0.582
NA	10	0.031	NA
14	12	0.000	0.424
6	14	0.001	0.426
3	5	0.063	0.451
6	5	0.016	0.500
6	5	0.016	0.500
23	18	9.53674316406249e-07	0.461
13.5	26	9.53674316406249e-07	0.304
23	19	4.76837158203125e-07	0.528
16.66666667	47	2.32830643653869e-10	0.076
61	74	3.3881317890172e-21	0.918
21	7	6.103515625e-05	0.021
43	44	5.68434188608079e-14	0.898

3	4	0.063	0.652
21	7	6.103515625e-05	0.021
12	4	0.004	0.053
46	26	1.45519152283668e-11	0.126
23	15	1.9073486328125e-06	0.274
83	27	2.7755575615629e-17	0.015
55	41	3.5527136788005e-15	0.380
5	15	0.001	0.231
NA	11	0.016	NA
92	71	2.06795153138258e-25	0.290
11	14	0.000	0.865
8	6	0.008	0.413
1	9	0.031	0.033
2	7	0.063	0.196
38	71	5.55111512312579e-17	0.196
42	79	8.67361737988403e-19	0.190
14	1	0.004	0.001
11	22	1.52587890625e-05	0.270
19	3	0.000	0.005
11	17	6.103515625e-05	0.889
7	NA	0.063	NA
21	14	3.814697265625e-06	0.293
8	6	0.008	0.413
5	5	0.031	0.671
52	74	1.08420217248551e-19	0.515
55	47	4.44089209850065e-16	0.579
5	5	0.031	0.671
21	13	7.62939453125e-06	0.231
17	13	3.0517578125e-05	0.326
21	17	1.9073486328125e-06	0.502
7	6	0.016	0.525
13	11	0.000	0.412
16	15	1.52587890625e-05	0.511
8	6	0.008	0.413
4	3	0.063	0.741
4	4	0.063	0.952
21	17	1.9073486328125e-06	0.502
4	4	0.063	0.952
NA	7	0.063	NA
3	5	0.063	0.451
NA	7	0.063	NA
NA	8	0.063	NA
4	7	0.016	0.731
134	121	2.93873587705572e-39	0.512
19	7	0.000	0.032
134	NA	6.77626357803444e-21	NA
130	86	3.08148791101956e-33	0.146
5	8	0.016	0.826
3	6	0.063	0.599
3	5	0.063	0.451
4	5	0.063	0.906
5	12	0.004	0.400
4	12	0.004	0.279
8	16	0.000	0.582
4	9	0.016	0.491

2	8	0.031	0.140
NA	8	0.063	NA
14	15	6.103515625e-05	0.657
64	32	3.5527136788005e-15	0.069
11	10	0.001	0.480
25	17	4.76837158203125e-07	0.311
6	11	0.004	0.681
14	14	6.103515625e-05	0.579
NA	14	0.008	NA
18	11	6.103515625e-05	0.180
13	10	0.000	0.331
14.5	11.66666667	0.000	0.368
10	7	0.004	0.261
NA	8	0.063	NA
NA	10	0.031	NA
3	6	0.063	0.599
4	10	0.008	0.405
6	4	0.031	0.327
66	59	2.168404344971e-19	0.654
4	6	0.031	0.895
20	29	5.96046447753907e-08	0.637
5	9	0.008	0.702
5	5	0.031	0.671
8	15	0.000	0.655
7	NA	0.063	NA
16	4	0.001	0.026
14	5	0.001	0.028
40	21	9.31322574615481e-10	0.128
10	10	0.001	0.579
3	4	0.063	0.652
28.25	10.14285714	1.9073486328125e-06	0.023
14.33333333	8	0.000	0.138
28	27	3.72529029846191e-09	0.743
48	58	1.11022302462516e-16	0.792
6	6	0.016	0.671
6	19	0.000	0.200
11.5	26	1.9073486328125e-06	0.179
3	7	0.031	0.461
52	66	1.73472347597681e-18	0.704
NA	13	0.016	NA
18	22	9.53674316406249e-07	0.891
NA	11	0.016	NA
4	3	0.063	0.741
7	5	0.016	0.376
8	8	0.004	0.671
7	8.333333333	0.004	0.853
15	20	3.814697265625e-06	0.759
3	4	0.063	0.652
18	54	1.45519152283668e-11	0.016
5	4	0.063	0.465
30	5	3.814697265625e-06	0.003
5	7	0.016	0.970
3.5	9	0.016	0.384
3	9	0.016	0.279
5	11	0.004	0.511

9	3	0.016	0.053
NA	14	0.008	NA
4	6	0.031	0.895
11	NA	0.016	NA
34	72	1.11022302462516e-16	0.112
8	15	0.000	0.655
38	53	1.4210854715202e-14	0.546
9	13	0.000	0.975
6	6	0.016	0.671
5	19	0.000	0.116
4	7	0.016	0.731
12	NA	0.016	NA
10	NA	0.031	NA
7	NA	0.063	NA
8	7	0.004	0.544
8	16	0.000	0.582
14.6	27.5	4.76837158203125e-07	0.325
7	23	3.0517578125e-05	0.180
19	49	5.82076609134675e-11	0.040
26	7	1.52587890625e-05	0.027
3	12	0.004	0.140
4	5	0.063	0.906
30	11	9.53674316406249e-07	0.026
NA	8	0.063	NA
55	39	7.10542735760099e-15	0.320
321	196	1.53342073315196e-68	0.010
7	7	0.008	0.671
5	3	0.063	0.527
11	8	0.001	0.288
7	3	0.031	0.113
20	13	1.52587890625e-05	0.271
21	16	3.814697265625e-06	0.430
7	NA	0.063	NA
NA	10	0.031	NA
143	138	0.001	0.755
2	5	0.063	0.192
6	2	0.063	0.152
4	13	0.004	0.185
6	3	0.063	0.169
6.75	9	0.004	0.976
438	519	3.70800009297219e-130	0.479
35	NA	0.038	NA
275	325	4.90909346529781e-91	0.487
36	33	5.82076609134675e-11	0.698
99	103	3.94430452610504e-31	0.771
570	531	6.6321629748562e-158	0.626
569	533	3.34011058941486e-158	0.646
312	315	2.99627286700306e-95	0.930
395	403	7.74518382969872e-121	0.972
570	554	6.62433728422274e-170	0.782
591	582	3.94841270698451e-177	0.833
263	258	5.39760534693409e-79	0.818
5	9	0.008	0.702
31	16	5.96046447753907e-08	0.120
3	4	0.063	0.652

NA	10	0.031	NA
9	9	0.002	0.579
6	6	0.016	0.671
49	33	4.54747350886465e-13	0.264
11	9	0.001	0.382
12	12	0.000	0.579
18	13	1.52587890625e-05	0.283
12	1	0.016	0.001
10	5	0.004	0.169
1	12	0.016	0.014
6.5	30	3.814697265625e-06	0.037
10	5	0.004	0.169
10	5	0.004	0.169
5	2	0.063	0.237
2	5	0.063	0.192
7	4	0.016	0.233
11	7	0.002	0.202
4	9	0.016	0.491
3	5	0.063	0.451
9	3	0.016	0.053
2	5	0.063	0.192
21	17	1.9073486328125e-06	0.502
1	6	0.063	0.057
11	7	0.002	0.202
2	5	0.063	0.192
41	45	1.13686837721616e-13	0.970
13	14	6.103515625e-05	0.663
11	10	0.001	0.480
3	4	0.063	0.652
22	21	2.38418579101562e-07	0.728
4	5	0.063	0.906
76	70	1.05879118406788e-22	0.545
11	2	0.016	0.018
9	5	0.008	0.219
52	89	8.47032947254304e-22	0.324
5	13	0.002	0.333
13	9	0.000	0.254
14	14	6.103515625e-05	0.579
17.5	23	9.53674316406249e-07	0.780
NA	9	0.063	NA
2	7	0.063	0.196
5	2	0.063	0.237
22	25	5.96046447753907e-08	0.995
43	41	2.27373675443232e-13	0.768
23	10	1.52587890625e-05	0.059
64	60	2.168404344971e-19	0.737
NA	14	0.008	NA
4	9	0.016	0.491
4	19	0.000	0.054
3	14	0.004	0.057
25	36	9.31322574615481e-10	0.647
5	10	0.004	0.599
10	NA	0.031	NA
270	322	7.85454954447655e-90	0.459
19	18	3.814697265625e-06	0.717

43	35	1.81898940354586e-12	0.503
19	19	1.9073486328125e-06	0.797
12	11	0.000	0.488
397.5	487.5	8.80525457171081e-134	0.379
7	NA	0.063	NA
4	3	0.063	0.741
10	9	0.001	0.470
14	21	3.814697265625e-06	0.590
3	6	0.063	0.599
8	8	0.004	0.671
2	5	0.063	0.192
13	4	0.004	0.015
9	NA	0.063	NA
14	13	6.103515625e-05	0.501
13	15	6.103515625e-05	0.744
10	10	0.001	0.579
2	7	0.063	0.196
26	13	9.53674316406249e-07	0.106
10	7	0.004	0.261
12	6	0.002	0.097
12	5	0.004	0.051
4	4	0.063	0.952
17	32	5.96046447753907e-08	0.325
22.5	50	1.45519152283668e-11	0.088
12	28	9.53674316406249e-07	0.160
3	6	0.063	0.599
NA	12	0.016	NA
8	13	0.001	0.826
10	13	0.000	0.892
67	71	1.6940658945086e-21	0.807
151	145	2.80259692864967e-45	0.736
52	60	1.38777878078145e-17	0.877
56	62	1.73472347597681e-18	0.954
75	84	8.2718061255303e-25	0.918
92	91	2.01948391736578e-28	0.673
3	5	0.063	0.451
4	8	0.016	0.599
7	3	0.031	0.113

Log2 Ratio(differentiating NSC / proliferating NSC)	GO.BP Positive regulation of neurogenesis	GO.BP RNA related processes	Unique peptide count control	Unique peptide count differentiating NSC
-0.68	FALSE	FALSE	NA	3
-0.29	FALSE	FALSE	NA	3
-0.42	FALSE	FALSE	NA	1
-1.32	FALSE	FALSE	NA	8
1.00	FALSE	FALSE	NA	2
-0.42	FALSE	FALSE	NA	5
-0.51	FALSE	FALSE	NA	6
NA	FALSE	FALSE	NA	NA
-0.22	FALSE	FALSE	NA	19
-1.17	FALSE	FALSE	NA	7
-1.77	FALSE	FALSE	NA	5
0.61	FALSE	FALSE	NA	17
-0.49	FALSE	FALSE	NA	30
NA	FALSE	FALSE	NA	7
2.58	FALSE	FALSE	NA	5
1.32	FALSE	FALSE	NA	10
2.32	FALSE	FALSE	NA	8
1.63	FALSE	FALSE	NA	14
-0.65	FALSE	FALSE	NA	18
0.42	FALSE	FALSE	NA	4
-0.49	FALSE	FALSE	NA	22
2.17	FALSE	FALSE	NA	9
0.98	FALSE	FALSE	NA	26
0.00	FALSE	FALSE	NA	6
1.14	FALSE	FALSE	NA	9
NA	FALSE	FALSE	NA	NA
2.97	FALSE	FALSE	5	38
-0.78	FALSE	FALSE	NA	12
2.81	FALSE	FALSE	NA	24
-0.79	FALSE	FALSE	5	38
-0.38	FALSE	FALSE	NA	58
0.26	FALSE	FALSE	NA	60
-0.12	FALSE	FALSE	NA	15
-0.20	FALSE	FALSE	NA	13
-0.32	FALSE	FALSE	NA	12
0.38	TRUE	FALSE	NA	10
0.36	FALSE	FALSE	NA	5
0.42	FALSE	TRUE	NA	4
0.68	FALSE	FALSE	NA	7
NA	FALSE	FALSE	NA	NA
0.00	FALSE	FALSE	NA	13
0.58	FALSE	FALSE	NA	19
-0.19	FALSE	FALSE	NA	15
-0.07	FALSE	FALSE	NA	8
-0.74	FALSE	FALSE	NA	3
NA	FALSE	FALSE	NA	8
1.32	FALSE	FALSE	NA	3
-0.58	FALSE	FALSE	NA	6
0.35	FALSE	FALSE	NA	3

0.58	FALSE	FALSE	NA	16
0.66	FALSE	FALSE	NA	24
-1.47	FALSE	FALSE	NA	8
0.00	FALSE	TRUE	NA	7
0.75	FALSE	FALSE	NA	37
1.00	TRUE	FALSE	NA	6
1.46	FALSE	FALSE	NA	10
0.58	FALSE	FALSE	NA	16
0.47	FALSE	FALSE	NA	27
-0.32	FALSE	FALSE	NA	21
0.00	FALSE	FALSE	NA	8
-1.58	FALSE	FALSE	NA	1
-1.54	FALSE	FALSE	NA	15
0.05	FALSE	FALSE	NA	11
0.61	FALSE	FALSE	NA	8
-0.87	FALSE	FALSE	NA	5
-0.55	FALSE	FALSE	NA	10
-0.64	FALSE	FALSE	NA	11
-0.35	FALSE	FALSE	NA	8
-0.82	FALSE	FALSE	NA	14
0.58	FALSE	FALSE	NA	9
1.00	FALSE	FALSE	NA	14
1.08	FALSE	TRUE	NA	20
-0.78	FALSE	FALSE	NA	7
-0.52	FALSE	FALSE	NA	12
0.42	FALSE	FALSE	NA	5
0.42	FALSE	FALSE	NA	5
0.58	FALSE	FALSE	NA	4
0.35	FALSE	FALSE	NA	6
0.22	FALSE	FALSE	NA	7
-1.17	FALSE	FALSE	NA	4
-1.29	FALSE	FALSE	NA	15
0.47	FALSE	FALSE	NA	8
-0.07	FALSE	FALSE	NA	18
1.00	FALSE	FALSE	NA	9
-0.58	FALSE	FALSE	NA	3
-1.32	FALSE	FALSE	NA	2
0.42	FALSE	FALSE	NA	3
-0.85	FALSE	FALSE	NA	4
NA	FALSE	FALSE	NA	NA
-0.42	FALSE	FALSE	NA	7
NA	FALSE	FALSE	NA	NA
0.93	FALSE	FALSE	NA	34
-0.34	FALSE	FALSE	NA	33
-2.46	FALSE	FALSE	NA	2
-2.46	FALSE	FALSE	NA	2
NA	FALSE	FALSE	NA	NA
-1.17	FALSE	FALSE	NA	6
-0.32	FALSE	FALSE	NA	4
1.32	FALSE	FALSE	NA	9
-0.28	FALSE	FALSE	NA	11
NA	FALSE	FALSE	NA	NA
-1.87	FALSE	FALSE	NA	4
-0.68	FALSE	TRUE	NA	4
0.49	FALSE	FALSE	NA	5

-0.77	FALSE	FALSE	NA	10
-0.36	FALSE	FALSE	NA	6
NA	FALSE	FALSE	NA	9
-1.78	FALSE	FALSE	NA	5
NA	FALSE	FALSE	NA	7
0.42	FALSE	FALSE	NA	4
-1.51	FALSE	FALSE	NA	14
1.00	FALSE	FALSE	NA	6
-0.42	FALSE	FALSE	NA	2
-0.47	FALSE	FALSE	NA	4
-0.55	FALSE	FALSE	NA	11
-2.81	FALSE	FALSE	NA	1
0.15	FALSE	FALSE	NA	9
-1.17	FALSE	FALSE	NA	4
-0.58	FALSE	FALSE	NA	3
-0.74	FALSE	FALSE	NA	4
-0.66	FALSE	FALSE	NA	10
-2.01	FALSE	FALSE	NA	64
-4.00	FALSE	FALSE	NA	2
-0.88	FALSE	FALSE	NA	7
0.03	FALSE	FALSE	NA	23
-0.08	FALSE	FALSE	NA	12
-1.47	FALSE	FALSE	NA	5
0.27	FALSE	FALSE	NA	32
-1.00	FALSE	FALSE	NA	8
-0.58	FALSE	FALSE	NA	17
0.58	FALSE	FALSE	NA	6
0.54	FALSE	FALSE	NA	21
-0.81	FALSE	FALSE	NA	4
0.53	TRUE	FALSE	NA	12
-0.87	FALSE	FALSE	NA	6
0.15	FALSE	FALSE	NA	10
-0.06	FALSE	FALSE	NA	15
0.11	FALSE	FALSE	NA	12
1.87	FALSE	FALSE	NA	7
-1.22	FALSE	FALSE	NA	1
2.81	FALSE	TRUE	NA	7
0.64	FALSE	FALSE	NA	31
-0.64	FALSE	FALSE	NA	6
-1.23	FALSE	FALSE	NA	1
0.81	FALSE	FALSE	NA	6
0.85	FALSE	FALSE	NA	9
NA	FALSE	FALSE	NA	NA
-0.27	FALSE	FALSE	NA	35
-0.90	FALSE	FALSE	NA	29
0.19	FALSE	FALSE	NA	31
-0.04	FALSE	FALSE	NA	31
0.03	FALSE	FALSE	NA	26
0.11	FALSE	FALSE	NA	33
0.15	FALSE	FALSE	NA	38
-0.50	FALSE	FALSE	NA	16
0.58	FALSE	FALSE	NA	14
1.32	FALSE	FALSE	NA	15
0.22	FALSE	FALSE	NA	5
-0.58	FALSE	FALSE	NA	2

0.00	FALSE	TRUE	NA	4
-2.09	FALSE	FALSE	NA	4
NA	FALSE	FALSE	NA	NA
-1.46	FALSE	FALSE	NA	7
-0.81	FALSE	FALSE	NA	4
-0.24	FALSE	FALSE	NA	7
0.31	FALSE	FALSE	NA	16
0.00	FALSE	FALSE	NA	5
-0.42	FALSE	FALSE	NA	1
1.12	FALSE	TRUE	NA	7
-4.32	FALSE	FALSE	NA	1
NA	FALSE	FALSE	NA	NA
-0.48	FALSE	FALSE	NA	27
NA	FALSE	FALSE	NA	NA
NA	FALSE	FALSE	NA	NA
-1.32	FALSE	FALSE	NA	4
0.58	FALSE	FALSE	NA	5
1.58	FALSE	FALSE	NA	6
0.22	FALSE	FALSE	NA	4
-0.03	FALSE	FALSE	NA	95
-0.74	FALSE	FALSE	NA	9
-1.39	FALSE	FALSE	NA	4
-0.97	FALSE	FALSE	NA	11
-1.32	FALSE	TRUE	NA	8
NA	FALSE	FALSE	NA	7
NA	FALSE	FALSE	NA	NA
-0.42	FALSE	FALSE	NA	2
-1.57	FALSE	FALSE	NA	26
-1.07	FALSE	FALSE	NA	17
-1.05	FALSE	FALSE	NA	14
-1.07	FALSE	FALSE	NA	8
-1.21	FALSE	FALSE	NA	15
-1.21	FALSE	FALSE	NA	15
-1.22	FALSE	FALSE	NA	3
-0.42	FALSE	FALSE	NA	3
0.14	FALSE	FALSE	NA	10
0.15	FALSE	FALSE	NA	6
-0.38	FALSE	FALSE	NA	7
-0.81	FALSE	FALSE	NA	6
0.28	FALSE	FALSE	NA	10
-0.65	FALSE	FALSE	NA	5
-0.46	FALSE	FALSE	NA	5
-1.00	FALSE	FALSE	NA	3
0.93	FALSE	FALSE	NA	27
0.42	FALSE	TRUE	NA	2
-0.26	FALSE	FALSE	NA	5
1.04	FALSE	FALSE	NA	29
-0.58	FALSE	FALSE	NA	16
0.56	FALSE	FALSE	NA	28
-1.00	FALSE	FALSE	NA	5
-0.30	FALSE	FALSE	NA	14
-0.29	FALSE	FALSE	NA	8
0.00	FALSE	FALSE	NA	10
-0.68	FALSE	FALSE	NA	5
-0.58	FALSE	FALSE	NA	4

NA	FALSE	FALSE	NA	NA
0.00	FALSE	FALSE	NA	9
0.87	FALSE	FALSE	NA	11
0.36	FALSE	FALSE	NA	16
-1.38	FALSE	FALSE	NA	5
0.26	FALSE	FALSE	NA	6
1.00	FALSE	FALSE	NA	9
0.49	FALSE	FALSE	NA	7
0.58	FALSE	FALSE	NA	6
-2.32	FALSE	FALSE	NA	2
-1.72	FALSE	FALSE	NA	7
NA	FALSE	FALSE	NA	NA
-0.32	FALSE	FALSE	NA	6
-1.13	FALSE	FALSE	NA	1
NA	FALSE	FALSE	NA	NA
0.00	FALSE	FALSE	NA	5
0.11	FALSE	FALSE	NA	37
0.29	FALSE	FALSE	NA	3
0.00	FALSE	FALSE	NA	6
1.06	FALSE	FALSE	NA	13
0.85	FALSE	FALSE	NA	6
-2.58	FALSE	FALSE	NA	2
-1.75	FALSE	FALSE	NA	1
0.18	FALSE	FALSE	NA	12
0.00	FALSE	FALSE	NA	3
0.35	FALSE	FALSE	NA	39
-4.25	FALSE	FALSE	NA	1
-0.72	FALSE	TRUE	NA	19
0.57	FALSE	TRUE	NA	31
0.49	FALSE	FALSE	NA	6
1.93	FALSE	FALSE	NA	17
NA	FALSE	TRUE	NA	16
0.88	FALSE	TRUE	NA	27
-0.20	FALSE	FALSE	NA	35
-0.16	FALSE	TRUE	NA	31
-0.94	FALSE	TRUE	NA	8
0.85	FALSE	FALSE	NA	7
0.68	FALSE	FALSE	NA	7
1.32	FALSE	TRUE	NA	13
-0.32	FALSE	FALSE	NA	8
-3.17	FALSE	FALSE	NA	1
-2.32	FALSE	FALSE	NA	2
2.81	FALSE	TRUE	NA	7
1.44	FALSE	TRUE	NA	70
-2.00	FALSE	TRUE	NA	2
1.00	FALSE	TRUE	NA	6
-1.00	FALSE	TRUE	NA	3
0.42	FALSE	FALSE	NA	2
0.32	FALSE	FALSE	NA	9
0.22	FALSE	FALSE	NA	6
-0.74	FALSE	FALSE	NA	11
-0.32	FALSE	FALSE	NA	11
NA	FALSE	TRUE	NA	NA
-1.00	FALSE	FALSE	NA	6
1.00	FALSE	FALSE	NA	5

-0.45	FALSE	FALSE	NA	10
-0.47	FALSE	TRUE	NA	2
0.42	FALSE	FALSE	NA	5
NA	FALSE	FALSE	NA	NA
-2.50	FALSE	FALSE	NA	3
NA	FALSE	FALSE	NA	12
-1.14	FALSE	FALSE	NA	4
-0.54	FALSE	FALSE	NA	8
-1.12	FALSE	FALSE	NA	4
0.26	FALSE	FALSE	NA	3
0.42	FALSE	FALSE	NA	3
-0.44	FALSE	FALSE	NA	109
0.32	FALSE	FALSE	NA	10
-1.32	FALSE	FALSE	NA	2
0.74	FALSE	FALSE	NA	5
0.00	FALSE	FALSE	NA	4
0.32	FALSE	FALSE	NA	4
1.00	FALSE	FALSE	NA	5
2.05	FALSE	FALSE	NA	15
1.00	FALSE	FALSE	NA	8
-1.22	FALSE	TRUE	NA	3
-1.58	FALSE	FALSE	NA	2
-0.61	FALSE	FALSE	1	37
-2.95	FALSE	FALSE	NA	2
-0.87	FALSE	FALSE	NA	5
0.00	FALSE	FALSE	NA	7
0.31	FALSE	FALSE	NA	14
0.22	FALSE	FALSE	NA	46
NA	FALSE	FALSE	NA	7
0.60	FALSE	TRUE	NA	31
2.58	FALSE	FALSE	NA	6
0.00	FALSE	FALSE	NA	4
0.39	FALSE	FALSE	NA	5
0.30	FALSE	FALSE	NA	5
0.00	FALSE	FALSE	NA	14
-0.38	FALSE	FALSE	NA	7
-1.17	FALSE	FALSE	NA	4
-0.74	FALSE	FALSE	NA	6
-0.41	FALSE	FALSE	NA	23
-0.51	FALSE	FALSE	NA	12
-0.19	FALSE	FALSE	NA	21
-0.04	FALSE	FALSE	NA	63
-0.05	FALSE	FALSE	NA	34
-0.13	FALSE	FALSE	NA	24
0.33	FALSE	FALSE	NA	19
-0.28	FALSE	TRUE	NA	17
0.00	FALSE	FALSE	NA	11
0.26	FALSE	FALSE	NA	11
-0.24	FALSE	FALSE	NA	21
-0.15	FALSE	FALSE	NA	18
-0.19	FALSE	FALSE	NA	11
-0.19	FALSE	FALSE	NA	11
-0.35	FALSE	FALSE	NA	8
0.21	FALSE	FALSE	NA	28
-0.26	FALSE	FALSE	NA	9

0.15	FALSE	TRUE	NA	29
-0.09	FALSE	TRUE	NA	12
0.12	FALSE	TRUE	NA	19
-0.22	FALSE	TRUE	NA	13
-1.28	FALSE	TRUE	NA	5
-1.17	FALSE	TRUE	NA	26
-0.35	FALSE	FALSE	NA	19
-2.17	FALSE	FALSE	NA	2
-1.36	FALSE	FALSE	NA	2
-0.58	FALSE	FALSE	NA	8
-0.93	FALSE	FALSE	NA	10
-2.25	FALSE	FALSE	NA	4
-0.78	FALSE	FALSE	NA	6
0.17	FALSE	TRUE	NA	5
-0.17	FALSE	TRUE	NA	20
2.00	FALSE	FALSE	NA	8
0.42	FALSE	FALSE	NA	14
0.58	FALSE	FALSE	NA	6
-0.58	FALSE	FALSE	NA	4
-2.58	FALSE	FALSE	NA	1
NA	FALSE	FALSE	NA	NA
-1.58	FALSE	FALSE	NA	2
NA	FALSE	FALSE	NA	NA
-0.42	FALSE	FALSE	NA	5
-0.85	FALSE	FALSE	NA	2
-1.18	FALSE	FALSE	NA	9
-0.19	FALSE	FALSE	NA	57
1.58	FALSE	FALSE	NA	12
-0.32	FALSE	FALSE	NA	3
-3.00	FALSE	FALSE	NA	1
-4.17	FALSE	FALSE	NA	1
-0.35	FALSE	TRUE	NA	14
2.58	FALSE	FALSE	NA	11
-0.45	FALSE	FALSE	NA	9
1.22	FALSE	TRUE	NA	5
0.29	FALSE	TRUE	NA	9
-0.26	FALSE	TRUE	NA	4
-0.49	FALSE	TRUE	NA	5
0.00	FALSE	FALSE	NA	3
0.85	FALSE	TRUE	NA	5
0.89	FALSE	FALSE	NA	3
-0.32	FALSE	FALSE	NA	4
-0.57	FALSE	FALSE	NA	21
0.19	FALSE	FALSE	NA	6
0.00	FALSE	FALSE	NA	4
0.49	FALSE	FALSE	NA	6
-0.69	FALSE	FALSE	NA	10
-0.95	FALSE	FALSE	NA	11
0.77	FALSE	FALSE	NA	52
1.28	FALSE	TRUE	NA	14
-1.32	FALSE	FALSE	NA	8
NA	FALSE	FALSE	NA	7
-0.68	FALSE	FALSE	NA	7
0.00	FALSE	FALSE	NA	2
0.38	FALSE	FALSE	NA	18

0.17	FALSE	FALSE	NA	28
0.28	FALSE	FALSE	NA	11
-1.06	FALSE	FALSE	NA	10
-0.33	FALSE	FALSE	NA	139
0.00	FALSE	FALSE	NA	50
-2.00	FALSE	FALSE	NA	2
-1.46	FALSE	FALSE	NA	4
NA	FALSE	FALSE	NA	NA
NA	FALSE	FALSE	NA	NA
0.00	FALSE	FALSE	NA	4
1.07	FALSE	FALSE	NA	26
-0.77	FALSE	FALSE	NA	23
-0.47	FALSE	TRUE	NA	18
-2.58	FALSE	FALSE	NA	3
NA	FALSE	FALSE	NA	NA
0.05	FALSE	FALSE	NA	25
-0.93	FALSE	FALSE	NA	6
NA	FALSE	FALSE	NA	NA
0.28	FALSE	FALSE	NA	51
-0.22	FALSE	FALSE	2	27
0.00	FALSE	FALSE	NA	5
1.81	FALSE	TRUE	NA	2
1.70	FALSE	FALSE	NA	13
-0.19	FALSE	FALSE	NA	4
-2.34	FALSE	FALSE	NA	12
-2.58	FALSE	FALSE	NA	1
-2.42	FALSE	TRUE	NA	3
0.00	FALSE	FALSE	NA	2
0.00	FALSE	FALSE	NA	6
1.46	FALSE	FALSE	NA	9
-1.25	FALSE	FALSE	NA	8
-2.81	FALSE	FALSE	NA	1
1.39	FALSE	FALSE	NA	29
-0.54	FALSE	TRUE	NA	10
-0.36	FALSE	TRUE	NA	12
-2.58	FALSE	FALSE	NA	1
0.42	FALSE	FALSE	NA	4
-0.56	FALSE	TRUE	NA	10
-0.47	FALSE	FALSE	NA	23
NA	FALSE	FALSE	NA	NA
0.34	FALSE	FALSE	NA	15
-0.57	FALSE	TRUE	NA	16
-1.58	FALSE	TRUE	NA	1
-0.74	FALSE	FALSE	NA	5
NA	FALSE	FALSE	NA	NA
NA	FALSE	FALSE	NA	3
-2.36	FALSE	FALSE	NA	7
-1.05	FALSE	FALSE	NA	7
-0.05	FALSE	FALSE	NA	6
0.19	FALSE	TRUE	NA	4
NA	FALSE	FALSE	2	NA
NA	FALSE	FALSE	NA	7
1.00	FALSE	FALSE	NA	2
-0.94	FALSE	FALSE	NA	5
-1.31	FALSE	FALSE	NA	12

-1.17	FALSE	FALSE	NA	4
-2.22	FALSE	FALSE	NA	2
-0.22	FALSE	FALSE	2	27
-0.54	FALSE	FALSE	NA	10
NA	FALSE	FALSE	2	NA
-1.35	FALSE	FALSE	NA	6
0.19	FALSE	TRUE	NA	4
NA	FALSE	FALSE	NA	6
NA	FALSE	FALSE	NA	NA
NA	FALSE	FALSE	2	NA
NA	FALSE	FALSE	NA	10
0.29	FALSE	TRUE	NA	36
-0.68	FALSE	FALSE	NA	5
0.51	FALSE	FALSE	NA	5
-1.00	FALSE	FALSE	NA	3
0.00	FALSE	FALSE	NA	8
-0.26	FALSE	FALSE	NA	11
-0.74	FALSE	FALSE	NA	5
-0.92	FALSE	FALSE	NA	14
-2.17	FALSE	FALSE	NA	2
-1.00	FALSE	FALSE	NA	3
1.00	FALSE	FALSE	NA	2
-1.31	FALSE	FALSE	NA	12
-0.26	FALSE	FALSE	NA	11
0.00	FALSE	FALSE	NA	4
0.95	FALSE	FALSE	NA	24
1.00	FALSE	FALSE	NA	12
-0.68	FALSE	TRUE	NA	7
0.68	FALSE	FALSE	NA	14
-0.13	FALSE	FALSE	NA	10
-0.87	FALSE	FALSE	NA	6
NA	FALSE	FALSE	NA	NA
-0.05	FALSE	FALSE	NA	23
NA	FALSE	FALSE	NA	NA
-2.00	FALSE	FALSE	NA	2
-0.26	FALSE	FALSE	NA	8
NA	FALSE	FALSE	NA	17
0.00	FALSE	FALSE	NA	3
-0.58	FALSE	FALSE	NA	6
0.00	FALSE	FALSE	NA	6
-0.89	FALSE	FALSE	NA	7
1.17	FALSE	FALSE	NA	8
-0.26	FALSE	FALSE	NA	6
-0.49	FALSE	TRUE	NA	5
-0.74	FALSE	TRUE	NA	3
NA	FALSE	FALSE	NA	7
-0.74	FALSE	TRUE	NA	3
NA	FALSE	FALSE	NA	3
NA	FALSE	FALSE	NA	NA
NA	FALSE	FALSE	NA	4
NA	FALSE	FALSE	NA	9
NA	FALSE	FALSE	NA	4
0.85	FALSE	FALSE	NA	10
NA	FALSE	FALSE	NA	3
-0.17	FALSE	FALSE	NA	7

0.57	FALSE	FALSE	NA	32
1.42	FALSE	FALSE	NA	24
0.00	FALSE	FALSE	NA	11
1.70	FALSE	FALSE	NA	11
0.00	FALSE	FALSE	NA	5
0.42	FALSE	FALSE	NA	4
2.00	TRUE	FALSE	NA	7
0.00	FALSE	FALSE	NA	2
2.00	FALSE	FALSE	NA	8
-0.53	FALSE	FALSE	NA	33
0.19	FALSE	FALSE	NA	7
NA	FALSE	FALSE	NA	8
0.00	FALSE	FALSE	NA	3
-1.43	FALSE	FALSE	NA	13
NA	FALSE	FALSE	NA	4
2.91	FALSE	FALSE	NA	8
2.46	FALSE	FALSE	NA	14
2.46	FALSE	FALSE	NA	14
2.81	FALSE	FALSE	NA	10
NA	FALSE	FALSE	NA	NA
NA	FALSE	FALSE	NA	NA
NA	FALSE	FALSE	NA	NA
NA	FALSE	FALSE	NA	NA
0.12	FALSE	FALSE	NA	9
0.65	FALSE	FALSE	NA	13
0.65	FALSE	FALSE	NA	13
0.65	FALSE	FALSE	NA	13
0.65	FALSE	FALSE	NA	13
-0.94	FALSE	FALSE	NA	5
0.65	FALSE	FALSE	NA	13
0.65	FALSE	FALSE	NA	13
NA	FALSE	FALSE	NA	3
0.13	FALSE	FALSE	NA	3
1.03	FALSE	FALSE	NA	14
0.12	FALSE	FALSE	NA	9
0.12	FALSE	FALSE	NA	9
1.42	FALSE	FALSE	NA	13
NA	FALSE	FALSE	NA	NA
-1.32	FALSE	FALSE	NA	4
0.17	FALSE	TRUE	NA	9
0.21	FALSE	TRUE	NA	39
0.18	FALSE	TRUE	NA	51
0.29	FALSE	TRUE	NA	36
1.09	FALSE	FALSE	NA	22
0.23	FALSE	TRUE	NA	23
-0.43	FALSE	TRUE	NA	17
0.10	FALSE	FALSE	NA	17
0.31	FALSE	TRUE	NA	20
0.42	FALSE	TRUE	NA	25
0.57	FALSE	TRUE	NA	20
1.77	FALSE	TRUE	NA	11
1.52	FALSE	TRUE	NA	40
0.94	FALSE	TRUE	NA	37
0.46	FALSE	FALSE	NA	15
0.00	FALSE	TRUE	NA	32

1.14	FALSE	TRUE	NA	19
1.02	FALSE	TRUE	NA	50
0.18	FALSE	TRUE	NA	18
1.03	FALSE	FALSE	NA	9
0.10	FALSE	FALSE	NA	17
-3.00	FALSE	TRUE	NA	2
-1.36	FALSE	FALSE	NA	7
2.35	FALSE	TRUE	NA	20
NA	FALSE	FALSE	NA	NA
-0.36	FALSE	FALSE	NA	29
1.25	FALSE	FALSE	NA	16
-0.06	FALSE	FALSE	NA	58
-0.30	FALSE	FALSE	NA	74
0.35	FALSE	FALSE	NA	66
-0.42	FALSE	FALSE	NA	3
-0.50	FALSE	TRUE	NA	5
-0.50	FALSE	TRUE	NA	5
-0.33	FALSE	FALSE	NA	12
-0.96	FALSE	FALSE	NA	16
-0.19	FALSE	FALSE	NA	47
0.71	FALSE	TRUE	NA	55
-0.49	FALSE	FALSE	NA	55
0.94	FALSE	FALSE	NA	61
-0.74	FALSE	FALSE	NA	3
-0.64	FALSE	FALSE	NA	15
-0.32	FALSE	FALSE	NA	8
-0.10	FALSE	FALSE	NA	39
3.09	FALSE	FALSE	NA	12
0.74	FALSE	FALSE	NA	5
NA	FALSE	FALSE	NA	NA
0.28	FALSE	FALSE	NA	28
0.42	FALSE	FALSE	NA	18
0.67	FALSE	FALSE	NA	27
NA	FALSE	FALSE	NA	NA
-1.00	FALSE	FALSE	NA	6
-0.78	FALSE	FALSE	NA	6
-1.17	FALSE	FALSE	NA	3
-3.09	FALSE	FALSE	NA	2
-0.09	FALSE	FALSE	NA	29
-1.00	FALSE	FALSE	NA	16
0.08	FALSE	FALSE	NA	15
0.03	FALSE	FALSE	NA	56
1.54	FALSE	FALSE	NA	49
NA	FALSE	FALSE	NA	NA
0.33	FALSE	TRUE	NA	23
-0.22	FALSE	FALSE	NA	4
0.58	FALSE	FALSE	NA	6
2.17	FALSE	TRUE	NA	5
0.04	FALSE	TRUE	NA	24
0.00	FALSE	FALSE	NA	12
-1.26	FALSE	FALSE	NA	4
-0.91	FALSE	FALSE	NA	8
-0.45	FALSE	FALSE	NA	9
-1.12	FALSE	FALSE	NA	4
-0.27	FALSE	FALSE	NA	26

-0.32	FALSE	FALSE	NA	5
-0.25	FALSE	FALSE	NA	16
NA	FALSE	FALSE	NA	NA
-0.09	FALSE	FALSE	NA	6
0.29	FALSE	FALSE	NA	5
-0.67	FALSE	FALSE	NA	7
-0.72	FALSE	FALSE	NA	2
0.64	FALSE	FALSE	NA	2
-0.72	FALSE	FALSE	NA	2
NA	FALSE	FALSE	NA	7
0.15	FALSE	FALSE	NA	15
-0.39	FALSE	FALSE	NA	29
NA	FALSE	FALSE	NA	21
NA	FALSE	FALSE	NA	20
NA	FALSE	FALSE	NA	NA
NA	FALSE	FALSE	NA	NA
NA	FALSE	FALSE	NA	NA
-0.40	FALSE	FALSE	NA	2
-0.39	FALSE	FALSE	NA	7
0.00	FALSE	FALSE	NA	2
-0.46	FALSE	FALSE	NA	5
-3.95	FALSE	FALSE	NA	2
-2.77	FALSE	FALSE	NA	5
-1.81	FALSE	FALSE	NA	2
-1.22	FALSE	FALSE	NA	3
-0.46	FALSE	TRUE	NA	5
-0.19	FALSE	FALSE	NA	35
0.74	FALSE	FALSE	NA	5
-0.58	FALSE	FALSE	NA	10
0.62	FALSE	FALSE	NA	15
NA	FALSE	FALSE	NA	NA
0.42	FALSE	FALSE	NA	3
-1.22	FALSE	FALSE	NA	2
-0.32	FALSE	FALSE	NA	7
1.42	FALSE	FALSE	NA	7
-2.17	FALSE	FALSE	NA	2
2.58	FALSE	FALSE	NA	6
0.58	FALSE	FALSE	NA	31
1.00	FALSE	FALSE	NA	6
NA	FALSE	FALSE	NA	8
NA	FALSE	FALSE	NA	NA
NA	FALSE	FALSE	NA	NA
0.53	FALSE	FALSE	NA	36
0.13	FALSE	FALSE	NA	14
NA	FALSE	FALSE	NA	NA
-1.17	FALSE	FALSE	NA	4
-0.74	FALSE	FALSE	NA	3
-0.42	FALSE	TRUE	NA	2
-0.26	FALSE	TRUE	NA	4
-1.00	FALSE	FALSE	NA	3
-1.58	FALSE	FALSE	NA	2
-2.00	FALSE	FALSE	NA	3
1.32	FALSE	FALSE	NA	3
-1.58	FALSE	FALSE	NA	2
0.42	FALSE	FALSE	NA	3

-0.72	FALSE	FALSE	NA	35
-0.58	FALSE	FALSE	NA	6
-2.58	FALSE	FALSE	NA	1
-0.79	FALSE	FALSE	NA	12
-0.32	FALSE	FALSE	NA	4
0.13	FALSE	FALSE	NA	11
-1.22	TRUE	FALSE	NA	8
-1.22	FALSE	FALSE	NA	3
0.16	FALSE	FALSE	NA	38
0.55	FALSE	FALSE	NA	10
-0.73	FALSE	FALSE	NA	25
-0.81	FALSE	FALSE	NA	11
0.15	FALSE	FALSE	NA	18
0.00	FALSE	FALSE	NA	10
0.12	FALSE	FALSE	NA	14
0.94	FALSE	FALSE	NA	22
0.42	FALSE	FALSE	NA	2
0.00	FALSE	FALSE	NA	29
0.00	FALSE	FALSE	NA	4
0.90	FALSE	FALSE	NA	23
-0.53	FALSE	FALSE	NA	12
-0.32	FALSE	FALSE	NA	6
-0.73	FALSE	FALSE	NA	9
NA	FALSE	FALSE	NA	NA
0.07	FALSE	FALSE	NA	27
0.50	FALSE	FALSE	NA	32
0.25	FALSE	FALSE	NA	28
0.10	FALSE	FALSE	NA	19
0.40	FALSE	FALSE	NA	32
-1.96	FALSE	FALSE	NA	29
-0.04	FALSE	FALSE	NA	23
-0.53	FALSE	FALSE	NA	8
-0.42	FALSE	FALSE	NA	22
0.49	FALSE	FALSE	NA	7
0.32	FALSE	FALSE	NA	4
-0.74	FALSE	FALSE	NA	3
-0.32	FALSE	TRUE	NA	3
-2.58	FALSE	FALSE	NA	1
NA	FALSE	FALSE	NA	NA
-2.00	FALSE	FALSE	NA	2
-0.58	FALSE	FALSE	NA	3
-0.58	FALSE	FALSE	NA	3
0.49	FALSE	FALSE	NA	5
0.78	FALSE	FALSE	NA	11
1.00	FALSE	FALSE	NA	9
0.58	FALSE	FALSE	NA	6
0.00	FALSE	FALSE	NA	4
1.42	FALSE	FALSE	NA	4
0.58	FALSE	FALSE	NA	6
1.32	FALSE	FALSE	NA	4
0.74	FALSE	FALSE	NA	4
0.78	FALSE	FALSE	NA	7
0.74	FALSE	FALSE	NA	4
2.58	FALSE	FALSE	NA	5
0.00	FALSE	FALSE	NA	4

1.17	FALSE	FALSE	NA	6
-0.22	FALSE	FALSE	NA	5
1.58	FALSE	FALSE	NA	6
0.81	FALSE	TRUE	NA	5
1.22	FALSE	FALSE	NA	6
1.32	FALSE	FALSE	NA	3
1.58	FALSE	FALSE	NA	4
1.81	FALSE	FALSE	NA	6
1.00	FALSE	FALSE	NA	4
0.19	FALSE	FALSE	NA	5
0.47	FALSE	FALSE	NA	10
0.00	FALSE	FALSE	NA	5
1.32	FALSE	FALSE	NA	4
0.74	FALSE	FALSE	NA	5
-0.36	FALSE	FALSE	NA	7
1.32	FALSE	FALSE	NA	5
1.00	FALSE	FALSE	NA	7
0.26	FALSE	FALSE	NA	6
0.68	FALSE	FALSE	NA	6
0.15	FALSE	FALSE	NA	9
-0.13	FALSE	FALSE	NA	10
0.68	FALSE	FALSE	NA	12
-1.74	FALSE	FALSE	NA	3
-0.74	FALSE	FALSE	NA	3
0.00	FALSE	FALSE	NA	6
-0.61	FALSE	FALSE	NA	42
0.13	FALSE	FALSE	NA	11
0.73	FALSE	FALSE	NA	9
-2.58	FALSE	FALSE	NA	2
-1.81	FALSE	FALSE	NA	2
-1.74	FALSE	FALSE	NA	2
-0.40	FALSE	FALSE	NA	40
0.40	FALSE	FALSE	NA	22
NA	FALSE	FALSE	NA	NA
0.49	FALSE	FALSE	NA	42
0.07	FALSE	FALSE	NA	51
0.36	FALSE	FALSE	NA	15
1.02	FALSE	FALSE	NA	39
0.20	FALSE	FALSE	NA	41
-2.17	FALSE	FALSE	NA	2
NA	FALSE	FALSE	NA	NA
0.12	FALSE	FALSE	NA	103
1.58	FALSE	FALSE	NA	2
-0.71	FALSE	FALSE	NA	7
NA	FALSE	FALSE	NA	NA
0.38	FALSE	FALSE	NA	24
-2.12	FALSE	FALSE	NA	3
-1.58	FALSE	FALSE	NA	4
-2.91	FALSE	FALSE	NA	2
-1.42	FALSE	FALSE	NA	3
-1.22	FALSE	FALSE	NA	6
-0.29	FALSE	FALSE	NA	5
-0.36	FALSE	FALSE	NA	8
0.00	FALSE	FALSE	NA	6
NA	FALSE	FALSE	NA	NA

-0.27	FALSE	FALSE	NA	22
0.00	FALSE	FALSE	NA	9
0.42	FALSE	FALSE	NA	8
-0.87	FALSE	TRUE	NA	6
-1.81	FALSE	FALSE	NA	2
-0.06	FALSE	FALSE	NA	24
-0.74	FALSE	FALSE	NA	3
NA	FALSE	FALSE	NA	NA
-1.22	FALSE	FALSE	NA	3
-0.58	FALSE	FALSE	NA	27
0.00	FALSE	FALSE	NA	4
0.42	FALSE	FALSE	NA	4
-1.38	FALSE	FALSE	NA	4
-0.26	FALSE	FALSE	NA	116
2.70	FALSE	FALSE	NA	6
NA	FALSE	FALSE	NA	7
0.74	FALSE	TRUE	NA	5
0.81	FALSE	FALSE	NA	4
-2.00	FALSE	FALSE	NA	2
-1.70	FALSE	FALSE	NA	3
-1.00	FALSE	TRUE	NA	6
NA	FALSE	FALSE	NA	NA
0.80	FALSE	TRUE	NA	23
-2.46	FALSE	FALSE	NA	2
1.42	FALSE	TRUE	NA	8
-0.49	FALSE	TRUE	NA	4
-0.58	FALSE	FALSE	NA	4
0.12	FALSE	FALSE	NA	9
-0.71	FALSE	FALSE	NA	8
-1.32	FALSE	TRUE	NA	2
0.00	FALSE	FALSE	NA	14
0.30	FALSE	FALSE	NA	14
0.00	FALSE	TRUE	NA	15
1.00	FALSE	FALSE	NA	14
-1.58	FALSE	FALSE	NA	2
-2.58	FALSE	FALSE	NA	1
-1.32	FALSE	FALSE	NA	2
-0.58	FALSE	TRUE	NA	8
NA	FALSE	FALSE	NA	40
NA	FALSE	FALSE	NA	NA
0.42	FALSE	FALSE	NA	4
-0.42	FALSE	FALSE	NA	2
-0.68	FALSE	FALSE	NA	5
1.81	FALSE	FALSE	NA	7
0.42	FALSE	FALSE	NA	11
1.91	FALSE	FALSE	NA	14
-1.78	FALSE	FALSE	NA	22
0.74	FALSE	TRUE	NA	5
1.38	FALSE	FALSE	NA	7
-0.45	FALSE	FALSE	NA	7
0.00	FALSE	FALSE	NA	4
-0.83	FALSE	FALSE	NA	8
0.66	FALSE	FALSE	NA	31
0.27	FALSE	FALSE	NA	33
-0.49	FALSE	FALSE	NA	24

-0.56	FALSE	TRUE	NA	10
-1.22	FALSE	FALSE	NA	2
0.29	FALSE	FALSE	NA	8
-2.00	FALSE	FALSE	NA	2
2.66	FALSE	FALSE	NA	16
NA	FALSE	FALSE	NA	12
NA	FALSE	FALSE	NA	NA
NA	FALSE	FALSE	NA	NA
0.47	FALSE	TRUE	NA	24
0.44	FALSE	FALSE	NA	18
-0.25	FALSE	FALSE	NA	5
-0.20	FALSE	FALSE	NA	15
0.39	FALSE	FALSE	NA	9
3.46	FALSE	FALSE	NA	16
0.00	FALSE	FALSE	NA	7
0.81	FALSE	FALSE	NA	14
0.87	FALSE	FALSE	NA	14
-1.20	FALSE	FALSE	NA	9
-0.13	FALSE	FALSE	NA	25
1.48	FALSE	FALSE	NA	18
-0.32	FALSE	FALSE	NA	3
0.26	FALSE	FALSE	NA	5
NA	FALSE	FALSE	NA	NA
-0.14	FALSE	FALSE	NA	10
-0.86	FALSE	FALSE	NA	7
0.32	FALSE	FALSE	NA	6
1.32	FALSE	FALSE	NA	5
-1.32	FALSE	TRUE	NA	2
-3.00	FALSE	FALSE	NA	1
-0.72	FALSE	FALSE	NA	9
1.51	FALSE	FALSE	NA	25
-2.32	FALSE	FALSE	NA	2
-0.42	FALSE	TRUE	NA	3
1.49	FALSE	FALSE	NA	36
2.70	FALSE	FALSE	NA	13
0.00	FALSE	FALSE	NA	5
NA	FALSE	FALSE	NA	7
-1.79	FALSE	FALSE	NA	29
-1.77	FALSE	FALSE	NA	5
NA	FALSE	FALSE	NA	NA
1.58	FALSE	FALSE	NA	6
-0.32	FALSE	FALSE	NA	4
0.61	FALSE	TRUE	NA	31
-0.05	FALSE	TRUE	NA	24
-0.58	FALSE	TRUE	NA	4
-0.14	FALSE	TRUE	NA	7
0.81	FALSE	TRUE	NA	7
0.32	FALSE	TRUE	NA	4
-1.81	FALSE	TRUE	NA	2
0.76	FALSE	FALSE	NA	29
-2.12	FALSE	FALSE	NA	3
0.74	FALSE	TRUE	NA	8
2.58	FALSE	FALSE	NA	5
-0.22	FALSE	FALSE	NA	5
0.14	FALSE	FALSE	NA	15

0.15	FALSE	FALSE	NA	13
NA	FALSE	FALSE	NA	NA
NA	FALSE	FALSE	NA	NA
-0.26	FALSE	TRUE	NA	10
-0.26	FALSE	FALSE	NA	10
0.35	FALSE	TRUE	NA	27
0.15	FALSE	FALSE	NA	7
0.74	FALSE	TRUE	NA	9
2.00	FALSE	FALSE	NA	7
-0.22	FALSE	FALSE	NA	7
-0.38	FALSE	FALSE	NA	10
1.26	FALSE	TRUE	NA	15
-0.42	FALSE	FALSE	NA	3
-0.28	FALSE	FALSE	NA	9
-0.85	FALSE	FALSE	NA	5
-0.49	FALSE	FALSE	NA	16
0.31	FALSE	FALSE	NA	17
1.81	FALSE	FALSE	NA	10
3.17	FALSE	FALSE	NA	7
0.19	FALSE	FALSE	NA	4
0.15	FALSE	FALSE	NA	12
-0.32	FALSE	FALSE	NA	4
-0.42	FALSE	FALSE	NA	4
0.81	FALSE	FALSE	NA	23
-0.22	FALSE	TRUE	NA	5
-0.12	FALSE	FALSE	NA	22
0.12	FALSE	TRUE	NA	16
0.32	FALSE	FALSE	NA	5
1.00	TRUE	TRUE	NA	11
0.00	FALSE	TRUE	NA	9
1.58	FALSE	TRUE	NA	5
1.32	FALSE	TRUE	NA	8
0.00	FALSE	TRUE	NA	6
-0.36	FALSE	TRUE	NA	19
-0.45	FALSE	FALSE	NA	10
-0.45	FALSE	FALSE	NA	10
-0.33	FALSE	FALSE	NA	11
-0.35	FALSE	FALSE	NA	9
0.00	FALSE	FALSE	NA	12
-1.70	FALSE	FALSE	NA	4
-2.58	FALSE	FALSE	NA	5
0.15	FALSE	FALSE	1	1
0.15	FALSE	TRUE	NA	18
0.23	FALSE	FALSE	NA	11
0.70	FALSE	TRUE	NA	9
0.39	FALSE	TRUE	NA	11
0.11	FALSE	FALSE	NA	9
1.04	FALSE	TRUE	NA	13
0.62	FALSE	TRUE	NA	14
0.61	FALSE	TRUE	NA	12
0.55	FALSE	TRUE	NA	8
0.36	FALSE	FALSE	NA	9
0.75	FALSE	TRUE	NA	7
0.40	FALSE	TRUE	NA	16
0.36	FALSE	TRUE	NA	10

0.19	FALSE	TRUE	NA	11
0.48	FALSE	TRUE	NA	23
-0.22	FALSE	TRUE	NA	25
0.45	FALSE	TRUE	NA	22
0.35	FALSE	TRUE	NA	24
-0.43	FALSE	TRUE	NA	22
0.66	FALSE	TRUE	NA	20
0.46	FALSE	TRUE	NA	36
-1.58	FALSE	TRUE	NA	2
-2.19	FALSE	TRUE	NA	25
0.40	FALSE	TRUE	NA	18
0.47	FALSE	TRUE	NA	22
0.40	FALSE	TRUE	NA	14
0.32	FALSE	TRUE	NA	42
0.57	FALSE	TRUE	NA	32
0.87	FALSE	TRUE	NA	9
0.00	FALSE	TRUE	NA	10
0.68	FALSE	TRUE	NA	24
0.27	FALSE	TRUE	NA	12
0.47	FALSE	TRUE	NA	10
2.81	FALSE	TRUE	NA	4
1.32	FALSE	TRUE	NA	4
1.00	FALSE	TRUE	NA	7
1.32	FALSE	TRUE	NA	5
0.42	FALSE	FALSE	NA	4
0.45	FALSE	TRUE	NA	23
1.00	FALSE	FALSE	NA	13
-1.25	FALSE	FALSE	NA	6
-4.00	FALSE	FALSE	NA	1
-1.81	FALSE	FALSE	NA	2
1.58	FALSE	FALSE	NA	4
0.36	FALSE	TRUE	NA	14
-1.42	FALSE	FALSE	NA	3
-1.38	FALSE	FALSE	NA	3
0.00	FALSE	FALSE	NA	7
0.42	FALSE	FALSE	NA	15
-0.17	FALSE	TRUE	NA	8
-0.81	FALSE	FALSE	NA	3
0.06	FALSE	FALSE	NA	16
-0.32	FALSE	FALSE	NA	19
-0.58	FALSE	TRUE	NA	4
0.06	FALSE	FALSE	NA	7
0.16	FALSE	FALSE	NA	8
-1.14	FALSE	FALSE	NA	5
-1.14	FALSE	FALSE	NA	5
NA	FALSE	FALSE	NA	NA
0.07	FALSE	FALSE	NA	8
-1.58	FALSE	FALSE	NA	2
0.07	FALSE	FALSE	NA	11
-0.49	FALSE	FALSE	NA	2
-1.58	FALSE	FALSE	NA	3
-0.45	FALSE	FALSE	NA	4
NA	FALSE	FALSE	NA	NA
-2.70	FALSE	FALSE	NA	2
-0.26	FALSE	FALSE	NA	4

0.00	FALSE	FALSE	NA	4
0.64	FALSE	FALSE	NA	10
-0.53	FALSE	FALSE	NA	10
-1.08	FALSE	FALSE	NA	7
0.16	FALSE	FALSE	NA	9
0.10	FALSE	FALSE	NA	3
0.42	FALSE	FALSE	NA	4
-1.32	FALSE	FALSE	NA	2
-1.32	FALSE	FALSE	NA	1
0.18	FALSE	TRUE	NA	12
0.85	FALSE	FALSE	NA	18
-1.74	FALSE	FALSE	NA	3
-2.17	FALSE	FALSE	NA	6
-0.87	FALSE	FALSE	NA	6
-0.74	FALSE	FALSE	NA	6
-0.06	FALSE	FALSE	NA	28
0.00	FALSE	FALSE	NA	4
NA	FALSE	FALSE	NA	NA
1.15	FALSE	FALSE	NA	14
0.36	FALSE	FALSE	NA	12
-0.49	FALSE	FALSE	NA	7
0.15	FALSE	TRUE	NA	6
0.00	FALSE	FALSE	NA	5
0.74	FALSE	FALSE	NA	15
0.84	FALSE	FALSE	NA	26
2.00	FALSE	FALSE	NA	7
0.00	FALSE	FALSE	NA	10
1.32	FALSE	FALSE	NA	4
-2.00	FALSE	FALSE	NA	2
-0.70	FALSE	FALSE	NA	30
-1.32	TRUE	FALSE	NA	2
-0.13	FALSE	FALSE	NA	9
-0.68	FALSE	FALSE	NA	5
-0.36	FALSE	FALSE	NA	6
-0.65	FALSE	FALSE	NA	7
-0.68	TRUE	FALSE	NA	2
0.30	FALSE	TRUE	NA	13
0.00	FALSE	TRUE	NA	5
0.32	FALSE	FALSE	NA	17
0.10	FALSE	FALSE	NA	7
-0.42	FALSE	FALSE	NA	2
0.42	FALSE	FALSE	NA	4
-1.37	FALSE	FALSE	NA	4
-1.12	FALSE	TRUE	NA	6
-0.36	FALSE	TRUE	NA	12
-1.06	FALSE	FALSE	NA	6
-1.28	FALSE	FALSE	NA	6
-0.67	FALSE	TRUE	NA	8
-0.54	FALSE	FALSE	NA	10
-1.05	FALSE	FALSE	NA	7
-1.81	FALSE	TRUE	NA	4
-1.05	FALSE	TRUE	NA	7
-1.70	FALSE	TRUE	NA	3
-0.56	FALSE	FALSE	NA	9
-0.64	FALSE	TRUE	NA	6

-0.55	FALSE	FALSE	NA	6
-0.94	FALSE	TRUE	NA	8
-0.57	FALSE	FALSE	NA	16
-0.74	FALSE	FALSE	NA	5
-0.45	FALSE	TRUE	NA	19
-0.42	FALSE	FALSE	NA	8
-0.62	FALSE	TRUE	NA	7
-1.17	FALSE	FALSE	NA	4
-1.62	FALSE	TRUE	NA	15
-0.35	FALSE	TRUE	NA	11
-1.32	FALSE	TRUE	NA	6
-1.22	FALSE	TRUE	NA	4
-0.74	FALSE	TRUE	NA	5
-0.87	FALSE	TRUE	NA	4
-0.68	FALSE	FALSE	NA	5
-0.42	FALSE	FALSE	NA	3
-0.68	FALSE	FALSE	NA	5
-0.51	FALSE	TRUE	NA	2
-0.86	FALSE	TRUE	NA	3
-1.34	FALSE	TRUE	NA	12
-0.39	FALSE	TRUE	NA	20
-0.84	FALSE	FALSE	NA	16
-2.19	FALSE	TRUE	NA	8
-1.31	FALSE	FALSE	NA	12
NA	FALSE	FALSE	NA	11
-0.45	FALSE	TRUE	1	14
-1.64	FALSE	FALSE	NA	9
1.05	FALSE	FALSE	NA	21
-1.81	FALSE	FALSE	NA	3
-1.87	FALSE	TRUE	NA	4
-2.36	FALSE	FALSE	NA	7
-3.70	FALSE	FALSE	NA	2
-0.19	FALSE	TRUE	NA	5
-0.60	FALSE	FALSE	NA	11
-0.80	FALSE	TRUE	NA	10
-0.87	FALSE	TRUE	NA	9
0.19	FALSE	TRUE	NA	7
-0.51	FALSE	TRUE	NA	7
-0.88	FALSE	TRUE	NA	4
-1.47	FALSE	TRUE	NA	9
-0.39	FALSE	TRUE	NA	15
-0.38	FALSE	FALSE	NA	13
-0.83	FALSE	TRUE	NA	21
-0.56	FALSE	TRUE	NA	15
-0.26	FALSE	FALSE	NA	11
0.16	FALSE	TRUE	NA	6
-0.34	FALSE	TRUE	NA	4
-0.50	FALSE	TRUE	NA	5
-0.56	FALSE	TRUE	NA	4
-1.35	FALSE	FALSE	NA	6
-0.68	FALSE	TRUE	NA	5
-0.16	FALSE	TRUE	NA	10
0.40	FALSE	FALSE	NA	7
0.42	FALSE	FALSE	NA	2
-1.87	FALSE	TRUE	NA	3

-0.39	FALSE	TRUE	NA	26
-0.47	FALSE	FALSE	NA	23
-1.41	FALSE	TRUE	NA	21
-0.11	FALSE	TRUE	NA	13
-0.34	FALSE	TRUE	NA	8
NA	FALSE	FALSE	NA	NA
-0.51	FALSE	FALSE	NA	14
-0.53	FALSE	TRUE	NA	12
-0.82	FALSE	TRUE	NA	13
0.00	FALSE	FALSE	NA	18
0.00	FALSE	FALSE	NA	18
-1.32	FALSE	TRUE	NA	2
0.81	FALSE	FALSE	NA	4
1.22	FALSE	TRUE	NA	6
-1.82	FALSE	FALSE	NA	12
-0.85	FALSE	FALSE	NA	4
-2.58	FALSE	FALSE	NA	1
-0.38	FALSE	FALSE	NA	8
-0.07	FALSE	FALSE	NA	24
-0.17	FALSE	FALSE	NA	11
-0.42	FALSE	FALSE	NA	2
-2.06	TRUE	FALSE	NA	14
0.49	FALSE	FALSE	NA	22
0.49	FALSE	FALSE	NA	28
2.00	FALSE	FALSE	NA	8
3.46	FALSE	FALSE	NA	8
NA	FALSE	FALSE	NA	8
0.32	FALSE	FALSE	NA	3
-0.42	FALSE	FALSE	NA	2
-0.28	FALSE	TRUE	NA	21
0.40	FALSE	TRUE	NA	34
0.15	FALSE	TRUE	NA	12
NA	FALSE	FALSE	NA	NA
-3.00	FALSE	FALSE	NA	1
-0.36	FALSE	FALSE	NA	7
-0.26	FALSE	FALSE	NA	10
-0.49	FALSE	FALSE	NA	5
-0.81	FALSE	FALSE	NA	4
0.64	FALSE	FALSE	NA	10
-2.12	FALSE	FALSE	NA	5
-0.49	FALSE	FALSE	NA	11
-1.00	FALSE	FALSE	NA	6
NA	FALSE	FALSE	NA	NA
0.22	FALSE	FALSE	NA	12
-1.22	FALSE	FALSE	NA	5
-0.74	FALSE	FALSE	NA	2
0.26	FALSE	FALSE	NA	4
0.26	FALSE	FALSE	NA	3
0.35	FALSE	FALSE	NA	14
-0.95	FALSE	FALSE	NA	17
0.28	FALSE	FALSE	NA	15
-1.50	FALSE	FALSE	NA	18
-0.28	FALSE	TRUE	NA	22
1.58	TRUE	FALSE	NA	12
-0.03	FALSE	TRUE	NA	10

-0.42	FALSE	FALSE	NA	3
1.58	FALSE	TRUE	NA	17
1.58	FALSE	TRUE	NA	9
0.82	FALSE	TRUE	NA	39
0.62	FALSE	TRUE	NA	18
1.62	FALSE	TRUE	NA	47
0.42	FALSE	TRUE	NA	29
-1.58	FALSE	FALSE	NA	5
NA	FALSE	FALSE	NA	NA
0.37	FALSE	FALSE	NA	41
-0.35	FALSE	TRUE	NA	11
0.42	FALSE	FALSE	NA	6
-3.17	FALSE	FALSE	NA	1
-1.81	FALSE	FALSE	NA	1
-0.90	FALSE	FALSE	NA	11
-0.91	FALSE	FALSE	NA	25
3.81	FALSE	TRUE	NA	13
-1.00	FALSE	FALSE	NA	11
2.66	FALSE	FALSE	NA	19
-0.63	FALSE	FALSE	NA	11
NA	FALSE	FALSE	NA	7
0.58	FALSE	FALSE	NA	17
0.42	FALSE	FALSE	NA	7
0.00	FALSE	TRUE	NA	4
-0.51	FALSE	FALSE	NA	40
0.23	FALSE	TRUE	NA	49
0.00	FALSE	TRUE	NA	5
0.69	FALSE	FALSE	NA	10
0.39	FALSE	TRUE	NA	11
0.30	FALSE	TRUE	NA	9
0.22	FALSE	TRUE	NA	5
0.24	FALSE	TRUE	NA	7
0.09	FALSE	TRUE	NA	11
0.42	FALSE	TRUE	NA	5
0.42	FALSE	TRUE	NA	1
0.00	FALSE	TRUE	NA	2
0.30	FALSE	TRUE	NA	9
0.00	FALSE	FALSE	NA	3
NA	FALSE	FALSE	NA	NA
-0.74	FALSE	FALSE	NA	2
NA	FALSE	FALSE	NA	NA
NA	FALSE	FALSE	NA	NA
-0.81	FALSE	FALSE	NA	3
0.15	FALSE	FALSE	NA	91
1.44	FALSE	FALSE	NA	14
NA	FALSE	FALSE	NA	91
0.60	FALSE	FALSE	NA	91
-0.68	FALSE	FALSE	NA	4
-1.00	FALSE	FALSE	NA	2
-0.74	FALSE	FALSE	NA	2
-0.32	FALSE	FALSE	NA	3
-1.26	FALSE	FALSE	NA	5
-1.58	FALSE	FALSE	NA	4
-1.00	FALSE	FALSE	NA	8
-1.17	FALSE	FALSE	NA	4

-2.00	FALSE	TRUE	NA	2
NA	FALSE	FALSE	NA	NA
-0.10	FALSE	TRUE	NA	12
1.00	FALSE	TRUE	NA	22
0.14	FALSE	FALSE	NA	3
0.56	FALSE	TRUE	NA	11
-0.87	FALSE	TRUE	NA	3
0.00	FALSE	TRUE	NA	8
NA	FALSE	TRUE	NA	NA
0.71	FALSE	TRUE	NA	4
0.38	FALSE	TRUE	NA	4
0.31	FALSE	TRUE	NA	20
0.51	FALSE	FALSE	NA	6
NA	FALSE	FALSE	NA	NA
NA	FALSE	FALSE	NA	NA
-1.00	FALSE	FALSE	NA	3
-1.32	FALSE	FALSE	NA	4
0.58	FALSE	FALSE	NA	6
0.16	FALSE	FALSE	NA	33
-0.58	FALSE	FALSE	NA	4
-0.54	FALSE	TRUE	NA	13
-0.85	FALSE	FALSE	NA	5
0.00	FALSE	FALSE	NA	5
-0.91	FALSE	FALSE	NA	7
NA	FALSE	FALSE	NA	7
2.00	FALSE	FALSE	NA	10
1.49	FALSE	FALSE	NA	10
0.93	FALSE	TRUE	NA	26
0.00	FALSE	FALSE	NA	10
-0.42	FALSE	FALSE	NA	3
1.48	FALSE	TRUE	NA	25
0.84	FALSE	FALSE	NA	7
0.05	FALSE	TRUE	NA	15
-0.27	FALSE	FALSE	NA	29
0.00	FALSE	FALSE	NA	5
-1.66	FALSE	FALSE	NA	5
-1.18	FALSE	FALSE	NA	13
-1.22	FALSE	FALSE	NA	2
-0.34	FALSE	FALSE	NA	31
NA	FALSE	FALSE	NA	NA
-0.29	FALSE	FALSE	NA	7
NA	FALSE	FALSE	NA	NA
0.42	FALSE	FALSE	NA	4
0.49	FALSE	FALSE	NA	7
0.00	FALSE	FALSE	NA	7
-0.25	FALSE	TRUE	NA	1
-0.42	FALSE	FALSE	NA	8
-0.42	FALSE	FALSE	NA	3
-1.58	FALSE	FALSE	NA	6
0.32	FALSE	FALSE	NA	5
2.58	FALSE	FALSE	NA	20
-0.49	FALSE	FALSE	NA	4
-1.36	FALSE	FALSE	NA	3
-1.58	FALSE	FALSE	NA	1
-1.14	FALSE	FALSE	NA	3

1.58	FALSE	FALSE	NA	7
NA	FALSE	FALSE	NA	NA
-0.58	FALSE	FALSE	NA	4
NA	FALSE	FALSE	NA	9
-1.08	FALSE	FALSE	NA	1
-0.91	FALSE	FALSE	NA	4
-0.48	FALSE	TRUE	NA	24
-0.53	FALSE	FALSE	NA	6
0.00	FALSE	FALSE	NA	6
-1.93	FALSE	FALSE	NA	4
-0.81	FALSE	FALSE	NA	4
NA	FALSE	FALSE	NA	10
NA	FALSE	FALSE	NA	10
NA	FALSE	FALSE	NA	7
0.19	FALSE	FALSE	NA	6
-1.00	FALSE	FALSE	NA	5
-0.91	FALSE	FALSE	NA	7
-1.72	FALSE	FALSE	NA	6
-1.37	FALSE	FALSE	NA	17
1.89	FALSE	FALSE	NA	25
-2.00	FALSE	FALSE	NA	3
-0.32	FALSE	TRUE	NA	4
1.45	FALSE	FALSE	NA	21
NA	FALSE	FALSE	NA	NA
0.50	FALSE	FALSE	NA	30
0.71	TRUE	TRUE	1	5
0.00	FALSE	TRUE	NA	5
0.74	FALSE	TRUE	NA	4
0.46	FALSE	TRUE	NA	10
1.22	FALSE	TRUE	NA	7
0.62	FALSE	TRUE	NA	14
0.39	FALSE	FALSE	NA	14
NA	FALSE	FALSE	NA	7
NA	FALSE	FALSE	NA	NA
0.05	FALSE	FALSE	1	1
-1.32	FALSE	TRUE	NA	2
1.58	FALSE	FALSE	NA	5
-1.70	FALSE	FALSE	NA	4
1.00	FALSE	FALSE	NA	5
-0.42	FALSE	FALSE	NA	7
-0.24	FALSE	FALSE	3	43
NA	FALSE	FALSE	3	14
-0.24	FALSE	FALSE	NA	20
0.13	FALSE	FALSE	NA	4
-0.06	FALSE	FALSE	NA	7
0.10	FALSE	FALSE	3	43
0.09	FALSE	FALSE	3	43
-0.01	FALSE	FALSE	NA	21
-0.03	FALSE	FALSE	NA	21
0.04	FALSE	FALSE	NA	43
0.02	FALSE	FALSE	NA	45
0.03	FALSE	FALSE	NA	22
-0.85	FALSE	FALSE	NA	4
0.95	FALSE	FALSE	NA	24
-0.42	FALSE	FALSE	NA	2

NA	FALSE	FALSE	NA	NA
0.00	FALSE	FALSE	NA	6
0.00	FALSE	FALSE	NA	6
0.57	FALSE	FALSE	NA	19
0.29	FALSE	TRUE	NA	10
0.00	FALSE	TRUE	NA	13
0.47	FALSE	FALSE	NA	16
3.58	FALSE	FALSE	NA	9
1.00	FALSE	FALSE	NA	2
-3.58	FALSE	FALSE	NA	1
-2.21	FALSE	FALSE	NA	5
1.00	FALSE	FALSE	NA	2
1.00	FALSE	TRUE	NA	2
1.32	FALSE	FALSE	NA	5
-1.32	FALSE	FALSE	NA	2
0.81	FALSE	FALSE	NA	6
0.65	FALSE	FALSE	NA	10
-1.17	FALSE	FALSE	NA	4
-0.74	FALSE	FALSE	NA	3
1.58	FALSE	FALSE	NA	7
-1.32	FALSE	FALSE	NA	2
0.30	FALSE	FALSE	NA	17
-2.58	FALSE	FALSE	NA	1
0.65	FALSE	FALSE	NA	11
-1.32	FALSE	FALSE	NA	2
-0.13	FALSE	FALSE	NA	36
-0.11	FALSE	FALSE	NA	12
0.14	FALSE	FALSE	NA	9
-0.42	FALSE	FALSE	NA	3
0.07	FALSE	FALSE	NA	15
-0.32	FALSE	FALSE	NA	3
0.12	FALSE	FALSE	NA	45
2.46	FALSE	FALSE	NA	9
0.85	FALSE	FALSE	NA	8
-0.78	FALSE	TRUE	NA	35
-1.38	FALSE	FALSE	NA	5
0.53	FALSE	FALSE	NA	8
0.00	FALSE	FALSE	NA	14
-0.39	FALSE	FALSE	NA	22
NA	FALSE	FALSE	NA	NA
-1.81	FALSE	FALSE	NA	2
1.32	FALSE	FALSE	NA	5
-0.18	FALSE	FALSE	NA	21
0.07	FALSE	FALSE	NA	26
1.20	FALSE	FALSE	NA	19
0.09	FALSE	FALSE	NA	41
NA	FALSE	FALSE	NA	NA
-1.17	FALSE	FALSE	NA	4
-2.25	FALSE	FALSE	NA	3
-2.22	FALSE	FALSE	NA	1
-0.53	FALSE	FALSE	NA	21
-1.00	FALSE	FALSE	NA	5
NA	FALSE	FALSE	NA	10
-0.25	FALSE	FALSE	NA	71
0.08	FALSE	FALSE	NA	18

0.30	FALSE	FALSE	NA	22
0.00	FALSE	FALSE	NA	13
0.13	FALSE	FALSE	NA	8
-0.29	FALSE	FALSE	NA	28
NA	FALSE	FALSE	NA	1
0.42	FALSE	FALSE	NA	4
0.15	FALSE	FALSE	NA	7
-0.58	FALSE	FALSE	NA	13
-1.00	FALSE	FALSE	NA	3
0.00	FALSE	FALSE	NA	8
-1.32	FALSE	FALSE	NA	2
1.70	FALSE	FALSE	NA	9
NA	FALSE	FALSE	NA	9
0.11	FALSE	FALSE	NA	11
-0.21	FALSE	TRUE	NA	8
0.00	FALSE	FALSE	NA	9
-1.81	FALSE	TRUE	NA	2
1.00	FALSE	TRUE	NA	20
0.51	FALSE	FALSE	NA	9
1.00	TRUE	FALSE	NA	12
1.26	TRUE	FALSE	NA	11
0.00	FALSE	TRUE	NA	3
-0.91	FALSE	FALSE	NA	16
-1.15	FALSE	TRUE	NA	18
-1.22	FALSE	FALSE	NA	6
-1.00	FALSE	FALSE	NA	3
NA	FALSE	FALSE	NA	NA
-0.70	FALSE	FALSE	NA	6
-0.38	FALSE	FALSE	NA	8
-0.08	FALSE	TRUE	NA	22
0.06	FALSE	FALSE	NA	29
-0.21	FALSE	FALSE	NA	17
-0.15	FALSE	FALSE	NA	18
-0.16	FALSE	FALSE	NA	25
0.02	FALSE	TRUE	NA	24
-0.74	FALSE	FALSE	NA	3
-1.00	FALSE	FALSE	NA	4
1.22	FALSE	FALSE	NA	7

Trim32 enriched Proteins

Unique peptide count proliferating NSC	Protein coverage (%) control	Protein coverage (%) Differentiating NSC	Protein coverage (%) proliferating NSC
5	NA	26	43.1
2	NA	8.1	8.1
3	NA	10.6	20.7
13	NA	36.5	63.9
1	NA	9.3	4.7
5	NA	34.9	34.9
9	NA	17.5	24
5	NA	NA	11.8
19	NA	43.2	37.4
13	NA	9.8	22
14	NA	9.4	26.4
11	NA	54.4	47.4
34	NA	15.1	19.6
NA	NA	18.6	NA
1	NA	7.9	2.8
6	NA	34.2	23.3
2	NA	24.8	7.4
5	NA	23.5	9.9
26	NA	40.1	51.2
3	NA	7.4	7.8
29	NA	20.8	32.7
2	NA	13.6	2.2
17	NA	40.7	36.8
6	NA	10.7	11.8
8	NA	27.9	24.9
18	NA	NA	49.6
12	16.8	70.9	45.3
11	NA	26.1	35.4
8	NA	46.7	24.9
38	16.8	70.9	79.2
43	NA	67.5	61.7
55	NA	67.3	71.5
10	NA	52.7	38.6
11	NA	44.1	47.1
11	NA	19.8	35
9	NA	20.8	33.7
5	NA	15	15
3	NA	5.2	4.3
5	NA	19.9	12.7
10	NA	NA	5.3
11	NA	48.2	38.5
11	NA	15.2	7.7
16	NA	71.3	74.5
8	NA	44.7	35.3
5	NA	10.6	20.3
NA	NA	39.2	NA
2	NA	14.3	9.4
8	NA	13	16.9
2	NA	4.4	4.4

10	NA	18.9	18.4
25	NA	49.2	77.7
16	NA	27.8	54
5	NA	25.5	35.3
24	NA	25.8	15.9
3	NA	8.5	4.7
3	NA	15.7	5
12	NA	28.8	20.9
24	NA	11.3	8.9
24	NA	26.3	29.2
8	NA	5.3	5
4	NA	1.2	6
11	NA	36	30
9	NA	28.3	28.3
9	NA	33.1	45
8	NA	10.9	33.6
14	NA	12.6	17.7
18	NA	12.2	22.3
11	NA	9.8	12.3
21	NA	22.1	30
5	NA	21.6	11.7
6	NA	25.8	8.7
14	NA	57.4	46.1
12	NA	13.1	27.8
19	NA	26.2	41.7
5	NA	35.9	35.9
5	NA	35.9	35.9
5	NA	31.7	39.4
6	NA	41.1	45
5	NA	16.6	10
8	NA	7.1	13.3
33	NA	18.2	43.6
6	NA	48.9	43.5
11	NA	41.7	44.7
5	NA	30.9	30.9
6	NA	17.9	41.1
3	NA	10.6	15.6
3	NA	16.7	16.7
7	NA	10.1	23.6
7	NA	NA	13.4
11	NA	21.1	25.5
15	NA	NA	17.2
30	NA	43.9	52.6
29	NA	51.6	70.7
7	NA	7.4	26.6
5	NA	7	19.1
10	NA	NA	68.3
9	NA	28.6	55.4
5	NA	5.8	10
4	NA	16.4	12.1
11	NA	29.7	29.5
6	NA	NA	6.3
12	NA	4.9	15.7
5	NA	26.4	26.4
3	NA	15.9	8.4

14	NA	13.5	20.6
5	NA	34.9	35.4
NA	NA	12.2	NA
11	NA	15.1	33.5
NA	NA	19.5	NA
3	NA	8.5	9.4
38	NA	4.3	12.2
3	NA	22.3	11.7
2	NA	6.3	7.3
7	NA	37.7	52
13	NA	35.9	38.3
7	NA	3.4	19.3
7	NA	12.6	13.8
8	NA	10.7	18.9
9	NA	14.7	46.3
5	NA	15.1	22.2
13	NA	31	63.3
72	NA	39.5	46.5
19	NA	3.5	27.2
9	NA	29.5	73.8
19	NA	51.4	48.3
13	NA	47.9	48.3
8	NA	6.3	19.1
27	NA	30.1	28
15	NA	7.4	15.3
13	NA	20.6	26.9
4	NA	20	11.2
13	NA	39.4	27.1
7	NA	21.9	59.8
13	NA	28.7	32.1
8	NA	30.2	40
6	NA	39.9	25.5
15	NA	47.3	47.7
12	NA	17.6	18.1
4	NA	32.6	21.2
4	NA	19.7	23.5
1	NA	5	1.5
28	NA	47.5	46.4
7	NA	24.9	28.6
2	NA	1.8	3.6
4	NA	13	9.2
5	NA	5.6	3.3
6	NA	NA	30.5
29	NA	69	67.9
28	NA	48.4	56.1
25	NA	57.5	59.4
28	NA	60.1	62.7
20	NA	46.7	50.7
24	NA	61.9	57
30	NA	62.4	62.4
19	NA	7.3	17.5
8	NA	25.8	16.3
8	NA	31	16.8
6	NA	17.7	23
3	NA	15.7	21.5

4	NA	5.9	5.4
12	NA	9.8	44.1
4	NA	NA	14.2
12	NA	29.7	49.5
4	NA	11.3	11.8
8	NA	39.9	43.4
11	NA	57.8	57.8
4	NA	44	31.9
3	NA	5.2	16.9
6	NA	33.1	33.1
18	NA	2.1	38.6
11	NA	NA	6.4
29	NA	69.3	70.6
7	NA	NA	6.7
7	NA	NA	7
6	NA	15.8	27.8
4	NA	16.5	22.1
2	NA	10.6	3.9
3	NA	21.8	17.6
77	NA	59.9	59.9
14	NA	9.7	13
6	NA	27	43.8
15	NA	54.5	75.5
20	NA	3.1	9.7
NA	NA	13.8	NA
9	NA	NA	17.2
3	NA	8	12.1
44	NA	25.7	45.8
27	NA	23.6	40.6
15	NA	19.9	22.7
11	NA	35.4	59.7
26	NA	24.1	40.1
26	NA	24.8	41.3
6	NA	3.6	6.8
3	NA	34.7	34.7
8	NA	26	21.9
7	NA	16.5	21.7
8	NA	18.7	24.6
8	NA	21	28.1
9	NA	41.7	34.3
7	NA	28	34.9
5	NA	41.6	41.6
3	NA	22.6	22.6
14	NA	46.4	34.4
3	NA	4.9	6.4
3	NA	33.7	33.7
19	NA	49.4	42.5
22	NA	24.9	32
20	NA	34.6	22.7
6	NA	20.5	24.3
14	NA	42.7	42.7
9	NA	29.4	35.7
8	NA	38.6	49.3
8	NA	7.4	10.9
6	NA	6.5	9.3

19	NA	NA	28
9	NA	18.6	19.3
6	NA	10.4	5.5
18	NA	32.4	34.1
12	NA	8.6	19.1
5	NA	9.5	7.5
5	NA	14.1	6.6
5	NA	9.6	6.5
4	NA	6.2	4.4
10	NA	1.2	6.6
12	NA	33.6	46.5
5	NA	NA	24.9
7	NA	30.5	48.6
10	NA	2.7	9.4
11	NA	NA	26.4
6	NA	16.2	19.7
23	NA	63.1	53.1
8	NA	11.3	15.3
5	NA	13.2	12
10	NA	42.7	29.6
4	NA	12.5	8.6
9	NA	9.1	38.5
27	NA	1.1	29.5
11	NA	37.1	34.8
3	NA	16.8	22.1
24	NA	41.6	25.9
14	NA	2	49
23	NA	25.5	41.4
18	NA	32.5	26.3
3	NA	14.4	8.6
5	NA	19.7	7.3
NA	NA	21.8	NA
15	NA	41.8	41.4
34	NA	47.6	56.3
29	NA	43.6	48.7
15	NA	28.4	48.4
5	NA	23	17.6
4	NA	15.1	22.2
6	NA	10.1	10.6
10	NA	7.9	10.2
7	NA	0.9	7.8
10	NA	2.4	13.8
1	NA	6	1
46	NA	33.7	34.5
8	NA	6.7	26.2
3	NA	8.2	4
6	NA	8.6	19.8
3	NA	5	7.8
8	NA	30.8	21.4
6	NA	14.3	14.3
13	NA	43.1	49.9
12	NA	40.3	48.5
6	NA	NA	26.3
11	NA	10.3	18.4
3	NA	13.5	9.7

11	NA	24.5	26.5
5	NA	8.7	24.2
6	NA	8.4	10.4
6	NA	NA	9.9
17	NA	1.7	10.2
NA	NA	20.6	NA
7	NA	13.9	27.5
12	NA	27.7	39.6
11	NA	0.5	1.7
3	NA	24.2	24.2
2	NA	14.8	14.8
134	NA	25.7	35
5	NA	15.2	6.8
5	NA	5	13.8
2	NA	8.7	5.5
3	NA	49.4	49.4
3	NA	49.4	49.4
4	NA	22.3	15.9
9	NA	42.1	33.1
4	NA	21.2	15.1
7	NA	2.5	9.3
2	NA	15.5	15.5
34	2.2	63.6	65.2
6	NA	12.4	45.3
2	NA	38.9	13
5	NA	54.6	43.7
12	NA	27.9	32.3
34	NA	34.3	45.5
NA	NA	10.9	NA
25	NA	32.8	36.3
1	NA	5	0.9
4	NA	59.3	59.3
5	NA	33.3	33.3
6	NA	36.1	43.1
15	NA	28.2	38.4
9	NA	31.1	39
7	NA	17.4	23.9
9	NA	17.4	26.9
23	NA	66.7	69.8
15	NA	40.8	51.4
18	NA	44.3	46.4
63	NA	45.5	45.7
35	NA	50.2	52.8
30	NA	27.8	34.6
17	NA	42.3	40.7
20	NA	43.4	52.6
10	NA	44	36.3
12	NA	48.1	51.9
22	NA	75.6	68.8
14	NA	71.4	63.1
10	NA	37.2	29.5
10	NA	36.9	29.3
7	NA	45.4	45.4
25	NA	53.7	44.7
10	NA	32.9	35.3

19	NA	55.4	55.4
12	NA	32.4	34.9
13	NA	21.9	33.1
15	NA	24.2	27.8
8	NA	25.3	36.9
40	NA	19.6	28
22	NA	24.9	32.9
5	NA	1.5	3.6
6	NA	10.9	32.7
12	NA	22.1	31.9
13	NA	53.2	53.2
16	NA	5.6	18.9
7	NA	31.4	52.2
7	NA	8.9	12.8
16	NA	44.8	47.9
2	NA	10.8	2.3
10	NA	30.9	23.2
4	NA	34.7	17.3
6	NA	18.8	35
4	NA	4	17.5
7	NA	NA	39
4	NA	10.1	25.1
10	NA	NA	59.5
6	NA	6.1	7.8
8	NA	24.4	29.7
19	NA	17	47
71	NA	43.3	55
4	NA	21.6	5.1
5	NA	3.2	4.1
7	NA	3.4	30.3
12	NA	3.5	45
16	NA	42.1	44.6
2	NA	24.9	11.1
12	NA	13.3	16
3	NA	32.8	21.5
7	NA	42.3	32.1
4	NA	20	18.8
6	NA	23.8	33.6
4	NA	20.1	24.2
5	NA	27.9	27.9
3	NA	29.5	29.5
5	NA	7.2	7.4
27	NA	25.3	33.1
4	NA	7.8	4.4
5	NA	14.5	18.3
4	NA	19.1	12.4
17	NA	22.6	42.3
21	NA	20	35.1
30	NA	28.4	16.9
7	NA	27.8	28.7
16	NA	8.7	16.9
NA	NA	32.7	NA
6	NA	20.3	20.3
2	NA	9.8	9.8
13	NA	55.4	58.5

25	NA	61.6	56.6
11	NA	22.6	23.2
20	NA	9.8	19.4
102	NA	45.3	54.2
46	NA	15.8	24.9
6	NA	3.9	13.5
11	NA	7	20.8
9	NA	NA	3.5
10	NA	NA	27.8
6	NA	27.3	36.1
23	NA	32.9	29.8
22	NA	46.4	38.3
20	NA	22.6	22.6
15	NA	4.7	26.6
10	NA	NA	20
18	NA	55.1	48.8
10	NA	18.3	25.1
4	NA	NA	32.5
46	NA	58.7	59.6
27	6.6	58.9	55.9
5	NA	5.3	4.5
2	NA	7.8	7.8
4	NA	19.6	7.5
8	NA	14.2	39.9
61	NA	5.4	29.5
6	NA	3.8	25.5
15	NA	2.9	11.6
2	NA	8.3	8.3
5	NA	39.5	32.5
4	NA	13.6	7.7
14	NA	3.7	14
7	NA	3.9	24.6
13	NA	48.2	24
9	NA	40.2	43.6
12	NA	37.6	39.3
1	NA	14	14
3	NA	10.6	8.3
12	NA	21.5	23.9
21	NA	66.7	64
3	NA	NA	24.3
10	NA	67.1	67.1
13	NA	51.9	51.9
4	NA	24.3	39.8
6	NA	21.1	20.3
12	NA	NA	31.6
NA	NA	16.5	NA
7	NA	34.3	34.9
9	NA	34.8	44.5
10	NA	54.5	54.5
3	NA	21.3	16.9
16	6.7	NA	56.7
NA	NA	32.9	NA
1	NA	14.1	7
8	NA	38.8	44.8
18	NA	40.2	43.8

4	NA	25.3	25.3
5	NA	13.8	26.8
27	6.7	59.8	56.7
9	NA	37.2	40.4
16	6.7	NA	56.7
4	NA	29.5	29.5
3	NA	21.8	17.2
NA	NA	28.7	NA
3	NA	NA	25.4
11	6.7	NA	44.6
NA	NA	21.9	NA
36	NA	39.1	39.3
5	NA	28.8	28.8
3	NA	37.8	24.1
5	NA	21.6	30.8
8	NA	27.6	27.6
10	NA	41.8	35.9
6	NA	29.5	34.3
10	NA	30.9	33.6
4	NA	16.4	29.8
5	NA	22.3	31.8
1	NA	13	6.5
18	NA	41.4	45.1
10	NA	42	36
4	NA	5.6	5.1
13	NA	39.4	32.5
7	NA	13.4	15.9
8	NA	40.5	40.5
8	NA	28.3	15.6
9	NA	22.3	33
7	NA	9.7	25.9
6	NA	NA	20.6
19	NA	78.9	77.9
8	NA	NA	15.7
8	NA	8.4	22.9
10	NA	23	23.2
NA	NA	19.9	NA
3	NA	19.4	19.4
7	NA	16.1	36.7
5	NA	24.3	35.2
9	NA	25.6	37
3	NA	22.7	9.1
7	NA	26.7	31.8
6	NA	15.2	16.9
4	NA	8.8	13.7
NA	NA	8.4	NA
4	NA	5.5	7.3
NA	NA	16.5	NA
4	NA	NA	49.6
NA	NA	31.2	NA
NA	NA	52.4	NA
NA	NA	31.2	NA
4	NA	42.5	21.6
NA	NA	16.9	NA
7	NA	29.9	29.9

18	NA	31.7	29.4
10	NA	46.3	22.1
12	NA	22.8	24.2
4	NA	23.2	7.3
5	NA	19.2	19.2
3	NA	10.6	8.3
2	NA	10.2	4.3
3	NA	11.4	26.2
2	NA	15.2	3.3
42	NA	28.4	35.4
7	NA	3.8	3.3
NA	NA	15.9	NA
4	NA	10.7	15.4
30	NA	13.6	34.6
NA	NA	19.2	NA
2	NA	30.9	10.3
4	NA	41.5	15.6
4	NA	39.4	14.9
3	NA	35.6	14.6
4	NA	NA	49.2
4	NA	NA	49.2
4	NA	NA	50
4	NA	NA	49.2
4	NA	56.8	51.2
8	NA	59.5	47.6
8	NA	59.5	47.6
8	NA	59.5	47.6
8	NA	59.5	47.6
8	NA	41.3	47.6
8	NA	59.5	47.6
8	NA	59.5	47.6
NA	NA	16.9	NA
6	NA	16.9	40.4
7	NA	53.4	51.5
4	NA	54.6	49.2
4	NA	55	49.6
8	NA	59.5	47.6
4	NA	NA	49.2
5	NA	16.7	25.2
11	NA	19.7	34.8
36	NA	58.4	53.4
47	NA	71.7	77.3
36	NA	39.1	39.3
23	NA	35.4	43.2
19	NA	38.7	38.7
21	NA	30.7	34.4
9	NA	29.9	29.9
20	NA	47.7	47.7
22	NA	50.1	43.9
19	NA	37.9	39.4
3	NA	27.5	7.3
32	NA	59.2	60.9
30	NA	47.4	45.7
10	NA	29.4	24.6
28	NA	36.1	29.6

Trim32 enriched Proteins

1	NA	25.9	4.4
14	NA	36.6	20.1
17	NA	29.2	21
3	NA	14	4.6
9	NA	21.3	21.3
10	NA	4.8	24.6
17	NA	13.2	30.2
1	NA	78.2	6.3
11	NA	NA	45.2
22	NA	31.2	41.8
4	NA	36.5	10.4
46	NA	55.3	58.5
65	NA	64.8	68.2
58	NA	55.6	55.6
4	NA	9.6	14.3
7	NA	10.9	13.7
7	NA	10.9	13.7
11	NA	19.7	15.6
28	NA	28.3	49.8
52	NA	52.2	53.7
56	NA	62.1	63.5
68	NA	52.3	57.7
36	NA	71.6	46.9
5	NA	5.8	8.6
24	NA	4.4	6.8
10	NA	7.8	11.9
45	NA	40.7	44.3
2	NA	36.1	8.7
3	NA	15.4	10.7
7	NA	NA	3
18	NA	26	16.9
9	NA	43.1	30.3
15	NA	28.3	19.2
12	NA	NA	20.2
16	NA	15.6	46.7
12	NA	12.2	23.8
9	NA	3.8	11
15	NA	2.4	14.5
32	NA	35.1	37
26	NA	22.5	33.8
13	NA	20.1	20
52	NA	41	41.3
19	NA	36.6	14.3
7	NA	NA	10.9
23	NA	47.9	47.9
7	NA	18.7	25.7
4	NA	15.3	9.4
2	NA	14.4	5.4
24	NA	28.7	29.7
12	NA	12.4	15
9	NA	11.5	20.8
13	NA	20.5	30.9
12	NA	30.7	34
9	NA	11.6	20.7
30	NA	36.6	47.1

5	NA	6.1	6.1
17	NA	13.7	13.5
7	NA	NA	11.3
5	NA	12.6	8.1
4	NA	10.2	7.9
7	NA	6.8	6.8
5	NA	5.3	11.8
2	NA	3.7	3.7
5	NA	5.4	12.1
NA	NA	11.2	NA
14	NA	18.6	18.6
28	NA	17.8	18.8
NA	NA	18.1	NA
NA	NA	15.8	NA
9	NA	NA	12.3
17	NA	NA	13.4
6	NA	NA	6.6
3	NA	1.8	1.8
13	NA	8.9	8.9
2	NA	4.1	3.9
5	NA	9.1	9.3
28	NA	2	26.3
23	NA	7.1	28.9
7	NA	2.5	13.1
6	NA	4.9	10.5
11	NA	11.1	25.6
39	NA	37.8	42.5
3	NA	8.2	5.7
11	NA	51.1	67.2
10	NA	35.5	34.6
11	NA	NA	18.2
3	NA	5.1	5.8
5	NA	23	43.7
8	NA	12.5	16.6
3	NA	9.3	3.4
6	NA	6.1	24.9
1	NA	9.3	2.1
21	NA	44.6	39.1
2	NA	10.7	3
NA	NA	14.8	NA
7	NA	NA	10
1	NA	NA	0.5
25	NA	25.6	21.8
14	NA	28.5	31.2
11	NA	NA	47.6
8	NA	5.8	16.9
4	NA	26.2	30.8
2	NA	31.6	31.6
4	NA	26.3	26.3
6	NA	1.8	5
5	NA	6.6	13
7	NA	13.1	24.8
2	NA	13.5	13.5
6	NA	9.3	18.3
2	NA	18.2	14.7

55	NA	5.6	11.3
9	NA	9	13.5
4	NA	2.4	11
12	NA	44.7	52.5
5	NA	5.5	11.2
10	NA	5.3	5.2
18	NA	5.1	9.8
7	NA	3.9	8.7
38	NA	27.7	27.8
9	NA	32.8	32.1
31	NA	36.2	40.4
16	NA	14.6	19.4
16	NA	57.3	45.8
11	NA	27.6	28.7
12	NA	47.4	56
10	NA	67.3	62.1
2	NA	14	14
32	NA	41.6	43.2
4	NA	2.3	2.2
14	NA	25.8	14.3
9	NA	48.2	48.2
7	NA	30.5	48.6
9	NA	58.1	58.6
10	NA	NA	68.8
26	NA	38.3	35.8
30	NA	52	49
28	NA	36.2	36.1
20	NA	31.5	35.3
29	NA	43.4	39.1
26	NA	49.2	46.5
17	NA	59.2	59.2
13	NA	1.7	2.8
23	NA	25.3	36.8
5	NA	25.6	20.5
4	NA	8.9	11
3	NA	9.1	8.5
3	NA	21.1	21.1
4	NA	10.4	40.7
6	NA	NA	30.9
8	NA	1.4	6
6	NA	16.9	35.1
6	NA	18.6	38.7
4	NA	20.6	13.6
7	NA	38.1	26.5
5	NA	64	28.7
4	NA	20.7	12.5
4	NA	18.8	19.9
2	NA	23.9	12.2
4	NA	21.2	13.7
2	NA	20.6	13.4
3	NA	21.1	14.8
6	NA	35	31.6
3	NA	44.5	29.5
1	NA	15.1	3.8
4	NA	11.8	11.8

4	NA	21.4	15.8
6	NA	21.4	25.9
2	NA	40.8	14.6
3	NA	24.6	12.6
3	NA	23.2	10.8
1	NA	12.7	4.4
2	NA	21.1	10.2
2	NA	39.8	13.6
3	NA	17.4	14
5	NA	27.5	23.4
9	NA	29.8	28.7
5	NA	35.6	33.3
2	NA	29.2	17.5
3	NA	29	19.5
8	NA	17.8	22.2
2	NA	14.3	5.7
4	NA	19.8	12
5	NA	33	24.3
4	NA	28.8	15.6
7	NA	20.6	18.8
8	NA	43.4	43
9	NA	37.9	25.9
7	NA	13.8	31.4
4	NA	14.1	16.6
6	NA	22	24.3
38	NA	53.2	61.4
10	NA	4.9	4.8
11	NA	31	35.2
7	NA	9.9	30
4	NA	10.1	23.3
9	NA	4.1	22.3
31	NA	49.8	42.2
19	NA	29.2	25.2
7	NA	NA	27.1
28	NA	14.2	17.6
44	NA	24.5	26.8
9	NA	8.3	4.5
33	NA	12.4	15.7
36	NA	13.7	17.6
3	NA	0.8	1.4
22	NA	NA	14.8
93	NA	35.2	50.8
3	NA	13.3	14.9
9	NA	54.3	58.3
14	NA	NA	9.4
20	NA	27.5	23.7
10	NA	1.9	6.2
6	NA	24.4	28.9
14	NA	2.5	17.6
7	NA	2.6	7.6
9	NA	26	48.5
6	NA	3.2	3.6
9	NA	21.1	21.1
7	NA	21.9	26.9
11	NA	NA	46.8

19	NA	46.9	42.4
10	NA	14.6	14.7
4	NA	10.6	7.4
9	NA	10.3	12.4
6	NA	2.2	6.5
40	NA	31.1	33.4
5	NA	8.7	19.2
4	NA	NA	19.8
5	NA	19.2	28.9
32	NA	34.9	47.2
4	NA	16.2	14.6
3	NA	8.5	6.3
11	NA	3.8	10.5
117	NA	63	69.1
2	NA	8.8	4.7
NA	NA	11.3	NA
2	NA	34.6	21.6
4	NA	33.2	26.7
7	NA	1.3	6.2
11	NA	8.7	29.4
6	NA	7.7	8.8
8	NA	NA	8.1
22	NA	32.3	22.3
8	NA	12.4	47.8
3	NA	9.5	5.7
6	NA	11.4	15.7
6	NA	18.6	27.2
9	NA	22.9	23.2
12	NA	31.5	31.5
4	NA	20.6	37.1
12	NA	14.3	12.9
14	NA	49.7	49.7
14	NA	27.9	21.8
7	NA	40.8	22.6
4	NA	3.7	7.7
6	NA	3	21.4
4	NA	13.3	26.5
7	NA	21.1	33
NA	NA	20.5	NA
8	NA	NA	4.3
2	NA	11.4	6.4
4	NA	3.7	6.6
7	NA	6.5	13.9
2	NA	5.2	1.7
9	NA	12.6	10.7
4	NA	12.8	4.3
24	NA	62.4	70.7
3	NA	19.7	12.2
4	NA	34.7	22.1
8	NA	34	36.7
4	NA	9.3	8
13	NA	18.5	31.1
26	NA	42.4	51.9
23	NA	69.8	63.2
31	NA	42.3	52.2

12	NA	20.9	23.3
5	NA	13.4	23.8
6	NA	16.7	12.3
8	NA	5	17.9
3	NA	35.8	9.2
NA	NA	12.5	NA
7	NA	NA	43.4
8	NA	NA	17.1
18	NA	71.9	74.2
13	NA	58.3	58.3
5	NA	28.7	32.5
15	NA	75.5	75.5
8	NA	34.8	34.8
2	NA	40.9	5.5
7	NA	12.7	18.1
8	NA	21.5	13.3
8	NA	29.5	23.1
17	NA	19.6	41.6
23	NA	33.9	38.6
13	NA	33	38.4
5	NA	6.9	10
5	NA	28.1	22.3
7	NA	NA	11.3
9	NA	15.1	15.8
7	NA	58.6	56.4
4	NA	36.4	30.7
2	NA	23.6	8.7
5	NA	6	13.8
11	NA	3.7	55.1
14	NA	19.1	48.8
15	NA	41.7	35.6
10	NA	3.6	19.4
3	NA	32.1	32.1
17	NA	45.4	42.6
2	NA	20.9	3.1
5	NA	5.3	5.3
NA	NA	13	NA
91	NA	6.6	21.9
13	NA	9.3	24.3
5	NA	NA	26.6
2	NA	9.7	4.1
4	NA	14.7	14.2
22	NA	22.4	15.9
23	NA	26	24.1
5	NA	15.5	26.9
6	NA	38.1	33.3
3	NA	57	25.6
4	NA	42.7	42.7
6	NA	2.2	6.6
22	NA	58.8	58.8
11	NA	13	36.5
4	NA	27.2	20.3
1	NA	9.1	2
10	NA	11.4	29.2
9	NA	30.9	34.8

8	NA	24.5	30.3
7	NA	NA	26.6
6	NA	NA	7
12	NA	49.2	58.3
12	NA	47.6	56.6
20	NA	49.4	42.1
7	NA	12	11.2
5	NA	22.6	13.4
2	NA	13.5	4.2
8	NA	29	35.2
12	NA	13.8	17.1
7	NA	55.4	25.9
4	NA	6.7	8.6
10	NA	41.6	41.6
7	NA	6.9	9.7
26	NA	25.7	40.5
10	NA	56.3	56.3
4	NA	44.4	22.2
1	NA	15.6	4.3
6	NA	15.7	25.2
17	NA	8.7	12.2
5	NA	9.3	11.7
6	NA	10.5	16
17	NA	26.7	26.5
6	NA	23.6	26.5
19	NA	60.9	50.7
14	NA	34.2	30.3
4	NA	21.5	19.7
9	NA	41.7	26.2
10	NA	30.3	31.7
2	NA	8.4	4.1
4	NA	17.7	11.5
6	NA	8	8.1
22	NA	6.7	9.2
12	NA	46.5	46.5
12	NA	46.5	46.5
12	NA	44	53.8
13	NA	31.4	45.7
12	NA	47.4	46.1
12	NA	2.5	9.8
19	NA	1.7	9.3
1	8.1	8.1	8.1
16	NA	71.5	52.5
10	NA	59.8	50
7	NA	34.1	25.9
9	NA	64.4	43.7
9	NA	51.5	51.5
11	NA	52.8	48
14	NA	63.3	63.3
12	NA	59.2	58.8
7	NA	46.3	36.8
7	NA	47.8	39
7	NA	40.9	40.9
15	NA	63.6	61
8	NA	52.1	34.5

10	NA	59.2	41.2
19	NA	62.5	52.5
21	NA	58	55.4
17	NA	60.4	45.2
20	NA	65.3	57.4
22	NA	55.2	62.3
19	NA	61.4	59.6
29	NA	52.6	42.2
5	NA	12.6	35.1
22	NA	62.8	57.6
13	NA	47.6	34.6
19	NA	64.9	64.9
10	NA	65.8	54.5
41	NA	57.2	58.9
26	NA	62.8	52.6
9	NA	38.8	33.5
12	NA	22.6	27.6
21	NA	63	54.8
9	NA	45.5	34.6
9	NA	37.1	30
1	NA	18.5	4.5
2	NA	18.9	10.9
4	NA	30.3	20.1
2	NA	4.3	1.3
2	NA	14.5	9.3
14	NA	46.1	53.5
5	NA	28.9	9.2
8	NA	35.6	43.1
15	NA	1.1	18
5	NA	13.5	22.5
2	NA	2.2	1
12	NA	26.1	27.5
7	NA	3.5	7.3
9	NA	3.9	11.6
7	NA	31.8	31.8
10	NA	64.2	50.3
9	NA	19.8	20.3
10	NA	12.3	35
12	NA	46.9	43.4
22	NA	26.8	30.8
5	NA	10.3	19.1
8	NA	39.6	45.5
8	NA	26	41
7	NA	21.3	32.4
7	NA	21.1	32.1
3	NA	NA	14.4
8	NA	26	46
4	NA	12.6	24.8
7	NA	34.3	42.8
4	NA	14.2	27.4
7	NA	12.4	44.3
5	NA	22.9	23.4
7	NA	NA	42
13	NA	2.2	11.7
5	NA	20.5	27

4	NA	20.5	20.5
7	NA	41.2	41.2
8	NA	30.8	39.4
10	NA	28.5	62.3
7	NA	23.7	34.3
5	NA	16.4	27.1
3	NA	4	3.6
5	NA	7.8	24.5
5	NA	2.4	18.2
9	NA	44.6	44.2
8	NA	40.3	40.3
10	NA	2.1	4.7
14	NA	8.5	29
7	NA	20.7	45.1
7	NA	20.7	45.1
32	NA	45.3	52.9
4	NA	8.8	8.1
4	NA	NA	18.1
7	NA	17.6	9.2
11	NA	16.2	24.2
7	NA	7.8	13
6	NA	55.6	46.4
5	NA	40.4	23.8
7	NA	36.3	19.4
14	NA	38.7	19.6
2	NA	26.4	8.1
10	NA	42.8	47.1
2	NA	20.6	10.6
6	NA	8.5	23.1
30	NA	38.8	48.9
5	NA	4.2	11.7
10	NA	32.4	35.8
8	NA	14	31.2
7	NA	19	25
9	NA	30.1	38.6
4	NA	10.4	19.2
11	NA	42.1	30
4	NA	17.4	11.8
16	NA	37.1	33.5
6	NA	39.6	31.1
2	NA	22.3	22.3
3	NA	3.3	2.8
13	NA	17.5	47.9
9	NA	37.4	43.5
12	NA	37.6	39.3
8	NA	28.4	37
9	NA	30	37.9
7	NA	29	31.8
9	NA	40.2	43.6
9	NA	37	47.3
8	NA	25	41.5
10	NA	39.2	48.9
7	NA	18	27.3
7	NA	37.5	38.8
8	NA	50.8	61.7

6	NA	51.6	51.6
10	NA	45.7	52.1
13	NA	50.4	50.4
6	NA	29.2	35.6
12	NA	49	48.3
5	NA	43.4	43.4
7	NA	27.7	32.1
4	NA	24.4	24.4
17	NA	38.5	40.9
13	NA	59.1	67
11	NA	39.2	50.4
7	NA	33.3	45.2
6	NA	22.2	21.4
6	NA	26.4	38.2
5	NA	30.5	30.5
4	NA	22.6	23.6
5	NA	28.6	28.6
3	NA	28.3	42.4
7	NA	50	50
19	NA	29.6	44.4
16	NA	41.1	46.1
12	NA	34.1	36.8
19	NA	27.4	45.9
18	NA	41.4	45.1
NA	NA	36.1	NA
11	4.3	45.1	45.5
9	NA	60.4	60.9
16	NA	55.8	57.7
3	NA	51.8	51.8
10	NA	69.6	82.6
21	NA	10.2	48.4
14	NA	4.9	30.3
7	NA	33.6	34
7	NA	47.9	41.8
13	NA	51.9	55.1
10	NA	54.5	53.8
9	NA	43	42.4
12	NA	39.1	40.4
6	NA	40.7	64.8
12	NA	58.5	58.5
18	NA	58.9	64.4
17	NA	64.4	64.4
23	NA	62.5	62.5
15	NA	51.7	51.7
10	NA	41	35.2
3	NA	28.6	25.2
6	NA	41	41
6	NA	29.4	30.1
4	NA	29.3	29.3
4	NA	29.6	29.6
7	NA	50.4	50.4
9	NA	54.5	54.5
5	NA	40.5	40.5
3	NA	30.4	30.4
6	NA	48.2	48.2

20	NA	74.5	75.7
21	NA	66.7	64
17	NA	54	60.8
11	NA	47.3	49.5
8	NA	29.7	29.7
6	NA	NA	21.2
12	NA	60.8	63.4
10	NA	48.1	51.4
15	NA	40.2	49.5
19	NA	56.9	56.9
19	NA	56.1	56.1
4	NA	6.4	17.1
3	NA	14.7	9.6
3	NA	20.9	11.8
25	NA	10.7	20.8
8	NA	6.9	13.5
6	NA	2.7	19.9
10	NA	30.8	37.3
16	NA	42.4	38.8
11	NA	17.6	26.2
2	NA	2	2
19	NA	20.5	29.1
15	NA	45.2	46.5
19	NA	42.5	45.4
2	NA	32	5.4
1	NA	10	1.4
NA	NA	10.8	NA
4	NA	23.2	27.3
3	NA	11.1	15.2
19	NA	50	47.7
22	NA	41.4	25.6
10	NA	39.6	43.2
4	NA	NA	15.9
7	NA	1.6	15.6
8	NA	6.1	6.4
12	NA	11.1	23.3
7	NA	14.9	24.5
4	NA	12.1	16.8
9	NA	5.4	4.8
6	NA	22.3	34
15	NA	19.2	26.8
11	NA	11.5	23.3
9	NA	NA	10.3
9	NA	13.9	9.9
12	NA	5.2	14.4
3	NA	27.1	31.2
4	NA	30.9	30.9
3	NA	6	6
10	NA	31.3	23.9
13	NA	49	58.7
12	NA	35.7	34.3
24	NA	29.7	53.3
22	NA	44.7	47.4
6	NA	34.5	17.4
9	NA	54.9	40.5

4	NA	5.4	8.1
7	NA	14.2	9.7
2	NA	16.4	4.4
20	NA	29.3	17.6
9	NA	18.1	13.3
13	NA	30.7	13.9
25	NA	35.8	26.9
10	NA	9.9	40.4
8	NA	NA	16.9
41	NA	72	62.9
12	NA	15.2	15.4
3	NA	51.5	32.5
7	NA	5.1	30.3
4	NA	3.4	13.7
17	NA	34.9	55.4
17	NA	42.6	49.3
1	NA	10	1.1
22	NA	6.7	23.8
3	NA	15.8	3.7
16	NA	8.9	13.6
NA	NA	4.8	NA
12	NA	25.7	18.6
6	NA	10	8.8
4	NA	15.5	22.7
38	NA	35.1	52.6
31	NA	19.4	17.9
5	NA	10.6	16.5
10	NA	28.2	28.2
9	NA	29.4	36.5
9	NA	16.9	25.5
5	NA	16.9	28.4
5	NA	54.6	54.6
8	NA	49.2	49.2
4	NA	31	31
2	NA	12	25
4	NA	15.1	24.4
9	NA	16.2	24.6
4	NA	6.7	8.6
7	NA	NA	19.3
3	NA	15	25
7	NA	NA	17.6
3	NA	NA	3.8
6	NA	11.9	29.2
84	NA	37.8	42.6
4	NA	4.4	1.5
NA	NA	37.2	NA
66	NA	39.9	38.6
4	NA	14.3	10
5	NA	2.1	5
4	NA	24.5	40.9
2	NA	33.1	33.1
10	NA	10.5	22.6
10	NA	8.5	22.6
13	NA	17	27.7
6	NA	8.9	12.1

7	NA	2.6	11.9
6	NA	NA	31.2
15	NA	11.1	17.5
18	NA	52.4	47.2
3	NA	14.5	14.5
8	NA	37.2	31.1
4	NA	6.9	8.1
6	NA	19.8	20.2
7	NA	NA	18.3
6	NA	16.1	20.2
7	NA	17.1	16.7
18	NA	42.4	44.3
5	NA	49.3	43.9
4	NA	NA	21
5	NA	NA	36.6
4	NA	4.5	6.5
5	NA	31.2	46.2
4	NA	15.9	9.4
32	NA	55.1	54.3
5	NA	6.8	16.7
17	NA	52.9	61.4
8	NA	8.9	14.2
5	NA	7.6	9.9
13	NA	28.3	45.1
NA	NA	11.2	NA
4	NA	32.4	15
5	NA	8.2	5.3
19	NA	32.1	24.7
10	NA	7.3	7
4	NA	17.5	17.9
1	NA	51.5	6.1
3	NA	13.4	9.8
13	NA	28.7	32.4
30	NA	44	47.2
4	NA	6.7	5.6
12	NA	43.5	53.7
14	NA	37.9	45.2
4	NA	17.8	45.8
28	NA	62.6	63.1
5	NA	NA	12.7
10	NA	29.9	33.6
11	NA	NA	16.5
3	NA	8.2	6.1
5	NA	7.9	7.5
7	NA	21.7	22
3	NA	5.7	16.5
10	NA	24.5	25
3	NA	17.7	17.7
40	NA	3.8	31.9
4	NA	9	7.7
5	NA	10.8	2.6
5	NA	13.7	21.5
4	NA	10.4	24.9
4	NA	4.8	25.1
4	NA	8.5	20

3	NA	12.4	8.6
11	NA	NA	40.2
6	NA	15.3	19.6
NA	NA	25.9	NA
1	NA	1.5	1.5
7	NA	2.8	4.7
27	NA	31	39.4
7	NA	6.8	10.8
6	NA	9.1	8.2
14	NA	7.1	47.2
6	NA	6.9	14.1
NA	NA	12.3	NA
NA	NA	7.3	NA
NA	NA	5.9	NA
5	NA	16.1	21.1
7	NA	17.6	27.8
14	NA	18.9	44.4
13	NA	21.8	41.9
37	NA	15.8	35.5
7	NA	9.4	3.5
5	NA	22.1	54.1
3	NA	10.4	13.9
6	NA	34.7	9.8
11	NA	NA	16.5
22	NA	40.6	34.9
7	1.1	4.9	10.2
6	NA	9.6	14
2	NA	8.8	4
8	NA	26.4	22.3
3	NA	46.6	17.9
11	NA	46.1	30.4
9	NA	35.3	24.5
NA	NA	6	NA
4	NA	NA	19
1	4.1	4.1	4.1
4	NA	8.5	15.8
2	NA	10.1	11
10	NA	7.1	19.7
3	NA	21.4	11.1
7	NA	0.2	0.2
36	11.8	74.1	80.5
NA	11.8	43.4	NA
19	NA	52	57.1
4	NA	8.3	8.3
7	NA	11.1	11.1
39	9.4	74.2	74.2
39	9.4	74.2	74.2
18	NA	51.6	40.7
22	NA	61.9	62.2
40	NA	73.9	74.2
41	NA	74.1	74.3
23	NA	64.7	72
7	NA	14.6	19.5
13	NA	39.4	32.5
3	NA	6.3	10

10	NA	NA	20.6
5	NA	42.9	42.9
6	NA	16.3	19.7
15	NA	66.1	65.7
5	NA	26.4	22.2
10	NA	44.6	41.9
11	NA	11.9	14.7
1	NA	19.9	2.8
1	NA	14.1	7
9	NA	1.1	12.8
19	NA	5.9	28.8
1	NA	5.9	3
4	NA	2.5	16.9
2	NA	29.5	16.4
3	NA	10.5	19.5
4	NA	27.9	16.9
6	NA	53.3	46.7
8	NA	3.7	7.8
4	NA	14.7	19.3
3	NA	31.3	21.8
4	NA	5.6	11
15	NA	24.5	21.3
6	NA	1.1	6.7
7	NA	13.3	9.2
4	NA	15.9	28
39	NA	9.1	10.5
11	NA	6.1	5.2
7	NA	44.8	41.8
3	NA	8.1	7.5
13	NA	56.2	46.2
4	NA	13	20.5
42	NA	42.4	40.8
2	NA	22.8	4.1
4	NA	15.6	9.6
47	NA	37.3	50.7
11	NA	12.8	31.6
5	NA	24.1	21.5
14	NA	20.2	17.9
18	NA	60.2	45.2
9	NA	NA	10.3
7	NA	0.8	3.8
2	NA	4.5	2.1
24	NA	19.5	22.4
25	NA	46.9	49.2
9	NA	20.8	9.7
45	NA	18.6	21.3
12	NA	NA	4.6
9	NA	5.4	12.9
8	NA	9.2	44.6
7	NA	4.9	43.2
24	NA	14.1	24.5
6	NA	35.2	39.8
NA	NA	12	NA
63	NA	69.7	75.6
16	NA	21.4	19.1

13	NA	52.4	59.8
9	NA	34.9	45.8
7	NA	27.2	35.7
26	NA	59.2	57.3
NA	NA	0.7	NA
2	NA	3.4	1.7
6	NA	49.5	45.1
16	NA	20.1	23.5
5	NA	16	31
8	NA	18.3	17.3
4	NA	1.8	4.5
4	NA	17.3	7.5
NA	NA	13.2	NA
9	NA	55.2	47.2
9	NA	34.2	34.2
8	NA	30.4	29.1
5	NA	12.8	38.4
12	NA	22.6	14.3
7	NA	9.9	7.3
6	NA	21.9	10.7
5	NA	21.1	10.7
4	NA	4.4	6.3
23	NA	36.3	49.1
14	NA	53.1	65.8
9	NA	21.1	32.7
4	NA	15.2	20.7
9	NA	NA	19.7
7	NA	12.9	14.5
11	NA	17.4	27.2
14	NA	63.4	63.4
23	NA	74.1	74.1
11	NA	53	53
14	NA	56.9	56.9
18	NA	50.8	50.8
19	NA	69.4	69.4
5	NA	7.7	13.8
6	NA	4.4	6.8
3	NA	5.7	4.7
