# Supplementary material for: The RNA helicase DDX6 regulates cell-fate specification in neural stem cells via miRNAs
Source: Nucleic Acids Res. 2015 Feb 26;43(5):2638–54. doi: 10.1093/nar/gkv138 (PMC4357729; doi:10.1093/nar/gkv138)
Supplement: SUPPLEMENTARY DATA [file supp_gkv138_nar-03344-y-2014-File012.pdf]

Enriched GOBP categories by differentially more abunda		
**Gene Ontology ID**	**Odds ratio**	**Adjusted p-value**
GO:0044237	3.19	0.0002
GO:0008152	2.93	0.0020
GO:0044238	2.64	0.0023
GO:0009987	4.30	0.0029
GO:0044281	2.31	0.0029
GO:0019752	2.80	0.0060
GO:0043436	2.80	0.0060
GO:0006082	2.77	0.0062
GO:0042180	2.77	0.0062
GO:0008150	4.67	0.0086
GO:0016054	4.95	0.0089
GO:0046395	4.95	0.0089
GO:0010467	2.02	0.0106
GO:0034641	1.96	0.0148
GO:0000377	2.96	0.0155
GO:0000398	2.96	0.0155
GO:0043487	9.06	0.0155
GO:0043488	9.06	0.0155
GO:0006807	1.94	0.0172
GO:0000375	2.89	0.0174
GO:0007091	8.41	0.0180
GO:0055114	2.65	0.0187
GO:0008380	2.70	0.0219
GO:0033539	23.16	0.0233
GO:0006397	2.47	0.0265
GO:0090304	1.88	0.0276
GO:0050769	6.92	0.0276
GO:0030049	9.34	0.0291
GO:0033275	9.34	0.0291
GO:0043489	9.34	0.0291
GO:0048255	9.34	0.0291
GO:0070252	9.34	0.0291
GO:0006635	6.53	0.0291
GO:0009062	6.53	0.0291
GO:0072329	6.53	0.0291
GO:0016584	17.37	0.0291
GO:0070934	17.37	0.0291
GO:0016070	1.87	0.0294
GO:0006099	6.19	0.0328
GO:0009109	6.19	0.0328
GO:0010948	6.19	0.0328
GO:0046356	6.19	0.0328
GO:0044085	2.02	0.0330
GO:0010720	5.88	0.0360
GO:0044260	1.79	0.0360
GO:0043170	1.80	0.0360
GO:0051187	5.59	0.0385
GO:0032787	3.22	0.0394
GO:0022607	2.02	0.0497
GO:0006139	1.73	0.0497
GO:0019395	5.10	0.0500
GO:0034440	5.10	0.0500

GO:0016053	3.61	0.0540
GO:0046394	3.61	0.0540
GO:0009060	4.89	0.0562
GO:0055006	9.93	0.0582
GO:0055013	9.93	0.0582
GO:0030048	6.22	0.0582
GO:0051289	6.22	0.0582
GO:0009063	4.51	0.0674
GO:0030258	4.51	0.0674
GO:0006396	1.97	0.0690
GO:0030239	8.69	0.0693
GO:0008104	0.36	0.0700
GO:0015980	2.61	0.0702
GO:0006084	4.34	0.0702
GO:0009310	4.34	0.0702
GO:0006631	3.25	0.0702
GO:0007088	5.48	0.0713
GO:0051783	5.48	0.0713
GO:0051090	3.62	0.0720
GO:0070727	0.27	0.0744
GO:0031324	2.10	0.0786
GO:0031032	7.72	0.0786
GO:0044242	4.04	0.0819
GO:0016071	1.85	0.0831
GO:0045333	3.01	0.0890
GO:0015031	0.35	0.0890
GO:0045184	0.35	0.0890
GO:0006936	3.90	0.0890
GO:0055007	6.95	0.0916
GO:0034613	0.27	0.0935

nt proteins in differentiating NSCs in comparison to proliferating NSCs
**Gene Ontology name**
cellular metabolic process
metabolic process
primary metabolic process
cellular process
small molecule metabolic process
carboxylic acid metabolic process
oxoacid metabolic process
organic acid metabolic process
cellular ketone metabolic process
biological_process
organic acid catabolic process
carboxylic acid catabolic process
gene expression
cellular nitrogen compound metabolic process
RNA splicing, via transesterification reactions with bulged adenosine as nucleophile
nuclear mRNA splicing, via spliceosome
regulation of RNA stability
regulation of mRNA stability
nitrogen compound metabolic process
RNA splicing, via transesterification reactions
mitotic metaphase/anaphase transition
oxidation-reduction process
RNA splicing
fatty acid beta-oxidation using acyl-CoA dehydrogenase
mRNA processing
nucleic acid metabolic process
positive regulation of neurogenesis
muscle filament sliding
actin-myosin filament sliding
RNA stabilization
mRNA stabilization
actin-mediated cell contraction
fatty acid beta-oxidation
fatty acid catabolic process
monocarboxylic acid catabolic process
nucleosome positioning
CRD-mediated mRNA stabilization
RNA metabolic process
tricarboxylic acid cycle
coenzyme catabolic process
negative regulation of cell cycle process
acetyl-CoA catabolic process
cellular component biogenesis
positive regulation of cell development
cellular macromolecule metabolic process
macromolecule metabolic process
cofactor catabolic process
monocarboxylic acid metabolic process
cellular component assembly
nucleobase-containing compound metabolic process
fatty acid oxidation
lipid oxidation

organic acid biosynthetic process
carboxylic acid biosynthetic process
aerobic respiration
cardiac cell development
cardiac muscle cell development
actin filament-based movement
protein homotetramerization
cellular amino acid catabolic process
lipid modification
RNA processing
myofibril assembly
protein localization
energy derivation by oxidation of organic compounds
acetyl-CoA metabolic process
amine catabolic process
fatty acid metabolic process
regulation of mitosis
regulation of nuclear division
regulation of sequence-specific DNA binding transcription factor activity
cellular macromolecule localization
negative regulation of cellular metabolic process
actomyosin structure organization
cellular lipid catabolic process
mRNA metabolic process
cellular respiration
protein transport
establishment of protein localization
muscle contraction
cardiac muscle cell differentiation
cellular protein localization
