# Supplementary material for: The RNA helicase DDX6 regulates cell-fate specification in neural stem cells via miRNAs
Source: Nucleic Acids Res. 2015 Feb 26;43(5):2638–54. doi: 10.1093/nar/gkv138 (PMC4357729; doi:10.1093/nar/gkv138)
Supplement: SUPPLEMENTARY DATA [file supp_gkv138_nar-03344-y-2014-File013.pdf]

Identified proteins belonging to the key GO categories (neurogenesis and RNA regulation) ar

Uniprot accession	Gene symbol	MGI Description	Identified in the current study
Q99JY9	ACTR3	ARP3 actin-related protein 3	Yes
Q99MU3	ADAR	adenosine deaminase, RNA-specific	No
Q8CJG1	AGO1	argonaute RISC catalytic subunit 1	No
Q8CJG0	AGO2	argonaute RISC catalytic subunit 2	Yes
P31750	AKT1	thymoma viral proto-oncogene 1	No
O08583	ALYREF	Aly/REF export factor	Yes
Q8BZQ7	ANAPC2	anaphase promoting complex subunit	Yes
P28352	APEX1	apurinic/apyrimidinic endonuclease 1	Yes
P51908	APOBEC1	apolipoprotein B mRNA editing enzym	No
P12023	APP	amyloid beta (A4) precursor protein	No
Q99PT1	ARHGDIA	Rho GDP dissociation inhibitor (GDI) a	No
Q62388	ATM	ataxia telangiectasia mutated homolog	No
O70445	BARD1	BRCA1 associated RING domain 1	No
Q04211	BTG2	B cell translocation gene 2, anti-prolife	No
Q60865	CAPRIN1	cell cycle associated protein 1	Yes
Q8CH18	CCAR1	cell division cycle and apoptosis regula	Yes
P24860	Ccnb1	cyclin B1	No
Q61458	CCNH	cyclin H	No
Q9CWK3	CD2BP2	CD2 antigen (cytoplasmic tail) binding	No
Q6A068	CDC5L	cell division cycle 5-like (S. pombe)	Yes
Q14AX6	CDK12	cyclin-dependent kinase 12	No
Q03147	CDK7	cyclin-dependent kinase 7	No
Q9Z0H4	CELF2	CUGBP, Elav-like family member 2	No
P60824	CIRBP	cold inducible RNA binding protein	Yes
Q61189	CLNS1A	chloride channel, nucleotide-sensitive,	No
Q6ZQ08	CNOT1	CCR4-NOT transcription complex, sub	Yes
Q8BT14	CNOT4	CCR4-NOT transcription complex, sub	No
Q60809	CNOT7	CCR4-NOT transcription complex, sub	No
Q9D8X5	CNOT8	CCR4-NOT transcription complex, sub	No
Q6NVF9	CPSF6	cleavage and polyadenylation specific	Yes
Q8BIQ5	CSTF2	cleavage stimulation factor, 3' pre-RNA	No
Q9CYC6	DCP2	DCP2 decapping enzyme homolog (S.	No
Q91VR5	DDX1	DEAD (Asp-Glu-Ala-Asp) box polypept	Yes
Q501J6	DDX17	DEAD (Asp-Glu-Ala-Asp) box polypept	Yes
Q9Z1N5	DDX39B	DEAD (Asp-Glu-Ala-Asp) box polypept	Yes
Q91VN6	DDX41	DEAD (Asp-Glu-Ala-Asp) box polypept	No
Q8BTS0;Q61656	DDX5	DEAD (Asp-Glu-Ala-Asp) box polypept	Yes
P54823	DDX6	DEAD (Asp-Glu-Ala-Asp) box polypept	Yes
G3X8X0	DHX16	DEAH (Asp-Glu-Ala-His) box polypepti	No
E9QNN1;O70133	DHX9	DEAH (Asp-Glu-Ala-His) box polypepti	Yes
Q9CSH3	DIS3	DIS3 mitotic control homolog (S. cerev	Yes
Q99KV1	DNAJB11	DnaJ (Hsp40) homolog, subfamily B, n	Yes
Q61501	E2F1	E2F transcription factor 1	No
Q3UJB9;D6RE33	EDC4	enhancer of mRNA decapping 4	Yes
A2AH85;G3UZ34;O08	EFTUD2	elongation factor Tu GTP binding dom	Yes
P60843	EIF4A1	eukaryotic translation initiation factor 4	Yes
Q8BGD9	EIF4B	eukaryotic translation initiation factor 4	Yes
P63073	EIF4E	eukaryotic translation initiation factor 4	Yes
Q6NZJ6	EIF4G1	eukaryotic translation initiation factor 4	Yes
P70372	ELAVL1	ELAV (embryonic lethal, abnormal visi	Yes

Q61701	ELAVL4	ELAV (embryonic lethal, abnormal visi	No
B2RWS6	EP300	E1A binding protein p300	No
Q9EQY0	ERN1	endoplasmic reticulum (ER) to nucleus	No
Q8VBV3	EXOSC2	exosome component 2	Yes
Q921I9	EXOSC4	exosome component 4	Yes
P56959	FUS	fused in sarcoma	Yes
Q8BX17	GEMIN5	gem (nuclear organelle) associated pr	Yes
Q9DBA9	GTF2H1	general transcription factor II H, polype	No
P70288	HDAC2	histone deacetylase 2	Yes
Q9CX86	HNRNPA0	heterogeneous nuclear ribonucleoprot	Yes
Q5EBP8;P49312	HNRNPA1	heterogeneous nuclear ribonucleoprot	Yes
O88569	HNRNPA2B1	heterogeneous nuclear ribonucleoprot	Yes
Q60668	HNRNPD	heterogeneous nuclear ribonucleoprot	Yes
Q9Z2X1	HNRNPF	heterogeneous nuclear ribonucleoprot	Yes
O35737	HNRNPH1	heterogeneous nuclear ribonucleoprot	Yes
D3YWT1;D3Z3N4	HNRNPH3	heterogeneous nuclear ribonucleoprot	Yes
P61979	HNRNPK	heterogeneous nuclear ribonucleoprot	Yes
Q8R081	HNRNPL	heterogeneous nuclear ribonucleoprot	Yes
Q9D0E1	HNRNPM	heterogeneous nuclear ribonucleoprot	Yes
G3XA10;Q8VEK3	HNRNPU	heterogeneous nuclear ribonucleoprot	Yes
Q61696	HSPA1A	heat shock protein 1A	Yes
P63017	HSPA8	heat shock protein 8	Yes
P14602	HSPB1	heat shock protein 1	No
P41136	ID2	inhibitor of DNA binding 2	No
Q9ERI5	JMJD6	jumonji domain containing 6	No
Q3U0V1	KHSRP	KH-type splicing regulatory protein	Yes
P16110	LGALS3	lectin, galactose binding, soluble 3	No
O35900	LSM2	LSM2 homolog, U6 small nuclear RNA	Yes
Q9QXA5	LSM4	LSM4 homolog, U6 small nuclear RNA	Yes
Q5SUF2	LUC7L3	LUC7-like 3 (S. cerevisiae)	No
P14873	MAP1B	microtubule-associated protein 1B	Yes
Q9WUI1	MAPK11	mitogen-activated protein kinase 11	No
P47811	MAPK14	mitogen-activated protein kinase 14	No
P49138	MAPKAPK2	MAP kinase-activated protein kinase 2	No
P10637	MAPT	microtubule-associated protein tau	No
P51949	MNAT1	menage a trois 1	No
P22366	MYD88	myeloid differentiation primary respons	No
P10085	MYOD1	myogenic differentiation 1	No
P60322	NANOS2	nanos homolog 2 (Drosophila)	No
Q3UYV9	NCBP1	nuclear cap binding protein subunit 1	Yes
Q9CQ49	NCBP2	nuclear cap binding protein subunit 2	No
Q9Z0W1	NGFR	nerve growth factor receptor (TNFR su	No
Q9D0T1	NHP2L1	NHP2 non-histone chromosome protei	No
Q99K48;F6XLC7	NONO	non-POU-domain-containing, octamer	Yes
Q8VE62	PAIP1	polyadenylate binding protein-interactir	No
Q8VDG3	PARN	poly(A)-specific ribonuclease (deadeny	No
P60335	PCBP1	poly(rC) binding protein 1	Yes
Q9CR73	PNRC2	proline-rich nuclear receptor coactivato	No
P08775	POLR2A	polymerase (RNA) II (DNA directed) po	Yes
Q923G2	POLR2H	polymerase (RNA) II (DNA directed) po	Yes
Q6RI63	PPARG	peroxisome proliferator activated recep	No
O70343	PPARGC1A	peroxisome proliferative activated rece	No
P63330	PPP2CA	protein phosphatase 2 (formerly 2A), c	Yes
Q76MZ3	PPP2R1A	protein phosphatase 2 (formerly 2A), re	Yes
Q6P1F6	PPP2R2A	protein phosphatase 2 (formerly 2A), re	Yes

Q0VGB7	PPP4R2	protein phosphatase 4, regulatory subu	Yes
P20444	PRKCA	protein kinase C, alpha	No
P28867	PRKCD	protein kinase C, delta	No
Q8CIG8	PRMT5	protein arginine N-methyltransferase 5	Yes
Q99KP6	PRPF19	PRP19/PSO4 pre-mRNA processing fa	Yes
Q922U1	PRPF3	PRP3 pre-mRNA processing factor 3 h	No
Q61136	PRPF4B	PRP4 pre-mRNA processing factor 4 h	No
Q91YR7	PRPF6	PRP6 pre-mRNA splicing factor 6 hom	Yes
Q99PV0	PRPF8	pre-mRNA processing factor 8	Yes
Q9R1P4	PSMA1	proteasome (prosome, macropain) sub	Yes
P70195	PSMB7	proteasome (prosome, macropain) sub	Yes
P62192	PSMC1	protease (prosome, macropain) 26S su	Yes
P46471	PSMC2	proteasome (prosome, macropain) 26S	Yes
O88685	PSMC3	proteasome (prosome, macropain) 26S	Yes
P54775	PSMC4	proteasome (prosome, macropain) 26S	Yes
P62196	PSMC5	protease (prosome, macropain) 26S su	Yes
P62334	PSMC6	proteasome (prosome, macropain) 26S	Yes
Q3TXS7	PSMD1	proteasome (prosome, macropain) 26S	Yes
Q9Z2X2	PSMD10	proteasome (prosome, macropain) 26S	Yes
Q9WVJ2	PSMD13	proteasome (prosome, macropain) 26S	Yes
Q8VDM4	PSMD2	proteasome (prosome, macropain) 26S	Yes
O35226	PSMD4	proteasome (prosome, macropain) 26S	Yes
Q9CR00	PSMD9	proteasome (prosome, macropain) 26S	Yes
G3UXZ5;P97371	PSME1	proteasome (prosome, macropain) act	Yes
P61290	PSME3	proteaseome (prosome, macropain) ac	Yes
Q5SSW2	PSME4	proteasome (prosome, macropain) act	Yes
Q8BHL8	PSMF1	proteasome (prosome, macropain) inh	No
Q8CB58;Q8BGJ5;Q9	PTBP1	polypyrimidine tract binding protein 1	Yes
Q91Z31	PTBP2	polypyrimidine tract binding protein 2	No
Q3UEB3	PUF60	poly-U binding splicing factor 60	Yes
Q9JJ43	RBFOX1	RNA binding protein, fox-1 homolog (C	No
Q8BP71	RBFOX2	RNA binding protein, fox-1 homolog (C	No
Q8BIF2	RBFOX3	RNA binding protein, fox-1 homolog (C	No
Q6PHZ5	RBM15B	RNA binding motif protein 15B	No
Q62176	RBM38	RNA binding motif protein 38	No
Q8VH51	RBM39	RNA binding motif protein 39	No
Q8C7Q4	RBM4	RNA binding motif protein 4	No
Q9CWZ3	RBM8A	RNA binding motif protein 8a	No
Q4VGL6	RC3H1	RING CCCH (C3H) domains 1	No
Q04207	RELA	v-rel reticuloendotheliosis viral oncoge	Yes
Q9QUI0	RHOA	ras homolog gene family, member A	Yes
Q05921	RNASEL	ribonuclease L (2', 5'-oligoisoadenylate	No
Q9CR57	RPL14	ribosomal protein L14	Yes
Q9D823	RPL37	ribosomal protein L37	No
P99027	RPLP2	ribosomal protein, large P2	Yes
P62264	RPS14	ribosomal protein S14	Yes
P62908	RPS3	ribosomal protein S3	Yes
Q9JKY0	RQCD1	rcd1 (required for cell differentiation) h	Yes
Q99P72	RTN4	reticulon 4	Yes
Q9Z315	SART1	squamous cell carcinoma antigen reco	No
Q9CY58	SERBP1	serpine1 mRNA binding protein 1	Yes
Q07235	SERPINE2	serine (or cysteine) peptidase inhibitor,	Yes
A2BE93;Q9EQU5	SET	SET nuclear oncogene	Yes
A2AKX3	SETX	senataxin	No
Q99NB9	SF3B1	splicing factor 3b, subunit 1	Yes

Q8QZY9	SF3B4	splicing factor 3b, subunit 4	No
Q8VIJ6	SFPQ	splicing factor proline/glutamine rich (p	Yes
Q3USH5	SFSWAP	splicing factor, suppressor of white-apı	No
Q60665	SKIL	SKI-like	No
P97440	SLBP	stem-loop binding protein	No
P41251	SLC11A1	solute carrier family 11 (proton-couple	No
Q9CU62	SMC1A	structural maintenance of chromosom	Yes
Q8BKX6	SMG1	SMG1 homolog, phosphatidylinositol 3	No
Q6ZPY2	SMG5	Smg-5 homolog, nonsense mediated r	No
Q5RJH6	SMG7	Smg-7 homolog, nonsense mediated r	No
P97801	SMN1	survival motor neuron 1	No
Q8BGT7	SMNDC1	survival motor neuron domain containi	Yes
Q6P4T2	SNRNP200	small nuclear ribonucleoprotein 200 (U	Yes
P27048	SNRPB	small nuclear ribonucleoprotein B	Yes
Q62241	SNRPC	U1 small nuclear ribonucleoprotein C	No
P62315	SNRPD1	small nuclear ribonucleoprotein D1	Yes
P62317	SNRPD2	small nuclear ribonucleoprotein D2	Yes
P62320	SNRPD3	small nuclear ribonucleoprotein D3	Yes
P62305	SNRPE	small nuclear ribonucleoprotein E	Yes
P62307	SNRPF	small nuclear ribonucleoprotein polype	Yes
Q9CSN1	SNW1	SNW domain containing 1	No
Q7M6Y2	SOX11	SRY-box containing gene 11	No
Q8BZX4	SREK1	splicing regulatory glutamine/lysine-ric	No
O70551	SRPK1	serine/arginine-rich protein specific kin	No
O54781	SRPK2	serine/arginine-rich protein specific kin	Yes
Q6PDM2	SRSF1	serine/arginine-rich splicing factor 1	Yes
Q9R0U0	SRSF10	serine/arginine-rich splicing factor 10	No
P84104	SRSF3	serine/arginine-rich splicing factor 3	Yes
Q542V3;E9Q4U8;A2A	SRSF4	serine/arginine-rich splicing factor 4	Yes
O35326;E9QKW3;Q6	SRSF5	serine/arginine-rich splicing factor 5	Yes
Q3TWW8	SRSF6	serine/arginine-rich splicing factor 6	Yes
Q8BL97	SRSF7	serine/arginine-rich splicing factor 7	Yes
P32067	SSB	Sjogren syndrome antigen B	Yes
Q9CY97	SSU72	Ssu72 RNA polymerase II CTD phosp	No
Q7TMK9;G3UZI2;G3U	SYNCRIP	synaptotagmin binding, cytoplasmic R	Yes
Q921F2	TARDBP	TAR DNA binding protein	Yes
P52912;D3Z4H6;D3Y	TIA1	cytotoxic granule-associated RNA binc	Yes
Q99JY1	TIRAP	toll-interleukin 1 receptor (TIR) domain	No
Q9CR75	TNFRSF12A	tumor necrosis factor receptor superfa	No
Q8BFY9	TNPO1	transportin 1	Yes
Q8CH72	TRIM32	tripartite motif-containing 32	Yes
Q8BUH1	TXNL4B	thioredoxin-like 4B	No
P26369	U2AF2	U2 small nuclear ribonucleoprotein au	Yes
P0CG50	UBC	ubiquitin C	Yes
Q9EPU0	UPF1	UPF1 regulator of nonsense transcript	Yes
10R2	UPF2	UPF2 regulator of nonsense transcript	No
Q00731	VEGFA	vascular endothelial growth factor A	No
Q99J09	WDR77	WD repeat domain 77	Yes
P27641	XRCC5	X-ray repair complementing defective	Yes
E9Q0B8;P23475	XRCC6	X-ray repair complementing defective	Yes
P62960	YBX1	Y box protein 1	Yes
Q9Z2C8	YBX2	Y box protein 2	No
Q9CQV8	YWHAB	tyrosine 3-monooxygenase/tryptophan	Yes
P63101	YWHAZ	tyrosine 3-monooxygenase/tryptophan	Yes
P22893	ZFP36	zinc finger protein 36	No

P23950	ZFP36L1	zinc finger protein 36, C3H type-like 1	No
P63101	YWHAZ	tyrosine 3-monooxygenase/tryptophan	Yes
P22893	ZFP36	zinc finger protein 36	No
P23950	ZFP36L1	zinc finger protein 36, C3H type-like 1	No

d their 1st protein-protein interaction neighbours

Enriched key GO terms
positive regulation of neurogenesis
mRNA processing;RNA processing;mRNA metabolic process
mRNA metabolic process
mRNA processing;RNA processing;mRNA metabolic process
mRNA metabolic process
mRNA processing;RNA processing;mRNA metabolic process
positive regulation of neurogenesis
regulation of mRNA stability;regulation of RNA stability;mRNA metabolic process
mRNA stabilization;RNA stabilization;regulation of mRNA stability;regulation of RNA stability;mRNA p
mRNA processing;RNA processing;mRNA metabolic process
positive regulation of neurogenesis
mRNA metabolic process
mRNA processing;RNA processing;mRNA metabolic process
mRNA processing;RNA processing;mRNA metabolic process
positive regulation of neurogenesis
mRNA processing;RNA processing;mRNA metabolic process
mRNA processing;RNA processing;mRNA metabolic process
mRNA processing;RNA processing;mRNA metabolic process
mRNA processing;RNA processing;mRNA metabolic process
mRNA processing;RNA processing;mRNA metabolic process
mRNA processing;RNA processing;mRNA metabolic process
mRNA processing;RNA processing;mRNA metabolic process
mRNA processing;RNA processing;mRNA metabolic process
mRNA stabilization;RNA stabilization;regulation of mRNA stability;regulation of RNA stability;mRNA m
mRNA processing;RNA processing;mRNA metabolic process
mRNA processing;RNA processing;mRNA metabolic process
mRNA processing;RNA processing;mRNA metabolic process
mRNA processing;RNA processing;mRNA metabolic process
mRNA processing;RNA processing;mRNA metabolic process
mRNA processing;RNA processing;mRNA metabolic process
mRNA processing;RNA processing;mRNA metabolic process
mRNA metabolic process
mRNA processing;RNA processing;mRNA metabolic process
RNA processing
mRNA processing;RNA processing;mRNA metabolic process
mRNA processing;RNA processing;mRNA metabolic process
mRNA processing;RNA processing;mRNA metabolic process
mRNA metabolic process
mRNA processing;RNA processing;mRNA metabolic process
CRD-mediated mRNA stabilization;mRNA stabilization;RNA stabilization;regulation of mRNA stability;
RNA processing;mRNA metabolic process
mRNA metabolic process
mRNA stabilization;RNA stabilization;regulation of mRNA stability;regulation of RNA stability;mRNA m
mRNA metabolic process
mRNA processing;RNA processing;mRNA metabolic process
mRNA processing;RNA processing;mRNA metabolic process
mRNA processing;RNA processing;mRNA metabolic process
mRNA processing;RNA processing;mRNA metabolic process
mRNA processing;RNA processing;mRNA metabolic process
mRNA stabilization;RNA stabilization;regulation of mRNA stability;regulation of RNA stability;mRNA m

mRNA processing;RNA processing;mRNA metabolic process
positive regulation of neurogenesis
mRNA processing;RNA processing;mRNA metabolic process
RNA processing;mRNA metabolic process
RNA processing;mRNA metabolic process
mRNA processing;RNA processing;mRNA metabolic process
mRNA processing;RNA processing;mRNA metabolic process
mRNA processing;RNA processing;mRNA metabolic process
positive regulation of neurogenesis
mRNA processing;RNA processing;mRNA metabolic process
mRNA processing;RNA processing;mRNA metabolic process
mRNA processing;RNA processing;mRNA metabolic process
mRNA stabilization;RNA stabilization;regulation of mRNA stability;regulation of RNA stability;mRNA p
mRNA processing;RNA processing;mRNA metabolic process
mRNA processing;RNA processing;mRNA metabolic process
mRNA processing;RNA processing;mRNA metabolic process
mRNA processing;RNA processing;mRNA metabolic process
mRNA processing;RNA processing;mRNA metabolic process
mRNA processing;RNA processing;mRNA metabolic process
CRD-mediated mRNA stabilization;mRNA stabilization;RNA stabilization;regulation of mRNA stability;
mRNA metabolic process
mRNA metabolic process
mRNA metabolic process
positive regulation of neurogenesis
mRNA processing;RNA processing;mRNA metabolic process
mRNA processing;RNA processing;mRNA metabolic process
mRNA processing;RNA processing;mRNA metabolic process
mRNA processing;RNA processing;mRNA metabolic process
mRNA processing;RNA processing;mRNA metabolic process
mRNA processing;RNA processing;mRNA metabolic process
positive regulation of neurogenesis
mRNA metabolic process
mRNA metabolic process
mRNA stabilization;RNA stabilization;regulation of mRNA stability;regulation of RNA stability;mRNA m
positive regulation of neurogenesis
mRNA processing;RNA processing;mRNA metabolic process
mRNA stabilization;RNA stabilization;regulation of mRNA stability;regulation of RNA stability;mRNA m
mRNA processing;RNA processing;mRNA metabolic process
mRNA metabolic process
mRNA processing;RNA processing;mRNA metabolic process
mRNA processing;RNA processing;mRNA metabolic process
positive regulation of neurogenesis
mRNA processing;RNA processing;mRNA metabolic process
mRNA processing;RNA processing;mRNA metabolic process
mRNA stabilization;RNA stabilization;regulation of mRNA stability;regulation of RNA stability;mRNA p
mRNA processing;RNA processing;mRNA metabolic process
mRNA processing;RNA processing;mRNA metabolic process
mRNA metabolic process
mRNA processing;RNA processing;mRNA metabolic process
mRNA processing;RNA processing;mRNA metabolic process
positive regulation of neurogenesis
mRNA processing;RNA processing;mRNA metabolic process
RNA processing;mRNA metabolic process
RNA processing;mRNA metabolic process
mRNA metabolic process

mRNA processing;RNA processing;mRNA metabolic process
mRNA metabolic process
mRNA metabolic process
mRNA processing;RNA processing;mRNA metabolic process
positive regulation of neurogenesis;mRNA processing;RNA processing;mRNA metabolic process
mRNA processing;RNA processing;mRNA metabolic process
mRNA processing;RNA processing;mRNA metabolic process
mRNA processing;RNA processing;mRNA metabolic process
mRNA processing;RNA processing;mRNA metabolic process
mRNA metabolic process
mRNA metabolic process
mRNA metabolic process
mRNA metabolic process
mRNA metabolic process
mRNA metabolic process
mRNA metabolic process
mRNA metabolic process
mRNA metabolic process
mRNA metabolic process
mRNA metabolic process
mRNA metabolic process
mRNA metabolic process
mRNA metabolic process
mRNA metabolic process
mRNA metabolic process
mRNA metabolic process
mRNA metabolic process
mRNA processing;RNA processing;mRNA metabolic process
mRNA processing;RNA processing;mRNA metabolic process
mRNA processing;RNA processing;mRNA metabolic process
mRNA processing;RNA processing;mRNA metabolic process
mRNA processing;RNA processing;mRNA metabolic process
mRNA processing;RNA processing;mRNA metabolic process
mRNA processing;RNA processing;mRNA metabolic process
mRNA stabilization;RNA stabilization;regulation of mRNA stability;regulation of RNA stability;mRNA p
mRNA processing;RNA processing;mRNA metabolic process
mRNA processing;RNA processing;mRNA metabolic process
mRNA processing;RNA processing;mRNA metabolic process
regulation of mRNA stability;regulation of RNA stability;mRNA metabolic process
positive regulation of neurogenesis
positive regulation of neurogenesis
mRNA processing;RNA processing;mRNA metabolic process
RNA processing;mRNA metabolic process
mRNA metabolic process
mRNA metabolic process
RNA processing;mRNA metabolic process
mRNA metabolic process
mRNA processing;RNA processing;mRNA metabolic process
positive regulation of neurogenesis
mRNA processing;RNA processing;mRNA metabolic process
regulation of mRNA stability;regulation of RNA stability;mRNA metabolic process
positive regulation of neurogenesis
mRNA metabolic process
RNA processing
mRNA processing;RNA processing;mRNA metabolic process

mRNA processing;RNA processing;mRNA metabolic process
mRNA processing;RNA processing;mRNA metabolic process
mRNA processing;RNA processing;mRNA metabolic process
positive regulation of neurogenesis
mRNA processing;RNA processing;mRNA metabolic process
mRNA stabilization;RNA stabilization;regulation of mRNA stability;regulation of RNA stability;mRNA m
mRNA processing;RNA processing;mRNA metabolic process
mRNA metabolic process
mRNA metabolic process
mRNA metabolic process
mRNA processing;RNA processing;mRNA metabolic process
mRNA processing;RNA processing;mRNA metabolic process
mRNA processing;RNA processing;mRNA metabolic process
mRNA processing;RNA processing;mRNA metabolic process
mRNA processing;RNA processing;mRNA metabolic process
mRNA processing;RNA processing;mRNA metabolic process
mRNA processing;RNA processing;mRNA metabolic process
mRNA processing;RNA processing;mRNA metabolic process
mRNA processing;RNA processing;mRNA metabolic process
mRNA processing;RNA processing;mRNA metabolic process
mRNA processing;RNA processing;mRNA metabolic process
positive regulation of neurogenesis
mRNA processing;RNA processing;mRNA metabolic process
mRNA processing;RNA processing;mRNA metabolic process
mRNA processing;RNA processing;mRNA metabolic process
mRNA processing;RNA processing;mRNA metabolic process
mRNA processing;RNA processing;mRNA metabolic process
mRNA processing;RNA processing;mRNA metabolic process
mRNA processing;RNA processing;mRNA metabolic process
mRNA processing;RNA processing;mRNA metabolic process
mRNA processing;RNA processing;mRNA metabolic process
mRNA processing;RNA processing;mRNA metabolic process
RNA processing;mRNA metabolic process
mRNA processing;RNA processing;mRNA metabolic process
CRD-mediated mRNA stabilization;mRNA stabilization;RNA stabilization;regulation of mRNA stability;
mRNA stabilization;RNA stabilization;regulation of mRNA stability;regulation of RNA stability;mRNA p
mRNA processing;RNA processing;mRNA metabolic process
mRNA stabilization;RNA stabilization;regulation of mRNA stability;regulation of RNA stability;mRNA m
positive regulation of neurogenesis
mRNA metabolic process
positive regulation of neurogenesis
mRNA processing;RNA processing;mRNA metabolic process
mRNA processing;RNA processing;mRNA metabolic process
mRNA metabolic process
mRNA metabolic process
mRNA metabolic process
mRNA stabilization;RNA stabilization;regulation of mRNA stability;regulation of RNA stability;mRNA m
mRNA processing;RNA processing;mRNA metabolic process
positive regulation of neurogenesis
positive regulation of neurogenesis
CRD-mediated mRNA stabilization;mRNA stabilization;RNA stabilization;regulation of mRNA stability;
mRNA stabilization;RNA stabilization;regulation of mRNA stability;regulation of RNA stability;mRNA m
mRNA metabolic process
mRNA metabolic process
regulation of mRNA stability;regulation of RNA stability;mRNA processing;RNA processing;mRNA me

regulation of mRNA stability;regulation of RNA stability;mRNA metabolic process
mRNA metabolic process
regulation of mRNA stability;regulation of RNA stability;mRNA processing;RNA processing;mRNA me
regulation of mRNA stability;regulation of RNA stability;mRNA metabolic process

rocessing;RNA processing;mRNA metabolic process

ıetabolic process

ıregulation of RNA stability;mRNA processing;RNA processing;mRNA metabolic process

ıetabolic process

ıetabolic process

rocessing;RNA processing;mRNA metabolic process

regulation of RNA stability;mRNA processing;RNA processing;mRNA metabolic process

netabolic process

netabolic process

rocessing;RNA processing;mRNA metabolic process

rocessing;RNA processing;mRNA metabolic process

ıetabolic process

ıregulation of RNA stability;mRNA processing;RNA processing;mRNA metabolic process
rocessing;RNA processing;mRNA metabolic process

ıetabolic process

ıetabolic process

ıregulation of RNA stability;mRNA processing;RNA processing;mRNA metabolic process
ıetabolic process

ɛtabolic process

etabolic process
